# Hydrocyclization/Defluorination of CF_3_‐Substituted Acrylamides: Insights from Kinetics of Hydrogen Atom Transfer

**DOI:** 10.1002/advs.202501799

**Published:** 2025-04-25

**Authors:** Yanjun Wan, Ronghui Shao, Jack R. Norton

**Affiliations:** ^1^ College of Pharmaceutical Sciences Zhejiang University of Technology Hangzhou 310014 China; ^2^ Department of Chemistry Columbia University 3000 Broadway New York NY 10027 USA; ^3^ College of Chemistry Naikai University Tianjin 300071 China

**Keywords:** defluorination, *gem*‐difluoroalkenes, hydrocyclization, hydrogen atom (hydride) transfer, nickel hydride

## Abstract

The introduction of F‐containing groups into organic molecules can significantly alter their physical and chemical properties. Particularly, *gem*‐difluoroalkenes serve as versatile precursors for a broad variety of organofluorine compounds, commonly used in agrochemicals, pharmaceuticals, and materials science. Based on the kinetics of H• transfer to acrylamide (*k*_H_ = 2.28 × 10^−4^ m ^−1^ s^−1^ at 300 K in toluene), the study describes a nickel‐hydride‐(or Li[BEt_3_H]) initiated hydrocyclization/defluorination of CF_3_‐substituted acrylamides, offering alternative access to 4‐fluorovinyl‐substituted 2‐pyrrolidones (Seletracetam derivatives that are antiepileptic drug candidates). This process proceeds with high yields and remarkable chemo‐ and regioselectivity. The hydrocyclization/defluorination can be initiated by either H• or H^–^ transfer, followed by a *5‐exo‐trig* cyclization and subsequent fluorine elimination. The strategy has been applied in the late‐stage functionalization of drug molecules, providing a valuable tool in the synthesis of pharmaceutical compounds.

## Graphical abstract

The study develops a H•/H^–^ transfer strategy enabling efficient hydrocyclization/defluorination of CF_3_‐acrylamides to synthesize Seletracetam derivatives with *gem*‐difluoroalkenes. Mechanistic studies reveal distinct pathways: the Ni^II^‐H/PhSiH_3_ system operates through a hydrogen atom transfer process, while the 1,4‐Michael addition of H^–^ appears to be responsible for the Li[BEt_3_H] system.

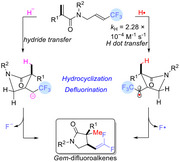

## 1 Introduction

**Scheme 1:**
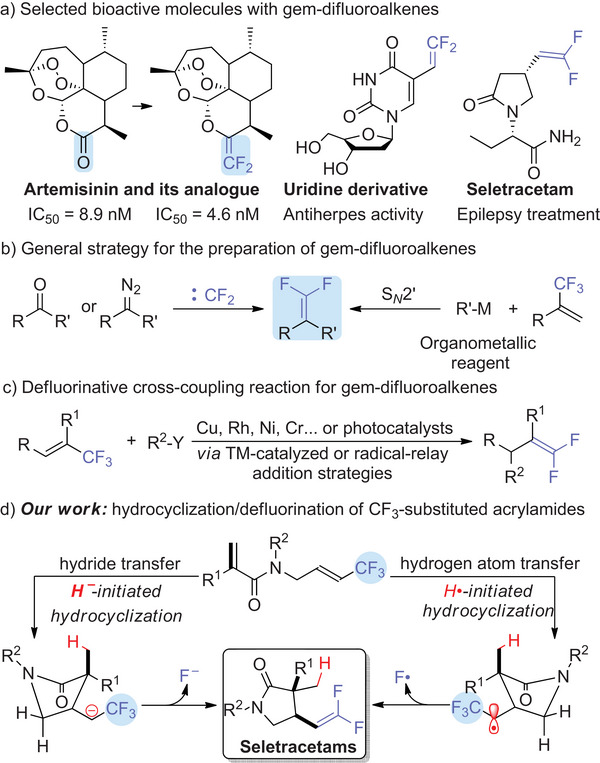
Representative bioactive molecules containing *gem*‐difluoroalkenyl moiety and its synthetic strategies.

Incorporation of fluorine or fluorinated moieties into organic molecules often enhances their lipophilicity, solubility, metabolic stability, and dipole moment.^[^ ^1^, ^2^, ^3^, ^4^ ^]^ Among fluorine‐containing functional groups, *gem*‐difluoroalkenes are particularly versatile precursors, enabling the synthesis of a wide range of organofluorine compounds,^[^ ^5^, ^6^, ^7^, ^8^ ^]^ including numerous biologically active molecules (**Scheme** **1**a). The group is also well‐established as bioisosteres of carbonyl compounds, offering increased metabolic stability and, consequently, improved pharmaceutical performance.^[^ ^9^, ^10^, ^11^ ^]^ A common synthetic strategy to mitigate in vivo metabolism involves replacing carbonyl groups in drug candidates with *gem*‐difluoroalkene moieties. For example, Seletracetam, designed to mimic the anti‐epileptic drug Levetiracetam, shows enhanced binding affinity for its target, superior efficacy, and improved CNS tolerability, thanks to the introduction of the *gem*‐difluorovinyl group.^[^ ^12^, ^13^, ^14^ ^]^

The general approach to the preparation of *gem*‐difluoroalkenes often relies on functional group interconversion, such as the difluoromethylenation of carbonyls or diazo compounds or hydrazones through the Wittig reaction, and the defluorination of trifluoromethylalkenes by strong nucleophiles.^[^ ^15^, ^16^, ^17^, ^18^, ^19^, ^20^ ^]^ However, these methods typically involve organometallic reagents (Grignard reagents or organolithium reagents), highly reactive intermediates, or harsh reaction conditions, thus limiting their substrate scope. Catalytic strategies for the synthesis of *gem*‐difluoroalkenes are thus in high demand, and recent years have seen significant advances in the development of efficient methods for constructing these structures (Scheme 1b).

Transition‐metal‐catalyzed nucleophilic attack on trifluoromethyl alkenes provides alternative access to *gem*‐difluoroalkenes via intermolecular cross‐coupling and subsequent defluorination. Mechanistic studies have demonstrated the high electrophilicity of trifluoromethyl vinyls, resulting in Rh‐, Cu‐ and Ni‐catalyzed asymmetric defluorinative arylations, alkylations, allylations, borylations, and silylations.^[^ ^21^, ^22^, ^23^, ^24^, ^25^, ^26^, ^27^, ^28^, ^29^, ^30^ ^]^ Another promising strategy involves radicals, which allow the defluorination of trifluoromethyl groups to be achieved through either photocatalysis or Ni catalysis.^[^ ^31^, ^32^, ^33^, ^34^, ^35^, ^36^ ^]^ These methods are initiated with the addition of radicals to CF_3_‐substituted alkenes; *β*‐fluorine elimination then gives alkylated,^[^ ^37^, ^38^, ^39^, ^40^ ^]^ arylated,^[^ ^41^ ^]^ borylated,^[^ ^42^ ^]^ or hydrogenated^[^ ^43^ ^]^ *gem*‐difluoroalkenes. Hydrodefluorination, the simplest such transformation, has garnered significant attention.^[^ ^43^, ^44^, ^45^, ^46^, ^47^, ^48^ ^]^ For example, in 2020, our group reported a pincer nickel‐hydride‐catalyzed hydrodefluorination of *α*‐CF_3_ styrenes that began with radicals generated by hydrogen atom transfer (HAT) (Scheme 1c).^[^ ^43^ ^]^

We have generated many carbon‐centered radicals through such H• transfers to olefins,^[^ ^49^, ^50^, ^51^ ^]^ and have demonstrated that H• transfer to *α*‐substituted acrylate esters can initiate radical cyclizations.^[^ ^52^ ^]^ We have measured the rate constants for H• transfer to phenyl‐, alkyl‐, and carbomethoxy‐substituted olefins from CpCr(CO)_3_H. Now, we have transferred hydrogen atoms (H•), or added hydrides (H^–^), to the terminal C═C of CF_3_‐substituted acrylamides. This addition results in cyclization and defluorination, giving Seletracetam derivatives — *γ*‐lactams that feature a *gem*‐difluoroalkene moiety (Scheme 1d, hydrocyclization/defluorination process); meanwhile, the formation of a methyl group at the C3 position of Seletracetam likely enhances its biological activity and physical properties.^[^ ^53^ ^]^

## 2 Discussion and Results

### 2.1 Kinetic Investigation of H• Transfer to Acrylamides

**Scheme 2:**
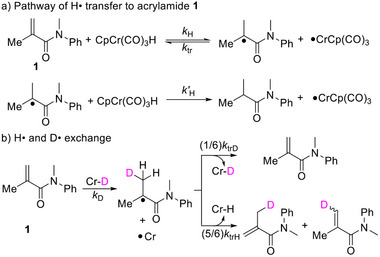
Pathways for the reaction of methacrylamide **1** with CpCr(CO)_3_H and CpCr(CO)_3_D respectively.

We began by measuring the rate constant for the transfer of H• from CpCr(CO)_3_H to methacrylamide **1**. Treatment of **1** with an excess of CpCr(CO)_3_H in toluene at room temperature gave only a trace of the hydrogenation product, implying that *k′*_H_ is much smaller than *k*_tr_ as shown in **Scheme** **2**a.

**Figure 1:**
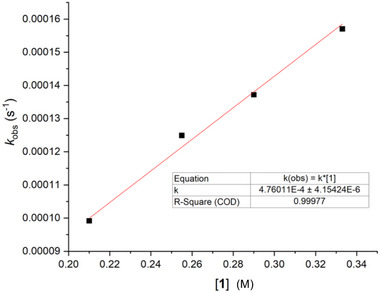
Plot of *k*_obs_ versus [**1**], for H• transfer at 300 K in toluene‐*d*_8_.

In the presence of CpCr(CO)_3_D, a large excess (>10 equiv) of **1** readily undergoes H/D exchange (Scheme 2b), with the hydride signal from CpCr(CO)_3_H appearing in the ^1^H NMR spectrum. By monitoring the growth of the Cr‐H signal over time, we obtained the pseudo‐first‐order rate constant *k*_obs_, which is linearly dependent on the concentration of **1** (**Figure** **1**). This observation confirmed that the H/D exchange between **1** and Cr‐D follows second‐order kinetics (Equation (1)).

(1) $$\frac{d\left[\mathrm{CrH}\right]}{dt}=k\left[1\right]\;\left[\mathrm{CrD}\right]=k_{\mathrm{obs}}\;\left[\mathrm{CrD}\right]$$

The value of *k* reflects both the rate constant *k*_D_ for D• abstraction from Cr‐D and the fractional probability that H• will return to Cr• after the initial D• transfer (the details are given in the Section S2.4, Supporting Information). From Figure 1, *k* is 4.76 × 10^−4^ m ^−1^ s^−1^ at 300 K, and *k*_D_ is therefore 5.08 × 10^−4^ m ^−1^ s^−1^ at that temperature. Application of the established H/D kinetic isotope effect for C to Cr transfer^[^ ^54^ ^]^ implies that *k*_H_ is 2.28 × 10^−4^ m ^−1^ s^−1^ at 300 K. Thus, acrylamides are reasonable acceptors for H•, as previously reported for their reaction with cobalt‐hydride.^[^ ^55^ ^]^

### 2.2 Optimization Studies

We then considered whether HAT could be used to trigger the hydrocyclization/defluorination of *CF_3_*‐*substituted* acrylamides. CpCr(CO)_3_H proved unable to initiate the hydrodefluorination of *α*‐CF_3_ styrenes,^[^ ^43^ ^]^ although the iso‐PmBox Ni^II^‐H (**1a**)/silane system, developed by the Gade group, is well established as highly effective for such transformations.^[^ ^56^, ^57^, ^58^, ^59^, ^60^ ^]^ The Ni^I^ from such an Ni^II^‐H can abstract an X• from an organic halide, forming a Ni^II^ halide, which can, in turn, react with a silane or a boron hydride to regenerate the Ni^II^‐H.

We began by treating (*E*)‐*N*,2‐diphenyl‐*N*‐(4,4,4‐trifluorobut‐2‐en‐1‐yl)acrylamide **2a** with 1.0 equiv of phenylsilane (PhSiH_3_) in the presence of 10 mol.% Ni^II^‐H catalyst **1a** in toluene (0.2 m) under argon at room temperature for 12 h. The *gem*‐difluorovinyl‐substituted pyrrolidin‐2‐one **3a** was obtained in 93% yield with excellent diastereoselectivity (*cis*/*trans* = 11:1) (Entry 1, **Table** **1**). (A preference for the *cis* product is well established for 5‐*exo* radical cyclizations.)^[^ ^61^, ^62^, ^63^ ^]^ The reaction yielded a similar result when the amount of PhSiH_3_ was reduced to 0.5 equiv (Entry 2). A slightly decreased yield (85% or 88%) was obtained when the reaction was conducted at a lower concentration (0.1 m) or catalyzed by a smaller amount of (5 mol.%) of the **1a** (Entries 3 and 4). As shown in Entry 5, catalyst **1b** resulted in product **3a** in 68% yield with a modest diastereoselectivity (8:1). In contrast, replacing catalyst **1a** with a catalytic system comprising 10 mol.% Ni(OAc)_2_•4H_2_O and the ligand **L1** (**L2**) failed to generate any detectable product (Entries 6 and 7). Other hydrides, ((EtO)_3_SiH, Et_3_SiH, *t*BuNH_2_BH_3_, and LiBEt_3_H) proved less effective, although a stoichiometric amount of Li[BEt_3_H] was able to give **3a** (82%) in the absence of 1a (Entries 8–11). (However, the diastereoselectivity (*cis*/*trans* = 2:1) was poor.) Presumably the carbanion formed by the 1,4‐Michael addition of a hydride to **2a** can cyclize onto the electrophilic CF_3_‐substituted C═C. Importantly, such a hydride transfer process has expanded the methodology for the synthesis of *gem*‐difluoroalkenes. Through comparative analysis of both reaction systems, we conclude that the nickel catalyst plays a critical role in the stereochemical control during the fluorine atom abstraction (Scheme 4d). Additionally, changing solvents did not improve the yield (Entries 12–14). The use of methanol altered the reaction pathway, giving the hydrogenation product in high yield (73%).

**Table 1:** Optimization of the reaction conditions.

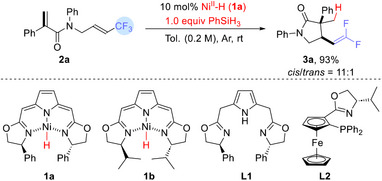		
Entry	Deviation from standard conditions^a)^	Yield^b)^
1	**2a** (0.1 mmol), 10 mol.% Ni^II^‐H (**1a**), PhSiH_3_ (0.1 mmol), in toluene (0.2 m) under Ar at room temperature for 12 hours	93% (11:1)
2	0.5 equiv. of PhSiH_3_ instead of 1 equiv. of PhSiH_3_	92% (11:1)
3	0.1 m instead of 0.2 m	85% (7.5:1)
4	5 mol.% **1a** instead of 10 mol.% **1a**	88% (10:1)
5	10 mol.% **1b** instead of 10 mol.% **1a**	68% (8:1)
6	10 mol.% Ni(OAc)_2_•4H_2_O and **L1** instead of **1a**	‐
7	10 mol.% Ni(OAc)_2_•4H_2_O and **L2** instead of **1a**	‐
8	1.0 equiv. of (EtO)_3_SiH instead of PhSiH_3_	75% (11:1)
9	1.0 equiv. of Et_3_SiH instead of PhSiH_3_	Trace
10	1.0 equiv. of *t*BuNH_2_BH_3_ instead of PhSiH_3_	68% (6:1)
11	1.0 equiv. of Li[BEt_3_H]^c)^ without **1a** for 0.5 hours	82% (2:1)
12	THF instead of toluene	70% (10:1)
13	MeCN instead of toluene	Trace
14	MeOH instead of toluene	Trace

^a)^ Standard conditions: substrate **2a** (0.1 mmol), 10 mol.% Ni^II^‐H (**1a**), PhSiH_3_ (0.1 mmol), in toluene (0.2 m) under Ar at room temperature for 12 hours;

^b)^ Yield and diastereoselectivity were determined by ^19^F NMR of its crude reaction using fluorobenzene as an internal standard;

^c)^ 1.0 m Li[BEt_3_H] in THF.

### 2.3 Substrate Scope of the Reaction

We then examined the applicability of our hydrocyclization/defluorination reaction over a range of substrates (**Table** **2**). Both electron‐donating and electron‐withdrawing substituents worked, as well as different substitution patterns on the aromatic ring (R^1^ group). The products, *gem*‐difluorovinyl‐substituted pyrrolidin‐2‐ones (**3a**–**3e**, **3i,** and **3j**), were formed in excellent yields (88% – 98%) with good diastereoselectivities (*cis*/*trans* > 5:1). The observed selectivity difference between *para*‐electron‐withdrawing (**3e**, **3i** and **3j**) and electron‐donating (**3c** and **3d**) substrates stems presumably from their distinct influences on the cyclization transition state. When the R^1^ was replaced with ClC_6_H_4_‐ (**2f**) or BrC_6_H_4_‐ (**2g** and **2h**), the Ni^II^‐H/PhSiH_3_ system no longer worked. Previous research has demonstrated that a Ni(I) species can abstract a halogen atom (Br, Cl) from an aryl halide via XAT (halogen atom transfer) process to generate a Ni^II^‐aryl complex, and a Ni^II^‐X (Br, Cl) species, which are unable to regenerate Ni^II^‐H (**1a**) catalyst from PhSiH_3_.^[^ ^57^, ^58^ ^]^

**Table 2:** Substrate scope of the H•/H^–^‐enabled hydrocyclization/defluorination of acrylamides.

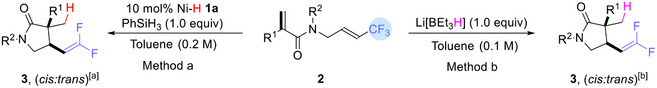
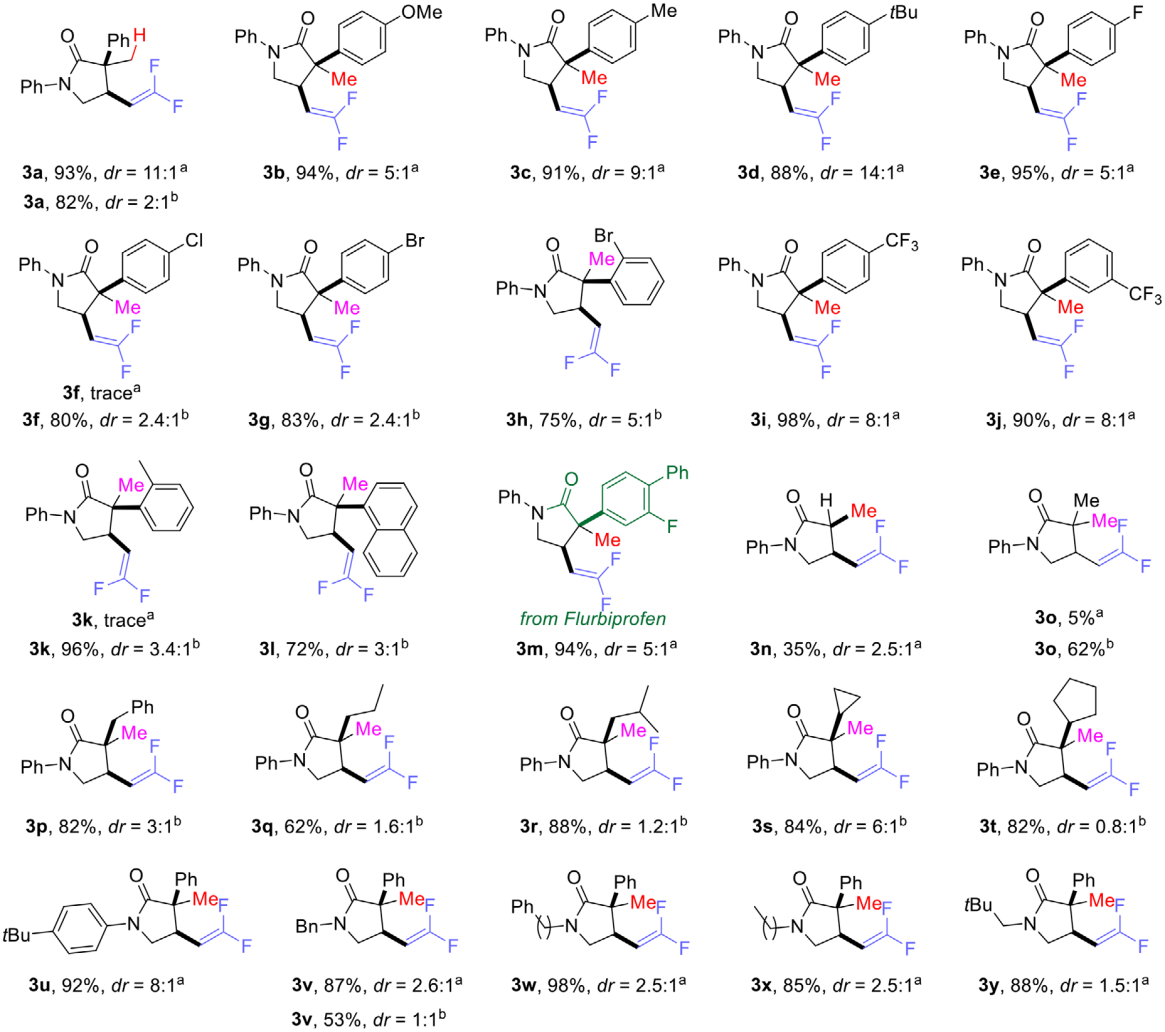

^a)^ Substrate **2** (0.1 mmol), PhSiH_3_ (1.0 equiv, 0.1 mmol), and 10 mol.% **1a** in toluene (0.5 mL) under argon at room temperature for 12 hours;

^b)^ Substrate **2** (0.1 mmol), and Li[BEt_3_H] (0.10 mmol) in toluene (1 mL) under argon at room temperature for 0.5 hours;

^c)^ Yields and diastereoselectivities were determined by ^19^F NMR using fluorobenzene as an internal standard, unless noted.

To our delight, Li[BEt_3_H] proved to be an effective hydride source, presumably by a Michael addition to the terminal C═C of substrates (**2f**–**2** **h**), as illustrated in Table 2. The desired products **3f**–**3h** were obtained in good yields, although with diastereoselectivities lower than those obtained with the Ni^II^‐H/PhSiH_3_ system. Substrates bearing 2‐MeC_6_H_4_ (**2k**) or naphthyl (**2l**) were not compatible with the Ni^II^‐H/PhSiH_3_ system, presumably because of steric congestion. Li[BEt_3_H] also carried out the hydrocyclization/defluorination, giving good yields of **3k** and **3l**.

The increasing importance of *gem*‐difluoroalkenes in agrochemical and medicinal chemistry makes it desirable to functionalize pharmaceutically relevant molecules at a late stage in their synthesis. The Ni^II^‐H/PhSiH_3_ system proved effective in this regard; for example, the Flurbiprofen derivative **2** **m** was functionalized via H• transfer to give the product **3** **m** in 95% yield with reasonable diastereoselectivity (*cis*/*trans* = 5:1). In contrast, the yield dramatically decreased (35%) when there was no substituent on the terminal olefin (**3n**, R^1^ = H) presumably because the radical intermediate was not appropriately stabilized.

Substrates (**2o**‐**2t**) with aliphatic substituents, R^1^ = methyl, benzyl, propyl, isobutyl, cyclopropyl or cyclopentyl, did not react with the Ni^II^‐H catalyst, presumably because the Ni^II^‐H is not able to transfer H• to **2o**‐**2t**. In other words, the slowest step in our reaction is the H• transfer to the terminal C═C of substrate.^[^ ^49^ ^]^ The H^–^ transfer effectively addressed this issue, leading to **3o**‐**3t** in good yields. It was obvious that aryl protecting groups on nitrogen (**2a** and **2u**) offered higher diastereoselectivities (e.g., 8:1 in **3u**) than alkyl protecting groups **2v**‐**2y** (e.g., 1.5:1 to 2.6:1 in **3v**‐**3y**) with Ni^II^‐H/PhSiH_3_. Other substrates such as **S1**, **S2**, and **S3** (See details in Supporting Information) proved ineffective for the transformation in either the Ni‐H/PhSiH₃ system or the LiBEt₃H system.

### 2.4 Synthetic Applications

**Scheme 3:**
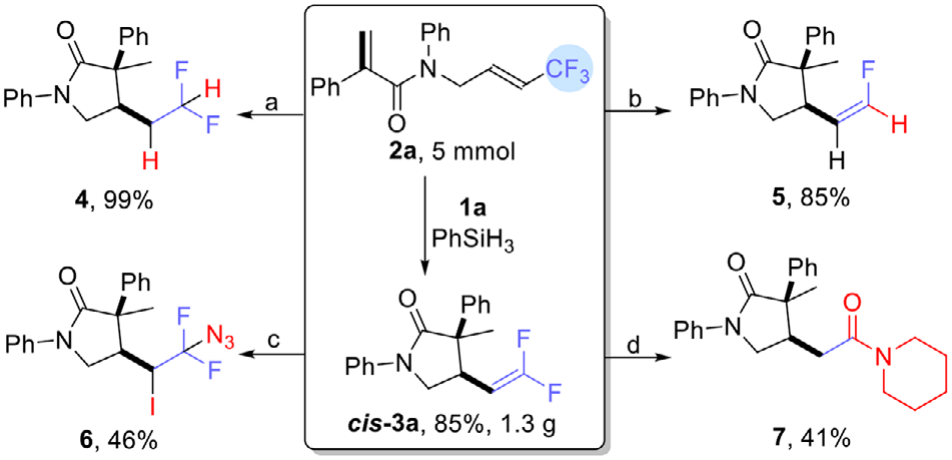
Synthetic applications of ***cis*‐3a**. a) Hydrogenation of ***cis*‐3a** with Pd/C catalyst. b) Synthesis of *Z*‐Fluoroalkenes. c) Difunctionalization of ***cis*‐3a**. d) Dehydrofluorination and hydrolysis of ***cis*‐3a**. See more details in the Section S2.3 (Supporting Information).

We were able to perform a gram‐scale reaction of **2a** (5 mmol) with PhSiH_3_ (2.5 mmol) in the presence of 5 mol.% Ni^II^‐H catalyst **1a**, giving ***cis***‐**3a** in 85% yield (1.3 g) with no loss of diastereoselectivity (**Scheme** **3**). The further transformations in Scheme 3 were readily carried out. The *gem*‐difluoro C═C of ***cis***‐**3a** was easily reduced with a Pd/C catalyst under H_2_, affording the hydrogenated product **4** in a quantitative yield. A modification of the previously reported Cu‐catalyzed hydrodefluorination of *gem*‐difluoroalkenes^[^ ^64^ ^]^ converted ***cis***‐**3a** into the *Z*‐monofluoroalkene **5** in 85% yield. The CsF‐promoted difunctionalization^[^ ^36^ ^]^ of ***cis***‐**3a** with TMSN_3_ and I_2_ gave the azide‐substituted **6** in 46% yield. Finally, the addition of morpholine to ***cis***‐**3a**, followed by dehydrofluorination and hydrolysis,^[^ ^65^ ^]^ gave the amide **7** (41%).

### 2.5 Mechanistic Investigation

**Scheme 4:**
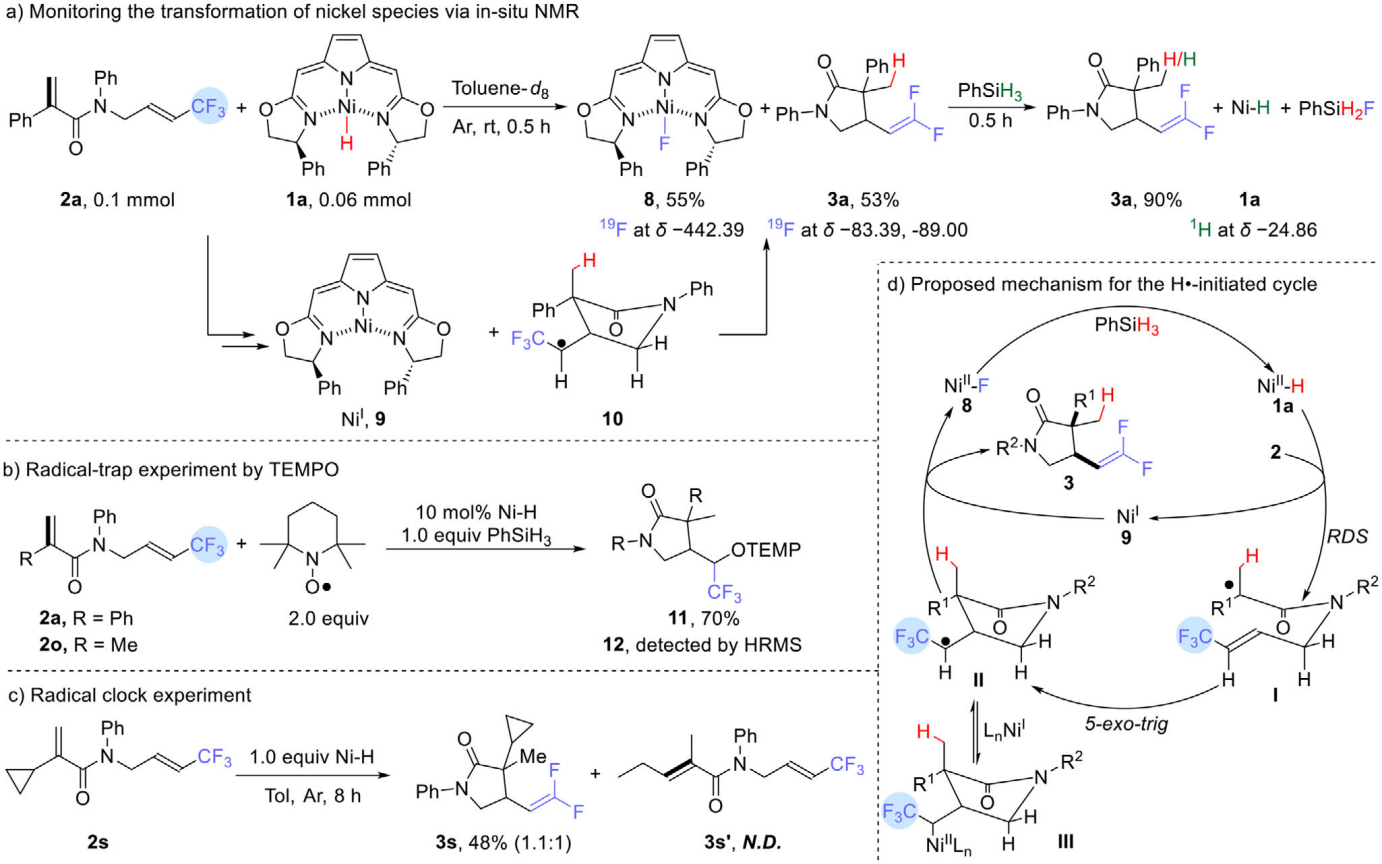
Mechanistic studies of the Ni‐H/PhSiH_3_ system for the hydrocyclization/defluorination.

To understand the mechanistic difference between the Ni^II^‐H/PhSiH_3_ system and the Li[BEt_3_H] system, the mechanistic experiments in **Scheme** **4** have been carried out. We have monitored the reaction of 0.06 mmol Ni^II^‐H **1a** with an excess of the substrate **2a** (0.1 mmol) in toluene‐*d*_8_ by ^19^F NMR (Scheme 4a). The signal of **2a** (a ^19^F at *δ* −63.21 relative to CFCl_3_) decreased as the ^19^F signals of **3a** appeared at *δ* −83.39, −89.00, while the ^19^F NMR resonance of the known Ni^II^‐F complex **8** appeared at *δ* −442.39.^[^ ^43^, ^59^ ^]^ The addition of 0.05 mmol PhSiH_3_ caused the ^19^F peaks of **8** and **2a** to disappear, and the ^1^H NMR signal for Ni^II^‐H **1a** to reappear at *δ* −24.86. Presumably the transfer of H• from Ni^II^‐H (**1a**) to **2a** gave the Ni^I^ complex **9**, and the Ni^I^ then removed a fluorine atom from the intermediate **10** to give **8**; the PhSiH_3_ then regenerated the Ni^II^‐H (**1a**).

We then attempted to trap a radical intermediate in this reaction. In the presence of TEMPO (0.2 mmol), we obtained the cyclized TEMPO‐adduct **11** in 70% yield from 0.1 mmol **2a** (TEMPO‐adduct **12** from **2o** was detected by HRMS) (Scheme 4b) — proving that a radical reaction can be initiated by H• transfer to the terminal C═C of acrylamides. A ring‐opening experiment was performed using cyclopropyl‐substituted substrate **2s** and the Ni–H species **1a** (1.0 equiv) in Scheme 4c. The reaction afforded product **3s** in 48% yield (*dr* = 1.1:1), with no detectable ring‐opening product **3s′**. While the unsubstituted cyclopropylcarbinyl radical undergoes ring‐opening at 10^8^ s^−1^ at room temperature,^[^ ^66^ ^]^ the carboxamido substituent presumably lowers the rate of this ring opening relative to the competing *5‐exo‐trig* cyclization (about 10^5^ to 10^7^ s^−1^).^[^ ^67^ ^]^ Sufficiently stabilizing substituents at the α position of cyclopropylcarbinyl radicals are know to makes their ring‐opening reversible, and to make the ring‐closed form strongly favored at equilibrium.^[^ ^68^ ^]^ Our observations agree well with prior reports that cyclopropylcarbinyl radicals with stabilizing substituents open at rates orders of magnitude slower than the unsubstituted systems.^[^ ^67^ ^]^

The proposed mechanism for the Ni^II^‐H/PhSiH_3_ is shown in Scheme 4d. The transfer of the H• from **1a** to **2a** (the rate‐determining step, RDS) forms the radical intermediate **I** and leaves the Ni^I^ complex **9**. The *5‐exo‐trig* cyclization of **I** gives **II**, with the bulky group (R^1^) in a pseudoequatorial position to minimize 1,3‐diaxial interactions;^[^ ^61^, ^62^, ^63^ ^]^ the R^1^ thus becomes *cis* to the substituent on the other carbon. The Ni^I^ complex **9** (possibly in equilibrium with the metal‐stabilized **III**) abstracts an F atom from **II**, yielding the product **3** and the Ni^II^‐F complex **8**, from which the Ni^II^‐H (**1a**) is regenerated by PhSiH_3_.

**Scheme 5:**
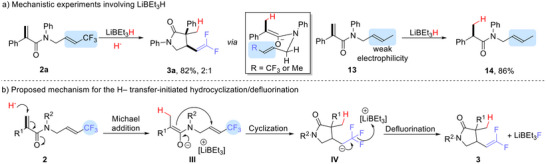
Mechanistic investigation of hydride transfer‐initiated hydrocyclization/defluorination.

The Li[BEt_3_H]‐enabled hydrocyclization/defluorination of a CF_3_‐substituted acrylamide is shown in **Scheme** **5**. Apparently, H^–^ transfer to acrylamides can also trigger our cyclization. Substrate **13**, with a methyl instead of a CF_3_ substituent, gave no cyclization with LiBEt_3_H; only the hydrogenated product **14** was obtained in 86% yield (Scheme 5a), presumably attributed to the fact that the allyl of **13** is less electrophilic than the CF_3_‐substituted vinyl of **2a**.

The mechanism we propose for the Li[BEt_3_H hydrocyclization/defluorination is shown in Scheme 5b. A Hydride‐initiated 1,4‐Michael addition of acrylamide forms intermediate **III**, which then converts into a carbanion **IV** via an intermolecular cyclization. Subsequently, in the presence of [LiBEt_3_]^+^, the defluorination of **IV** generates product **3**.

## 3 Conclusion

We have measured the rate constant for H• transfer to acrylamide (*k*_H_ = 2.28 × 10^−4^ m ^−1^ s^−1^ at 300 K in toluene). Using the H•/H^–^ transfer strategy, we have facilitated the hydrocyclization/defluorination of CF_3_‐substituted acrylamides and have prepared a series of 3‐methy substituted Seletracetams containing *gem*‐difluoroalkenes. Transfer of either H• from Ni^II^‐H or H^–^ from LiBEt_3_H to the terminal C═C of substrate **2** can initiate the hydrocyclization/defluorination. A radical process appears to be responsible for the Ni^II^‐H/PhSiH_3_ system, while the 1,4‐Michael addition of H^–^ appears to be responsible for the Li[BEt_3_H] system. Moreover, the strategy has been used for the late‐stage functionalization of pharmaceuticals, including that of an analog of Flurbiprofen. This reaction should be useful for the synthesis of fluorinated compounds in pharmaceutical manufacturing.

## 4 Experimental Section

### General Procedure for the Synthesis Products with the Ni^II^‐H/PhSiH_3_

A solution of substrate **2** (0.1 mmol), PhSiH_3_ (0.1 mmol, 10.8 mg), and 10 mol.% Ni^II^‐H (4.4 mg) in toluene (0.5 mL) was stirred in a 5 mL vial under argon atmosphere at room temperature for 12 hours. The reaction was monitored by TLC until the full conversion of substrate **2**. Then the solvent was removed under reduced pressure. The residue was purified by column chromatography (7–10% ethyl acetate in hexane) to give the corresponding ***cis*‐3** or ***trans*‐3**. The yield and diastereoselectivity of product **3** were determined by ^19^F NMR of the crude reaction mixture using fluorobenzene as an internal standard, as shown in Table 2.

### *cis*‐4‐(2,2‐Difluorovinyl)‐3‐methyl‐1,3‐diphenylpyrrolidin‐2‐one (*cis*‐3a)

(white solid) *Yield of* ***3a***: 93% (*dr* = 11:1), determined by ^19^F NMR of the crude reaction mixture using fluorobenzene as an internal standard. ^1^H NMR (500 MHz, CDCl_3_, 25 °C, TMS) δ 7.76–7.69 (m, 2H), 7.44–7.38 (m, 2H), 7.35–7.28 (m, 2H), 7.27–7.23 (m, 1H), 7.22–7.17 (m, 1H), 7.14–7.08 (m, 2H), 3.82 (dd, *J* = 9.7, 7.7 Hz, 1H), 3.61 (ddd, *J* = 24.4, 10.4, 2.2 Hz, 1H), 3.47 (t, *J* = 9.9 Hz, 1H), 3.27–3.17 (m, 1H), 1.68 (s, 3H); ^13^C NMR (126 MHz, CDCl_3_, 25 °C, TMS) δ 176.4, 157.1 (dd, *J_C‐F_* = 290.3, 288.6 Hz), 138.9, 138.4, 129.1, 128.7, 127.4, 126.9, 125.0, 120.0, 75.74 (dd, *J_C‐F_* = 25.1, 17.5 Hz), 53.54 (d, *J_C‐F_* = 2.0 Hz), 50.25 (t, *J_C‐F_* = 3.0 Hz), 39.98 (d, *J_C‐F_* = 5.1 Hz), 22.9; ^19^F NMR (471 MHz, CDCl_3_) δ ‐82.41 (d, *J* = 39.2 Hz), ‐87.93 (dd, *J* = 39.2, 24.4 Hz); HRMS (EI) m/z calcd for C_19_H_18_F_2_NO [M+H]^+^ 314.1351, found 314.1356.

### General Procedure for the Synthesis Products with the Li[BEt_3_H]

To a solution of substrate **2** (0.1 mmol) in toluene (1 mL) in a 5 mL of vial was added Li[BEt_3_H] (1.0 equiv, 1 m in THF) under argon atmosphere at room temperature for 0.5 hours. The reaction was monitored by TLC until the full conversion of substrate **2**. Then the reaction was quenched by H_2_O (5 mL, *Caution: Gas release*) and extracted with EtOAc (5 mL × 3). The combined organic extracts were dried with Na_2_SO_4_, filtered, and concentrated under reduced pressure. The residue was purified by column chromatography (7–10% ethyl acetate in hexane) to give the corresponding ***cis*‐3** or ***trans*‐3**. The yield and diastereoselectivity of products **3** were determined by ^19^F NMR of the crude reaction mixture using fluorobenzene as an internal standard, as shown in Table 2

### *cis*‐3‐(4‐Chlorophenyl)‐4‐(2,2‐difluorovinyl)‐3‐methyl‐1‐phenylpyrrolidin‐2‐one (*cis*‐3f): Colorless Oil         Yield o ***3f***: 80% (*dr* = 2.4:1), determined by ^19^F NMR of the crude reaction mixture using fluorobenzene as an internal standard. ^1^H NMR (500 MHz, CDCl_3_, 25 °C, TMS) δ 7.71 (d, *J* = 8.0 Hz, 2H), 7.49 – 7.37 (m, 2H), 7.30 (d, *J* = 8.2 Hz, 2H), 7.22 (t, *J* = 7.4 Hz, 1H), 7.06 (d, *J* = 8.2 Hz, 2H), 3.85 (dd, *J* = 9.8, 7.7 Hz, 1H), 3.62 (ddd, *J* = 24.2, 10.4, 2.2 Hz, 1H), 3.45 (t, *J* = 9.8 Hz, 1H), 3.29 – 3.17 (m, 1H), 1.67 (s, 3H); ^13^C NMR (126 MHz, CDCl_3_, 25 °C, TMS) δ 175.8, 159.4 – 154.8 (m), 138.7, 137.1, 133.4, 129.1, 128.9, 128.4, 125.2, 120.0, 75.5 (dd, *J_C‐F_* = 25.1, 17.6 Hz), 53.2 (m), 50.2 (t, *J_C‐F_* = 2.9 Hz), 39.8 (d, *J_C‐F_* = 5.1 Hz), 23.0; ^19^F NMR (471 MHz, CDCl_3_) δ ‐81.93 (d, *J* = 37.9 Hz), ‐87.34 (dd, *J* = 38.0, 24.2 Hz); HRMS (EI) m/z calcd for C_19_H_17_ClF_2_NO [M+H]^+^ 348.0961, found 348.0958

*Yield of* ***3f***: 80% (*dr* = 2.4:1), determined by ^19^F NMR of the crude reaction mixture using fluorobenzene as an internal standard. ^1^H NMR (500 MHz, CDCl_3_, 25 °C, TMS) δ 7.71 (d, *J* = 8.0 Hz, 2H), 7.49 – 7.37 (m, 2H), 7.30 (d, *J* = 8.2 Hz, 2H), 7.22 (t, *J* = 7.4 Hz, 1H), 7.06 (d, *J* = 8.2 Hz, 2H), 3.85 (dd, *J* = 9.8, 7.7 Hz, 1H), 3.62 (ddd, *J* = 24.2, 10.4, 2.2 Hz, 1H), 3.45 (t, *J* = 9.8 Hz, 1H), 3.29 – 3.17 (m, 1H), 1.67 (s, 3H); ^13^C NMR (126 MHz, CDCl_3_, 25 °C, TMS) δ 175.8, 159.4 – 154.8 (m), 138.7, 137.1, 133.4, 129.1, 128.9, 128.4, 125.2, 120.0, 75.5 (dd, *J_C‐F_* = 25.1, 17.6 Hz), 53.2 (m), 50.2 (t, *J_C‐F_* = 2.9 Hz), 39.8 (d, *J_C‐F_* = 5.1 Hz), 23.0; ^19^F NMR (471 MHz, CDCl_3_) δ ‐81.93 (d, *J* = 37.9 Hz), ‐87.34 (dd, *J* = 38.0, 24.2 Hz); HRMS (EI) m/z calcd for C_19_H_17_ClF_2_NO [M+H]^+^ 348.0961, found 348.0958.

Further details of substrates **2** and products **3** can be obtained from the Supporting Information.

## Conflict of Interest

The authors declare no conflict of interest.

## Supporting information



Supporting Information

## Data Availability

The data that support the findings of this study are available in the supplementary material of this article.
