# Supplementary material for: Hydrocyclization/Defluorination of CF3‐Substituted Acrylamides: Insights from Kinetics of Hydrogen Atom Transfer
Source: Adv Sci (Weinh). 2025 Apr 25;12(26):2501799. doi: 10.1002/advs.202501799 (PMC12245017; doi:10.1002/advs.202501799)
Supplement: Supplementary file 1 — Supporting Information [file ADVS-12-2501799-s001.pdf]

ADVANCED SCIENCE
Open Access

Supporting Information

for *Adv. Sci.*, DOI 10.1002/advs.202501799

Hydrocyclization/Defluorination of CF_3-Substituted Acrylamides: Insights from Kinetics of Hydrogen Atom Transfer

*Yanjun Wan, Ronghui Shao and Jack R. Norton**

Supplementary Materials for

Hydrocyclization/Defluorination of CF_3-Substituted Acrylamides: Insights from Kinetics of Hydrogen Atom Transfer

Yanjun Wan,[1] Ronghui Shao,[2,3] and Jack R. Norton*,[2]

[1]College of Pharmaceutical Sciences, Zhejiang University of Technology, Hangzhou 310014, China

[2]Department of Chemistry, Columbia University, 3000 Broadway, New York, NY 10027, United States

[3]College of Chemistry, Nankai University, Tianjin 300071, China

*Correspondence: jrn11@columbia.edu

1. **General Information** 2
2. **Experimental Section** 3
   2.1 Synthesis and Characterization of Substrates 3
   2.2 General Procedure for the Synthesis of Products **3a-3y** 12
   2.3 General Procedure for the Synthetic Applications of ***cis*-3a** 21
   2.4 Kinetics for the Rate Constant of H• Transfer 23
   2.5 Mechanistic Experiments 26
   2.5.1 In-situ Transformation of Nickel Species 26
   2.5.2 TEMPO Trapping Experiment 28
   2.5.3 Hydrogenation of Substrate **13** 29
3. **Reference** 29
4. **NMR Spectra** 30

1. General Information

Materials: All the solvents and reagents were obtained from commercial sources and directly used without purification. Analytical thin-layer chromatography (TLC) was conducted on precoated silica gel plates (silica gel, Polygram SILG/UV 254 plates) with aluminum backing, using UV-light (254 and 365 nm). Flash column chromatography was performed on a silica gel (Merck, 230-400 mesh) column.

Instrumentation: NMR spectra were recorded on Bruker 500 Ascend, DRX 500, DRX 400, or DRX 300 spectrometer and are reported in ppm using $CDCl_3$ and toluene-d_8 as the NMR solvent with TMS as the internal standard. All coupling constants (*J*) are reported in hertz (Hz). Abbreviations are: s, singlet; d, doublet; t, triplet; q, quartet; bs, broad singlet. High-resolution mass spectra (HRMS) were measured on a 7T Bruker Daltonics FT-MS instrument by electrospray ionization (ESI) probe or atmospheric pressure solids analysis probe (ASAP).

2. Experimental Section

2.1 Synthesis and Characterization of Substrates

Substrate Scope

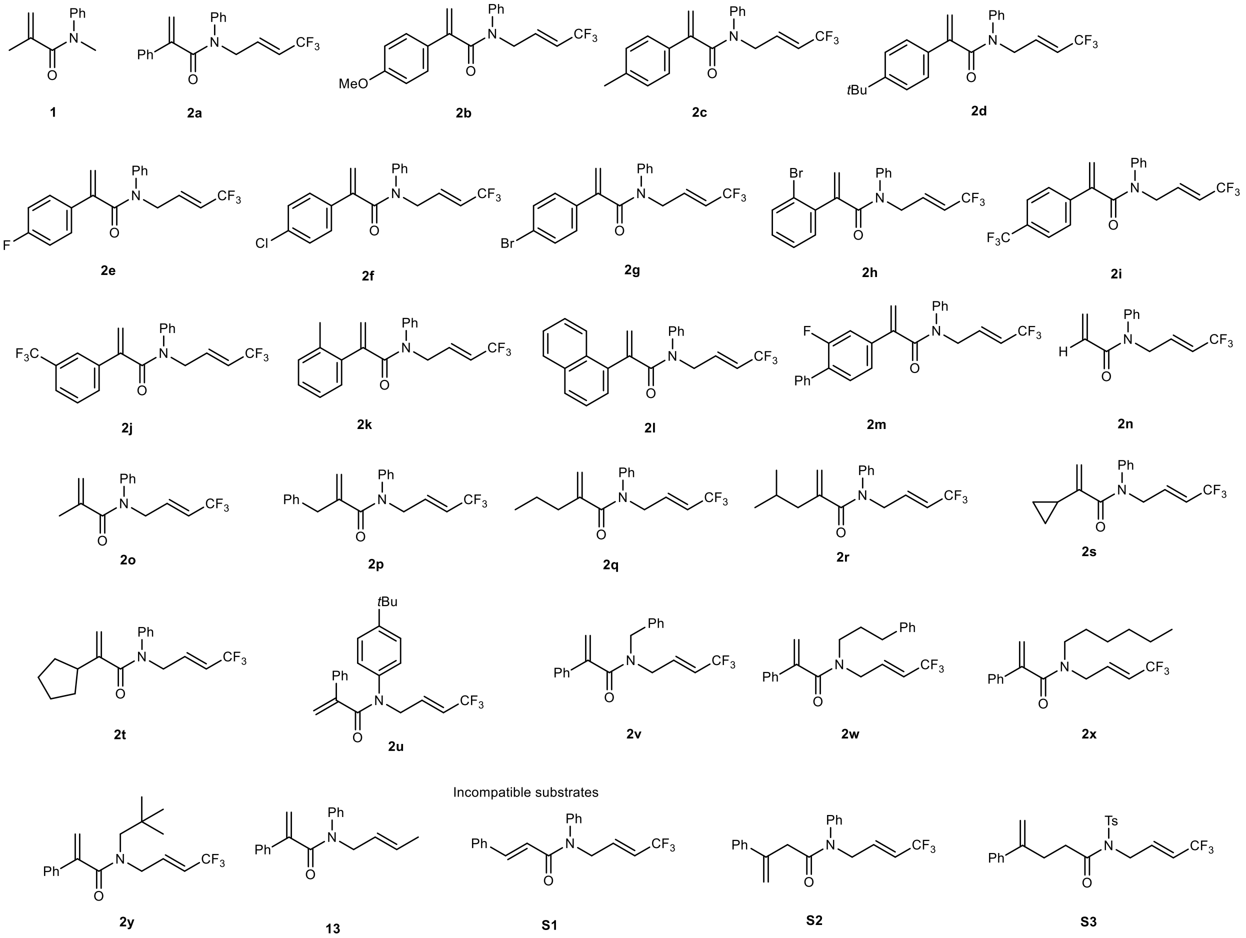

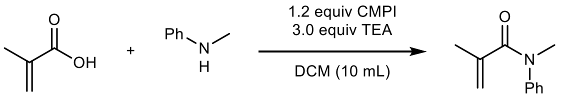

1

To a mixture of methacrylic acid (2.4 mmol, 206 mg), *N*-methylaniline (2 mmol, 214 mg), and 2-chloro-1-methylpyridinium iodide (CMPI, 2.4 mmol, 612 mg) in anhydrous 10 mL of DCM was added triethylamine (TEA, 6 mmol, 0.84 mL). The reaction mixture was stirred at 50 °C for 15 hours. The resulting mixture was cooled to room temperature, quenched with water (40 mL), and extracted with EtOAc (20 mL × 3). The combined organic phases were dried over anhydrous Na_2SO_4, filtered, and concentrated under reduced pressure and the residue was purified by column chromatography (Hex/EA = 10:1) to give substrates **1** (280 mg, 80%) as a white solid.

N-methyl-*N*-phenylmethacrylamide (1)

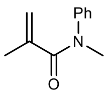

^{1}H NMR (500 MHz, $CDCl_3$, 25 ºC, TMS) δ 7.39 – 7.31 (m, 2H), 7.28 – 7.22 (m, 1H), 7.18 – 7.10 (m, 2H), 5.07 – 5.01 (m, 1H), 5.01 – 4.96 (m, 1H), 3.35 (s, 3H), 1.76 (s, 3H).

^{13}C NMR (126 MHz, $CDCl_3$, 25 ºC, TMS) δ 172.0, 144.7, 140.7, 129.2, 126.9, 126.5, 119.3, 37.6, 20.3.

HRMS (ESI) m/z calcd for $C_{11}H_{14}NO$ $[M+H]^+$ 176.1070, found 176.1073.

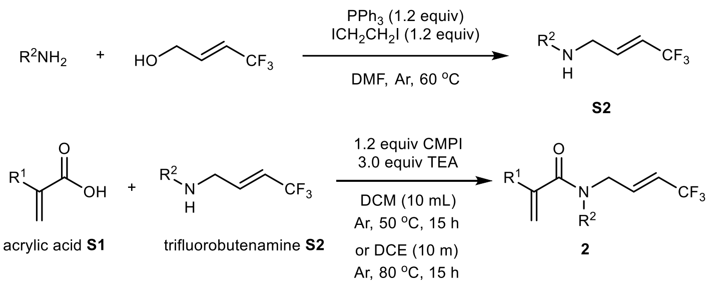

To a mixture of 4,4,4-trifluorobut-2-en-1-ol (5.0 mmol, 630 mg) and PPh_3 (6.0 mmol, 1.57 g) in DMF (20 mL) was added ICH_2CH_2I (6.0 mmol, 1.68 g) and amine (20 mmol) under argon at room temperature. Then the reaction mixture was stirred at 60 °C overnight. The resulting mixture was cooled to room temperature, quenched with water (40 mL), and extracted with EtOAc. The combined organic phases were dried over anhydrous Na_2SO_4, filtered, and concentrated under reduced pressure. The residue was purified by column chromatography on silica gel to give the trifluorobutenamine **S2**.[1]

To a mixture of acrylic acid **S1** (2.4 mmol), trifluorobutenamine **S2** (2 mmol), and 2-chloro-1-methylpyridinium iodide (CMPI, 2.4 mmol, 612 mg) in anhydrous 10 mL of DCM (or DCE) was added triethylamine (TEA, 6 mmol, 0.84 mL). The reaction mixture was stirred at 50 °C (or 80 °C) for 15 hours. The resulting mixture was cooled to room temperature, quenched with water (40 mL), and extracted with EtOAc (20 mL × 3). The combined organic phases were dried over anhydrous Na_2SO_4, filtered, and concentrated under reduced pressure. The residue was purified by column chromatography (Hex/EA = 10:1 or Hex/EA/TEA = 10:1:0.5) to give substrates **2**.

(*E*)-*N*,2-diphenyl-*N*-(4,4,4-trifluorobut-2-en-1-yl)acrylamide (2a)

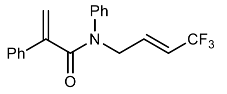

Prepared the general procedure in DCM at 50 °C and purified by flash column chromatograph using Hex/EA/TEA = 10:1:0.5 as eluent to give **2a** (478 mg, 72%) as a colorless oil.

^{1}H NMR (500 MHz, $CDCl_3$, 25 °C, TMS) δ 7.26 – 7.19 (m, 3H), 7.18 – 7.03 (m, 5H), 6.94 – 6.74 (m, 2H), 6.53 – 6.37 (m, 1H), 5.83 – 5.63 (m, 1H), 5.47 (s, 1H), 5.41 (s, 1H), 4.58 – 4.41 (m, 2H).

^{13}C NMR (126 MHz, $CDCl_3$, 25 °C, TMS) δ 170.4, 145.4, 141.7, 136.7, 134.8 (q, $J_{C\text{-}F}$ = 6.4 Hz), 129.1, 128.4, 128.1, 127.8, 127.5, 126.1, 122.6 (q, $J_{C\text{-}F}$ = 269.5 Hz), 121.2 (q, $J_{C\text{-}F}$ = 34.1 Hz), 118.6, 49.8.

^{19}F NMR (471 MHz, $CDCl_3$) δ -63.41 (d, J = 6.3 Hz).

HRMS (ESI) m/z calcd for $C_{19}H_{17}F_3NO$ $[M+H]^+$ 332.1257, found 332.1270.

(*E*)-2-(4-methoxyphenyl)-*N*-phenyl-*N*-(4,4,4-trifluorobut-2-en-1-yl)acrylamide (2b)

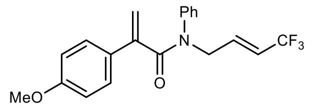

Prepared the general procedure in DCM at 50 °C and purified by flash column chromatograph using Hex/EA/TEA = 10:1:0.5 as eluent to give **2b** (410 mg, 57%) as a colorless oil.

^{1}H NMR (500 MHz, $CDCl_3$, 25 °C, TMS) δ 7.22 – 7.13 (m, 3H), 7.13 – 7.01 (m, 2H), 6.93 – 6.81 (m, 2H), 6.76 (d, J = 8.2 Hz, 2H), 6.57 – 6.37 (m, 1H), 5.82 – 5.65 (m, 1H), 5.38 (s, 1H), 5.25 (s, 1H), 4.58 – 4.40 (m, 2H), 3.78 (s, 3H).

^{13}C NMR (126 MHz, $CDCl_3$, 25 °C, TMS) δ 170.7, 159.6, 144.6, 141.9, 134.8 (q, $J_{C\text{-}F}$ = 6.4 Hz), 129.3, 129.0, 127.7, 127.5, 127.3, 122.6 (q, $J_{C\text{-}F}$ = 269.8 Hz), 121.2 (q, $J_{C\text{-}F}$ = 34.1 Hz), 116.5, 113.8, 55.3, 49.7.

^{19}F NMR (471 MHz, $CDCl_3$) δ -63.41 (d, J = 6.2 Hz).

HRMS (ESI) m/z calcd for $C_{20}H_{19}F_3NO_2$ $[M+H]^+$ 362.1362, found 362.1365.

(*E*)-*N*-phenyl-2-(*p*-tolyl)-*N*-(4,4,4-trifluorobut-2-en-1-yl)acrylamide (2c)

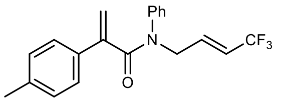

Prepared the general procedure in DCM at 50 °C and purified by flash column chromatograph using Hex/EA/TEA = 10:1:0.5 as eluent to give **2c** (520 mg, 75%) as a colorless oil.

^{1}H NMR (500 MHz, $CDCl_3$, 25 ºC, TMS) δ 7.21 – 7.11 (m, 3H), 7.11 – 6.97 (m, 4H), 6.97 – 6.74 (m, 2H), 6.58 – 6.34 (m, 1H), 5.82 – 5.60 (m, 1H), 5.43 (s, 1H), 5.30 (s, 1H), 4.60 – 4.37 (m, 2H), 2.30 (s, 3H).

^{13}C NMR (126 MHz, $CDCl_3$, 25 ºC, TMS) δ 170.6, 145.1, 141.9, 138.0, 134.9 (q, $J_{C\text{-}F}$ = 6.4 Hz), 133.8, 129.1, 129.0, 127.7, 127.5, 125.9, 122.6 (q, $J_{C\text{-}F}$ = 269.8 Hz), 121.2 (q, $J_{C\text{-}F}$ = 34.1 Hz), 117.3, 49.7, 21.1.

^{19}F NMR (471 MHz, $CDCl_3$) δ -63.42.

HRMS (ESI) m/z calcd for $C_{20}H_{19}F_3NO$ $[M+H]^+$ 346.1413, found 346.1418.

(*E*)-2-(4-(*tert*-butyl)phenyl)-*N*-phenyl-*N*-(4,4,4-trifluorobut-2-en-1-yl)acrylamide (2d)

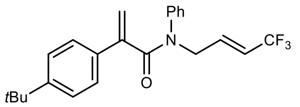

Prepared the general procedure in DCM at 50 °C and purified by flash column chromatograph using Hex/EA/TEA = 10:1:0.5 as eluent to give **2d** (482 mg, 62%) as a colorless oil.

^{1}H NMR (500 MHz, $CDCl_3$, 25 ºC, TMS) δ 7.29 – 7.21 (m, 2H), 7.19 – 6.99 (m, 5H), 6.98 – 6.78 (m, 2H), 6.62 – 6.36 (m, 1H), 5.84 – 5.64 (m, 1H), 5.52 – 5.39 (m, 1H), 5.34 (s, 1H), 4.57 – 4.44 (m, 2H), 1.29 (s, 9H).

^{13}C NMR (126 MHz, $CDCl_3$, 25 ºC, TMS) δ 170.6, 151.2, 145.0, 141.9, 134.9 (q, $J_{C\text{-}F}$ = 6.4 Hz), 133.9, 129.0, 127.7, 127.4, 125.9, 125.3, 122.6 (q, $J_{C\text{-}F}$ = 269.8 Hz), 121.2 (q, $J_{C\text{-}F}$ = 34.1 Hz), 117.9, 49.8, 34.5, 31.2.

^{19}F NMR (471 MHz, $CDCl_3$) δ -63.39 (d, J = 7.0 Hz).

HRMS (ESI) m/z calcd for $C_{23}H_{25}F_3NO$ $[M+H]^+$ 388.1883, found 388.1888.

(*E*)-2-(4-fluorophenyl)-*N*-phenyl-*N*-(4,4,4-trifluorobut-2-en-1-yl)acrylamide (2e)

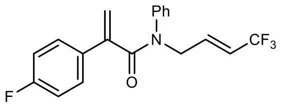

Prepared the general procedure in DCM at 50 °C and purified by flash column chromatograph using Hex/EA/TEA = 10:1:0.5 as eluent to give **2e** (595 mg, 85%) as a colorless oil.

^{1}H NMR (500 MHz, $CDCl_3$, 25 ºC, TMS) δ 7.23 – 7.13 (m, 3H), 7.13 – 6.99 (m, 2H), 6.98 – 6.75 (m, 4H), 6.56 – 6.34 (m, 1H), 5.84 – 5.66 (m, 1H), 5.41 (d, J = 9.6 Hz, 2H), 4.57 – 4.41 (m, 2H).

^{13}C NMR (126 MHz, $CDCl_3$, 25 ºC, TMS) δ 170.2, 162.6 (d, $J_{C\text{-}F}$ = 248.0 Hz), 144.4, 141.6, 134.6 (q, $J_{C\text{-}F}$ = 6.5 Hz), 133.0, 129.1, 127.9, 127.9, 127.8, 127.6, 122.5 (q, $J_{C\text{-}F}$ = 269.3 Hz), 121.4 (q, $J_{C\text{-}F}$ = 34.2 Hz), 118.7, 115.3 (d, $J_{C\text{-}F}$ = 21.7 Hz), 49.8.

^{19}F NMR (471 MHz, $CDCl_3$) δ -63.47 (d, J = 6.4 Hz), -112.70.

HRMS (ESI) m/z calcd for $C_{19}H_{16}F_4NO$ $[M+H]^+$ 350.1163, found 350.1168.

(*E*)-2-(4-chlorophenyl)-*N*-phenyl-*N*-(4,4,4-trifluorobut-2-en-1-yl)acrylamide (2f)

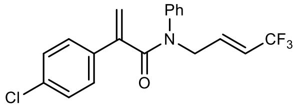

Prepared the general procedure in DCM at 50 °C and purified by flash column chromatograph using Hex/EA/TEA = 10:1:0.5 as eluent to give **2f** (505 mg, 69%) as a white solid.

^{1}H NMR (500 MHz, $CDCl_3$, 25 ºC, TMS) δ 7.25 – 7.13 (m, 5H), 7.12 – 6.97 (m, 2H), 6.93 – 6.76 (m, 2H), 6.52 – 6.37 (m, 1H), 5.74 (dq, J = 12.9, 6.1 Hz, 1H), 5.46 (s, 1H), 5.42 (s, 1H), 4.55 – 4.43 (m, 2H).

^{13}C NMR (126 MHz, $CDCl_3$, 25 ºC, TMS) δ 170.0, 144.3, 141.5, 135.2, 134.6 (q, $J_{C\text{-}F}$ = 6.4 Hz), 134.1, 129.2, 128.6, 127.8, 127.7, 127.4, 122.5 (q, $J_{C\text{-}F}$ = 269.8 Hz), 121.5 (q, $J_{C\text{-}F}$ = 34.1 Hz), 119.1, 49.8.

^{19}F NMR (471 MHz, $CDCl_3$) δ -63.46 (d, J = 6.3 Hz).

HRMS (ESI) m/z calcd for $C_{19}H_{16}ClF_3NO$ $[M+H]^+$ 366.0867, found 366.0869.

(*E*)-2-(4-bromophenyl)-*N*-phenyl-*N*-(4,4,4-trifluorobut-2-en-1-yl)acrylamide (2g)

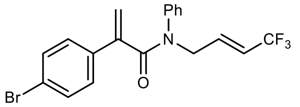

Prepared the general procedure in DCM at 50 °C and purified by flash column chromatograph using Hex/EA/TEA = 10:1:0.5 as eluent to give **2g** (590 mg, 72%) as a white solid.

^{1}H NMR (500 MHz, $CDCl_3$, 25 °C, TMS) δ 7.34 (d, J = 8.1 Hz, 2H), 7.22 – 7.12 (m, 3H), 7.00 (d, J = 8.2 Hz, 2H), 6.93 – 6.76 (m, 2H), 6.54 – 6.34 (m, 1H), 5.86 – 5.63 (m, 1H), 5.46 (s, 1H), 5.43 (s, 1H), 4.50 (d, J = 6.2 Hz, 2H).

^{13}C NMR (126 MHz, $CDCl_3$, 25 °C, TMS) δ 169.9, 144.3, 141.5, 135.7, 134.5 (q, J_{C-F} = 6.4 Hz), 131.5, 129.2, 127.8, 127.7, 127.7, 122.5 (q, J_{C-F} = 269.8 Hz), 122.2, 121.4 (q, J_{C-F} = 34.1 Hz), 119.2, 49.8.

^{19}F NMR (471 MHz, $CDCl_3$) δ -63.43 (d, J = 6.3 Hz).

HRMS (ESI) m/z calcd for $C_{19}H_{16}BrF_3NO$ $[M+H]^+$ 410.0362, found 410.0366.

(*E*)-2-(2-bromophenyl)-*N*-phenyl-*N*-(4,4,4-trifluorobut-2-en-1-yl)acrylamide (2h)

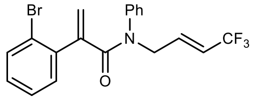

Prepared the general procedure in DCM at 50 °C and purified by flash column chromatograph using Hex/EA/TEA = 10:1:0.5 as eluent to give **2h** (590 mg, 64%) as a white solid.

^{1}H NMR (500 MHz, $CDCl_3$, 25 °C, TMS) δ 7.34 (dd, J = 7.9, 1.3 Hz, 1H), 7.06 – 7.01 (m, 3H), 6.91 (td, J = 7.6, 1.8 Hz, 1H), 6.86 (dd, J = 7.5, 1.3 Hz, 1H), 6.81 – 6.75 (m, 2H), 6.55 – 6.45 (m, 2H), 6.17 (d, J = 1.3 Hz, 1H), 5.76 – 5.66 (m, 1H), 5.53 (d, J = 1.3 Hz, 1H), 4.48 – 4.40 (m, 2H).

^{13}C NMR (126 MHz, $CDCl_3$, 25 °C, TMS) δ 168.6, 146.2, 141.4, 138.7, 134.9 (q, J_{C-F} = 6.4 Hz), 132.3, 131.1, 129.1, 128.7, 127.6, 127.5, 127.2, 121.6, 122.6 (q, J_{C-F} = 269.8 Hz), 121.3 (q, J_{C-F} = 34.1 Hz), 50.9.

^{19}F NMR (471 MHz, $CDCl_3$) δ -63.4.

HRMS (ESI) m/z calcd for $C_{19}H_{16}BrF_3NO$ $[M+H]^+$ 410.0362, found 410.0367.

(*E*)-*N*-phenyl-*N*-(4,4,4-trifluorobut-2-en-1-yl)-2-(4-(trifluoromethyl)phenyl)acrylamide (2i)

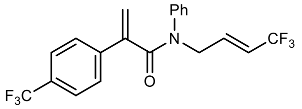

Prepared the general procedure in DCM at 50 °C and purified by flash column chromatograph using Hex/EA/TEA = 10:1:0.5 as eluent to give **2i** (590 mg, 69%) as a colorless oil.

^{1}H NMR (500 MHz, $CDCl_3$, 25 °C, TMS) δ 7.46 (d, J = 8.0 Hz, 2H), 7.23 (d, J = 8.0 Hz, 2H), 7.20 – 7.13 (m, 3H), 6.92 – 6.75 (m, 2H), 6.53 – 6.36 (m, 1H), 5.81 – 5.69 (m, 1H), 5.55 (d, J = 3.5 Hz, 2H), 4.56 – 4.45 (m, 2H).

^{13}C NMR (126 MHz, $CDCl_3$, 25 °C, TMS) δ 169.6, 144.4, 141.4, 140.3, 134.4 (q, J_{C-F} = 6.4 Hz), 129.2, 127.8, 127.8, 126.5, 125.3 (q, J_{C-F} = 3.6 Hz), 124.0 (q, J_{C-F} = 272.0 Hz), 122.5 (q, J_{C-F} = 269.8 Hz), 121.6 (q, J_{C-F} = 34.1 Hz), 120.8, 49.9.

^{19}F NMR (471 MHz, $CDCl_3$) δ -61.84, -63.51 (d, J = 7.1 Hz).

HRMS (ESI) m/z calcd for $C_{20}H_{16}F_6NO$ $[M+H]^+$ 400.1131, found 400.1136.

(*E*)-*N*-phenyl-*N*-(4,4,4-trifluorobut-2-en-1-yl)-2-(3-(trifluoromethyl)phenyl)acrylamide (2j)

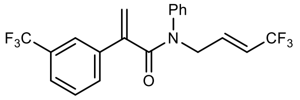

Prepared the general procedure in DCM at 50 °C and purified by flash column chromatograph using Hex/EA/TEA = 10:1:0.5 as eluent to give **2j** (472 mg, 59%) as a colorless oil.

^{1}H NMR (500 MHz, $CDCl_3$, 25 ºC, TMS) δ 7.45 (d, *J* = 7.3 Hz, 1H), 7.37 – 7.21 (m, 3H), 7.19 – 7.10 (m, 3H), 6.81 (d, *J* = 6.8 Hz, 2H), 6.54 – 6.39 (m, 1H), 5.81 – 5.68 (m, 1H), 5.61 (s, 1H), 5.53 (s, 1H), 4.57 – 4.41 (m, 2H).

^{13}C NMR (126 MHz, $CDCl_3$, 25 ºC, TMS) δ 169.6, 144.6, 141.3, 137.8, 134.4 (q, $J_{C\text{-}F}$ = 6.4 Hz), 129.4, 129.2, 128.8, 127.8, 127.8, 124.6 (m), 123.8 (q, $J_{C\text{-}F}$ = 272.5 Hz), 123.2 (q, $J_{C\text{-}F}$ = 3.6 Hz), 122.5 (d, $J_{C\text{-}F}$ = 271.1 Hz), 121.6 (q, $J_{C\text{-}F}$ = 34.1 Hz), 120.9, 49.9.

^{19}F NMR (471 MHz, $CDCl_3$) δ -62.02, -63.55 (d, *J* = 7.8 Hz).

HRMS (ESI) m/z calcd for $C_{20}H_{16}F_6NO$ $[M+H]^+$ 400.1131, found 400.1135.

(*E*)-*N*-phenyl-2-(*o*-tolyl)-*N*-(4,4,4-trifluorobut-2-en-1-yl)acrylamide (2k)

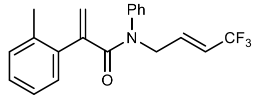

Prepared the general procedure in DCM at 50 °C and purified by flash column chromatograph using Hex/EA/TEA = 10:1:0.5 as eluent to give **2k** (546 mg, 79%) as a colorless oil.

^{1}H NMR (500 MHz, $CDCl_3$, 25 ºC, TMS) δ 7.13 – 7.04 (m, 3H), 7.02 – 6.96 (m, 1H), 6.95 (d, *J* = 7.4 Hz, 1H), 6.84 – 6.77 (m, 1H), 6.73 – 6.67 (m, 2H), 6.56 – 6.48 (m, 1H), 6.48 – 6.36 (m, 1H), 5.98 (d, *J* = 1.4 Hz, 1H), 5.72 – 5.60 (m, 1H), 5.36 (d, *J* = 1.4 Hz, 1H), 4.43 – 4.34 (m, 2H), 2.08 (s, 3H).

^{13}C NMR (126 MHz, $CDCl_3$, 25 ºC, TMS) δ 170.5, 146.5, 141.3, 137.2, 135.0, 134.6 (q, $J_{C\text{-}F}$ = 6.3 Hz), 129.9, 129.0, 128.9, 127.9, 127.5, 127.4, 125.6, 124.5, 122.5 (q, $J_{C\text{-}F}$ = 269.8 Hz), 121.5 (q, $J_{C\text{-}F}$ = 34.0 Hz), 50.5, 19.8.

^{19}F NMR (471 MHz, $CDCl_3$) δ -63.48 (d, *J* = 6.3 Hz).

HRMS (ESI) m/z calcd for $C_{20}H_{19}F_3NO$ $[M+H]^+$ 346.1413, found 346.1416.

(*E*)-2-(naphthalen-1-yl)-*N*-phenyl-*N*-(4,4,4-trifluorobut-2-en-1-yl)acrylamide (2l)

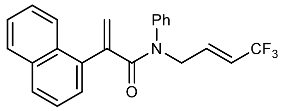

Prepared the general procedure in DCM at 50 °C and purified by flash column chromatograph using Hex/EA/TEA = 10:1:0.5 as eluent to give **2l** (550 mg, 72%) as a colorless oil.

^{1}H NMR (500 MHz, $CDCl_3$, 25 ºC, TMS) δ 7.86 – 7.81 (m, 1H), 7.77 – 7.73 (m, 1H), 7.58 (d, *J* = 8.2 Hz, 1H), 7.46 – 7.40 (m, 2H), 7.05 – 6.96 (m, 2H), 6.92 (t, *J* = 7.7 Hz, 2H), 6.68 – 6.55 (m, 1H), 6.48 – 6.31 (m, 3H), 6.19 (d, *J* = 1.6 Hz, 1H), 5.66 – 5.55 (m, 2H), 4.35 – 4.29 (m, 2H).

^{13}C NMR (126 MHz, $CDCl_3$, 25 ºC, TMS) δ 170.8, 145.9, 141.5, 135.4, 134.8 (q, $J_{C\text{-}F}$ = 6.4 Hz), 133.1, 130.6, 128.9, 128.2, 127.9, 127.6, 127.4, 126.8, 126.2, 125.8, 125.7, 125.2, 124.9, 122.6 (q, $J_{C\text{-}F}$ = 269.3 Hz), 121.0 (q, $J_{C\text{-}F}$ = 34.0 Hz), 50.6.

^{19}F NMR (471 MHz, $CDCl_3$) δ -63.40 (d, *J* = 6.3 Hz).

HRMS (ESI) m/z calcd for $C_{23}H_{19}F_3NO$ $[M+H]^+$ 382.1413, found 382.1415.

(*E*)-2-(2-fluoro-[1,1'-biphenyl]-4-yl)-*N*-phenyl-*N*-(4,4,4-trifluorobut-2-en-1-yl)acrylamide (2m)

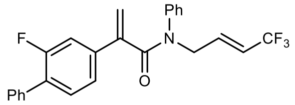

Prepared the general procedure in DCM at 50 °C and purified by flash column chromatograph using Hex/EA/TEA = 10:1:0.5 as eluent to give **2m** (510 mg, 60%) as a colorless oil.

^{1}H NMR (500 MHz, $CDCl_3$, 25 ºC, TMS) δ 7.54 – 7.50 (m, 2H), 7.47 – 7.40 (m, 2H), 7.40 – 7.33 (m, 1H), 7.35 – 7.28 (m, 1H), 7.24 – 7.15 (m, 3H), 7.07 – 6.99 (m, 1H), 7.00 – 6.85 (m, 3H), 6.56 – 6.40 (m, 1H), 5.82 – 5.70 (m, 1H), 5.54 (s, 1H), 5.45 (s, 1H), 4.58 – 4.44 (m, 2H).

^{13}C NMR (126 MHz, $CDCl_3$, 25 ºC, TMS) δ 169.8, 159.6 (d, $J_{C\text{-}F}$ = 248.4 Hz), 144.0 (d, $J_{C\text{-}F}$ = 2.0 Hz), 141.7, 137.7, 134.6 (q, *J* = 6.4 Hz), 135.2, 130.6 (d, $J_{C\text{-}F}$ = 2.9 Hz), 129.2, 128.9, 128.9, 128.5, 127.9, 127.8, 127.7, 122.6 (d, $J_{C\text{-}F}$ =

269.8 Hz), 122.2 (d, $J_{C\text{-}F}$ = 3.4 Hz), 121.5 (q, $J_{C\text{-}F}$ = 34.2 Hz), 119.5, 113.9 (d, $J_{C\text{-}F}$ = 24.3 Hz), 49.9.

^{19}F NMR (471 MHz, $CDCl_3$) δ -63.45 (d, *J* = 6.3 Hz), -116.87.

HRMS (ESI) m/z calcd for $C_{25}H_{20}F_4NO$ $[M+H]^+$ 426.1476, found 426.1476.

(*E*)-*N*-phenyl-*N*-(4,4,4-trifluorobut-2-en-1-yl)acrylamide (2n)

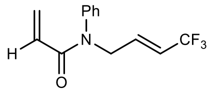

Prepared the general procedure in DCM at 50 °C and purified by flash column chromatograph using Hex/EA = 10:1 as eluent to give **2n** (261 mg, 51%) as a colorless oil.

^{1}H NMR (500 MHz, $CDCl_3$, 25 ºC, TMS) δ 7.46 – 7.35 (m, 3H), 7.17 – 7.12 (m, 2H), 6.50 – 6.38 (m, 2H), 6.04 (dd, *J* = 16.8, 10.3 Hz, 1H), 5.79 – 5.66 (m, 1H), 5.57 (dd, *J* = 10.3, 1.9 Hz, 1H), 4.55 – 4.38 (m, 2H).

^{13}C NMR (126 MHz, $CDCl_3$, 25 ºC, TMS) δ 165.5, 141.4, 134.9 (q, $J_{C\text{-}F}$ = 6.4 Hz), 129.8, 128.5, 128.3, 128.0, 127.9, 122.6 (q, $J_{C\text{-}F}$ = 269.6 Hz), 121.0 (q, $J_{C\text{-}F}$ = 34.1 Hz), 49.9.

^{19}F NMR (471 MHz, $CDCl_3$) δ -64.29 (d, *J* = 6.3 Hz).

HRMS (ESI) m/z calcd for $C_{13}H_{13}F_3NO$ $[M+H]^+$ 256.0944, found 256.0949.

(*E*)-*N*-phenyl-*N*-(4,4,4-trifluorobut-2-en-1-yl)methacrylamide (2o)

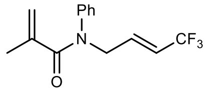

Prepared the general procedure in DCE at 80 °C and purified by flash column chromatograph using Hex/EA = 10:1 as eluent to give **2o** (297 mg, 55%) as a colorless oil.

^{1}H NMR (500 MHz, $CDCl_3$, 25 ºC, TMS) δ 7.38 – 7.32 (m, 2H), 7.31 – 7.26 (m, 1H), 7.11 – 7.06 (m, 2H), 6.51 – 6.40 (m, 1H), 5.78 – 5.66 (m, 1H), 5.11 – 5.06 (m, 1H), 5.06 – 5.01 (m, 1H), 4.50 – 4.42 (m, 2H), 1.78 – 1.73 (m, 3H).

^{13}C NMR (126 MHz, $CDCl_3$, 25 ºC, TMS) δ 171.6, 142.8, 140.2, 135.1 (q, $J_{C\text{-}F}$ = 6.4 Hz), 129.5, 127.5, 127.1, 122.6 (q, $J_{C\text{-}F}$ = 269.3 Hz), 120.9 (q, $J_{C\text{-}F}$ = 34.1 Hz), 120.3, 50.1, 20.2.

^{19}F NMR (471 MHz, $CDCl_3$) δ -63.38 (m).

HRMS (ESI) m/z calcd for $C_{14}H_{15}F_3NO$ $[M+H]^+$ 270.1100, found 270.1106.

(*E*)-2-benzyl-*N*-phenyl-*N*-(4,4,4-trifluorobut-2-en-1-yl)acrylamide (2p)

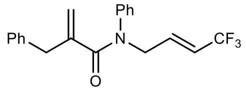

Prepared the general procedure in DCM at 50 °C and purified by flash column chromatograph using Hex/EA = 10:1 as eluent to give **2p** (429 mg, 62%) as a colorless oil.

^{1}H NMR (500 MHz, $CDCl_3$, 25 ºC, TMS) δ 7.33 – 7.26 (m, 2H), 7.27 – 7.25 (m, 1H), 7.25 – 7.22 (m, 3H), 7.11 – 7.07 (m, 2H), 6.70 – 6.63 (m, 2H), 6.35 – 6.22 (m, 1H), 5.57 – 5.46 (m, 1H), 5.06 (s, 1H), 4.99 (s, 1H), 4.42 – 4.35 (m, 2H), 3.47 (s, 2H).

^{13}C NMR (126 MHz, $CDCl_3$, 25 ºC, TMS) δ 170.4, 143.7, 142.6, 137.5, 134.7 (q, $J_{C\text{-}F}$ = 6.4 Hz), 129.4, 129.3, 128.5, 127.5, 127.3, 126.8, 122.6 (q, $J_{C\text{-}F}$ = 269.3 Hz), 120.5 (q, $J_{C\text{-}F}$ = 34.1 Hz), 120.1, 50.3, 40.7.

^{19}F NMR (471 MHz, $CDCl_3$) δ -63.32 (dd, *J* = 6.1, 2.6 Hz).

HRMS (ESI) m/z calcd for $C_{20}H_{19}F_3NO$ $[M+H]^+$ 346.1413, found 346.1416.

(*E*)-2-methylene-*N*-phenyl-*N*-(4,4,4-trifluorobut-2-en-1-yl)pentanamide (2q)

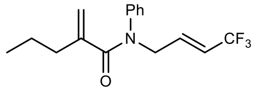

Prepared the general procedure in DCE at 80 °C and purified by flash column chromatograph using Hex/EA = 10:1 as eluent to give **2q** (268 mg, 45%) as a colorless oil.

^{1}H NMR (500 MHz, $CDCl_3$, 25 ºC, TMS) δ 7.38 – 7.32 (m, 2H), 7.30 – 7.25 (m, 1H), 7.12

– 7.06 (m, 2H), 6.49 – 6.39 (m, 1H), 5.78 – 5.64 (m, 1H), 5.07 (d, J = 1.2 Hz, 1H), 5.05 (d, J = 1.2 Hz, 1H), 4.51 – 4.41 (m, 2H), 2.05 (t, J = 7.7 Hz, 2H), 1.45 – 1.36 (m, 2H), 0.86 (t, J = 7.3 Hz, 3H).

^{13}C NMR (126 MHz, $CDCl_3$, 25 ºC, TMS) δ 171.5, 144.5, 142.8, 135.1 (q, $J_{C\text{-}F}$ = 6.4 Hz), 129.4, 127.5, 127.4, 122.6 (q, $J_{C\text{-}F}$ = 269.3 Hz), 121.0 (q, $J_{C\text{-}F}$ = 34.0 Hz), 119.0, 50.2, 35.7, 20.8, 13.6.

^{19}F NMR (471 MHz, $CDCl_3$) δ -63.40 (d, J = 6.5 Hz).

HRMS (ESI) m/z calcd for $C_{16}H_{19}F_3NO$ $[M+H]^+$ 298.1413, found 298.1415.

(*E*)-4-methyl-2-methylene-*N*-phenyl-*N*-(4,4,4-trifluorobut-2-en-1-yl)pentanamide (2r)

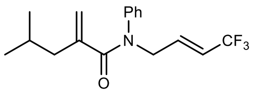

Prepared the general procedure in DCE at 80 °C and purified by flash column chromatograph using Hex/EA = 10:1 as eluent to give **2r** (262 mg, 42%) as a colorless oil.

^{1}H NMR (500 MHz, $CDCl_3$, 25 ºC, TMS) δ 7.38 – 7.32 (m, 2H), 7.31 – 7.26 (m, 1H), 7.12 – 7.07 (m, 2H), 6.48 – 6.39 (m, 1H), 5.79 – 5.64 (m, 1H), 5.14 (d, J = 1.1 Hz, 1H), 5.06 (d, J = 1.1 Hz, 1H), 4.48 – 4.42 (m, 2H), 1.88 (dd, J = 7.0, 1.3 Hz, 2H), 1.79 – 1.66 (m, 1H), 0.83 (s, 3H), 0.82 (s, 3H).

^{13}C NMR (126 MHz, $CDCl_3$, 25 ºC, TMS) δ 171.4, 143.8, 142.6, 135.1 (q, $J_{C\text{-}F}$ = 6.3 Hz), 129.4, 127.5, 127.5, 122.6 (q, $J_{C\text{-}F}$ = 269.3 Hz), 121.0 (q, $J_{C\text{-}F}$ = 34.2 Hz), 120.5, 50.3, 43.2, 26.5, 22.3.

^{19}F NMR (471 MHz, $CDCl_3$) δ -63.41 (dd, J = 6.1, 2.7 Hz).

HRMS (ESI) m/z calcd for $C_{17}H_{21}F_3NO$ $[M+H]^+$ 312.1570, found 312.1577.

(*E*)-2-cyclopropyl-*N*-phenyl-N-(4,4,4-trifluorobut-2-en-1-yl)acrylamide (2s)

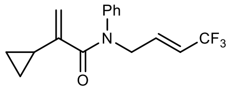

Prepared the general procedure in DCE at 80 °C and purified by flash column chromatograph using Hex/EA = 10:1 as eluent to give **2q** (364 mg, 61%) as a colorless oil.

^{1}H NMR (500 MHz, $CDCl_3$, 25 ºC, TMS) δ 7.39 – 7.31 (m, 2H), 7.30 – 7.25 (m, 1H), 7.15 – 7.10 (m, 2H), 6.46 (dtq, J = 16.1, 6.1, 2.1 Hz, 1H), 5.74 (dqt, J = 15.8, 6.3, 1.6 Hz, 1H), 4.99 (s, 1H), 4.89 (s, 1H), 4.53 – 4.45 (m, 2H), 1.37 – 1.28 (m, 1H), 0.67 – 0.59 (m, 2H), 0.44 – 0.37 (m, 2H).

^{13}C NMR (126 MHz, $CDCl_3$, 25 ºC, TMS) δ 171.2, 146.3, 142.6, 135.1 (q, $J_{C\text{-}F}$ = 6.4 Hz), 129.3, 127.5, 127.4, 122.6 (q, $J_{C\text{-}F}$ = 269.4 Hz), 120.9 (q, $J_{C\text{-}F}$ = 34.0 Hz), 115.4, 50.1, 13.8, 7.4.

^{19}F NMR (471 MHz, $CDCl_3$) δ -63.34 – -63.44 (m).

HRMS (ESI) m/z calcd for $C_{16}H_{17}F_3NO$ $[M+H]^+$ 296.1257, found 296.1252.

(*E*)-2-cyclopentyl-*N*-phenyl-*N*-(4,4,4-trifluorobut-2-en-1-yl)acrylamide (2t)

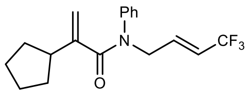

Prepared the general procedure in DCE at 80 °C and purified by flash column chromatograph using Hex/EA = 10:1 as eluent to give **2t** (356 mg, 55%) as a colorless oil.

^{1}H NMR (500 MHz, $CDCl_3$, 25 ºC, TMS) δ 7.38 – 7.31 (m, 2H), 7.30 – 7.24 (m, 1H), 7.12 – 7.07 (m, 2H), 6.50 – 6.39 (m, 1H), 5.78 – 5.64 (m, 1H), 5.09 (d, J = 1.7 Hz, 1H), 5.02 (d, J = 1.1 Hz, 1H), 4.50 – 4.44 (m, 2H), 2.57 – 2.44 (m, 1H), 1.81 – 1.70 (m, 2H), 1.70 – 1.59 (m, 2H), 1.58 – 1.48 (m, 2H), 1.43 – 1.30 (m, 2H).

^{13}C NMR (126 MHz, $CDCl_3$, 25 ºC, TMS) δ 171.8, 148.0, 142.8, 135.1 (q, $J_{C\text{-}F}$ = 6.4 Hz), 129.3, 127.7, 127.5, 122.6 (q, $J_{C\text{-}F}$ = 269.8 Hz), 120.9 (q, $J_{C\text{-}F}$ = 34.1 Hz), 117.0, 50.2, 43.0, 31.5, 24.6.

^{19}F NMR (471 MHz, $CDCl_3$) δ -63.39 (d, J = 6.3 Hz).

HRMS (ESI) m/z calcd for $C_{18}H_{21}F_3NO$ $[M+H]^+$ 324.1570, found 324.1575.

(*E*)-*N*-(4-(*tert*-butyl)phenyl)-2-phenyl-*N*-(4,4,4-trifluorobut-2-en-1-yl)acrylamide (2u)

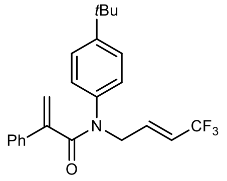

Prepared the general procedure in DCM at 50 °C and purified by flash column chromatograph using Hex/EA/TEA = 10:1:0.5 as eluent to give **2u** (566 mg, 73%) as a colorless oil.

^{1}H NMR (500 MHz, $CDCl_3$, 25 ºC, TMS) δ 7.22 – 7.13 (m, 3H), 7.13 – 7.07 (m, 2H), 7.07 – 6.97 (m, 2H), 6.73 (d, *J* = 8.1 Hz, 2H), 6.54 – 6.38 (m, 1H), 5.77 (dq, *J* = 13.5, 6.2 Hz, 1H), 5.47 (s, 1H), 5.43 (s, 1H), 4.48 (d, *J* = 6.1 Hz, 2H), 1.24 (s, 9H).

^{13}C NMR (126 MHz, $CDCl_3$, 25 ºC, TMS) δ 170.6, 150.6, 145.8, 138.9, 137.1, 135.0 (q, $J_{C\text{-}F}$ = 6.4 Hz), 128.2, 127.8, 127.4, 126.2, 125.8, 122.7 (d, $J_{C\text{-}F}$ = 269.3 Hz), 121.0 (q, $J_{C\text{-}F}$ = 34.0 Hz), 118.9, 49.8, 34.5, 31.2.

^{19}F NMR (471 MHz, $CDCl_3$) δ -63.33 (d, *J* = 6.3 Hz).

HRMS (ESI) m/z calcd for $C_{23}H_{25}F_3NO$ $[M+H]^+$ 388.1883, found 388.1887.

(*E*)-*N*-benzyl-2-phenyl-*N*-(4,4,4-trifluorobut-2-en-1-yl)acrylamide (2v)

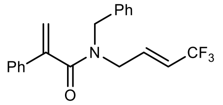

Prepared the general procedure in DCM at 50 °C and purified by flash column chromatograph using Hex/EA = 5:1 as eluent to give **2v** (450 mg, 65%) as a colorless oil.

Major rotamer: ^{1}H NMR (500 MHz, $CDCl_3$, 25 ºC, TMS) δ 7.50 – 7.25 (m, 9H), 7.07 – 7.04 (m, 1H), 6.38 – 6.28 (m, 1H), 5.74 (s, 1H), 5.66 – 5.58 (m, 1H), 5.47 (s, 1H), 4.42 (s, 2H), 4.17 – 4.07 (m, 2H).

^{13}C NMR (126 MHz, $CDCl_3$, 25 ºC, TMS) δ 171.1, 144.7, 136.4, 135.6, 134.6 (q, $J_{C\text{-}F}$ = 5.9 Hz), 129.1, 129.0, 128.9, 128.1, 127.3, 125.8, 122.6 (q, $J_{C\text{-}F}$ = 269.8 Hz), 120.9 (q, $J_{C\text{-}F}$ = 34.3 Hz), 115.0, 52.0, 47.4.

^{19}F NMR (471 MHz, $CDCl_3$) δ -63.32 (d, *J* = 6.8 Hz).

HRMS (ESI) m/z calcd for $C_{20}H_{19}F_3NO$ $[M+H]^+$ 346.1413, found 346.1416.

(*E*)-2-phenyl-*N*-(3-phenylpropyl)-*N*-(4,4,4-trifluorobut-2-en-1-yl)acrylamide (2w)

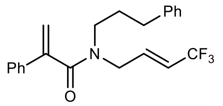

Prepared the general procedure in DCM at 50 °C and purified by flash column chromatograph using Hex/EA = 5:1 as eluent to give **2w** (380 mg, 51%) as a colorless oil.

Major rotamer: ^{1}H NMR (500 MHz, $CDCl_3$, 25 ºC, TMS) δ 7.49 – 7.17 (m, 9H), 7.07 – 6.99 (m, 1H), 6.46 – 6.34 (m, 1H), 5.72 – 5.66 (m, 1H), 5.64 (s, 1H), 5.36 (s, 1H), 4.22 – 4.16 (m, 2H), 3.20 – 3.12 (m, 2H), 2.43 (t, *J* = 7.5 Hz, 2H), 1.77 – 1.69 (m, 2H).

^{13}C NMR (126 MHz, $CDCl_3$, 25 ºC, TMS) δ 170.8, 145.1, 140.6, 135.7, 135.3 (q, $J_{C\text{-}F}$ = 5.9 Hz), 129.0, 128.9, 128.5, 128.2, 126.2, 125k.6, 122.7 (q, $J_{C\text{-}F}$ = 269.3 Hz), 120.5 (q, $J_{C\text{-}F}$ = 34.1 Hz), 114.6, 48.1, 44.7, 32.8, 30.1.

^{19}F NMR (471 MHz, $CDCl_3$) δ -63.23 (dd, *J* = 6.2, 2.8 Hz).

HRMS (ESI) m/z calcd for $C_{22}H_{23}F_3NO$ $[M+H]^+$ 374.1726, found 374.1729.

(*E*)-*N*-hexyl-2-phenyl-*N*-(4,4,4-trifluorobut-2-en-1-yl)acrylamide (2x)

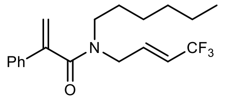

Prepared the general procedure in DCM at 50 °C and purified by flash column chromatograph using Hex/EA = 5:1 as eluent to give **2x** (380 mg, 51%) as a colorless oil.

^{1}H NMR (500 MHz, $CDCl_3$, 25 ºC, TMS) δ 7.50 – 7.38 (m, 2H), 7.40 – 7.28 (m, 3H), 6.48 – 6.35 (m, 1H), 5.78 – 5.71 (m, 2H), 5.37 (s, 1H), 4.22 – 4.15 (m, 2H), 3.14 (t, *J* = 7.8 Hz, 2H), 1.40 – 1.03 (m, 8H), 0.83 (t, *J* = 7.2 Hz, 3H).

^{13}C NMR (126 MHz, $CDCl_3$, 25 ºC, TMS) δ 170.8, 145.2, 135.7, 135.5 (q, $J_{C\text{-}F}$ = 6.2 Hz), 128.9, 128.8, 125.7, 122.7 (q, $J_{C\text{-}F}$ = 269.3 Hz), 120.3 (q, $J_{C\text{-}F}$ = 34.3 Hz), 114.4, 48.8, 44.6, 31.2, 28.5, 26.2, 22.4, 13.9.

^{19}F NMR (471 MHz, $CDCl_3$) δ -63.27 (dd, *J* = 5.8, 2.8 Hz).

HRMS (ESI) m/z calcd for $C_{19}H_{25}F_3NO$ $[M+H]^+$ 340.1883, found 340.1887.

(*E*)-*N*-neopentyl-2-phenyl-*N*-(4,4,4-trifluorobut-2-en-1-yl)acrylamide (2y)

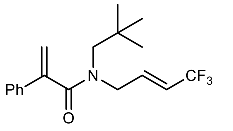

Prepared the general procedure in DCM at 50 °C and purified by flash column chromatograph using Hex/EA = 5:1 as eluent to give **2y** (450 mg, 69%) as a colorless oil.

Major rotamer: ^{1}H NMR (500 MHz, $CDCl_3$, 25 ºC, TMS) δ 7.47 – 7.43 (m, 2H), 7.39 – 7.32 (m, 3H), 6.15 – 6.04 (m, 1H), 5.66 (s, 1H), 5.60 – 5.50 (m, 1H), 5.35 (s, 1H), 4.04 – 3.98 (m, 2H), 3.30 (s, 2H), 1.01 (s, 9H).

^{13}C NMR (126 MHz, $CDCl_3$, 25 ºC, TMS) δ 172.0, 145.6, 135.6, 135.5 (q, $J_{C\text{-}F}$ = 6.1 Hz), 128.9, 128.8, 125.8, 122.5 (d, $J_{C\text{-}F}$ = 269.8 Hz), 120.3 (q, $J_{C\text{-}F}$ = 34.1 Hz), 114.9, 55.4, 50.7, 34.6, 28.7.

^{19}F NMR (471 MHz, $CDCl_3$) δ -63.41 (dd, J = 6.1, 2.7 Hz).

HRMS (ESI) m/z calcd for $C_{18}H_{23}F_3NO$ $[M+H]^+$ 326.1726, found 326.1728.

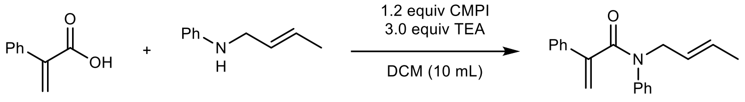

13

To a mixture of 2-phenylacrylic acid (2.4 mmol, 355 mg), (*E*)-*N*-(but-2-en-1-yl)aniline (2 mmol, 294 mg), and 2-chloro-1-methylpyridinium iodide (CMPI, 2.4 mmol, 612 mg) in anhydrous 10 mL of DCM was added triethylamine (TEA, 6 mmol, 0.84 mL). The reaction mixture was stirred at 50 °C for 15 hours. The resulting mixture was cooled to room temperature, quenched with water (40 mL), and extracted with EtOAc (20 mL × 3). The combined organic phases were dried over anhydrous Na_2SO_4, filtered, and concentrated under reduced pressure and the residue was purified by column chromatography (Hex/EA = 10:1) to give substrates **13** (432 mg, 78%) as a colorless oil.

(*E*)-*N*-(but-2-en-1-yl)-N,2-diphenylacrylamide (13)

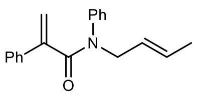

^{1}H NMR (500 MHz, $CDCl_3$, 25 ºC, TMS) δ 7.25 – 7.08 (m, 8H), 6.97 – 6.75 (m, 2H), 5.66 – 5.48 (m, 2H), 5.43 (s, 1H), 5.34 (s, 1H), 4.43 – 4.19 (m, 2H), 1.64 (d, J = 5.1 Hz, 3H).

^{13}C NMR (126 MHz, $CDCl_3$, 25 ºC, TMS) δ 170.1, 145.9, 142.3, 137.1, 129.6, 128.6, 128.3, 128.1, 127.9, 126.9, 126.1, 125.6, 117.7, 51.6, 17.7.

HRMS (ESI) m/z calcd for $C_{19}H_{20}NO$ $[M+H]^+$ 278.1539, found 278.1538.

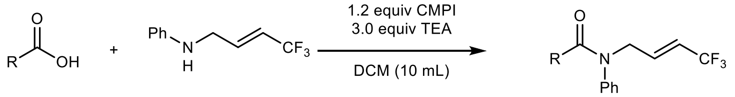

S1 and **S2**

To a mixture of carboxylic acid (2.4 mmol), (*E*)-*N*-(but-2-en-1-yl)aniline (2 mmol, 294 mg), and 2-chloro-1-methylpyridinium iodide (CMPI, 2.4 mmol, 612 mg) in anhydrous 10 mL of DCM was added triethylamine (TEA, 6 mmol, 0.84 mL). The reaction mixture was stirred at 50 °C for overnight. The resulting mixture was cooled to room temperature, quenched with water (40 mL), and extracted with EtOAc (20 mL × 3). The combined organic phases were dried over anhydrous Na_2SO_4, filtered, and concentrated under reduced pressure and the residue was purified by column chromatography (Hex/EA = 10:1) to give substrates **S1** (412 mg, 62%, white solid) and **S2** (268 mg, 39%, colorless oil).

***N*-phenyl-*N*-((*E*)-4,4,4-trifluorobut-2-en-1-yl)cinnamamide (S1)**

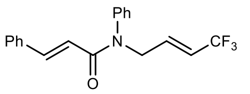

^{1}H NMR (500 MHz, $CDCl_3$, 25 ºC, TMS) δ 7.75 (d, J = 15.5 Hz, 1H), 7.51 – 7.46 (m, 2H), 7.46 – 7.40 (m, 1H), 7.37 – 7.29 (m, 5H), 7.25 – 7.20 (m, 2H), 6.57 – 6.46 (m, 1H), 6.35 (d, J = 15.5 Hz, 1H), 5.83 – 5.71 (m, 1H), 4.62 – 4.48 (m, 2H).

^{13}**C NMR** (126 MHz, $CDCl_3$, 25 ºC, TMS) δ 165.9, 142.9, 141.7, 135.1 (q, $J_{C\text{-}F}$ = 6.4 Hz), 134.9, 129.9, 129.8, 128.7, 128.2, 128.0, 127.9, 122.7 (q, $J_{C\text{-}F}$ = 269.5 Hz), 120.9 (q, $J_{C\text{-}F}$ = 34.1 Hz), 118.1, 50.1.

^{19}**F NMR** (471 MHz, $CDCl_3$) δ -63.31 (d, J = 4.1 Hz).

HRMS (ESI) m/z calcd for $C_{19}H_{17}F_3NO$ $[M+H]^+$ 332.1257, found 332.1265.

(*E*)-*N*,3-diphenyl-*N*-(4,4,4-trifluorobut-2-en-1-yl)but-3-enamide (S2)

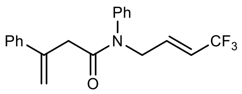

^{1}**H NMR** (500 MHz, $CDCl_3$, 25 ºC, TMS) δ 7.44 – 7.35 (m, 3H), 7.29 – 7.23 (m, 3H), 7.22 – 7.18 (m, 2H), 7.15 – 7.11 (m, 2H), 6.38 – 6.27 (m, 1H), 5.61 – 5.49 (m, 1H), 5.38 (s, 1H), 5.05 (s, 1H), 4.40 – 4.29 (m, 2H), 3.35 (s, 2H).

^{13}**C NMR** (126 MHz, $CDCl_3$, 25 ºC, TMS) δ 170.4, 142.2, 142.1, 140.2, 134.8 (q, $J_{C\text{-}F}$ = 6.4 Hz), 129.9, 128.5, 128.3, 127.9, 127.8, 126.0, 122.6 (q, $J_{C\text{-}F}$ = 269.6 Hz), 120.9 (q, $J_{C\text{-}F}$ = 33.8 Hz), 115.9, 49.9, 41.3.

^{19}**F NMR** (471 MHz, $CDCl_3$) δ -63.36 (d, J = 6.2 Hz).

HRMS (ESI) m/z calcd for $C_{20}H_{19}F_3NO$ $[M+H]^+$ 346.1413, found 346.1419.

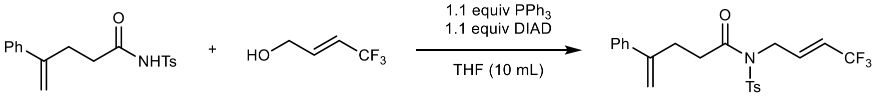

S3

To a mixture of 4,4,4-trifluorobut-2-en-1-ol (2.0 mmol) and alkynoic amide (2.2 mmol) in THF (10 mL) was added Ph_3P (2.2 mmol) and DIAD (2.2 mmol) under Ar at 0 ºC. The mixture was stirred at room temperature for 12 hours. The solvent was evaporated and the residue was purified by column chromatography (Hex/EA = 5:1) to give the desired product **S3** as a colorless oil (568 mg, 65%).

(*E*)-4-phenyl-*N*-tosyl-*N*-(4,4,4-trifluorobut-2-en-1-yl)pent-4-enamide (S3)

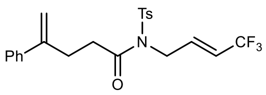

^{1}**H NMR** (500 MHz, $CDCl_3$, 25 ºC, TMS) δ 7.68 – 7.57 (m, 2H), 7.33 – 7.27 (m, 5H), 7.27 – 7.20 (m, 2H), 6.43 – 6.24 (m, 1H), 5.89 – 5.62 (m, 1H), 5.25 (d, J = 1.1 Hz, 1H), 5.00 (d, J = 1.4 Hz, 1H), 4.51 (dt, J = 5.7, 2.1 Hz, 2H), 2.78 (s, 4H), 2.41 (s, 3H).

^{13}**C NMR** (126 MHz, $CDCl_3$, 25 ºC, TMS) δ 172.0, 146.5, 145.4, 140.2, 136.1, 134.8 (q, $J_{C\text{-}F}$ = 6.5 Hz), 130.0, 128.4, 127.6, 127.6, 126.1, 122.6 (q, $J_{C\text{-}F}$ = 269.8 Hz), 121.3 (q, $J_{C\text{-}F}$ = 34.2 Hz), 113.3, 46.0, 35.3, 30.1, 21.6.

^{19}**F NMR** (471 MHz, $CDCl_3$) δ -63.44 (d, J = 3.6 Hz).

HRMS (ESI) m/z calcd for $C_{22}H_{23}F_3NO_3S$ $[M+H]^+$ 438.1345, found 438.1353.

2.2 General Procedure for the Synthesis of Products 3a-3y

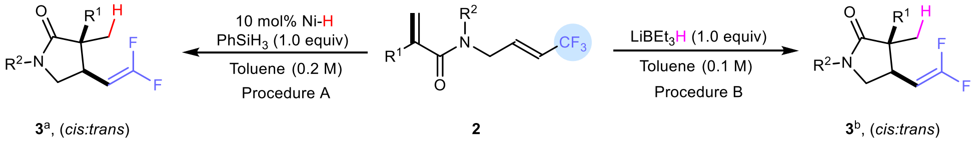

Procedure A: A solution of substrate **2** (0.1 mmol), $PhSiH_3$ (0.1 mmol, 10.8 mg), and 10 mol% Ni-H (4.4 mg) in toluene (0.5 mL) was stirred in a 5 mL of vial under argon atmosphere at room temperature for 12 hours. The reaction was monitored by TLC until the full conversion of substrate **2**. Then the solvent was removed under reduced pressure. The residue was purified by column chromatography (7% - 10% ethyl acetate in hexane) to give the corresponding ***cis*-3** or ***trans*-3**. The yield and diastereoselectivity of products **3** were determined by ^{19}F NMR of the crude reaction mixture using fluorobenzene as an internal standard, as shown in Fig. 4.

Procedure B: To a solution of substrate **2** (0.1 mmol) in toluene (1 mL) in a 5 mL of vial was added $LiBEt_3H$ (1.0

equiv, 1 M in THF) under argon atmosphere at room temperature for 0.5 hours. The reaction was monitored by TLC until the full conversion of substrate **2**. Then the reaction was quenched by H_2O (5 mL, *Caution: Gas release*) and extracted with EtOAc (5 mL × 3). The combined organic extracts were dried with Na_2SO_4, filtered, and concentrated under reduced pressure. The residue was purified by column chromatography (7% - 10% ethyl acetate in hexane) to give the corresponding ***cis*-3** or ***trans*-3**. The yield and diastereoselectivity of product **3** were determined by ^{19}F NMR of the crude reaction mixture using fluorobenzene as an internal standard, as shown in Fig. 4.

cis-4-(2,2-difluorovinyl)-3-methyl-1,3-diphenylpyrrolidin-2-one (_cis_-3a)

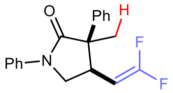

Prepared according to **Procedure A** and purified by flash column chromatograph using hexane/ethyl acetate (15:1) as eluent to give ***cis*-3a** as a white solid. Yield of **3a**: 93% (*dr* = 11:1), determined by ^{19}F NMR of the crude reaction mixture using fluorobenzene as an internal standard.

^{1}H NMR (500 MHz, $CDCl_3$, 25 ºC, TMS) δ 7.76 – 7.69 (m, 2H), 7.44 – 7.38 (m, 2H), 7.35 – 7.28 (m, 2H), 7.27 – 7.23 (m, 1H), 7.22 – 7.17 (m, 1H), 7.14 – 7.08 (m, 2H), 3.82 (dd, *J* = 9.7, 7.7 Hz, 1H), 3.61 (ddd, *J* = 24.4, 10.4, 2.2 Hz, 1H), 3.47 (t, *J* = 9.9 Hz, 1H), 3.27 – 3.17 (m, 1H), 1.68 (s, 3H).

^{13}C NMR (126 MHz, $CDCl_3$, 25 ºC, TMS) δ 176.4, 157.1 (dd, $J_{C\text{-}F}$ = 290.3, 288.6 Hz), 138.9, 138.4, 129.1, 128.7, 127.4, 126.9, 125.0, 120.0, 75.74 (dd, $J_{C\text{-}F}$ = 25.1, 17.5 Hz), 53.54 (d, $J_{C\text{-}F}$ = 2.0 Hz), 50.25 (t, $J_{C\text{-}F}$ = 3.0 Hz), 39.98 (d, $J_{C\text{-}F}$ = 5.1 Hz), 22.9.

^{19}F NMR (471 MHz, $CDCl_3$) δ -82.41 (d, *J* = 39.2 Hz), -87.93 (dd, *J* = 39.2, 24.4 Hz).

HRMS (ESI) m/z calcd for $C_{19}H_{18}F_2NO$ $[M+H]^+$ 314.1351, found 314.1356.

cis-4-(2,2-difluorovinyl)-3-(4-methoxyphenyl)-3-methyl-1-phenylpyrrolidin-2-one (_cis_-3b)

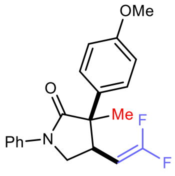

Prepared according to **Procedure A** and purified by flash column chromatograph using hexane/ethyl acetate (15:1) as eluent to give ***cis*-3b** as a white solid. Yield of **3b**: 94% (*dr* = 5:1), determined by ^{19}F NMR of the crude reaction mixture using fluorobenzene as an internal standard.

^{1}H NMR (500 MHz, $CDCl_3$, 25 ºC, TMS) δ 7.77 – 7.67 (m, 2H), 7.46 – 7.37 (m, 2H), 7.23 – 7.16 (m, 1H), 7.07 – 6.99 (m, 2H), 6.89 – 6.81 (m, 2H), 3.81 (dd, *J* = 9.7, 7.7 Hz, 1H), 3.77 (s, 3H), 3.65 (ddd, *J* = 24.5, 10.3, 2.3 Hz, 1H), 3.46 (t, *J* = 9.9 Hz, 1H), 3.24 – 3.14 (m, 1H), 1.65 (s, 3H).

^{13}C NMR (126 MHz, $CDCl_3$, 25 ºC, TMS) δ 176.6, 158.7, 157.0 (dd, $J_{C\text{-}F}$ = 290.1, 288.4 Hz), 138.9, 130.4, 129.0, 128.0, 124.9, 120.0, 114.1, 75.8 (dd, $J_{C\text{-}F}$ = 25.1, 17.5 Hz), 55.3, 52.9, 50.2 (t, $J_{C\text{-}F}$ = 2.9 Hz), 40.0 (d, $J_{C\text{-}F}$ = 5.2 Hz), 23.0.

^{19}F NMR (471 MHz, $CDCl_3$) δ -82.50 (d, *J* = 39.5 Hz), -88.01 (dd, *J* = 39.5, 24.5 Hz).

HRMS (ESI) m/z calcd for $C_{20}H_{20}F_2NO_2$ $[M+H]^+$ 344.1457, found 344.1459.

cis-4-(2,2-difluorovinyl)-3-methyl-1-phenyl-3-(_p_-tolyl)pyrrolidin-2-one (_cis_-3c)

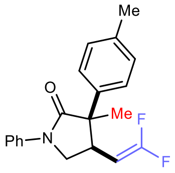

Prepared according to **Procedure A** and purified by flash column chromatograph using hexane/ethyl acetate (15:1) as eluent to give ***cis*-3c** as a white solid. Yield of **3c**: 91% (*dr* = 9:1), determined by ^{19}F NMR of the crude reaction mixture using fluorobenzene as an internal standard.

^{1}H NMR (500 MHz, $CDCl_3$, 25 ºC, TMS) δ 7.77 – 7.71 (m, 2H), 7.43 (dd, *J* = 8.7, 7.4 Hz, 2H), 7.24 – 7.18 (m, 1H), 7.17 – 7.11 (m, 2H), 7.04 – 6.98 (m, 2H), 3.82 (dd, *J* = 9.6, 7.7 Hz, 1H), 3.66 (ddd, *J* = 24.5, 10.4, 2.2 Hz, 1H), 3.48 (t, *J* = 9.9 Hz, 1H), 3.26 – 3.17 (m, 1H), 2.33 (s, 3H), 1.68 (s, 3H).

^{13}C NMR (126 MHz, $CDCl_3$, 25 ºC, TMS) δ 176.5, 157.0 (dd, $J_{C\text{-}F}$ = 290.4, 288.3 Hz), 138.9, 137.1, 135.3, 129.4, 129.0, 126.8, 124.9, 120.0, 75.8 (dd, $J_{C\text{-}F}$ = 25.1, 17.6 Hz), 53.2 (t, $J_{C\text{-}F}$ = 1.9 Hz), 50.3 (t, $J_{C\text{-}F}$ = 3.0 Hz), 39.9 (d, $J_{C\text{-}F}$ = 5.1 Hz), 22.9, 20.9.

^{19}F NMR (471 MHz, CDCl$_3$) δ -82.54 (d, J = 39.5 Hz), -88.07 (dd, J = 39.6, 24.5 Hz).

HRMS (ESI) m/z calcd for $C_{20}H_{20}F_2NO$ [M+H]$^+$ 328.1507, found 328.1506.

***cis*-3-(4-(*tert*-butyl)phenyl)-4-(2,2-difluorovinyl)-3-methyl-1-phenylpyrrolidin-2-one (*cis*-3d)**

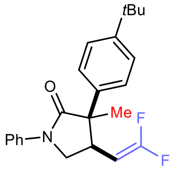

Prepared according to **Procedure A** and purified by flash column chromatograph using hexane/ethyl acetate (15:1) as eluent to give ***cis*-3d** as a white solid. Yield of **3d**: 88% (*dr* = 14:1), determined by ^{19}F NMR of the crude reaction mixture using fluorobenzene as an internal standard.

^{1}H NMR (500 MHz, CDCl$_3$, 25 ºC, TMS) δ 7.77 – 7.68 (m, 2H), 7.46 – 7.39 (m, 2H), 7.34 – 7.30 (m, 2H), 7.23 – 7.17 (m, 1H), 7.06 – 7.01 (m, 2H), 3.81 (dd, J = 9.6, 7.7 Hz, 1H), 3.68 (ddd, J = 24.5, 10.4, 2.2 Hz, 1H), 3.49 (t, J = 9.9 Hz, 1H), 3.27 – 3.16 (m, 1H), 1.67 (s, 3H), 1.29 (s, 9H).

^{13}C NMR (126 MHz, CDCl$_3$, 25 ºC, TMS) δ 176.6, 157.1 (dd, $J_{C\text{-}F}$ = 290.1, 288.4 Hz), 150.2, 138.9, 135.2, 129.0, 126.6, 125.6, 124.9, 120.0, 75.8 (dd, $J_{C\text{-}F}$ = 25.1, 17.5 Hz), 53.1, 50.3 (t, $J_{C\text{-}F}$ = 3.0 Hz), 40.0 (d, $J_{C\text{-}F}$ = 5.1 Hz), 34.4, 31.3, 22.8.

^{19}F NMR (471 MHz, CDCl$_3$) δ -82.53 (d, J = 39.6 Hz), -88.13 (dd, J = 39.7, 24.5 Hz).

HRMS (ESI) m/z calcd for $C_{23}H_{26}F_2NO$ [M+H]$^+$ 370.1977, found 370.1979.

***cis*-4-(2,2-difluorovinyl)-3-(4-fluorophenyl)-3-methyl-1-phenylpyrrolidin-2-one (*cis*-3e)**

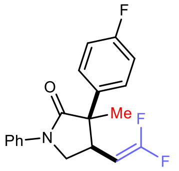

Prepared according to **Procedure A** and purified by flash column chromatograph using hexane/ethyl acetate (15:1) as eluent to give ***cis*-3e** as a white solid. Yield of **3e**: 95% (*dr* = 5:1), determined by ^{19}F NMR of the crude reaction mixture using fluorobenzene as an internal standard.

^{1}H NMR (500 MHz, CDCl$_3$, 25 ºC, TMS) δ 7.75 – 7.67 (m, 2H), 7.46 – 7.38 (m, 2H), 7.24 – 7.18 (m, 1H), 7.13 – 7.06 (m, 2H), 7.05 – 6.97 (m, 2H), 3.84 (dd, J = 9.8, 7.7 Hz, 1H), 3.61 (ddd, J = 24.3, 10.4, 2.2 Hz, 1H), 3.45 (t, J = 9.8 Hz, 1H), 3.27 – 3.17 (m, 1H), 1.67 (s, 3H).

^{13}C NMR (126 MHz, CDCl$_3$, 25 ºC, TMS) δ 176.1, 161.9 (d, $J_{C\text{-}F}$ = 246.7 Hz), 157.1 (dd, $J_{C\text{-}F}$ = 290.5, 288.8 Hz), 138.7, 134.3 (d, $J_{C\text{-}F}$ = 3.5 Hz), 129.1, 128.7 (d, $J_{C\text{-}F}$ = 8.0 Hz), 125.1, 120.0, 115.6 (d, $J_{C\text{-}F}$ = 21.3 Hz), 75.6 (dd, $J_{C\text{-}F}$ = 25.1, 17.7 Hz), 53.1 (t, $J_{C\text{-}F}$ = 2.0 Hz), 50.2 (t, $J_{C\text{-}F}$ = 3.0 Hz), 39.9 (d, $J_{C\text{-}F}$ = 5.1 Hz), 23.1.

^{19}F NMR (471 MHz, CDCl$_3$) δ -82.13 (d, J = 38.3 Hz), -87.53 (dd, J = 38.4, 24.3 Hz), -114.07 (tt, J = 8.7, 5.1 Hz).

HRMS (ESI) m/z calcd for $C_{19}H_{17}F_3NO$ [M+H]$^+$ 332.1257, found 332.1259.

***cis*-3-(4-chlorophenyl)-4-(2,2-difluorovinyl)-3-methyl-1-phenylpyrrolidin-2-one (*cis*-3f)**

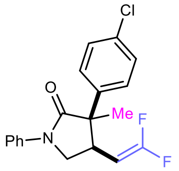

Prepared according to **Procedure B** and purified by flash column chromatograph using hexane/ethyl acetate (15:1) as eluent to give ***cis*-3f** as a colorless oil. Yield of **3f**: 80% (*dr* = 2.4:1), determined by ^{19}F NMR of the crude reaction mixture using fluorobenzene as an internal standard.

^{1}H NMR (500 MHz, CDCl$_3$, 25 ºC, TMS) δ 7.71 (d, J = 8.0 Hz, 2H), 7.49 – 7.37 (m, 2H), 7.30 (d, J = 8.2 Hz, 2H), 7.22 (t, J = 7.4 Hz, 1H), 7.06 (d, J = 8.2 Hz, 2H), 3.85 (dd, J = 9.8, 7.7 Hz, 1H), 3.62 (ddd, J = 24.2, 10.4, 2.2 Hz, 1H), 3.45 (t, J = 9.8 Hz, 1H), 3.29 – 3.17 (m, 1H), 1.67 (s, 3H).

^{13}C NMR (126 MHz, CDCl$_3$, 25 ºC, TMS) δ 175.8, 159.4 – 154.8 (m), 138.7, 137.1, 133.4, 129.1, 128.9, 128.4, 125.2, 120.0, 75.5 (dd, $J_{C\text{-}F}$ = 25.1, 17.6 Hz), 53.2 (m), 50.2 (t, $J_{C\text{-}F}$ = 2.9 Hz), 39.8 (d, $J_{C\text{-}F}$ = 5.1 Hz), 23.0.

^{19}F NMR (471 MHz, CDCl$_3$) δ -81.93 (d, J = 37.9 Hz), -87.34 (dd, J = 38.0, 24.2 Hz).

HRMS (ESI) m/z calcd for $C_{19}H_{17}ClF_2NO$ [M+H]$^+$ 348.0961, found 348.0958.

cis-3-(4-bromophenyl)-4-(2,2-difluorovinyl)-3-methyl-1-phenylpyrrolidin-2-one (cis-3g)

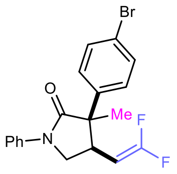

Prepared according to **Procedure B** and purified by flash column chromatograph using hexane/ethyl acetate (15:1) as eluent to give ***cis*-3g** as a colorless oil. Yield of **3g**: 83% (*dr* = 2.4:1), determined by ^{19}F NMR of the crude reaction mixture using fluorobenzene as an internal standard.

^{1}H NMR (500 MHz, $CDCl_3$, 25 ºC, TMS) δ 7.74 – 7.67 (m, 2H), 7.49 – 7.38 (m, 4H), 7.25 – 7.19 (m, 1H), 7.04 – 6.96 (m, 2H), 3.85 (dd, *J* = 9.8, 7.7 Hz, 1H), 3.62 (ddd, *J* = 24.2, 10.4, 2.1 Hz, 1H), 3.45 (t, *J* = 9.9 Hz, 1H), 3.28 – 3.17 (m, 1H), 1.66 (s, 3H).

^{13}C NMR (126 MHz, $CDCl_3$, 25 ºC, TMS) δ 175.7, 157.1 (dd, J_{C-F} = 290.3, 288.6 Hz), 138.7, 137.6, 131.8, 129.1, 128.8, 125.2, 121.5, 120.0, 75.5 (dd, J_{C-F} = 25.2, 17.7 Hz), 53.3 (m), 50.2 (t, J_{C-F} = 2.9 Hz), 39.8 (d, J_{C-F} = 5.1 Hz), 22.9.

^{19}F NMR (471 MHz, $CDCl_3$) δ -81.89 (d, *J* = 37.8 Hz), -87.31 (dd, *J* = 37.9, 24.2 Hz).

HRMS (ESI) m/z calcd for $C_{19}H_{17}BrF_2NO$ $[M+H]^+$ 392.0456, found 392.0452.

cis-3-(2-bromophenyl)-4-(2,2-difluorovinyl)-3-methyl-1-phenylpyrrolidin-2-one (cis-3h)

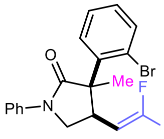

Prepared according to **Procedure B** and purified by flash column chromatograph using hexane/ethyl acetate (15:1) as eluent to give ***cis*-3h** as a colorless oil. Yield of **3h**: 75% (*dr* = 5:1), determined by ^{19}F NMR of the crude reaction mixture using fluorobenzene as an internal standard.

^{1}H NMR (500 MHz, $CDCl_3$, 25 ºC, TMS) δ 7.73 – 7.64 (m, 3H), 7.60 (dd, *J* = 7.9, 1.4 Hz, 1H), 7.46 – 7.38 (m, 2H), 7.35 – 7.28 (m, 1H), 7.23 – 7.17 (m, 1H), 7.13 (td, *J* = 7.6, 1.7 Hz, 1H), 4.16 (dd, *J* = 10.1, 7.9 Hz, 1H), 4.03 (ddd, *J* = 24.4, 10.5, 2.2 Hz, 1H), 3.70 (dd, *J* = 9.8, 5.3 Hz, 1H), 3.60 – 3.46 (m, 1H), 1.91 (s, 3H).

^{13}C NMR (126 MHz, $CDCl_3$, 25 ºC, TMS) δ 175.2, 156.6 (dd, J_{C-F} = 291.1, 288.4 Hz), 139.2, 139.2, 135.1, 130.5, 129.0, 128.9, 127.5, 125.1, 123.2, 120.4, 78.7 (dd, J_{C-F} = 24.8, 18.0 Hz), 54.6 (m), 52.1 (m), 37.7 (d, J_{C-F} = 5.2 Hz), 26.5.

^{19}F NMR (471 MHz, $CDCl_3$) δ -85.10 (d, *J* = 39.9 Hz), -87.45 (dd, *J* = 39.9, 24.4 Hz).

HRMS (ESI) m/z calcd for $C_{19}H_{17}BrF_2NO$ $[M+H]^+$ 392.0456, found 392.0457.

cis-4-(2,2-difluorovinyl)-3-methyl-1-phenyl-3-(4-(trifluoromethyl)phenyl)pyrrolidin-2-one (cis-3i)

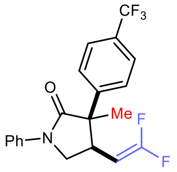

Prepared according to **Procedure A** and purified by flash column chromatograph using hexane/ethyl acetate (15:1) as eluent to give ***cis*-3i** as a colorless oil. Yield of **3i**: 98% (*dr* = 8:1), determined by ^{19}F NMR of the crude reaction mixture using fluorobenzene as an internal standard.

^{1}H NMR (500 MHz, $CDCl_3$, 25 ºC, TMS) δ 7.72 (d, *J* = 8.3 Hz, 2H), 7.60 (d, *J* = 8.2 Hz, 2H), 7.48 – 7.39 (m, 2H), 7.32 – 7.25 (m, 2H), 7.23 (t, *J* = 7.4 Hz, 1H), 3.89 (dd, *J* = 9.9, 7.6 Hz, 1H), 3.61 (ddd, *J* = 24.0, 10.4, 2.1 Hz, 1H), 3.48 (t, *J* = 9.8 Hz, 1H), 3.35 – 3.23 (m, 1H), 1.72 (s, 3H).

^{13}C NMR (126 MHz, $CDCl_3$, 25 ºC, TMS) δ 175.5, 158.4 (dd, J_{C-F} = 291.1, 289.3 Hz), 142.8, 138.6, 129.8 (q, J_{C-F} = 32.8 Hz), 129.1, 127.5, 125.6 (q, J_{C-F} = 3.8 Hz), 125.3, 124.0 (q, J_{C-F} = 272.0 Hz), 120.1, 75.5 (dd, J_{C-F} = 25.3, 17.9 Hz), 53.6 (m), 50.3 (t, J_{C-F} = 2.9 Hz), 39.9 (d, J_{C-F} = 5.1 Hz), 22.9.

^{19}F NMR (471 MHz, $CDCl_3$) δ -61.82, -81.78 (d, *J* = 37.3 Hz), -87.07 (dd, *J* = 37.3, 24.0 Hz).

HRMS (ESI) m/z calcd for $C_{20}H_{17}F_5NO$ $[M+H]^+$ 382.1225, found 382.1229.

cis-4-(2,2-difluorovinyl)-3-methyl-1-phenyl-3-(3-(trifluoromethyl)phenyl)pyrrolidin-2-one (cis-3j)

Prepared according to **Procedure A** and purified by flash column chromatograph using hexane/ethyl acetate (15:1) as eluent to give ***cis*-3j** as a white solid. Yield of **3j**: 90% (*dr* = 8:1), determined by ^{19}F NMR of the crude reaction mixture

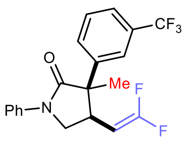

using fluorobenzene as an internal standard.

¹H NMR (500 MHz, $CDCl_3$, 25 ºC, TMS) δ 7.74 – 7.66 (m, 2H), 7.58 – 7.51 (m, 1H), 7.49 – 7.40 (m, 4H), 7.34 – 7.29 (m, 1H), 7.25 – 7.19 (m, 1H), 3.89 (dd, J = 9.9, 7.6 Hz, 1H), 3.57 (ddd, J = 24.0, 10.4, 2.1 Hz, 1H), 3.48 (t, J = 9.7 Hz, 1H), 3.32 – 3.23 (m, 1H), 1.72 (s, 3H).

¹³C NMR (126 MHz, $CDCl_3$, 25 ºC, TMS) δ 176.4, 157.1 (dd, $J_{C\text{-}F}$ = 290.3, 288.6 Hz), 138.9, 138.4, 129.1, 128.7, 127.4, 126.9, 125.0, 120.0, 75.7 (dd, $J_{C\text{-}F}$ = 25.1, 17.5 Hz), 53.5 (d, $J_{C\text{-}F}$ = 2.0 Hz), 50.3 (t, $J_{C\text{-}F}$ = 3.0 Hz), 40.0 (d, $J_{C\text{-}F}$ = 5.1 Hz), 22.9.

¹⁹F NMR (471 MHz, $CDCl_3$) δ -61.80, -81.91 (d, J = 37.0 Hz), -86.95 (dd, J = 37.2, 24.0 Hz).

HRMS (ESI) m/z calcd for $C_{20}H_{17}F_5NO$ $[M+H]^+$ 382.1225, found 382.122.

***cis*-4-(2,2-difluorovinyl)-3-methyl-1,3-diphenylpyrrolidin-2-one (*cis*-3k)**

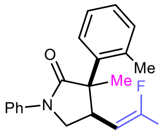

Prepared according to **Procedure B** and purified by flash column chromatograph using hexane/ethyl acetate (15:1) as eluent to give ***cis*-3k** as a colorless oil. Yield of **3k**: 96% (*dr* = 3.4:1), determined by ¹⁹F NMR of the crude reaction mixture using fluorobenzene as an internal standard.

¹H NMR (500 MHz, $CDCl_3$, 25 ºC, TMS) δ 7.73 – 7.66 (m, 2H), 7.45 – 7.38 (m, 2H), 7.23 – 7.18 (m, 2H), 7.17 – 7.11 (m, 3H), 3.97 (dd, J = 9.8, 8.0 Hz, 1H), 3.79 (ddd, J = 24.3, 10.6, 2.3 Hz, 1H), 3.51 (dd, J = 9.8, 7.7 Hz, 1H), 3.34 – 3.25 (m, 1H), 2.36 (s, 3H), 1.83 (s, 3H).

¹³C NMR (126 MHz, $CDCl_3$, 25 ºC, TMS) δ 177.1, 159.0 – 154.4 (m), 139.0, 137.2, 136.7, 132.9, 129.0, 128.5, 127.4, 126.3, 125.0, 120.1, 77.7 (dd, $J_{C\text{-}F}$ = 24.8, 17.8 Hz), 54.2 (m), 51.2 (t, $J_{C\text{-}F}$ = 3.1 Hz), 39.5 (d, $J_{C\text{-}F}$ = 5.1 Hz), 26.1, 22.7.

¹⁹F NMR (471 MHz, $CDCl_3$) δ -84.12 (d, J = 40.7 Hz), -88.31 (dd, J = 40.6, 24.3 Hz).

HRMS (ESI) m/z calcd for $C_{20}H_{20}F_2NO$ $[M+H]^+$ 328.1507, found 328.1508.

***cis*-4-(2,2-difluorovinyl)-3-methyl-3-(naphthalen-1-yl)-1-phenylpyrrolidin-2-one (*cis*-3l)**

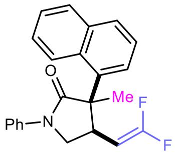

Prepared according to **Procedure B** and purified by flash column chromatograph using hexane/ethyl acetate (15:1) as eluent to give ***cis*-3l** as a white solid. Yield of **3l**: 72% (*dr* = 3:1), determined by ¹⁹F NMR of the crude reaction mixture using fluorobenzene as an internal standard.

¹H NMR (500 MHz, $CDCl_3$, 25 ºC, TMS) δ 8.05 – 7.99 (m, 1H), 7.89 – 7.83 (m, 1H), 7.77 (d, J = 8.1 Hz, 1H), 7.76 – 7.72 (m, 2H), 7.63 (dd, J = 7.4, 1.2 Hz, 1H), 7.48 – 7.41 (m, 4H), 7.39 (dd, J = 8.1, 7.4 Hz, 1H), 7.27 – 7.19 (m, 1H), 4.16 – 4.09 (m, 1H), 3.83 (ddd, J = 24.1, 10.6, 2.2 Hz, 1H), 3.66 – 3.55 (m, 2H), 2.03 (s, 3H).

¹³C NMR (126 MHz, $CDCl_3$, 25 ºC, TMS) δ 176.9, 159.5 – 153.9 (m), 139.1, 135.5, 135.0, 131.9, 129.7, 129.1, 128.9, 126.8, 125.6, 125.5, 125.2, 125.2, 125.2, 120.4, 78.9 (dd, $J_{C\text{-}F}$ = 24.7, 17.9 Hz), 54.5 (m), 51.4 (t, $J_{C\text{-}F}$ = 2.9 Hz), 39.6 (d, $J_{C\text{-}F}$ = 5.0 Hz), 26.4.

¹⁹F NMR (471 MHz, $CDCl_3$) δ -84.77 (d, J = 40.5 Hz), -88.70 (dd, J = 40.5, 24.1 Hz).

HRMS (ESI) m/z calcd for $C_{23}H_{20}F_2NO$ $[M+H]^+$ 364.1507, found 364.1509.

***cis*-4-(2,2-difluorovinyl)-3-(2-fluoro-[1,1'-biphenyl]-4-yl)-3-methyl-1-phenylpyrrolidin-2-one (*cis*-3m)**

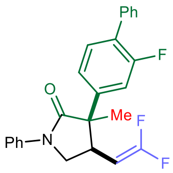

Prepared according to **Procedure A** and purified by flash column chromatograph using hexane/ethyl acetate (15:1) as eluent to give ***cis*-3m** as a white solid. Yield of **3m**: 94% (*dr* = 5:1), determined by ¹⁹F NMR of the crude reaction mixture using fluorobenzene as an internal standard.

¹H NMR (500 MHz, $CDCl_3$, 25 ºC, TMS) δ 7.76 – 7.70 (m, 2H), 7.55 – 7.49 (m, 2H), 7.46 – 7.37 (m, 5H), 7.37 – 7.33 (m, 1H), 7.24 – 7.19 (m, 1H), 6.98 (t, J = 1.0 Hz, 1H), 6.97 – 6.94 (m, 1H),

3.88 (dd, J = 9.8, 7.7 Hz, 1H), 3.74 (ddd, J = 24.2, 10.4, 2.1 Hz, 1H), 3.54 (t, J = 9.9 Hz, 1H), 3.31 – 3.21 (m, 1H), 1.70 (s, 3H).

^{13}C NMR (126 MHz, $CDCl_3$, 25 °C, TMS) δ 175.6, 159.8 (d, $J_{C\text{-}F}$ = 248.8 Hz), 157.2 (dd, $J_{C\text{-}F}$ = 290.6, 288.5 Hz), 140.2 (d, $J_{C\text{-}F}$ = 7.0 Hz), 138.7, 135.2, 130.9 (d, $J_{C\text{-}F}$ = 4.1 Hz), 129.1, 128.9 (d, $J_{C\text{-}F}$ = 3.0 Hz), 128.5, 128.1 (d, $J_{C\text{-}F}$ = 13.6 Hz), 127.9, 125.2, 122.9 (d, $J_{C\text{-}F}$ = 3.4 Hz), 120.1, 115.1 (d, $J_{C\text{-}F}$ = 24.2 Hz), 75.6 (dd, $J_{C\text{-}F}$ = 25.2, 17.7 Hz), 53.3 (m), 50.4 (t, $J_{C\text{-}F}$ = 2.9 Hz), 39.9 (d, $J_{C\text{-}F}$ = 5.1 Hz), 22.9.

^{19}F NMR (471 MHz, $CDCl_3$) δ -81.88 (d, J = 38.0 Hz), -87.27 (dd, J = 38.0, 24.2 Hz), -115.57 – -115.73 (m).

HRMS (ESI) m/z calcd for $C_{25}H_{21}F_3NO$ $[M+H]^+$ 408.1570, found 408.1577.

***cis*-4-(2,2-difluorovinyl)-3-methyl-1-phenylpyrrolidin-2-one (*cis*-3n)**

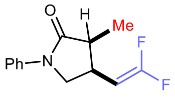

Prepared according to **Procedure A** and purified by flash column chromatograph using hexane/ethyl acetate (15:1) as eluent to give ***cis*-3n** as a white solid. Yield of **3n**: 35% (*dr* = 2.5:1), determined by ^{19}F NMR of the crude reaction mixture using fluorobenzene as an internal standard.

^{1}H NMR (400 MHz, $CDCl_3$, 25 °C, TMS) δ 7.66 – 7.53 (m, 2H), 7.42 – 7.31 (m, 2H), 7.14 (t, J = 7.4 Hz, 1H), 4.26 (ddd, J = 24.4, 9.6, 2.0 Hz, 1H), 3.87 (dd, J = 9.6, 8.0 Hz, 1H), 3.53 (t, J = 9.6 Hz, 1H), 2.89 – 2.74 (m, 1H), 2.37 (dq, J = 10.8, 7.0 Hz, 1H), 1.29 (d, J = 7.0 Hz, 3H).

^{13}C NMR (101 MHz, $CDCl_3$, 25 °C, TMS) δ 174.6, 157.6 (d, $J_{C\text{-}F}$ = 290.7, 288.7 Hz), 139.1, 128.9, 124.6, 119.7, 78.3 (dd, $J_{C\text{-}F}$ = 24.0, 19.2 Hz), 51.7 (t, $J_{C\text{-}F}$ = 2.9 Hz), 44.7 (t, $J_{C\text{-}F}$ = 2.1 Hz), 35.6 (d, $J_{C\text{-}F}$ = 5.1 Hz), 14.0.

^{19}F NMR (376 MHz, $CDCl_3$) δ -84.71 (d, J = 40.6 Hz), -88.37 (dd, J = 40.6, 24.4 Hz).

HRMS (ESI) m/z calcd for $C_{13}H_{14}F_2NO$ $[M+H]^+$ 238.1038, found 238.1042.

4-(2,2-difluorovinyl)-3,3-dimethyl-1-phenylpyrrolidin-2-one (3o)

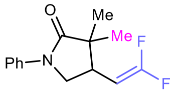

Prepared according to **Procedure B** and purified by flash column chromatograph using hexane/ethyl acetate (15:1) as eluent to give **3o** as a white solid. Yield of **3o**: 62%, determined by ^{19}F NMR of the crude reaction mixture using fluorobenzene as an internal standard.

^{1}H NMR (500 MHz, $CDCl_3$, 25 °C, TMS) δ 7.65 – 7.59 (m, 2H), 7.40 – 7.33 (m, 2H), 7.14 (tt, J = 7.4, 1.1 Hz, 1H), 4.24 (ddd, J = 24.3, 10.1, 2.4 Hz, 1H), 3.84 (dd, J = 9.7, 7.8 Hz, 1H), 3.57 – 3.49 (m, 1H), 3.02 – 2.93 (m, 1H), 1.25 (s, 3H), 1.07 (s, 3H).

^{13}C NMR (126 MHz, $CDCl_3$, 25 °C, TMS) δ 177.7, 157.3 (dd, $J_{C\text{-}F}$ = 291.1, 288.4 Hz), 139.3, 128.9, 124.6, 119.7, 75.2 (dd, $J_{C\text{-}F}$ = 24.2, 19.0 Hz), 50.1 (t, $J_{C\text{-}F}$ = 2.9 Hz), 45.1 (t, $J_{C\text{-}F}$ = 2.0 Hz), 38.3 (d, $J_{C\text{-}F}$ = 4.9 Hz), 23.6, 19.1.

^{19}F NMR (471 MHz, $CDCl_3$) δ -82.86 (d, J = 39.7 Hz), -87.20 (dd, J = 39.7, 24.3 Hz).

HRMS (ESI) m/z calcd for $C_{14}H_{16}F_2NO$ $[M+H]^+$ 252.1194, found 252.1194.

***cis*-3-benzyl-4-(2,2-difluorovinyl)-3-methyl-1-phenylpyrrolidin-2-one (*cis*-3p)**

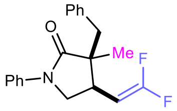

Prepared according to **Procedure B** and purified by flash column chromatograph using hexane/ethyl acetate (15:1) as eluent to give ***cis*-3p** as a white solid. Yield of **3p**: 82% (*dr* = 3:1), determined by ^{19}F NMR of the crude reaction mixture using fluorobenzene as an internal standard.

^{1}H NMR (500 MHz, $CDCl_3$, 25 °C, TMS) δ 7.44 – 7.37 (m, 2H), 7.36 – 7.30 (m, 2H), 7.19 – 7.08 (m, 6H), 4.54 (ddd, J = 24.6, 9.8, 2.4 Hz, 1H), 3.49 (dd, J = 9.4, 8.0 Hz, 1H), 3.11 – 3.00 (m, 1H), 2.92 (d, J = 13.9 Hz, 1H), 2.79 (t, J = 9.7 Hz, 1H), 2.75 (d, J = 13.9 Hz, 1H), 1.32 (s, 3H).

^{13}C NMR (126 MHz, $CDCl_3$, 25 °C, TMS) δ 176.3, 157.3 (dd, $J_{C\text{-}F}$ = 291.1, 288.4 Hz), 139.0, 136.9, 130.1, 128.8, 128.2, 126.9, 124.7, 120.1, 75.0 (dd, $J_{C\text{-}F}$ = 24.5, 18.3 Hz), 50.4 (t, $J_{C\text{-}F}$ = 2.5 Hz), 50.4 (m), 40.9, 38.8 (d, $J_{C\text{-}F}$ = 4.8

Hz), 24.0.

^{19}F NMR (471 MHz, $CDCl_3$) δ -82.40 (d, *J* = 38.7 Hz), -86.38 (dd, *J* = 38.8, 24.6 Hz).

HRMS (ESI) m/z calcd for $C_{20}H_{20}F_2NO$ $[M+H]^+$ 328.1507, found 328.1509.

***cis*-3-cyclopentyl-4-(2,2-difluorovinyl)-3-methyl-1-phenylpyrrolidin-2-one (*cis*-3q)**

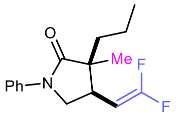

Prepared according to **Procedure B** and purified by flash column chromatograph using hexane/ethyl acetate (15:1) as eluent to give ***cis*-3q** as a colorless oil. Yield of **3q**: 62% (*dr* = 1.6:1), determined by ^{19}F NMR of the crude reaction mixture using fluorobenzene as an internal standard.

^{1}H NMR (500 MHz, $CDCl_3$, 25 °C, TMS) δ 7.67 – 7.59 (m, 2H), 7.40 – 7.33 (m, 2H), 7.17 – 7.11 (m, 1H), 4.31 (ddd, *J* = 24.2, 10.2, 2.4 Hz, 1H), 3.83 (dd, *J* = 9.7, 8.0 Hz, 1H), 3.56 (t, *J* = 9.5 Hz, 1H), 3.04 – 2.98 (m, 1H), 1.54 – 1.46 (m, 1H), 1.41 – 1.34 (m, 3H), 1.24 (s, 3H), 0.88 (t, *J* = 6.9 Hz, 3H).

^{13}C NMR (126 MHz, $CDCl_3$, 25 °C, TMS) δ 176.9, 157.3 (dd, J_{C-F} = 290.4 Hz, 288.2 Hz), 139.3, 128.9, 124.5, 119.7, 75.3 (dd, J_{C-F} = 24.1, 18.8 Hz), 50.6 (t, J_{C-F} = 2.8 Hz), 48.3 (m), 38.7 (d, J_{C-F} = 4.8 Hz), 36.2, 22.1, 17.6, 14.7.

^{19}F NMR (471 MHz, $CDCl_3$) δ -83.06 (d, *J* = 39.8 Hz), -87.11 (dd, *J* = 39.8, 24.2 Hz).

HRMS (ESI) m/z calcd for $C_{16}H_{20}F_2NO$ $[M+H]^+$ 280.1507, found 280.1513.

***cis*-4-(2,2-difluorovinyl)-3-isobutyl-3-methyl-1-phenylpyrrolidin-2-one (*cis*-3r)**

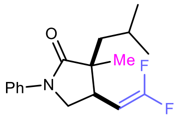

Prepared according to **Procedure B** and purified by flash column chromatograph using hexane/ethyl acetate (15:1) as eluent to give ***cis*-3r** and ***trans*-3r** as colorless oil, respectively. Yield of **3r**: 88% (*dr* = 1.2:1), determined by ^{19}F NMR of the crude reaction mixture using fluorobenzene as an internal standard.

^{1}H NMR (500 MHz, $CDCl_3$, 25 °C, TMS) δ 7.68 – 7.59 (m, 2H), 7.39 – 7.32 (m, 2H), 7.18 – 7.10 (m, 1H), 4.29 (ddd, *J* = 24.3, 10.1, 2.5 Hz, 1H), 3.83 (dd, *J* = 9.7, 7.8 Hz, 1H), 3.59 (t, *J* = 9.4 Hz, 1H), 3.05 – 2.95 (m, 1H), 1.87 (hd, *J* = 6.7, 5.0 Hz, 1H), 1.51 (dd, *J* = 14.2, 5.0 Hz, 1H), 1.33 – 1.22 (m, 4H), 0.92 (d, *J* = 6.6 Hz, 3H), 0.86 (d, *J* = 6.6 Hz, 3H).

^{13}C NMR (126 MHz, $CDCl_3$, 25 °C, TMS) δ 176.3, 157.3 (dd, J_{C-F} = 290.2 Hz, 288.0 Hz), 139.1, 128.9, 124.6, 120.0, 75.6 (dd, J_{C-F} = 24.2, 18.5 Hz), 51.0 (t, J_{C-F} = 2.8 Hz), 49.9 (pm), 45.0, 39.6 (d, J_{C-F} = 4.9 Hz), 28.3, 27.9, 25.2, 23.8, 22.5.

^{19}F NMR (471 MHz, $CDCl_3$) δ -83.04 (d, *J* = 39.8 Hz), -86.89 (dd, *J* = 39.8, 24.3 Hz).

HRMS (ESI) m/z calcd for $C_{17}H_{22}F_2NO$ $[M+H]^+$ 294.1664, found 294.1668.

***trans*-4-(2,2-difluorovinyl)-3-isobutyl-3-methyl-1-phenylpyrrolidin-2-one (*trans*-3r)**

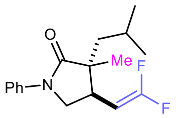

^{1}H NMR (500 MHz, $CDCl_3$, 25 °C, TMS) δ 7.61 – 7.56 (m, 2H), 7.39 – 7.32 (m, 2H), 7.18 – 7.10 (m, 1H), 4.25 (ddd, *J* = 24.6, 10.1, 2.4 Hz, 1H), 3.85 (dd, *J* = 9.5, 8.0 Hz, 1H), 3.52 (t, *J* = 9.4 Hz, 1H), 3.29 – 3.20 (m, 1H), 1.84 – 1.75 (m, 1H), 1.72 (dd, *J* = 14.3, 8.2 Hz, 1H), 1.42 (dd, *J* = 14.3, 4.5 Hz, 1H), 1.06 (s, 3H), 0.97 (d, *J* = 6.5 Hz, 3H), 0.94 (d, *J* = 6.6 Hz, 3H).

^{13}C NMR (126 MHz, $CDCl_3$, 25 °C, TMS) δ 177.3, 157.0 (dd, J_{C-F} = 290.5 Hz, 288.7 Hz), 139.3, 128.9, 124.5, 119.8, 75.6 (dd, J_{C-F} = 24.1, 18.8 Hz), 50.1 (t, J_{C-F} = 3.2 Hz), 48.1 (m), 44.3, 33.4 (d, J_{C-F} = 4.8 Hz), 24.8, 24.7, 23.0, 19.7.

^{19}F NMR (471 MHz, $CDCl_3$) δ -83.12 (d, *J* = 40.4 Hz), -86.85 (dd, *J* = 40.3, 24.6 Hz).

HRMS (ESI) m/z calcd for $C_{17}H_{22}F_2NO$ $[M+H]^+$ 294.1664, found 294.1667.

cis-3-cyclopropyl-4-(2,2-difluorovinyl)-3-methyl-1-phenylpyrrolidin-2-one (_cis_-3s)

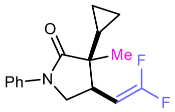

Prepared according to **Procedure B** and purified by flash column chromatograph using hexane/ethyl acetate (15:1) as eluent to give ***cis*-3s** as a white solid. Yield of **3s**: 84% (*dr* = 6:1), determined by ^{19}F NMR of the crude reaction mixture using fluorobenzene as an internal standard.

^{1}H NMR (500 MHz, $CDCl_3$, 25 ºC, TMS) δ 7.61 – 7.56 (m, 2H), 7.39 – 7.32 (m, 2H), 7.16 – 7.10 (m, 1H), 4.51 (ddd, J = 24.5, 10.1, 2.4 Hz, 1H), 3.75 (dd, J = 9.5, 8.1 Hz, 1H), 3.65 (t, J = 9.8 Hz, 1H), 3.11 – 3.02 (m, 1H), 1.18 (s, 3H), 0.84 – 0.74 (m, 1H), 0.58 – 0.49 (m, 1H), 0.50 – 0.41 (m, 1H), 0.43 – 0.37 (m, 1H), 0.26 – 0.19 (m, 1H).

^{13}C NMR (126 MHz, $CDCl_3$, 25 ºC, TMS) δ 175.3, 157.3 (dd, J_{C-F} = 290.2, 288.0 Hz), 139.2, 129.0, 124.7, 119.9, 75.5 (dd, J_{C-F} = 24.5, 18.5 Hz), 50.5 (t, J_{C-F} = 2.9 Hz), 48.0 (t, J_{C-F} = 2.0 Hz), 39.8 (d, J_{C-F} = 4.9 Hz), 20.8, 13.4, 1.5, -0.2.

^{19}F NMR (471 MHz, $CDCl_3$) δ -82.85 (d, J = 40.2 Hz), -87.61 (dd, J = 40.3, 24.5 Hz).

HRMS (ESI) m/z calcd for $C_{16}H_{18}F_2NO$ $[M+H]^+$ 278.1351, found 278.1356.

trans-3-cyclopentyl-4-(2,2-difluorovinyl)-3-methyl-1-phenylpyrrolidin-2-one (_trans_-3t)

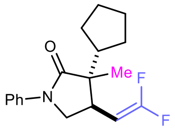

Prepared according to **Procedure B** and purified by flash column chromatograph using hexane/ethyl acetate (15:1) as eluent to give ***cis*-3t** and ***trans*-3t** as colorless oil, respectively. Yield of **3t**: 82% (*dr* = 0.8:1), determined by ^{19}F NMR of the crude reaction mixture using fluorobenzene as an internal standard.

^{1}H NMR (500 MHz, $CDCl_3$, 25 ºC, TMS) δ 7.59 (d, J = 8.1 Hz, 2H), 7.39 – 7.31 (m, 2H), 7.14 (t, J = 7.4 Hz, 1H), 4.29 (ddd, J = 24.2, 10.4, 2.4 Hz, 1H), 3.87 – 3.79 (m, 1H), 3.47 (t, J = 9.3 Hz, 1H), 3.25 – 3.14 (m, 1H), 2.20 (p, J = 8.5 Hz, 1H), 1.82 – 1.52 (m, 7H), 1.38 – 1.29 (m, 1H), 1.15 (s, 3H).

^{13}C NMR (126 MHz, $CDCl_3$, 25 ºC, TMS) δ 177.2, 156.7 (dd, J_{C-F} = 289.7 Hz, 287.5 Hz), 139.2, 128.8, 124.6, 119.9, 77.0 (dd, J_{C-F} = 23.6 Hz, 18.2 Hz), 50.5 (t, J_{C-F} = 3.1 Hz), 49.8 (m), 46.4, 33.3 (d, J_{C-F} = 4.7 Hz), 27.2 (d, J_{C-F} = 3.6 Hz), 25.5, 17.6.

^{19}F NMR (471 MHz, $CDCl_3$) δ -83.75 (d, J = 40.6 Hz), -87.33 (dd, J = 40.6, 24.2 Hz).

HRMS (ESI) m/z calcd for $C_{18}H_{22}F_2NO$ $[M+H]^+$ 306.1664, found 306.1666.

cis-3-cyclopentyl-4-(2,2-difluorovinyl)-3-methyl-1-phenylpyrrolidin-2-one (_cis_-3t)

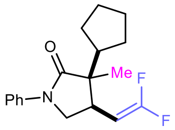

^{1}H NMR (500 MHz, $CDCl_3$, 25 ºC, TMS) δ 7.65 – 7.58 (m, 2H), 7.39 – 7.32 (m, 2H), 7.16 – 7.11 (m, 1H), 4.35 (ddd, J = 24.0, 10.3, 2.5 Hz, 1H), 3.78 (dd, J = 9.6, 8.5 Hz, 1H), 3.55 (t, J = 9.7 Hz, 1H), 3.12 – 3.02 (m, 1H), 1.97 – 1.87 (m, 1H), 1.79 – 1.67 (m, 3H), 1.62 – 1.43 (m, 4H), 1.29 (s, 3H), 1.25 – 1.16 (m, 1H).

^{13}C NMR (126 MHz, $CDCl_3$, 25 ºC, TMS) δ 176.3, 157.3 (dd, J_{C-F} = 290.2 Hz, 288.0 Hz), 139.1, 128.9, 124.6, 120.0, 75.6 (dd, J_{C-F} = 24.2, 18.5 Hz), 51.0 (t, J_{C-F} = 2.8 Hz), 49.9 (pm), 45.0, 39.6 (d, J_{C-F} = 4.9 Hz), 28.3, 27.9, 25.2, 23.8, 22.5.

^{19}F NMR (471 MHz, $CDCl_3$) δ -82.94 (d, J = 39.8 Hz), -87.31 (dd, J = 39.7, 24.0 Hz).

HRMS (ESI) m/z calcd for $C_{18}H_{22}F_2NO$ $[M+H]^+$ 306.1664, found 306.1671.

cis-1-(4-(_tert_-butyl)phenyl)-4-(2,2-difluorovinyl)-3-methyl-3-phenylpyrrolidin-2-one (_cis_-3u)

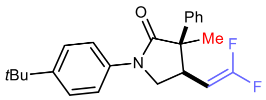

Prepared according to **Procedure B** and purified by flash column chromatograph using hexane/ethyl acetate (15:1) as eluent to give ***cis*-3u** as a white solid. Yield of **3u**: 92% (*dr* = 8:1), determined by ^{19}F NMR of the crude reaction mixture using fluorobenzene as an internal standard.

^{1}H NMR (500 MHz, $CDCl_3$, 25 ºC, TMS) δ 7.68 – 7.62 (m, 2H), 7.48 – 7.41 (m, 2H), 7.35 – 7.27 (m, 2H), 7.28 – 7.21 (m, 1H), 7.16 – 7.07 (m, 2H), 3.81 (dd, J = 9.7, 7.7 Hz, 1H), 3.61 (ddd, J = 24.5, 10.4, 2.2 Hz, 1H), 3.46 (t, J = 9.9 Hz, 1H), 3.26 – 3.16 (m, 1H), 1.68 (s, 3H), 1.34 (s, 9H).

^{13}C NMR (126 MHz, $CDCl_3$, 25 ºC, TMS) δ 176.2, 157.0 (dd, J_{C-F} = 289.8, 288.4 Hz) 148.0, 138.5, 136.3, 128.7, 127.4, 127.0, 125.9, 119.8, 75.8 (dd, J_{C-F} = 25.1, 17.4 Hz), 53.5 (m), 50.3 (t, J_{C-F} = 2.8 Hz), 40.0 (d, J_{C-F} = 5.0 Hz), 34.5, 31.4, 22.9.

^{19}F NMR (471 MHz, $CDCl_3$) δ -82.51 (d, J = 39.3 Hz), -88.02 (dd, J = 39.3, 24.5 Hz).

HRMS (ESI) m/z calcd for $C_{23}H_{26}F_2NO$ $[M+H]^+$ 370.1977, found 370.1981.

***cis*-1-benzyl-4-(2,2-difluorovinyl)-3-methyl-3-phenylpyrrolidin-2-one (*cis*-3v)**

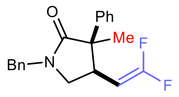

Prepared according to **Procedure A** and purified by flash column chromatograph using hexane/ethyl acetate (10:1) as eluent to give ***cis*-3v** as a white solid. Yield of **3v**: 87% (*dr* = 2.6:1), determined by ^{19}F NMR of the crude reaction mixture using fluorobenzene as an internal standard.

^{1}H NMR (500 MHz, $CDCl_3$, 25 ºC, TMS) δ 7.42 – 7.30 (m, 5H), 7.29 – 7.23 (m, 2H), 7.25 – 7.18 (m, 1H), 7.06 – 6.99 (m, 2H), 4.68 – 4.57 (m, 2H), 3.45 (ddd, J = 24.6, 10.4, 2.3 Hz, 1H), 3.29 (dd, J = 9.8, 7.8 Hz, 1H), 3.09 – 2.99 (m, 1H), 2.90 (t, J = 9.6 Hz, 1H), 1.61 (s, 3H).

^{13}C NMR (126 MHz, $CDCl_3$, 25 ºC, TMS) δ 177.1, 156.7 (dd, J_{C-F} = 289.9, 288.1 Hz), 138.8, 136.2, 128.9, 128.7, 128.5, 127.9, 127.2, 126.8, 76.2 (dd, J_{C-F} = 24.9, 17.7 Hz), 52.1 (t, J_{C-F} = 1.9 Hz), 48.8 (t, J_{C-F} = 2.9 Hz), 47.0, 40.6 (d, J_{C-F} = 5.1 Hz), 22.9.

^{19}F NMR (471 MHz, $CDCl_3$) δ -83.31 (d, J = 40.4 Hz), -88.60 (dd, J = 40.4, 24.6 Hz).

HRMS (ESI) m/z calcd for $C_{20}H_{20}F_2NO$ $[M+H]^+$ 328.1507, found 328.1525.

***cis*-4-(2,2-difluorovinyl)-3-methyl-3-phenyl-1-(3-phenylpropyl)pyrrolidin-2-one (*cis*-3w)**

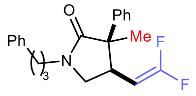

Prepared according to **Procedure A** and purified by flash column chromatograph using hexane/ethyl acetate (10:1) as eluent to give ***cis*-3w** as a colorless oil. Yield of **3w**: 98% (*dr* = 2.5:1), determined by ^{19}F NMR of the crude reaction mixture using fluorobenzene as an internal standard.

^{1}H NMR (500 MHz, $CDCl_3$, 25 ºC, TMS) δ 7.35 – 7.28 (m, 4H), 7.27 – 7.17 (m, 4H), 7.11 – 7.04 (m, 2H), 3.57 – 3.45 (m, 3H), 3.39 – 3.29 (m, 1H), 3.05 – 2.94 (m, 2H), 2.80 – 2.64 (m, 2H), 2.03 – 1.94 (m, 2H), 1.57 (s, 3H).

^{13}C NMR (126 MHz, $CDCl_3$, 25 ºC, TMS) δ 177.0, 156.8 (dd, J_{C-F} = 290.0, 288.3 Hz), 141.3, 138.7, 128.6, 128.5, 128.4, 127.2, 126.8, 126.1, 76.2 (dd, J_{C-F} = 24.8, 17.7 Hz), 52.3 (m), 49.4 (t, J_{C-F} = 2.9 Hz), 42.7, 40.7 (d, J_{C-F} = 5.0 Hz), 33.5, 29.1, 22.9.

^{19}F NMR (471 MHz, $CDCl_3$) δ -83.22 (d, J = 40.4 Hz), -88.60 (dd, J = 40.4, 24.5 Hz).

HRMS (ESI) m/z calcd for $C_{22}H_{24}F_2NO$ $[M+H]^+$ 356.1820, found 356.1825.

***cis*-4-(2,2-difluorovinyl)-1-hexyl-3-methyl-3-phenylpyrrolidin-2-one (*cis*-3x)**

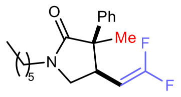

Prepared according to **Procedure A** and purified by flash column chromatograph using hexane/ethyl acetate (10:1) as eluent to give ***cis*-3x** as a colorless oil. Yield of **3x**: 85% (*dr* = 2.5:1), determined by ^{19}F NMR of the crude reaction mixture using fluorobenzene as an internal standard.

^{1}H NMR (500 MHz, $CDCl_3$, 25 ºC, TMS) δ 7.35 – 7.28 (m, 2H), 7.26 – 7.21 (m, 1H), 7.12 – 7.05 (m, 2H), 3.57 – 3.45 (m, 2H), 3.44 – 3.35 (m, 2H), 3.11 – 2.98 (m, 2H), 1.65 – 1.58 (m, 3H), 1.58 (s, 3H), 1.38 – 1.25 (m, 6H), 0.95 – 0.89 (m, 3H).

^{13}C NMR (126 MHz, $CDCl_3$, 25 ºC, TMS) δ 176.9, 156.8 (dd, $J_{C\text{-}F}$ = 290.1, 288.4 Hz), 138.8, 128.5, 127.1, 126.9, 76.3 (dd, $J_{C\text{-}F}$ = 24.7, 17.6 Hz), 52.4 (m), 49.3 (t, $J_{C\text{-}F}$ = 2.8 Hz), 42.9, 40.8 (d, $J_{C\text{-}F}$ = 5.1 Hz), 31.5, 27.5, 26.7, 23.0, 22.6, 14.0.

^{19}F NMR (471 MHz, $CDCl_3$) δ -83.33 (d, J = 40.8 Hz), -88.75 (dd, J = 40.8, 24.6 Hz).

HRMS (ESI) m/z calcd for $C_{19}H_{26}F_2NO$ $[M+H]^+$ 322.1977, found 322.1979.

***cis*-4-(2,2-difluorovinyl)-3-methyl-1-neopentyl-3-phenylpyrrolidin-2-one (*cis*-3y)**

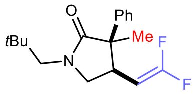

Prepared according to **Procedure A** and purified by flash column chromatograph using hexane/ethyl acetate (10:1) as eluent to give ***cis*-3y** and ***trans*-3y** as white solids, respectively. Yield of **3y**: 88% (*dr* = 1.5:1), determined by ^{19}F NMR of the crude reaction mixture using fluorobenzene as an internal standard.

^{1}H NMR (500 MHz, $CDCl_3$, 25 ºC, TMS) δ 7.35 – 7.29 (m, 2H), 7.26 – 7.22 (m, 1H), 7.15 – 7.09 (m, 2H), 3.54 (ddd, J = 24.6, 10.1, 2.3 Hz, 1H), 3.48 (dd, J = 9.5, 7.2 Hz, 1H), 3.31 – 3.18 (m, 2H), 3.17 – 3.03 (m, 2H), 1.59 (s, 3H), 1.03 (s, 9H).

^{13}C NMR (126 MHz, $CDCl_3$, 25 ºC, TMS) δ 178.1, 155.6 (dd, $J_{C\text{-}F}$ = 289.9, 288.2 Hz), 138.8, 128.5, 127.1, 127.0, 76.3 (dd, $J_{C\text{-}F}$ = 24.6, 17.7 Hz), 55.8, 52.7 (t, $J_{C\text{-}F}$ = 2.9 Hz), 52.1 (m), 41.0 (d, $J_{C\text{-}F}$ = 5.0 Hz), 33.9, 28.5, 23.4.

^{19}F NMR (471 MHz, $CDCl_3$) δ -83.42 (d, J = 40.8 Hz), -88.68 (dd, J = 40.8, 24.6 Hz).

HRMS (ESI) m/z calcd for $C_{18}H_{24}F_2NO$ $[M+H]^+$ 308.1820, found 308.1826.

***trans*-4-(2,2-difluorovinyl)-3-methyl-1-neopentyl-3-phenylpyrrolidin-2-one (*trans*-3y)**

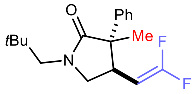

^{1}H NMR (500 MHz, $CDCl_3$, 25 ºC, TMS) δ 7.37 – 7.29 (m, 4H), 7.28 – 7.21 (m, 1H), 4.26 (ddd, J = 24.4, 9.9, 2.4 Hz, 1H), 3.57 (dd, J = 9.6, 6.9 Hz, 1H), 3.31 – 3.19 (m, 3H), 3.06 (d, J = 13.6 Hz, 1H), 1.41 (s, 3H), 0.99 (s, 9H).

^{13}C NMR (126 MHz, $CDCl_3$, 25 ºC, TMS) δ 177.5, 157.0 (dd, $J_{C\text{-}F}$ = 290.3, 288.2 Hz), 143.0, 128.5, 126.9, 126.5, 75.8 (dd, $J_{C\text{-}F}$ = 24.0, 19.0 Hz), 55.5, 52.6 (t, $J_{C\text{-}F}$ = 2.7 Hz), 51.9 (m), 40.8 (d, $J_{C\text{-}F}$ = 4.7 Hz), 33.9, 28.4, 18.4.

^{19}F NMR (471 MHz, $CDCl_3$) δ -83.68 (d, J = 39.8 Hz), -86.87 (dd, J = 39.8, 24.4 Hz).

HRMS (ESI) m/z calcd for $C_{18}H_{24}F_2NO$ $[M+H]^+$ 308.1820, found 308.1826.

2.3 General Procedure for the Synthetic Applications of *cis*-3a

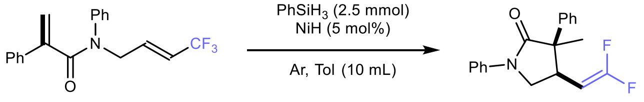

2a, 5 mmol

***cis*-3a**, 85%, 1.3 g

Gram-scale synthesis. A solution of substrate **2a** (5 mmol, 1.66 g), $PhSiH_3$ (2.5 mmol, 270 mg), and 5 mol% Ni-H (110 mg) in toluene (10 mL) was stirred in a 20 mL of vial under argon atmosphere at room temperature for 12 hours. Then the solvent was removed under reduced pressure. The residue was purified by column chromatography (7% ethyl acetate in hexane) to give the product ***cis*-3a** (85%, 1.3 g) as a white solid.

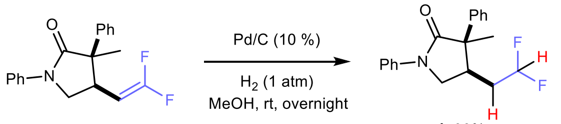

***cis*-3a**

4, 99%

A 10 mL round flask with stir bar was charged with ***cis*-3a** (0.1 mmol, 31.3 mg), Pd/C (5 mg) and MeOH (1 mL) under H_2 (1 atm). The mixture was stirred at room temperature for 12 hours. Then the solvent was removed under

reduced pressure. The residue was purified by column chromatography (7% ethyl acetate in hexane) to give the product **4** in 99% yield (31.2 mg) as a white solid.

***cis*-4-(2,2-difluoroethyl)-3-methyl-1,3-diphenylpyrrolidin-2-one (4)**

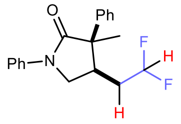

^{1}H NMR (500 MHz, $CDCl_3$, 25 ºC, TMS) δ 7.80 – 7.70 (m, 2H), 7.45 – 7.38 (m, 2H), 7.35 – 7.28 (m, 2H), 7.28 – 7.23 (m, 1H), 7.23 – 7.17 (m, 1H), 7.15 – 7.08 (m, 2H), 5.83 (tt, *J* = 56.2, 3.9 Hz, 1H), 4.02 – 3.90 (m, 1H), 3.56 (t, *J* = 10.0 Hz, 1H), 2.63 (tdd, *J* = 10.8, 7.7, 3.4 Hz, 1H), 1.95 – 1.78 (m, 1H), 1.70 (s, 3H), 1.30 – 1.20 (m, 1H).

^{13}C NMR (126 MHz, $CDCl_3$, 25 ºC, TMS) δ 176.6, 139.0, 138.7, 129.0, 128.9, 127.5, 126.6, 125.0, 120.1, 116.3 (t, $J_{C\text{-}F}$ = 240.0 Hz), 52.7, 51.2 (m), 38.5 (t, $J_{C\text{-}F}$ = 4.1 Hz), 34.0 (t, $J_{C\text{-}F}$ = 21.1 Hz), 22.7.

^{19}F NMR (471 MHz, $CDCl_3$) δ -113.00 (dddd, *J* = 282.9, 55.9, 21.8, 13.3 Hz), -115.62 (ddt, *J* = 282.9, 56.4, 19.7 Hz).

HRMS (ESI) m/z calcd for $C_{1}9H_{20}F_2NO$ $[M+H]^+$ 316.1507, found 316.1513.

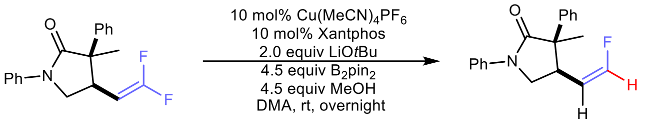

cis-3a **5**, 85%

The **5** was synthesized according to the literature with slight modification.[2] A 5 mL vial with a stir bar was charged with ***cis*-3a** (0.1 mol, 31.3 mg), $Cu(MeCN)_4PF_6$ (3.7 mg, 10 mol %), Xantphos (5.8 mg, 10 mol %), LiO*t*Bu (0.2 mmol, 16.0 mg), B_2pin_2 (0.45 mmol, 114 mg), and MeOH (0.45 mmol, 8 mg) in DMA (1 mL) under argon at room temperature. The formed mixture was stirred for 12 hours and monitored by TLC. Then the reaction was quenched by H_2O and extracted with EtOAc (5 mL × 3). The combined organic extracts were dried with Na_2SO_4, filtered, and concentrated under reduced pressure. The residue was purified by column chromatography (7% ethyl acetate in hexane) to give **5** in 85% yield (25 mg) as a colorless oil.

***cis*-(*Z*)-4-(2-fluorovinyl)-3-methyl-1,3-diphenylpyrrolidin-2-one (5)**

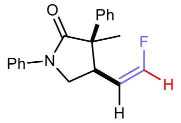

^{1}H NMR (500 MHz, $CDCl_3$, 25 ºC, TMS) δ 7.79 – 7.72 (m, 2H), 7.45 – 7.38 (m, 2H), 7.33 – 7.26 (m, 2H), 7.27 – 7.22 (m, 1H), 7.22 – 7.18 (m, 1H), 7.16 – 7.11 (m, 2H), 6.53 (ddd, *J* = 84.6, 4.8, 0.9 Hz, 1H), 4.19 (ddd, *J* = 41.4, 9.7, 4.8 Hz, 1H), 3.84 (dd, *J* = 9.4, 7.5 Hz, 1H), 3.62 – 3.55 (m, 1H), 3.53 – 3.45 (m, 1H), 1.69 (s, 3H).

^{19}F NMR (471 MHz, $CDCl_3$) δ -126.00 (dd, *J* = 84.6, 41.4 Hz).

^{13}C NMR (126 MHz, $CDCl_3$, 25 ºC, TMS) δ 176.7, 150.1 (d, $J_{C\text{-}F}$ = 262.0 Hz), 139.0, 138.8, 129.0, 128.5, 127.2, 127.0, 124.9, 120.0, 107.6 (d, $J_{C\text{-}F}$ = 2.7 Hz), 53.7 (d, $J_{C\text{-}F}$ = 1.7 Hz), 50.2 (d, $J_{C\text{-}F}$ = 2.6 Hz), 39.5 (d, $J_{C\text{-}F}$ = 4.0 Hz), 23.1.

HRMS (ESI) m/z calcd for $C_{19}H_{19}FNO$ $[M+H]^+$ 296.1445, found 296.1448.

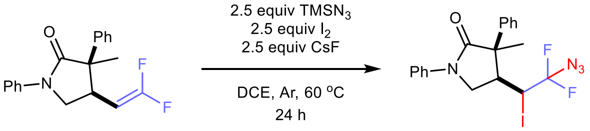

cis-3a **6**, 46%

According to the literature with slight modification,[3] trimethylsilylazide (0.25 mmol, 30 mg) was added to a solution of ***cis*-3a** (0.1 mmol, 31.3 mg), CsF (0.25 mmol, 40 mg) and I_2 (0.25 mmol, 66 mg) in anhydrous 1,2-dichloroethane (1 mL) under argon in 5 mL vial. The reaction mixture was stirred at 60 °C for 24 hours and monitored by TLC. The reaction mixture was cooled to room temperature and was quenched by H_2O (5 mL). The resulting mixture was then extracted with EtOAc (5 mL × 3). The combined organic extracts were dried with Na_2SO_4, filtered, and concentrated under reduced pressure. The residue was purified by column chromatography (10% ethyl acetate in

hexane) to give **6** in 46% yield (22 mg) as a colorless oil.

4-(2-azido-2,2-difluoro-1-iodoethyl)-3-methyl-1,3-diphenylpyrrolidin-2-one (6)

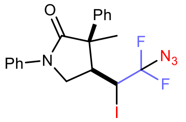

^{1}H NMR (400 MHz, $CDCl_3$, 25 °C, TMS) δ 7.78 – 7.70 (m, 2H), 7.47 – 7.31 (m, 7H), 7.25 – 7.20 (m, 1H), 4.06 (dd, J = 10.5, 7.5 Hz, 1H), 3.94 (dd, J = 10.5, 5.8 Hz, 1H), 3.86 (td, J = 9.3, 2.2 Hz, 1H), 2.43 (ddd, J = 7.7, 5.7, 2.2 Hz, 1H), 1.75 (s, 3H).

^{19}F NMR (376 MHz, $CDCl_3$) δ -70.74 (dd, J = 173.7, 8.8 Hz), -72.31 (dd, J = 173.7, 9.7 Hz).

^{13}C NMR (101 MHz, $CDCl_3$, 25 °C, TMS) δ 174.9, 138.6, 137.1, 129.0, 129.0, 127.9, 127.8, 125.2, 122.4 (t, $J_{C\text{-}F}$ = 268.9 Hz), 120.6, 53.1, 52.7, 42.3, 27.3 (t, $J_{C\text{-}F}$ = 27.4 Hz), 25.1.

HRMS (ESI) m/z calcd for $C_{19}H_{18}F_2IN_4O$ $[M+H]^+$ 483.0488, found 483.0493.

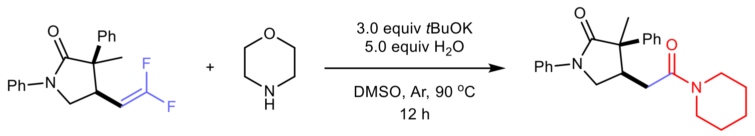

cis-**3a** morpholine **7**, 41%

According to the literature,[4] a 5 mL vial with a stir bar was charged with ***cis*-3a** (0.1 mmol, 31.3 mg), KO*t*Bu (0.3 mmol, 33.6 mg), morpholine (0.2 mmol, 17.4 mg), and H_2O (0.5 mmol, 9 mg) in DMSO (1 mL) under argon. The reaction was stirred at 90 °C for 12 h and monitored by TLC. After the completion of reaction, the reaction mixture was quenched with H_2O (5 mL) and extracted with ethyl acetate (5 mL × 3). The combined organic extracts were dried with Na_2SO_4, filtered, and concentrated under reduced pressure. The residue was purified by column chromatography (100% ethyl acetate) to give **7** in 41% yield (15.4 mg) as a white solid.

***cis*-3-methyl-4-(2-morpholino-2-oxoethyl)-1,3-diphenylpyrrolidin-2-one (7)**

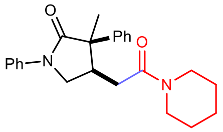

^{1}H NMR (500 MHz, $CDCl_3$, 25 °C, TMS) δ 7.80 – 7.74 (m, 2H), 7.44 – 7.37 (m, 2H), 7.36 – 7.29 (m, 2H), 7.28 – 7.23 (m, 1H), 7.22 – 7.15 (m, 3H), 4.21 (dd, J = 10.0, 7.7 Hz, 1H), 3.62 (t, J = 4.8 Hz, 2H), 3.59 – 3.49 (m, 4H), 3.45 (dd, J = 10.0, 8.7 Hz, 1H), 3.14 (t, J = 4.8 Hz, 2H), 2.99 – 2.89 (m, 1H), 2.22 (dd, J = 16.4, 4.1 Hz, 1H), 1.73 (s, 3H), 1.73 – 1.66 (m, 1H).

^{13}C NMR (126 MHz, $CDCl_3$, 25 °C, TMS) δ 176.9, 169.4, 139.8, 139.2, 128.9, 128.7, 127.2, 126.7, 124.7, 120.0, 66.8, 66.3, 52.9, 51.4, 45.6, 41.9, 40.4, 33.6, 23.2.

HRMS (ESI) m/z calcd for $C_{23}H_{27}N_2O_3$ $[M+H]^+$ 379.2016, found 379.2019.

2.4 Kinetics for the Rate Constant of H• Transfer

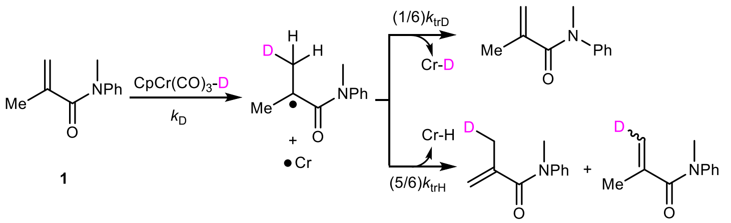

The H/D exchange of 1 (>10 equiv) and $CpCr(CO)_3D$ in toluene-d_8 in a *J-Young* tube for indicated time at 300 K. The height of the hydride of $CpCr(CO)_3H$ peak relative to the height the internal standard was recorded as a function of time. Two pulses were used for each kinetic point, with 250 seconds between the two pulses. The kinetic data were fit to an exponential, as shown below:

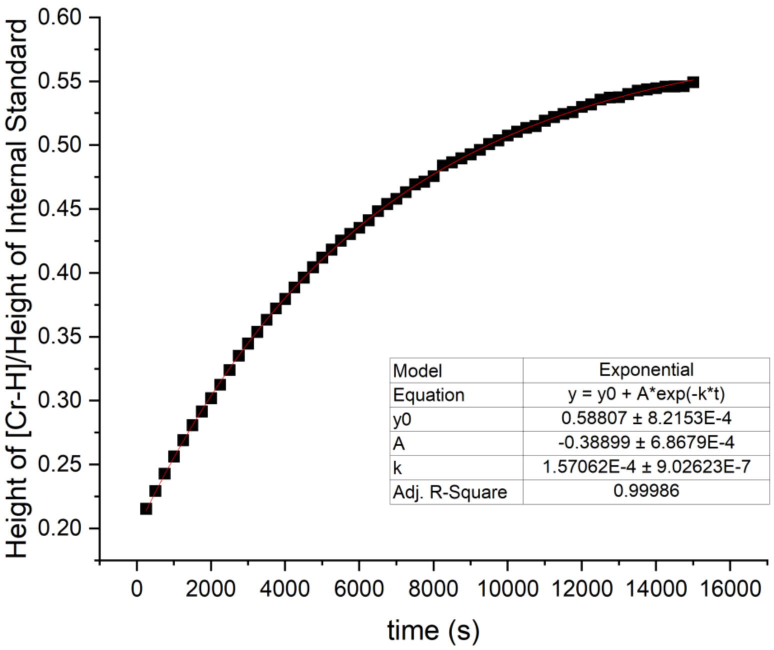

Fig. S1 | H/D exchange between 1 (0.333 M) and $CpCr(CO)_3D$ (0.033 M). (k_{obs} = 1.571 × 10^{-4} s^{-1})

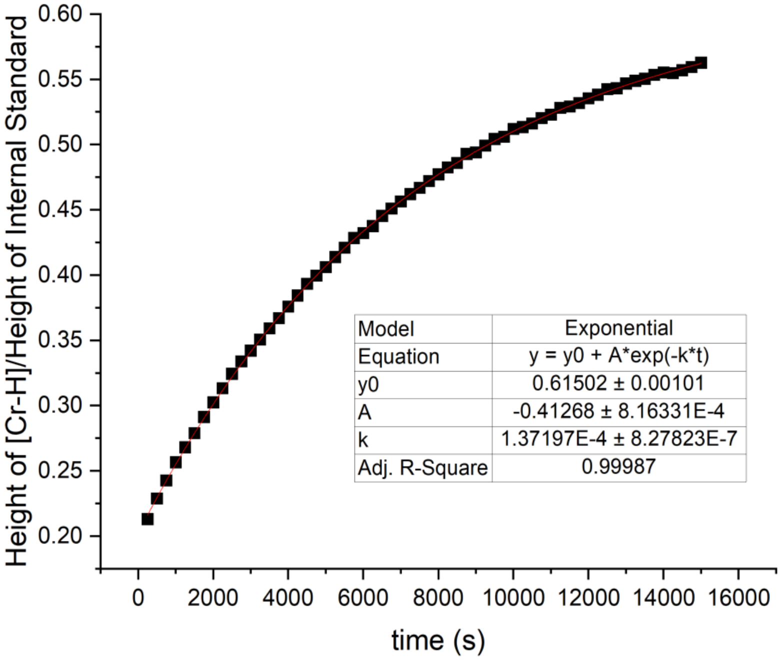

Fig. S2 | H/D exchange between 1 (0.290 M) and $CpCr(CO)_3D$ (0.029 M). (k_{obs} = 1.372 × 10^{-4} s^{-1})

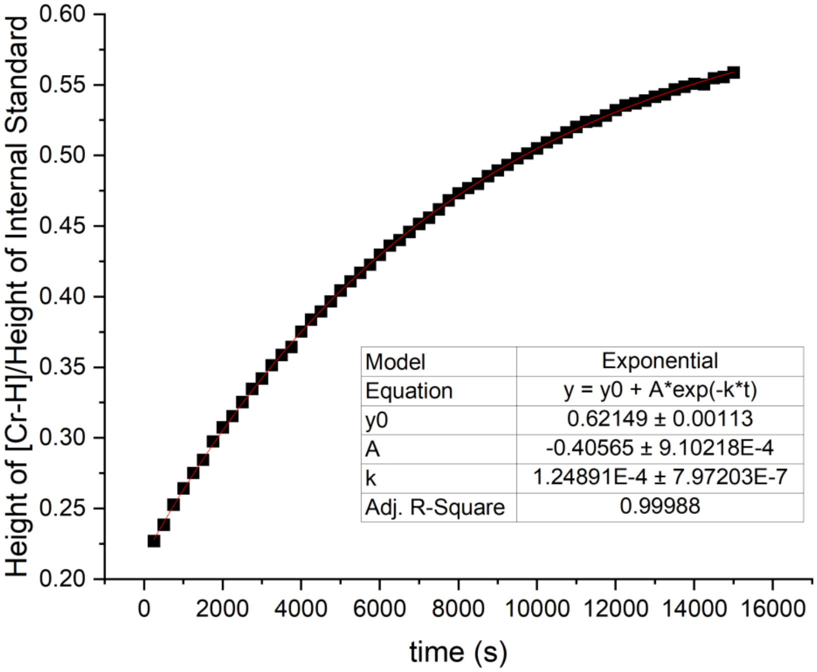

Fig. S3 | H/D exchange between 1 (0.255 M) and $CpCr(CO)_3D$ (0.025 M). ($k_{obs} = 1.249 \times 10^{-4}$ s^{-1})

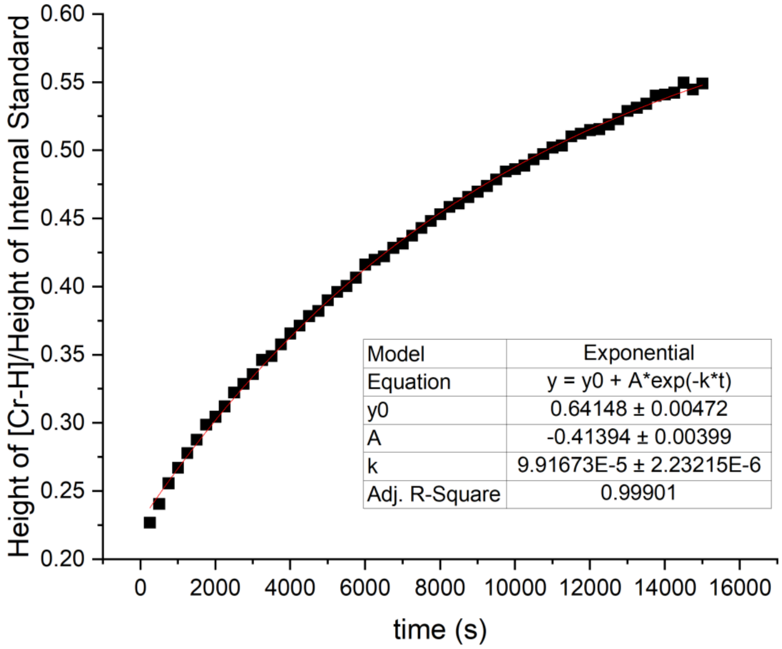

Fig. S4 | H/D exchange between 1 (0.21 M) and $CpCr(CO)_3D$ (0.020 M). ($k_{obs} = 0.992 \times 10^{-4}$ s^{-1})

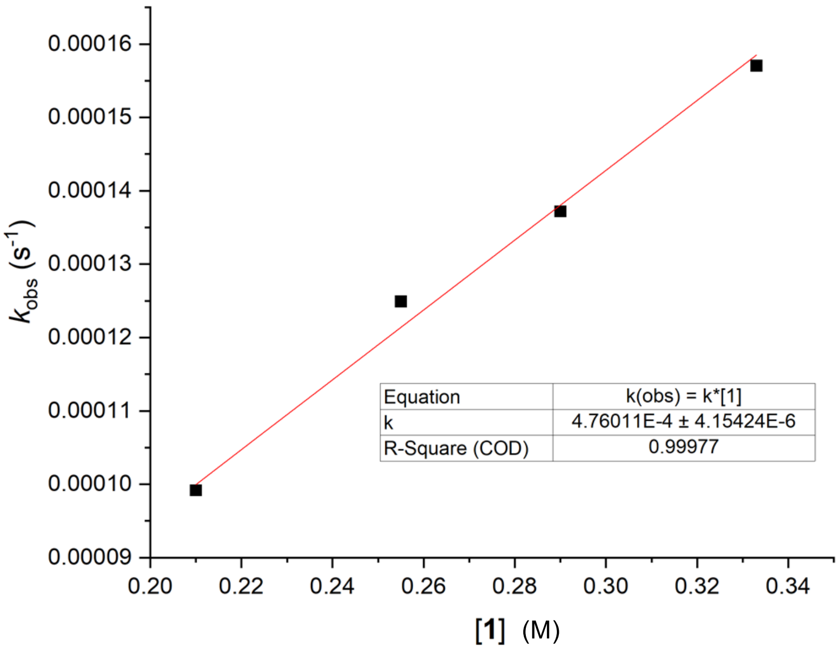

Fig. S5 | Plot of k_{obs} vs [1] for D• transfer to 1 at 300 K in toluene-d_8

By monitoring these changes over time, we obtain the pseudo-first-order rate constant k_{obs}, which is a linear function of [**1**] (Fig. S5). The slope of this line reflects both the initial D• abstraction from Cr-D and the back-transfer of H and D (rate constants k_{trH} and k_{trD}), therefore, k is 4.76 × 10^{-4} M^{-1} s^{-1} at 300 K (eq 1). Extracting k_D, the rate constant for the first D• transfer, from k requires knowledge of the fractional probability S ($k = Sk_D$) that H• will return to Cr• after the initial transfer. According to our previous research,[5] the isotope effect k_{trH}/k_{trD} can be estimated as 3, we therefore can write the fractional probability S as in eq 2, giving that k_D is 5.08 × 10^{-4} M^{-1} s^{-1} at 300 K (eq 3).

$$\frac{d[CrH]}{dt} = k[\mathbf{1}][CrD] = k_{obs}[CrD] \qquad \text{(eq 1)}$$

$$S = \frac{\left(\frac{5}{6}\right)k_{trH}[Cr\cdot]}{\left(\frac{5}{6}\right)k_{trH}[Cr\cdot]+\left(\frac{1}{6}\right)k_{trD}[Cr\cdot]} = \frac{5\frac{k_{trH}}{k_{trD}}}{5\frac{k_{trH}}{k_{trD}}+1} \approx \frac{15}{16} \qquad \text{(eq 2)}$$

$$k = Sk_D \qquad \text{(eq 3)}$$

$$k_H = k_D\frac{k_{CrH}}{k_{CrD}} \qquad \text{(eq 4)}$$

Correction by the established k_H/k_D (0.45)[6] provides that k_H is 2.28 × 10^{-4} M^{-1} s^{-1} at 300 K in toluene-d_8.

2.5 Mechanistic Experiments

2.5.1 In-situ Transformation of Nickel Species

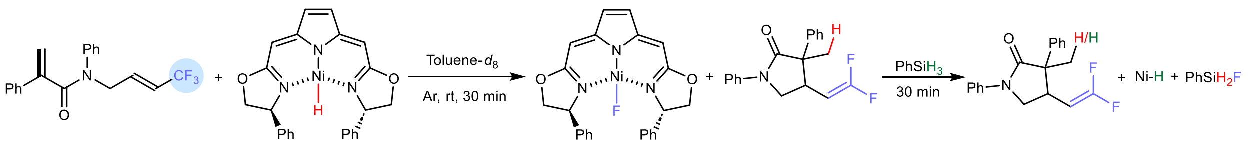

2a, 0.1 mmol — Ni-H, 0.06 mmol — **8**, 55% (^{19}F at δ −442.39) — **3a**, 53% (^{19}F at δ −82.41, -87.93) — **3a**, 90% (^{1}H at δ −24.86)

To a solution of **2a** (0.1 mol, 33.1 mg) in toluene-d_8 (0.5 mL) in a 5 mL vial was added Ni-H (0.06 mmol, 26.4 mg) under argon at room temperature. The reaction mixture was stirred for 30 mins and was transferred into a *J*-

Young tube. Then we used ^{19}F NMR to monitor the reaction, the characteristic ^{19}F NMR resonance of the Ni^{II}-F complex **8** appeared at δ -442.39. As the reaction progressed, the decrease of **2a** (a ^{19}F at δ -63.21) is accompanied by the appearance of **3a** (^{19}F at δ -83.39, -89.00), as shown below.

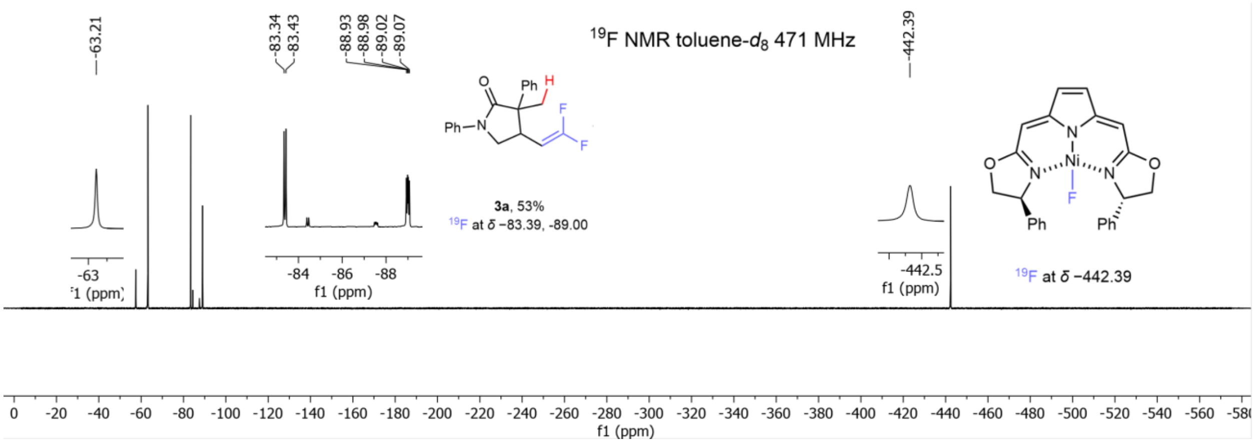

Upon adding 0.5 equiv of $PhSiH_3$, the ^{19}F peaks of **8** and the rest of **2a** disappeared, at same time, the ^{19}F signal for $PhSiH_2F$ was observed at -140.01 and the ^1H NMR signal for Ni^{II}-H reappeared at δ -24.86, as shown below.

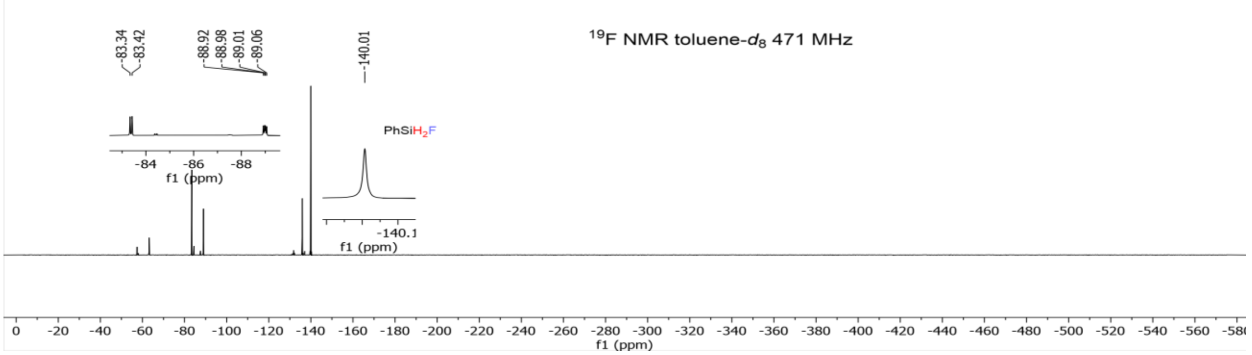

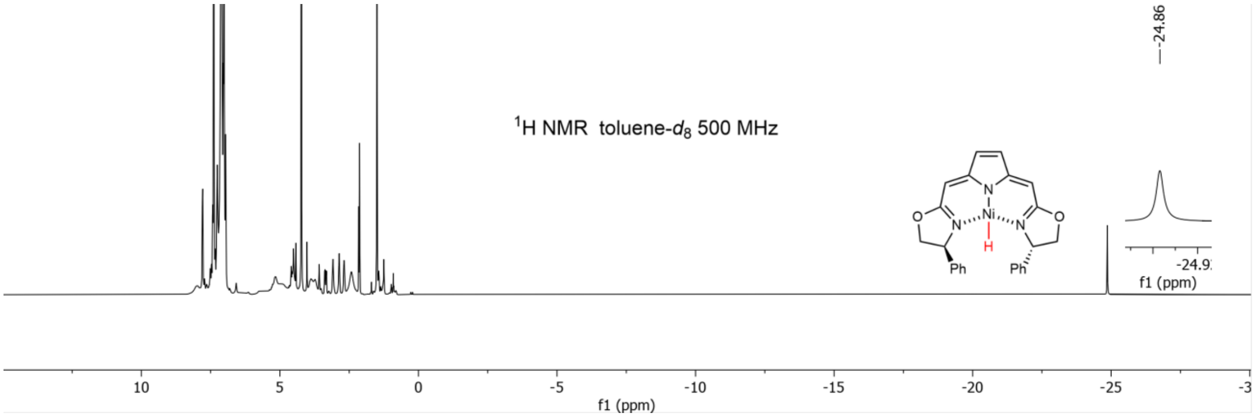

2.5.2 TEMPO Trapping Experiment

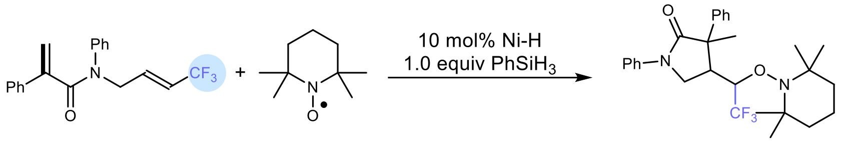

2a, 2.0 equiv **11**, 70%

A solution of substrate **2a** (0.1 mmol), $PhSiH_3$ (0.1 mmol, 10.8 mg), TEMPO (0.2 mmol, 31.2 mg), and 10 mol% Ni-H (4.4 mg) in toluene (0.5 mL) was stirred in a 5 mL of vial under argon atmosphere at room temperature for 3 hours. The reaction was monitored by TLC until the full conversion of the substrate **2a**. Then the solvent was removed under reduced pressure. The residue was purified by column chromatography (7% ethyl acetate in hexane) to give the product **11** (70%, 34.2 mg) as a white solid.

3-methyl-1,3-diphenyl-4-(2,2,2-trifluoro-1-((2,2,6,6-tetramethylpiperidin-1-yl)oxy)ethyl)pyrrolidin-2-one (11)

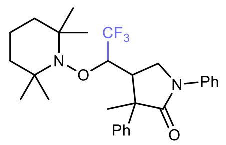

^{1}H NMR (500 MHz, $CDCl_3$, 25 °C, TMS) δ 7.77 – 7.73 (m, 2H), 7.46 – 7.39 (m, 4H), 7.38 – 7.31 (m, 2H), 7.25 – 7.17 (m, 2H), 4.25 (qd, J = 7.4, 5.0 Hz, 1H), 4.20 (dd, J = 10.3, 7.8 Hz, 1H), 3.99 (dd, J = 10.3, 7.9 Hz, 1H), 2.96 (td, J = 7.8, 5.1 Hz, 1H), 1.74 (s, 3H), 1.37 – 1.15 (m, 6H), 1.12 – 1.01 (m, 3H), 0.97 – 0.78 (m, 6H), 0.37 (s, 3H).

^{13}C NMR (126 MHz, $CDCl_3$, 25 °C, TMS) δ 175.9, 139.3, 139.1, 129.0, 129.0, 127.5, 125.08 (q, $J_{C\text{-}F}$ = 287.9 Hz), 124.8, 120.1, 76.5 (q, $J_{C\text{-}F}$ = 27.4 Hz), 61.2 – 60.6 (m), 52.4, 46.5, 45.1, 41.1 – 40.6 (m), 33.3 – 32.5 (m), 29.7, 25.7, 20.5, 16.9.

^{19}F NMR (471 MHz, $CDCl_3$) δ -68.04 (d, J = 7.4 Hz).

HRMS (ESI) m/z calcd for $C_{28}H_{36}F_3N_2O_2$ $[M+H]^+$ 489.2723, found 489.2729.

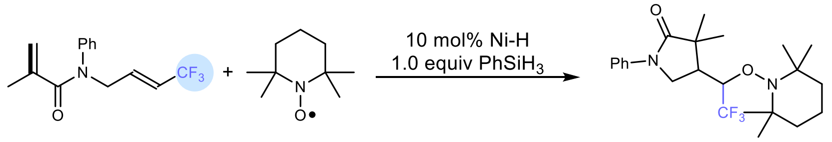

2o 2.0 equiv **12**, detected by HRMS

A solution of substrate **2o** (0.1 mmol), $PhSiH_3$ (0.1 mmol, 10.8 mg), TEMPO (0.2 mmol, 31.2 mg), and 10 mol% Ni-H (4.4 mg) in toluene (0.5 mL) was stirred in a 5 mL of vial under argon atmosphere at room temperature for 24 hours. The reaction solution was analyzed by HRMS (ESI), revealing a molecular ion at m/z 427.2551 $[M+H]^+$ (calcd. 427.2567 for $C_{23}H_{34}F_3N_2O_2^+$). The result demonstrated the formation of TEMPO-adduct **12**.

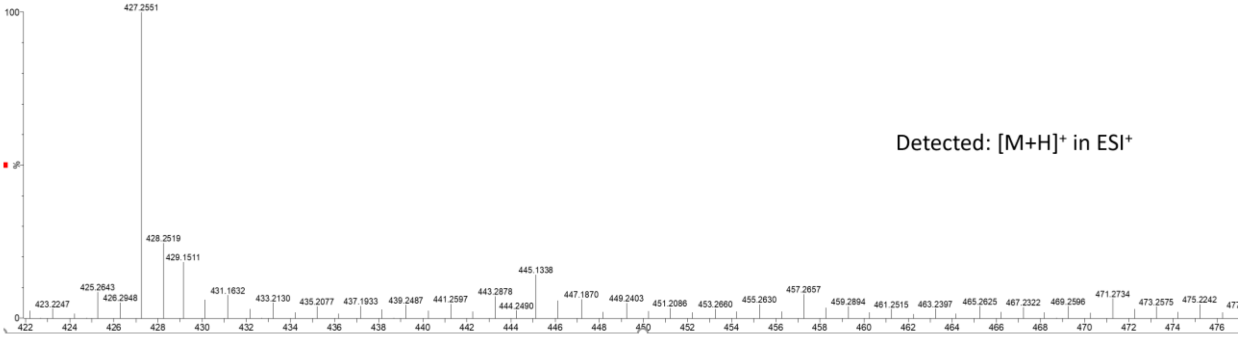

2.5.3 Hydrogenation of Substrate 13

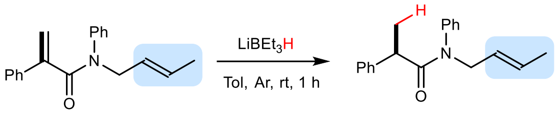

13 **14**, 86%

To a solution of substrate **13** (0.1 mmol, 27.7 mg) in toluene (1 mL) in a 5 mL of vial was added $LiBEt_3H$ (1.2 equiv, 1 N in THF) under argon atmosphere at room temperature. The reaction mixture was stirred for 1 hour and monitored by TLC until the full conversion of substrate **13**. Then the reaction was quenched by H_2O (5 mL, *Caution: Gas release*) and extracted with EtOAc (5 mL × 3). The combined organic extracts were dried with Na_2SO_4, filtered, and concentrated under reduced pressure. The residue was purified by column chromatography (7% ethyl acetate in hexane) to give the product **14** in 86% yield (24 mg) as a colorless oil.

***E*-*N*-(but-2-en-1-yl)-*N*,2-diphenylpropanamide (14)**

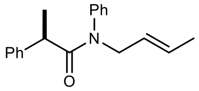

^{1}H NMR (500 MHz, $CDCl_3$, 25 °C, TMS) δ 7.33 – 7.29 (m, 3H), 7.18 (dd, J = 9.8, 6.9 Hz, 3H), 7.02 – 6.99 (m, 2H), 6.97 – 6.82 (m, 2H), 5.50 – 5.35 (m, 2H), 4.22 (dd, J = 14.3, 6.0 Hz, 1H), 4.15 (dd, J = 14.3, 6.0 Hz, 1H), 3.57 (q, J = 7.0 Hz, 1H), 1.60 (d, J = 5.7 Hz, 3H), 1.38 (d, J = 6.9 Hz, 3H).

^{13}C NMR (126 MHz, $CDCl_3$, 25 °C, TMS) δ 173.4, 142.3, 142.1, 129.3, 129.2, 128.9, 128.3, 127.8, 127.6, 126.5, 125.8, 51.8, 43.5, 20.3, 17.6.

HRMS (ESI) m/z calcd for $C_{19}H_{22}NO$ $[M+H]^+$ 280.1696, found 280.1699.

3. Reference

[1] J. Chen, J. H. Lin, J. C. Xiao, "Dehydroxylation of alcohols for nucleophilic substitution" *Chem. Commun.* **2018**, *54*, 7034-7037.

[2] J. F. Hu, X. W. Han, Y. Yuan, Z. Z. Shi, "Stereoselective Synthesis of Z Fluoroalkenes through Copper-Catalyzed Hydrodefluorination of *gem*-Difluoroalkenes with Water" *Angew. Chem. Int. Ed.* **2017**, *56*, 13342-13346.

[3] R. Cheng, Y. Q. Sang, X. Gao, S. Zhang, X. S. Xue, X. G. Zhang, "Highly γ-Selective Arylation and Carbonylative Arylation of 3-Bromo-3,3-difluoropropene via Nickel Catalysis" *Angew. Chem. Int. Ed.* **2021**, *60*, 12386-12391.

[4] B. Y. Wang, X. H. Zhao, Q. Y. Liu, S. Cao, "Direct defluorinative amidation-hydrolysis reaction of *gem*-difluoroalkenes with *N*,*N*-dimethylformamide, and primary and secondary amines" *Org. Biomol. Chem.* **2018**, *16*, 8546-8552.

[5] R. M. Bullock, E. G. Samsel, "Hydrogen-Atom Transfer-Reactions of Transition-Metal Hydrides - Kinetics and Mechanism of the Hydrogenation of Alpha-Cyclopropylstyrene by Metal-Carbonyl Hydrides" *J. Am. Chem. Soc.* **1990**, *112*, 6886-6898.

[6] L. H. Tang, E. T. Papish, G. P. Abramo, J. R. Norton, M. H. Baik, R. A. Friesner, A. Rappé, "Kinetics and thermodynamics of H• transfer from $(\eta^5\text{-}C_5R_5)Cr(CO)_3H$ (R = Ph, Me, H) to methyl methacrylate and styrene" *J. Am. Chem. Soc.* **2003**, *125*, 10093-10102.

4. NMR Spectra

***N*-methyl-*N*-phenylmethacrylamide (1)**

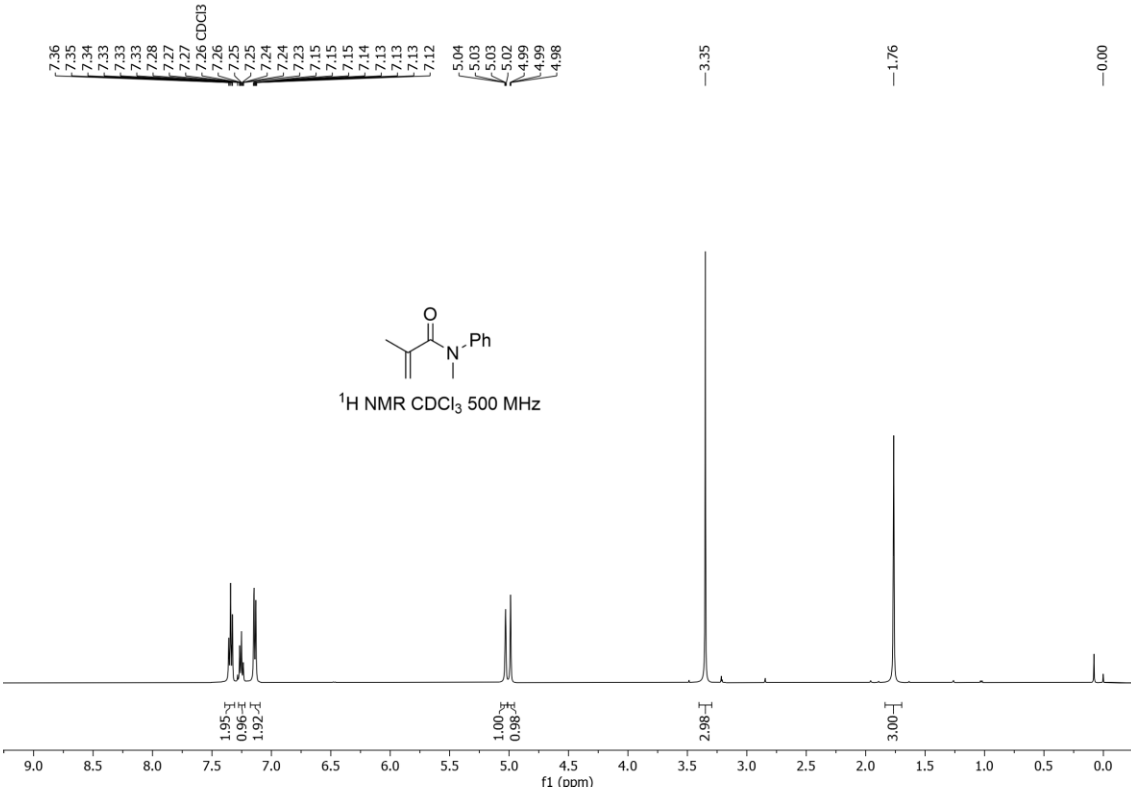

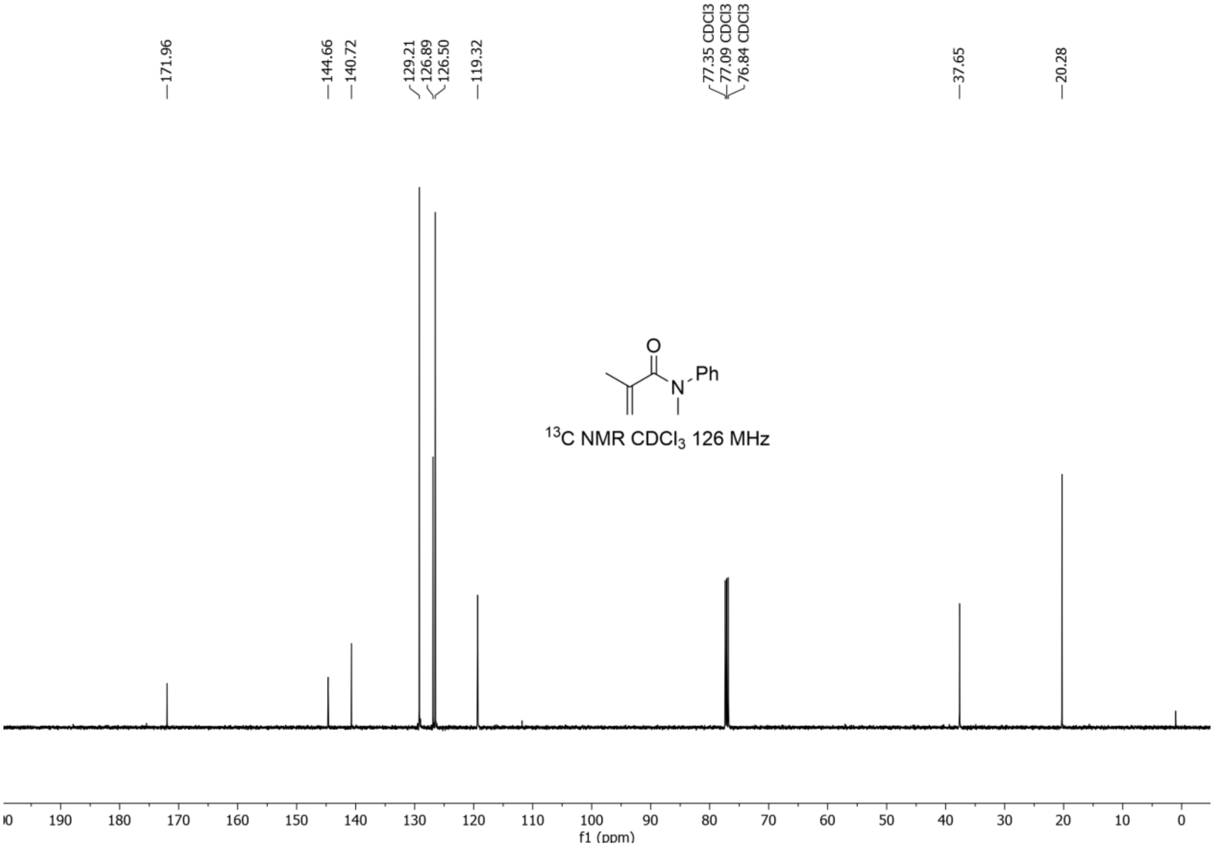

(*E*)-*N*,2-diphenyl-*N*-(4,4,4-trifluorobut-2-en-1-yl)acrylamide (2a)

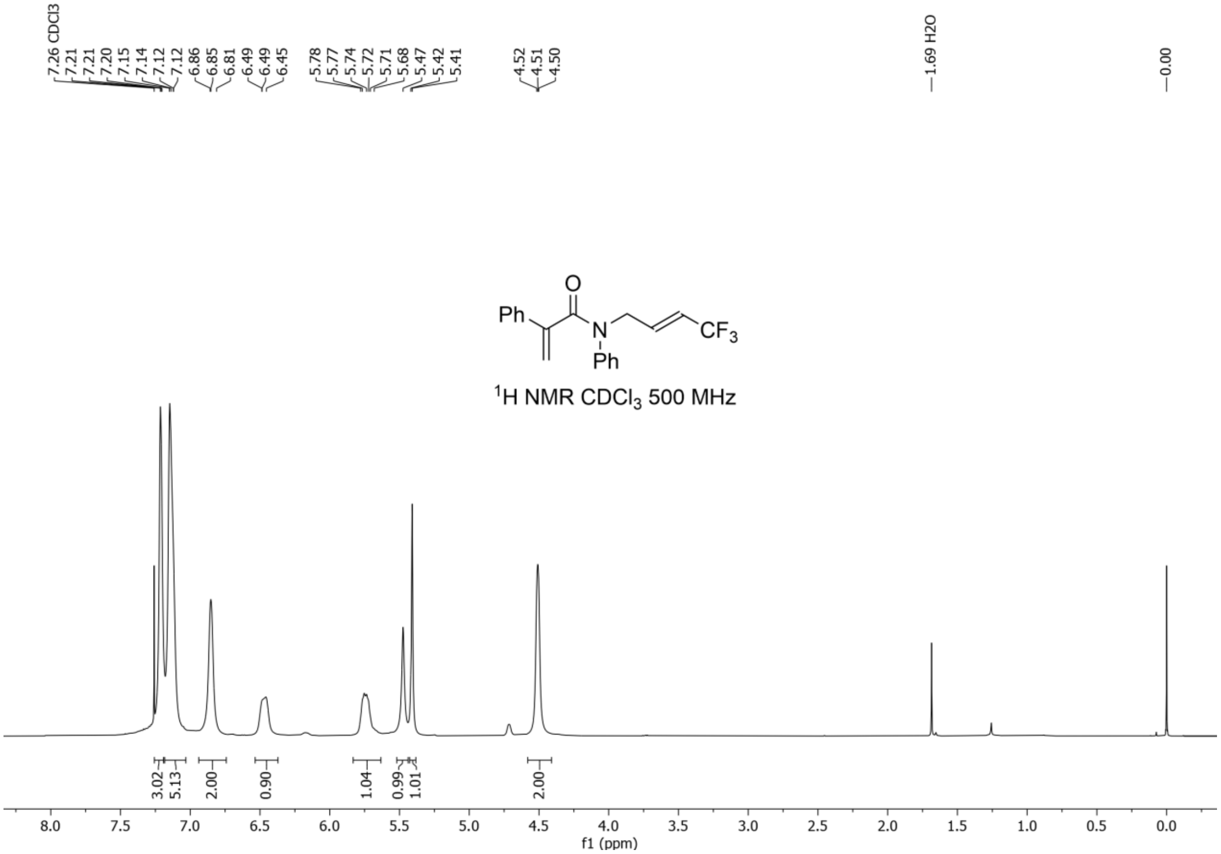

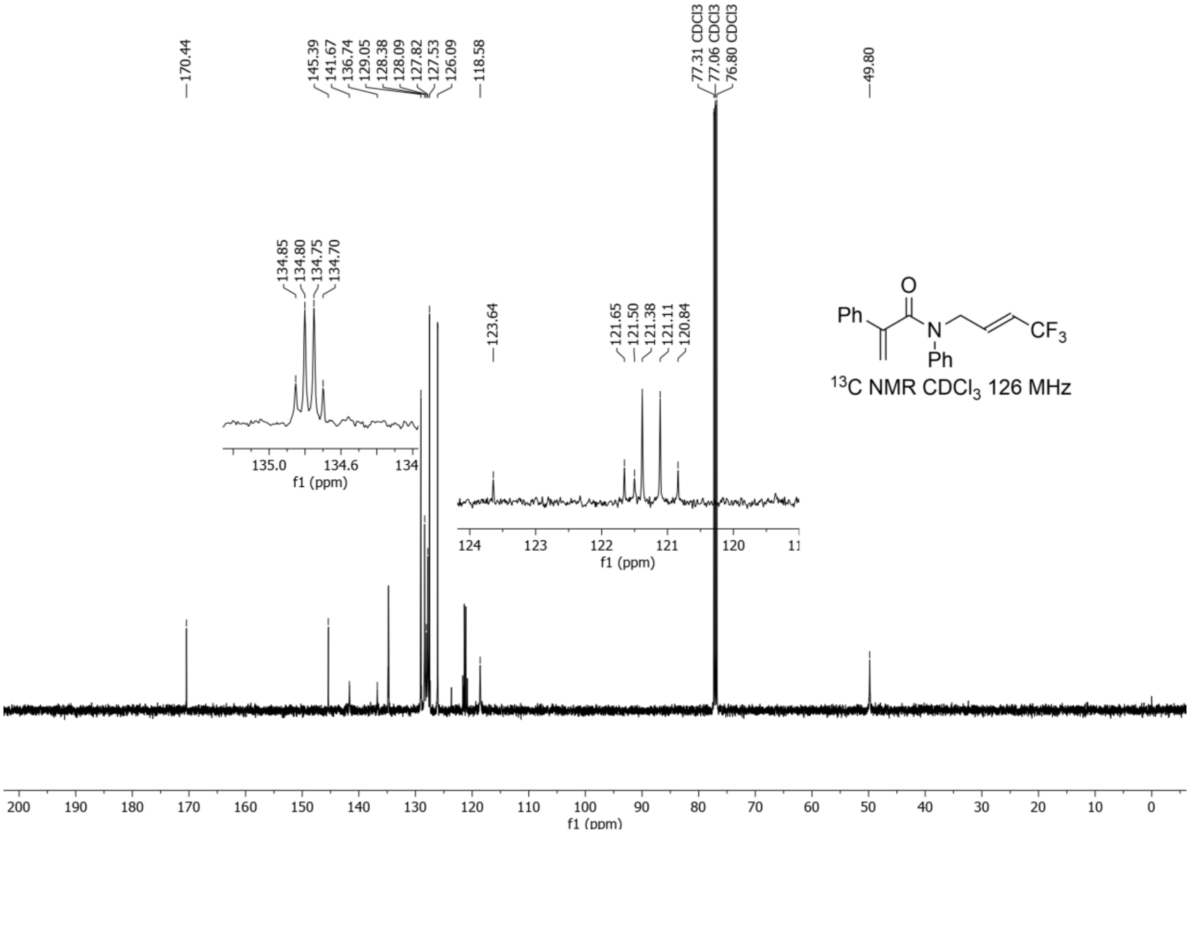

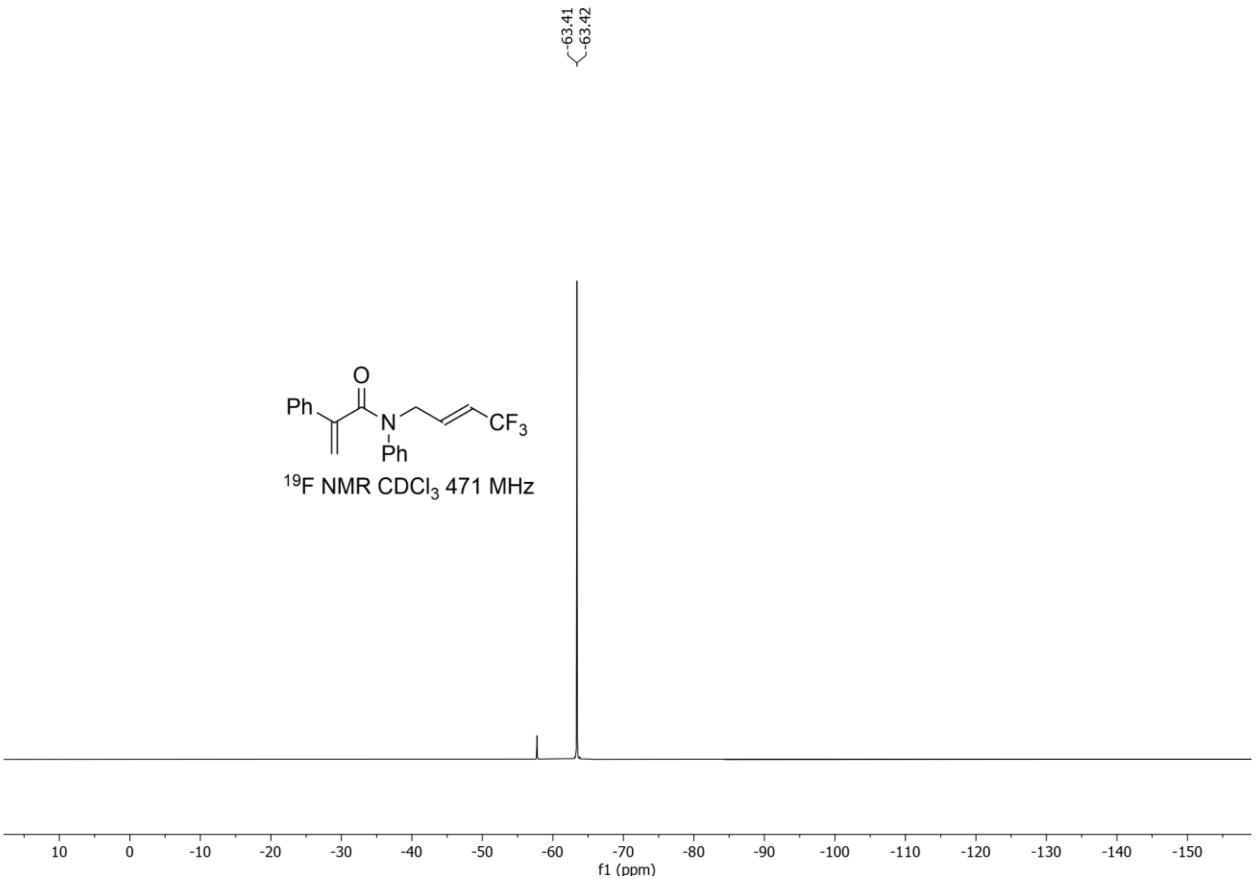

(*E*)-2-(4-methoxyphenyl)-*N*-phenyl-*N*-(4,4,4-trifluorobut-2-en-1-yl)acrylamide (2b)

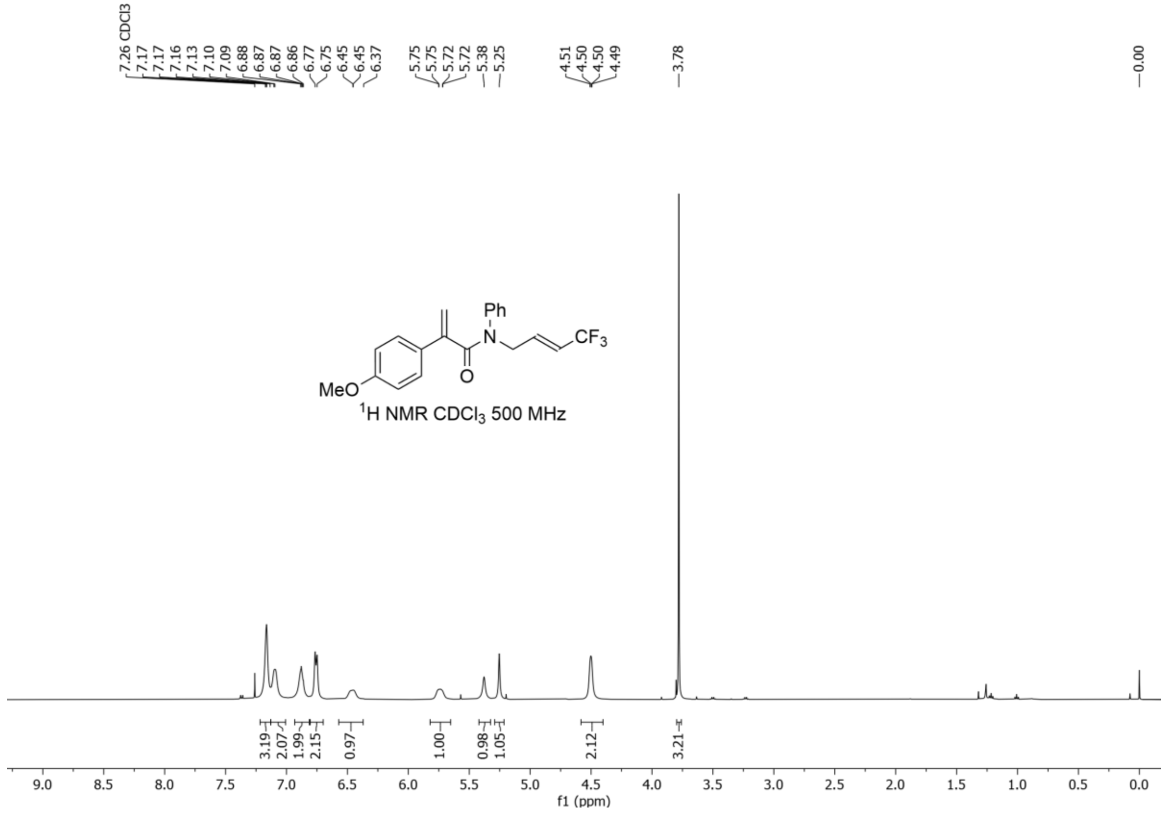

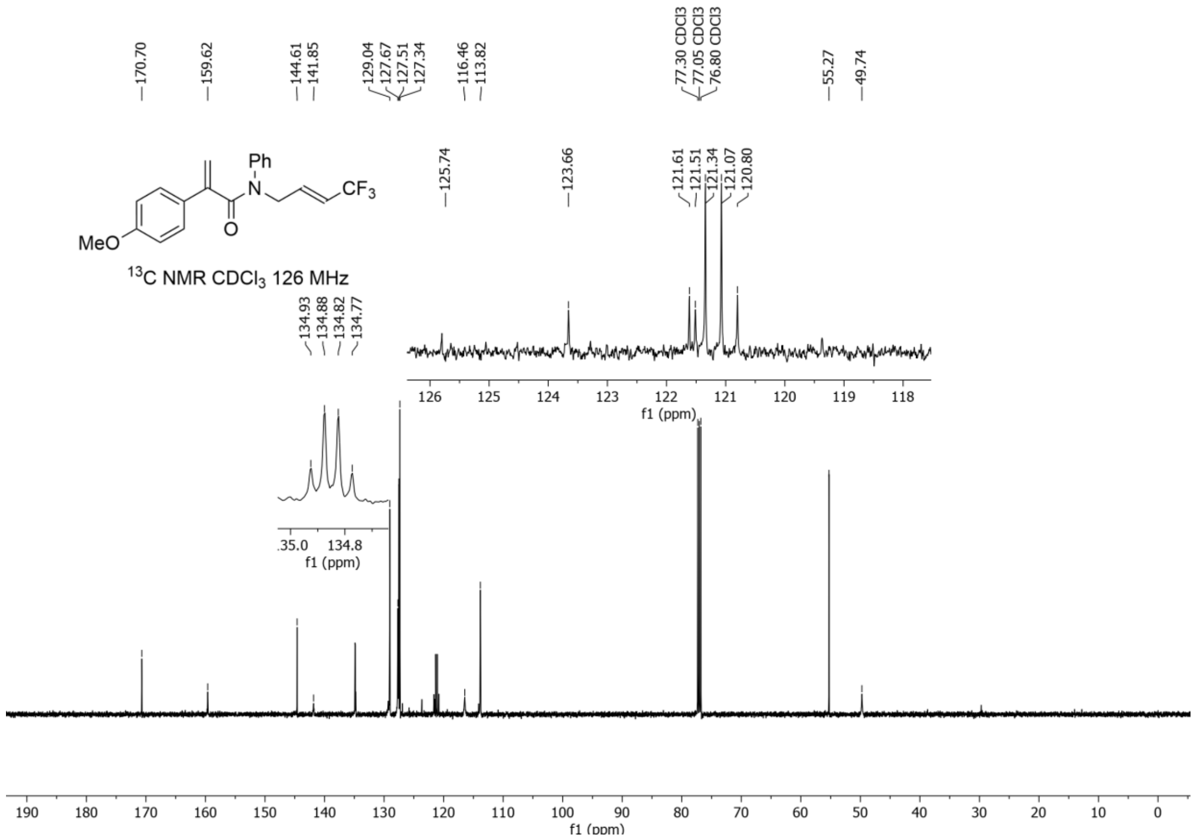

170.70
159.62
144.61
141.85
129.04
127.67
127.51
127.34
116.46
113.82
77.30 CDCl3
77.05 CDCl3
76.80 CDCl3
55.27
49.74
Ph
N
CF3
O
MeO
13C NMR CDCl3 126 MHz
125.74
123.66
121.61
121.51
121.34
121.07
120.80
134.93
134.88
134.82
134.77
f1 (ppm)
f1 (ppm)
f1 (ppm)

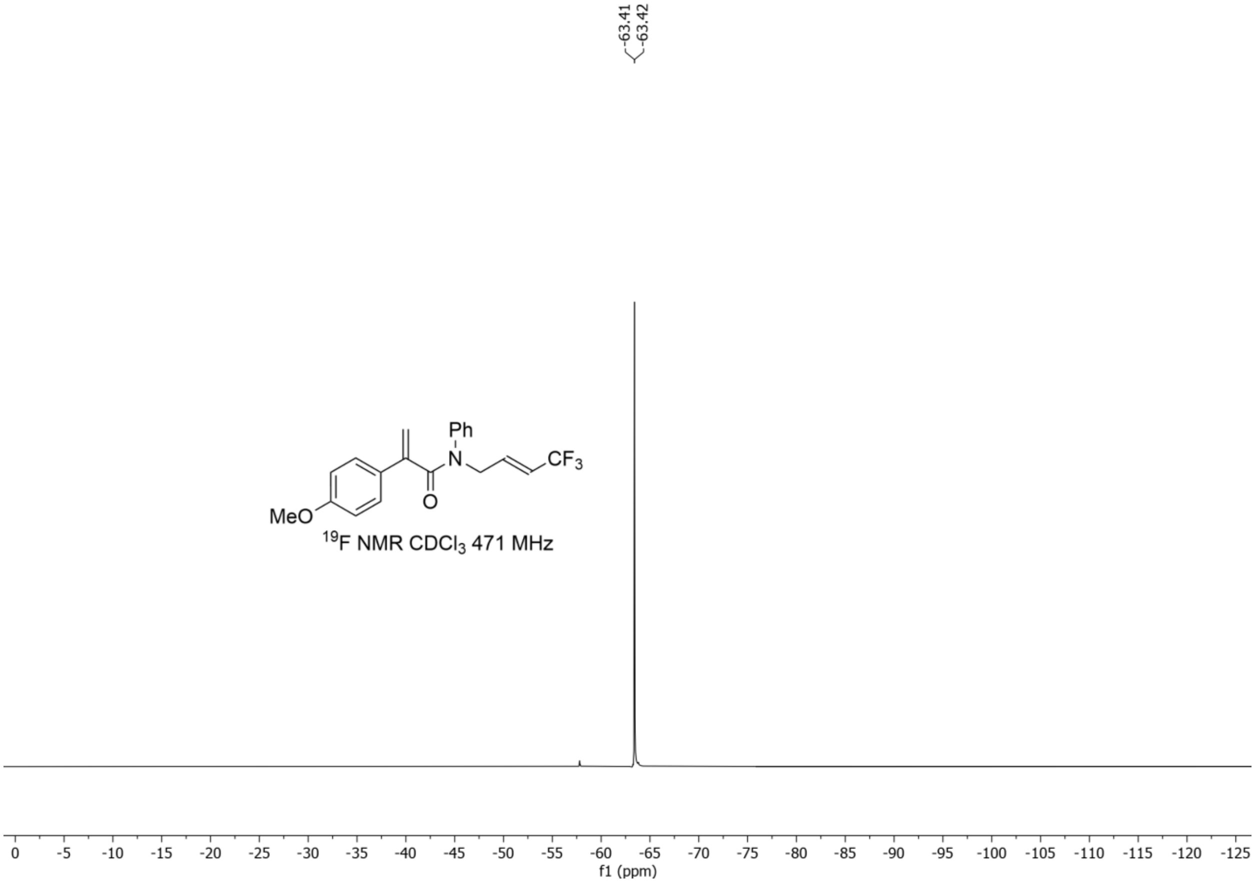

-63.41
-63.42
Ph
N
CF3
O
MeO
19F NMR CDCl3 471 MHz
f1 (ppm)

(*E*)-*N*-phenyl-2-(*p*-tolyl)-*N*-(4,4,4-trifluorobut-2-en-1-yl)acrylamide (2c)

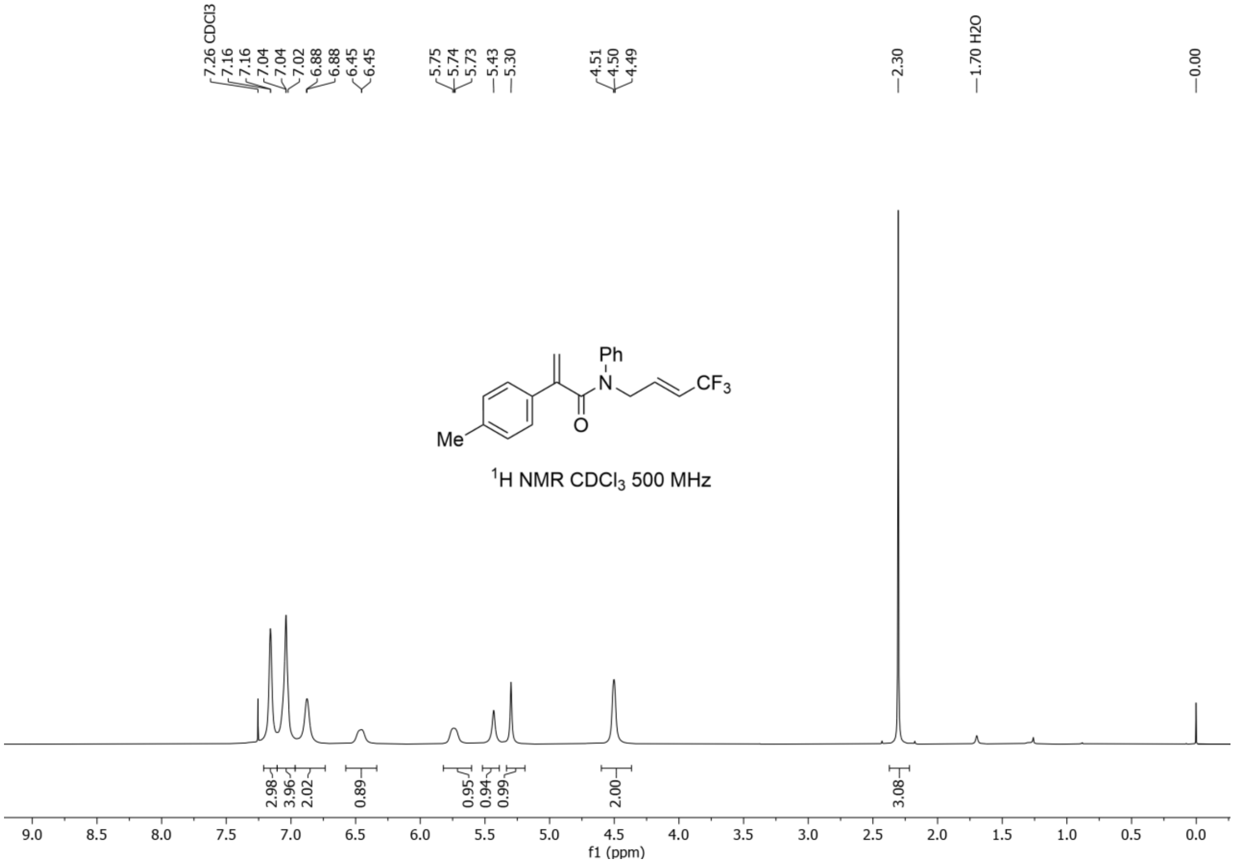

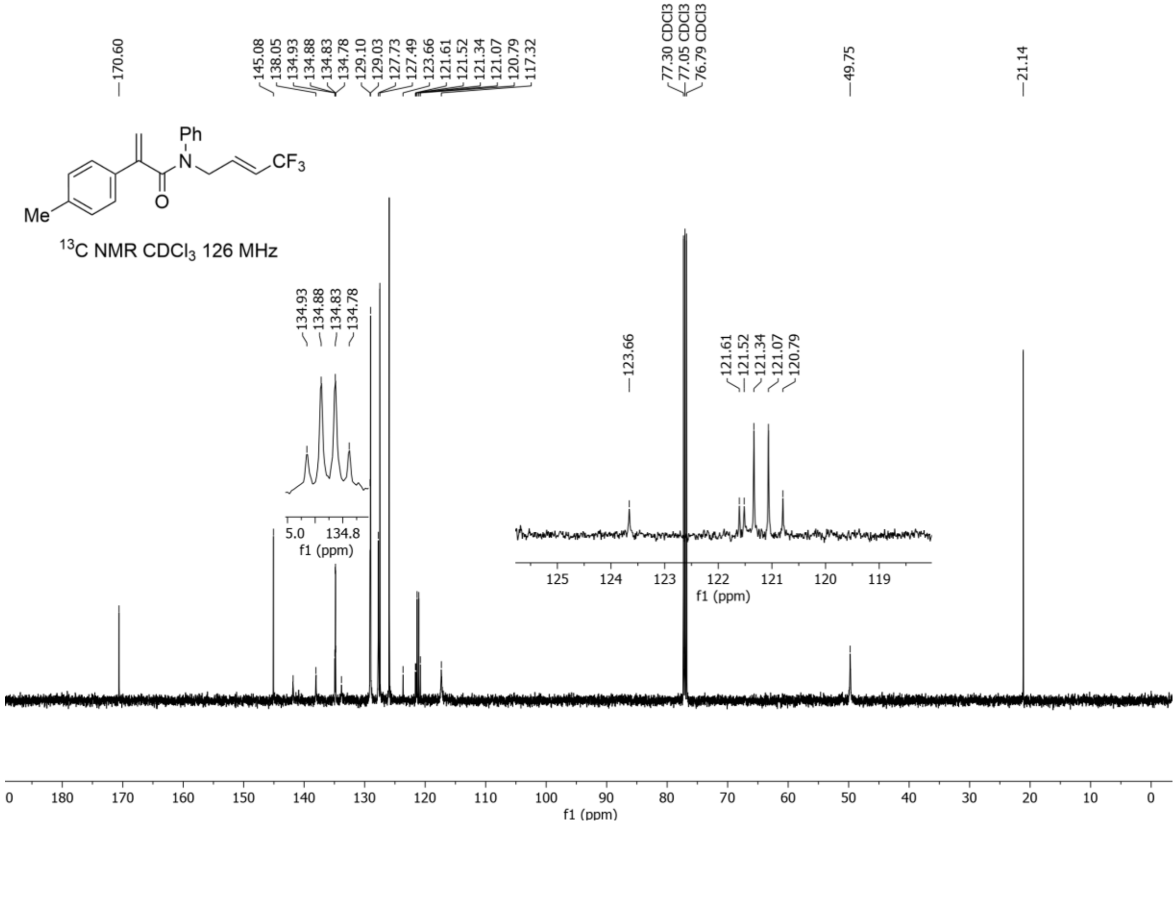

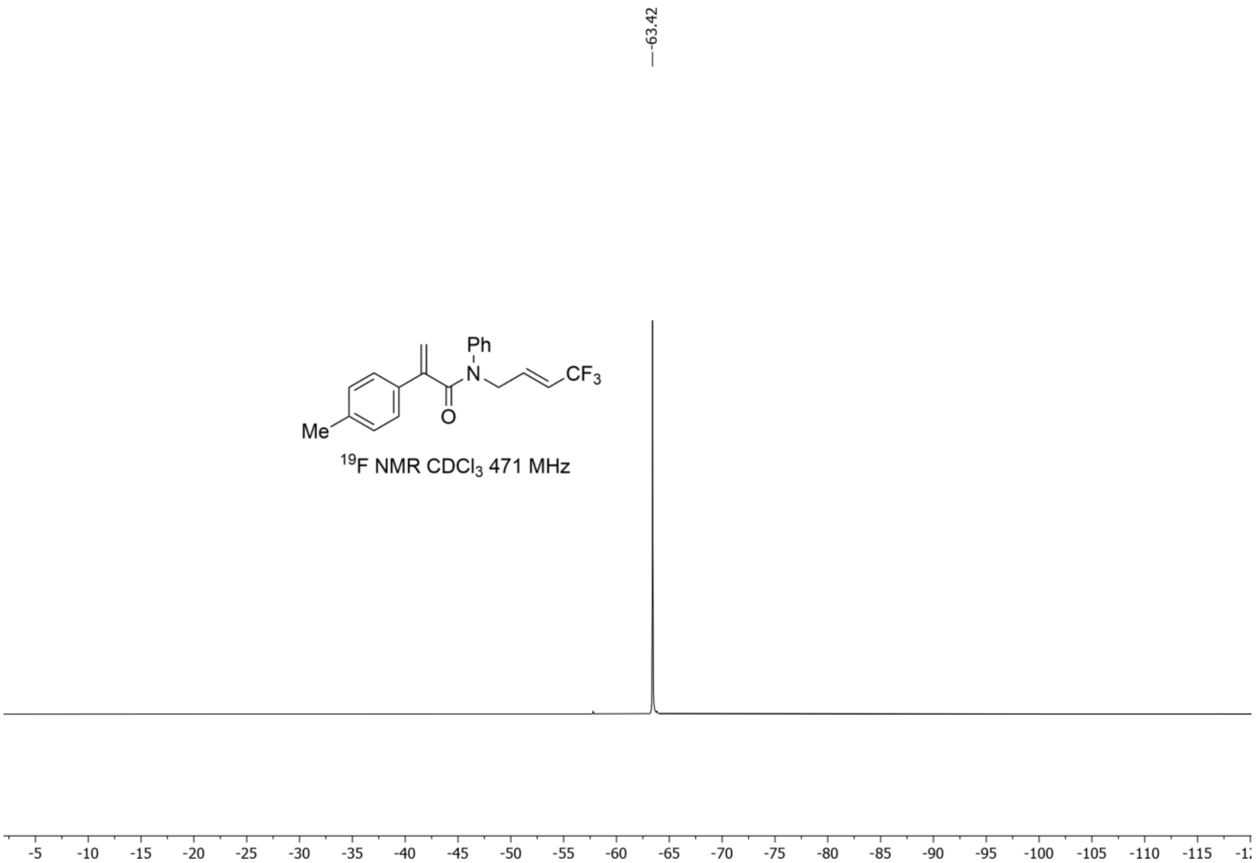

(*E*)-2-(4-(*tert*-butyl)phenyl)-*N*-phenyl-*N*-(4,4,4-trifluorobut-2-en-1-yl)acrylamide (2d)

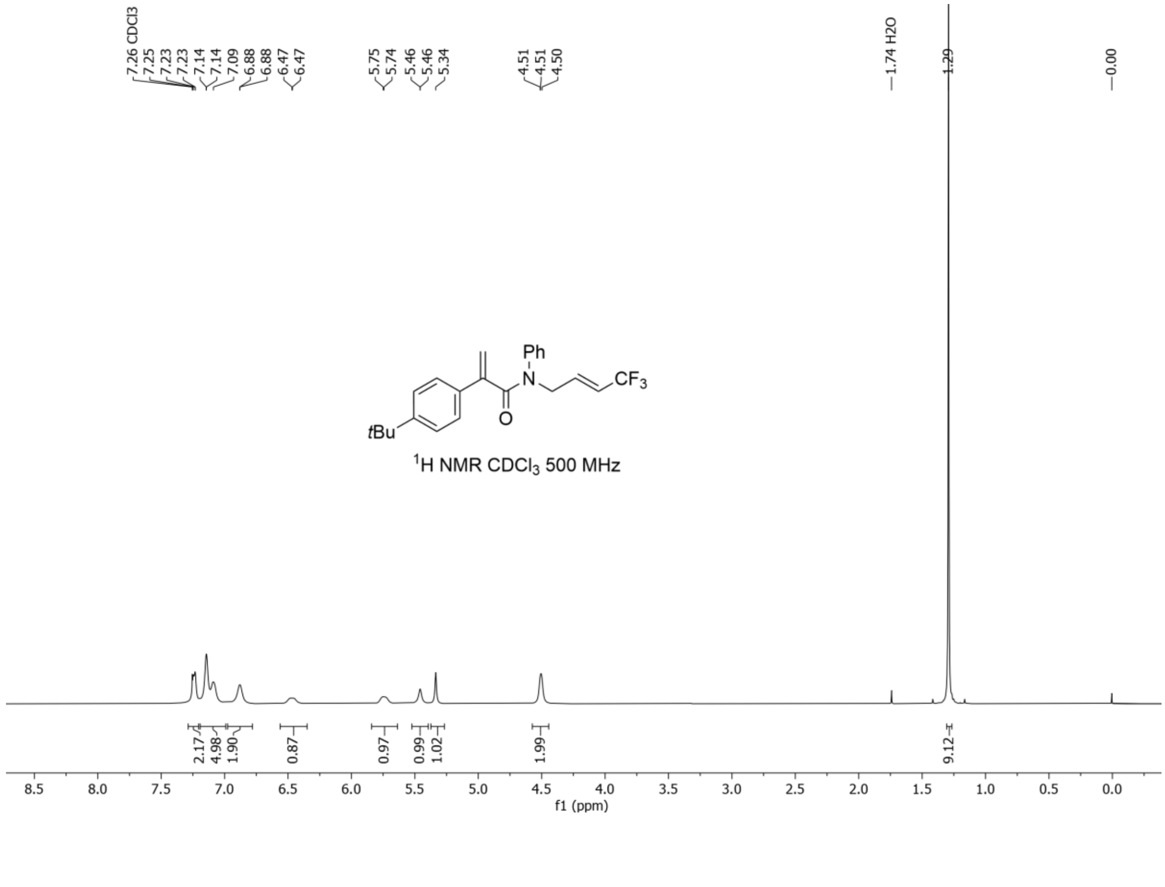

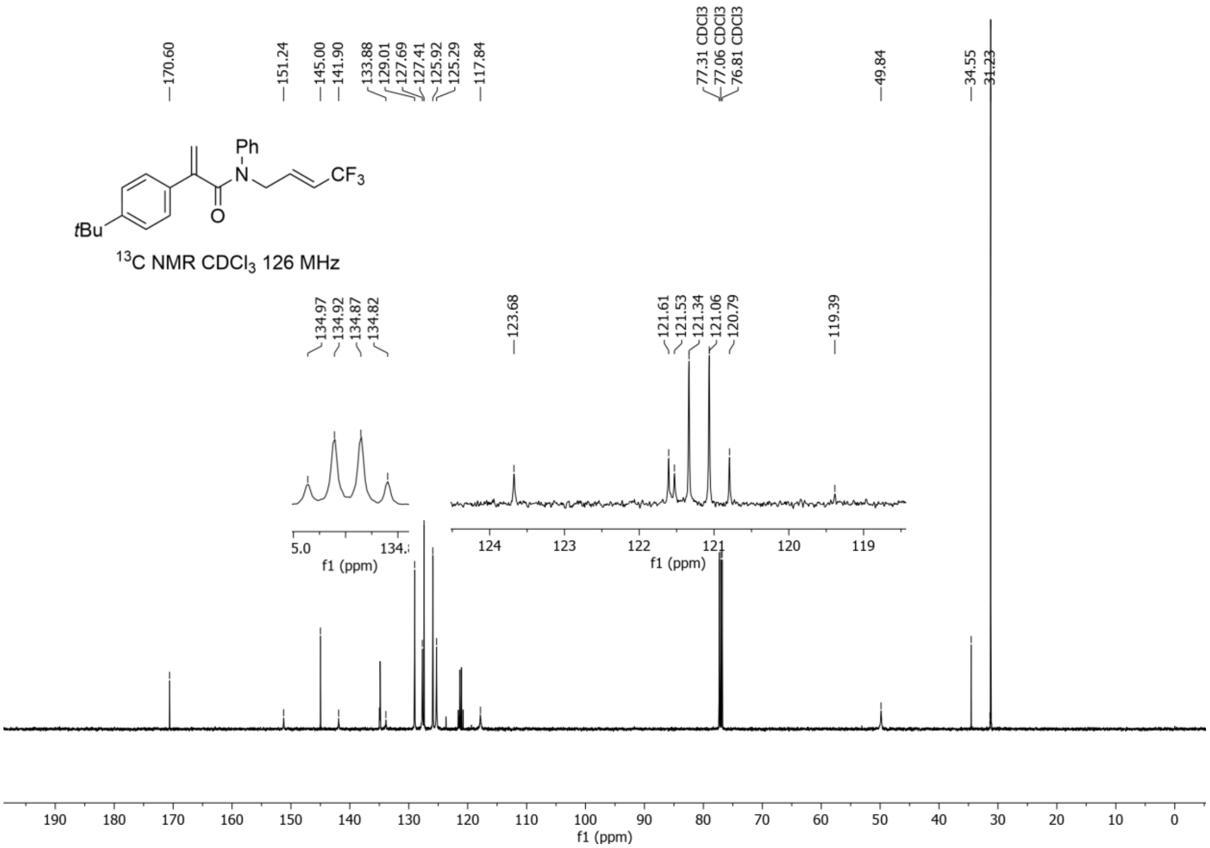
170.60
151.24
145.00
141.90
133.88
129.01
127.69
127.41
125.92
125.29
117.84
77.31 CDCl3
77.06 CDCl3
76.81 CDCl3
49.84
34.55
31.23
Ph
N
CF3
O
tBu
13C NMR CDCl3 126 MHz
134.97
134.92
134.87
134.82
123.68
121.61
121.53
121.34
121.06
120.79
119.39
f1 (ppm)

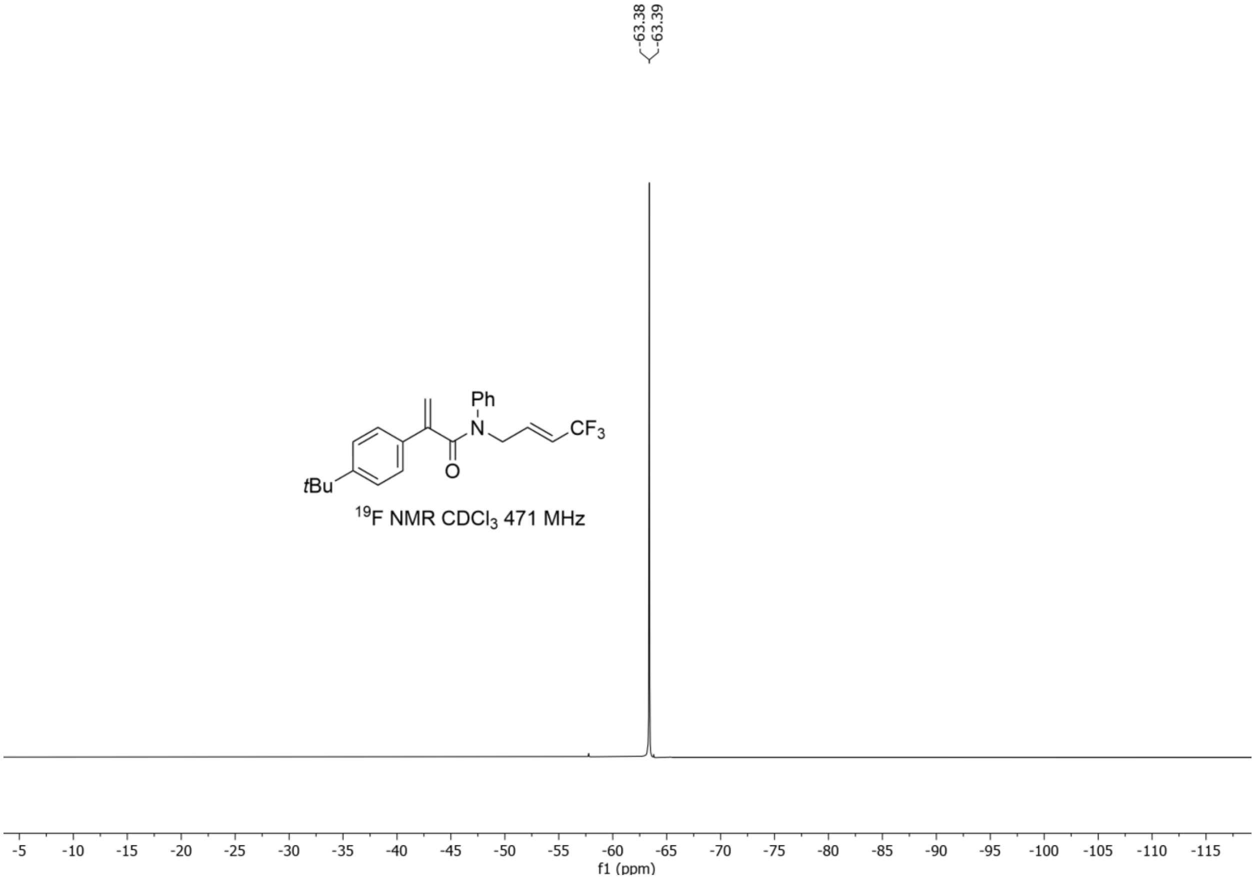
-63.38
-63.39
Ph
N
CF3
O
tBu
19F NMR CDCl3 471 MHz
f1 (ppm)

(*E*)-2-(4-fluorophenyl)-*N*-phenyl-*N*-(4,4,4-trifluorobut-2-en-1-yl)acrylamide (2e)

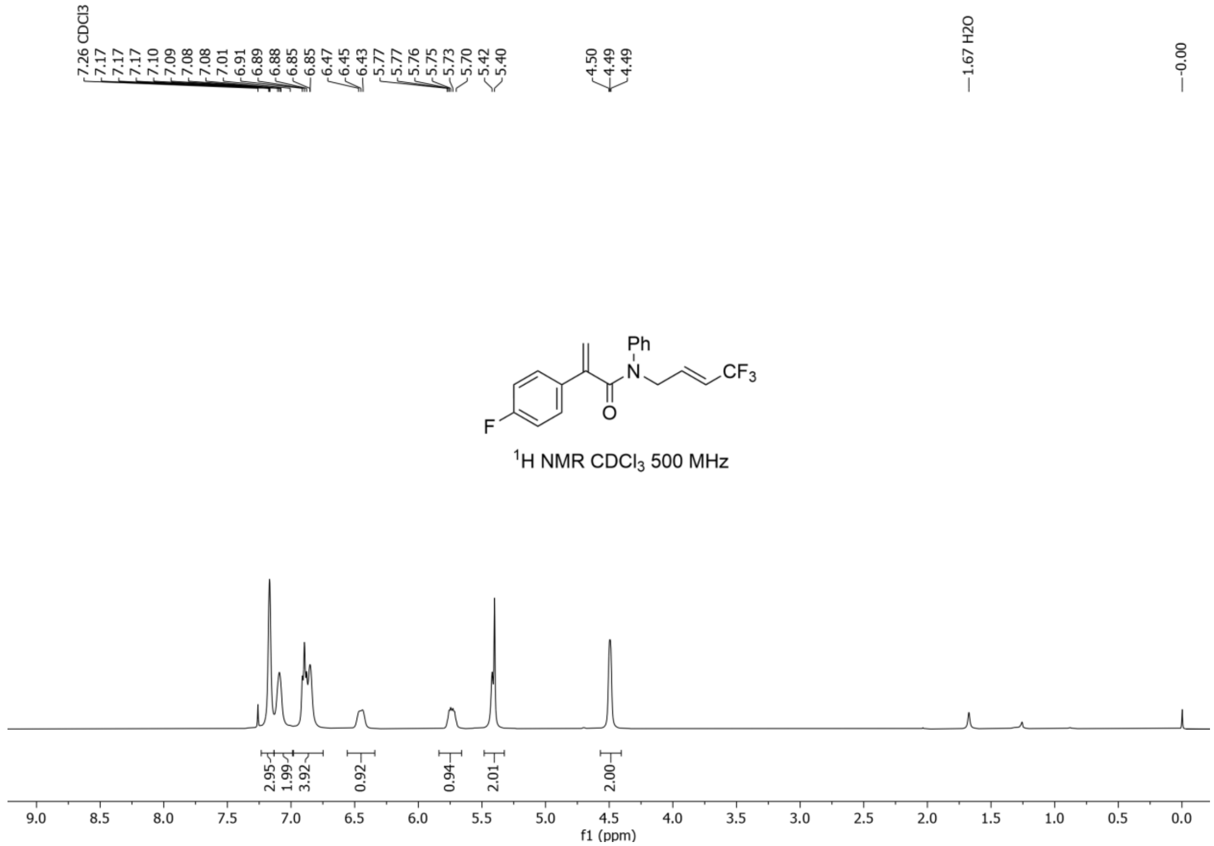

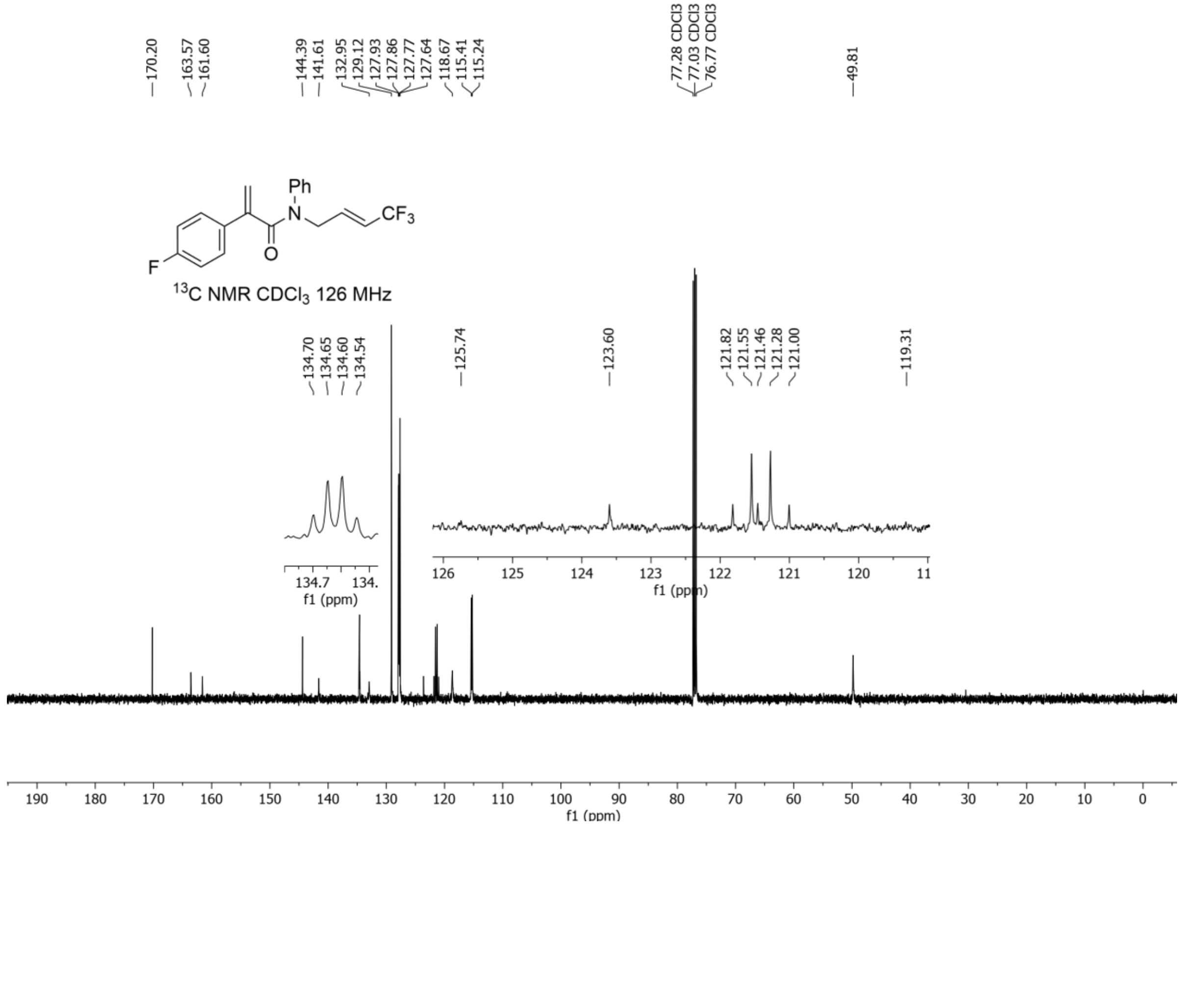

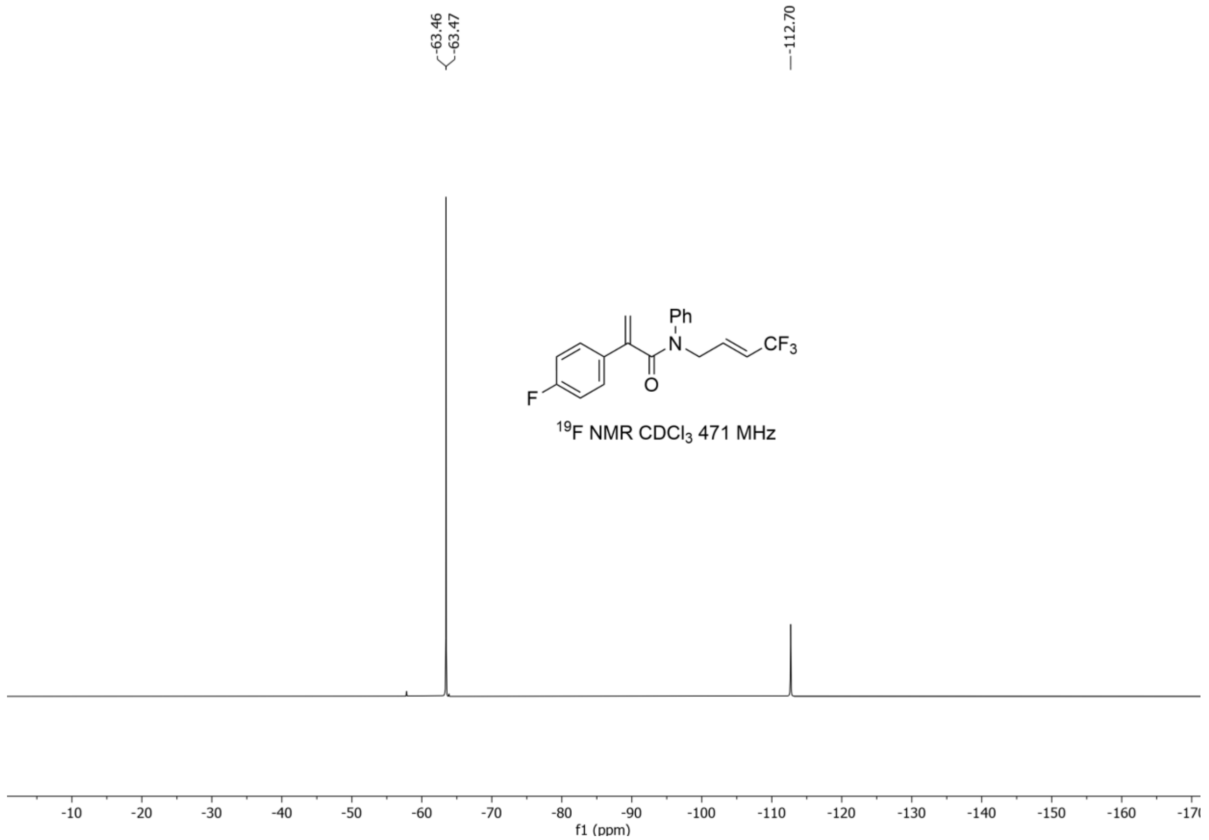

(*E*)-2-(4-chlorophenyl)-*N*-phenyl-*N*-(4,4,4-trifluorobut-2-en-1-yl)acrylamide (2f)

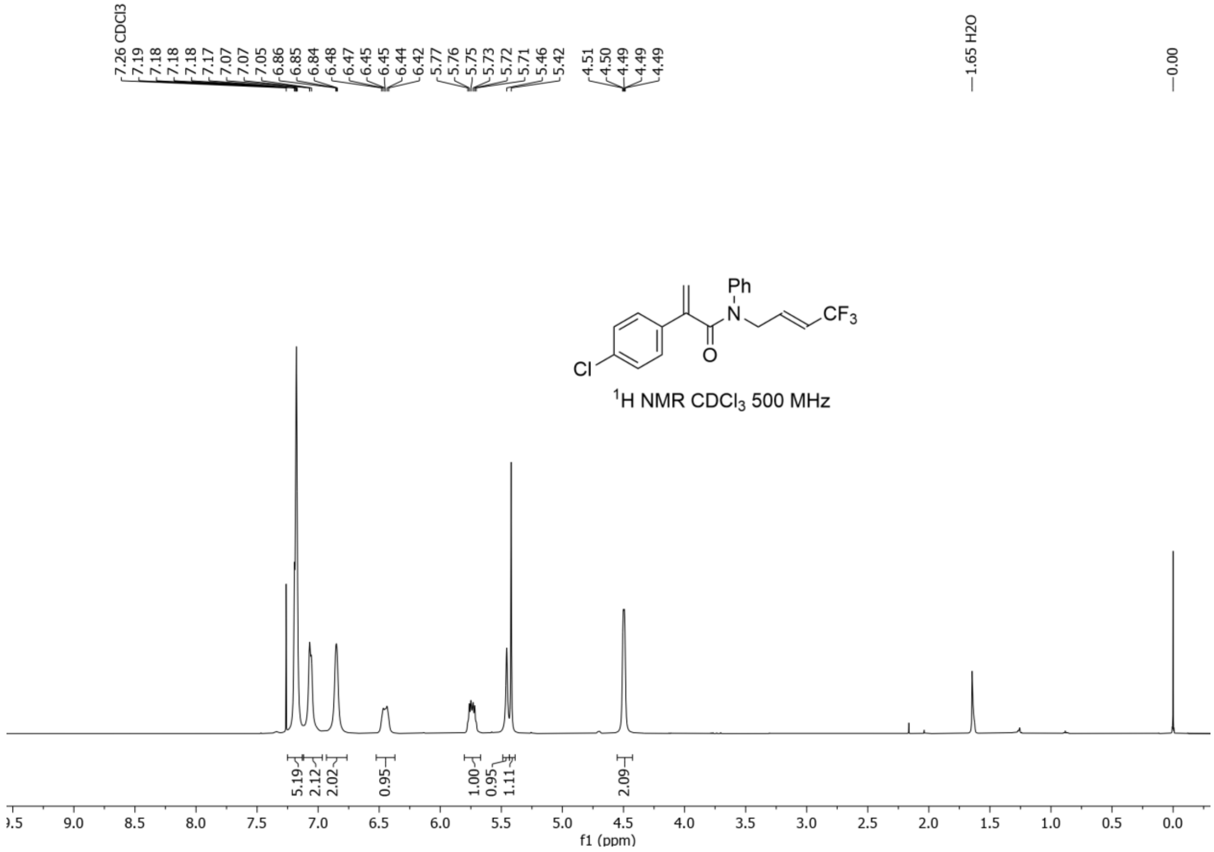

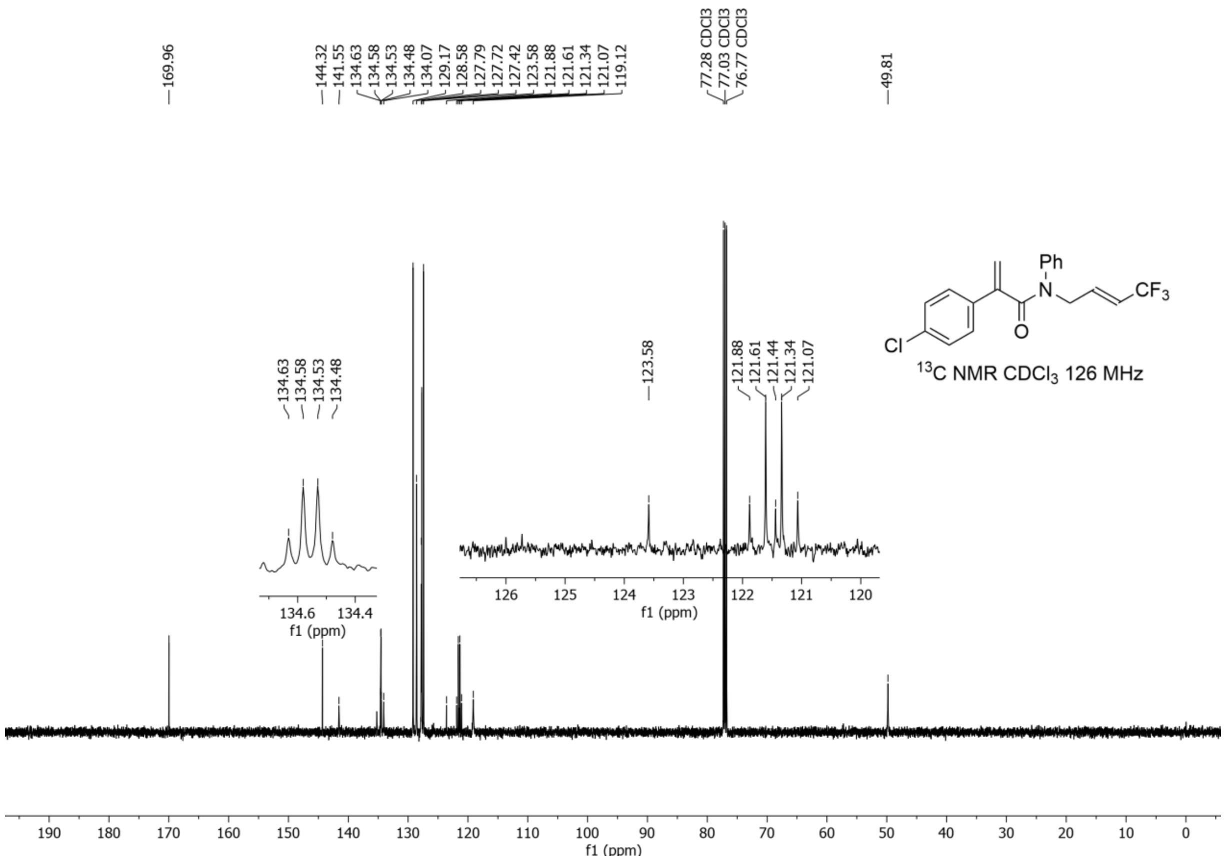

169.96
144.32
141.55
134.63
134.58
134.53
134.48
134.07
129.17
128.58
127.79
127.72
127.42
123.58
121.88
121.61
121.34
121.07
119.12
77.28 CDCl3
77.03 CDCl3
76.77 CDCl3
49.81
134.63
134.58
134.53
134.48
134.6
134.4
f1 (ppm)
123.58
121.88
121.61
121.44
121.34
121.07
126
125
124
123
122
121
120
f1 (ppm)
Ph
N
CF3
Cl
O
13C NMR CDCl3 126 MHz
190
180
170
160
150
140
130
120
110
100
90
80
70
60
50
40
30
20
10
0
f1 (ppm)

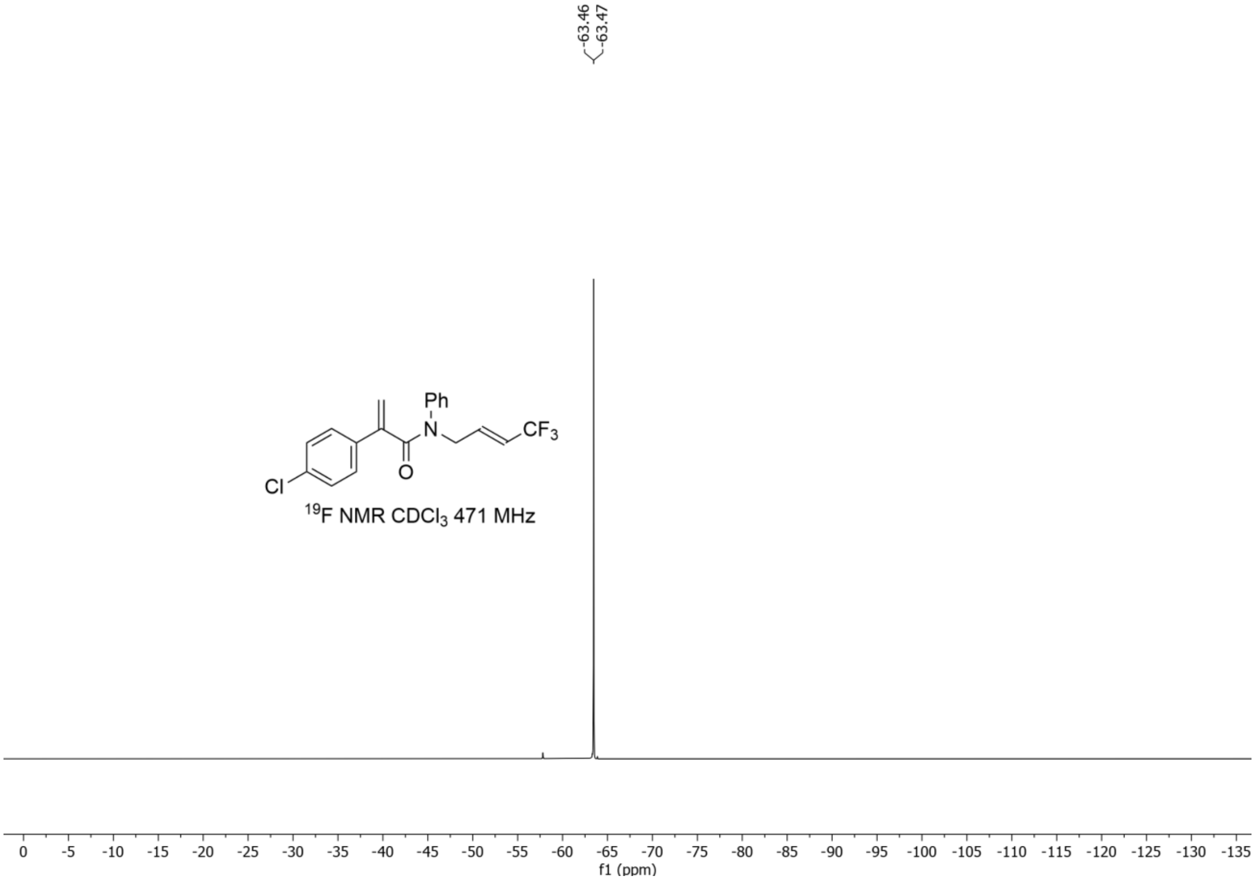

-63.46
-63.47
Ph
N
CF3
Cl
O
19F NMR CDCl3 471 MHz
0
-5
-10
-15
-20
-25
-30
-35
-40
-45
-50
-55
-60
-65
-70
-75
-80
-85
-90
-95
-100
-105
-110
-115
-120
-125
-130
-135
f1 (ppm)

(*E*)-2-(4-bromophenyl)-*N*-phenyl-*N*-(4,4,4-trifluorobut-2-en-1-yl)acrylamide (2g)

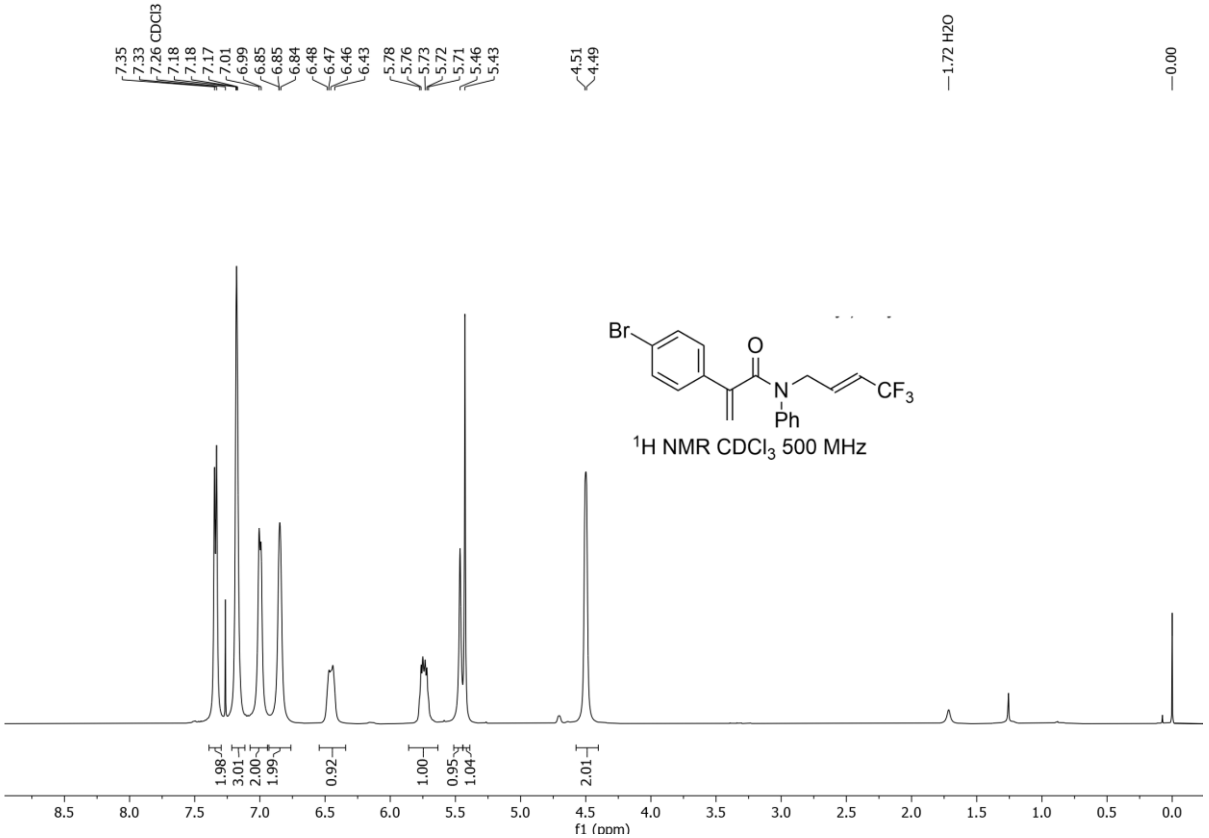

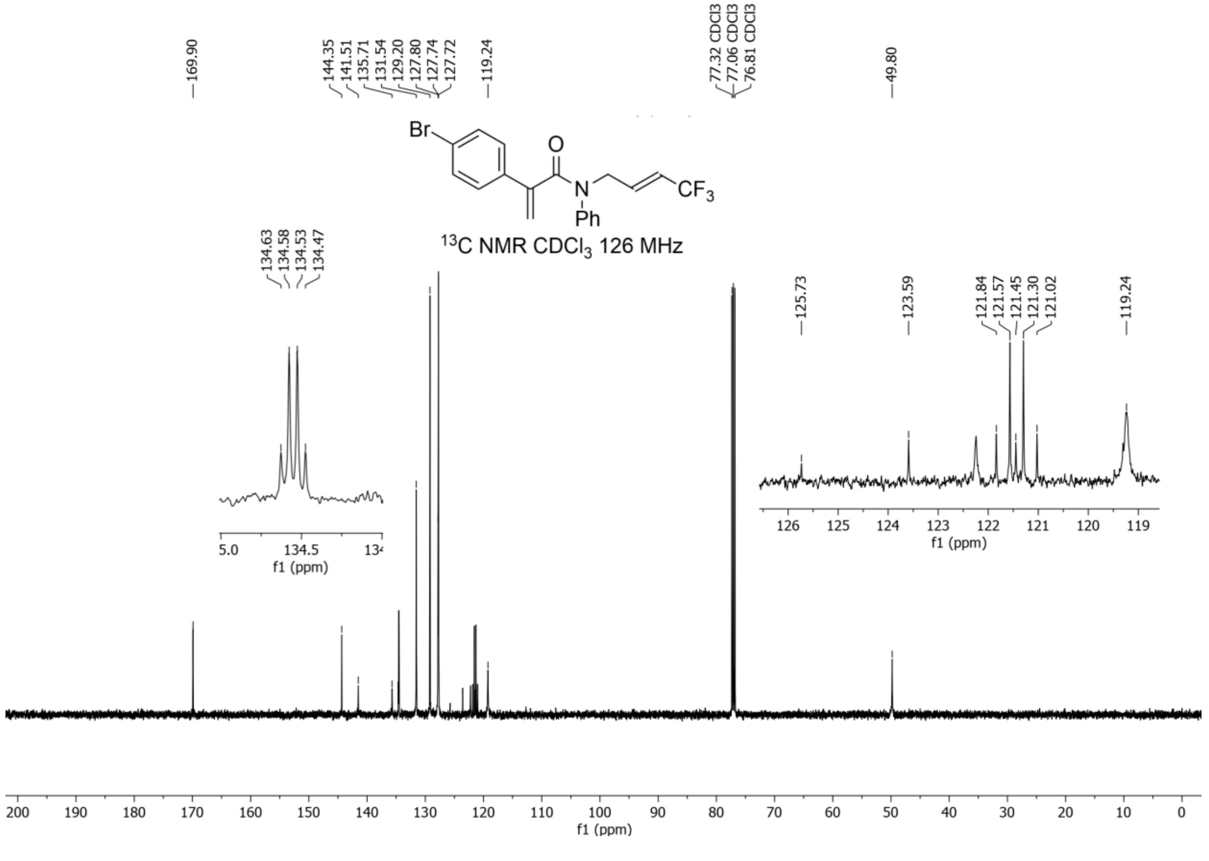

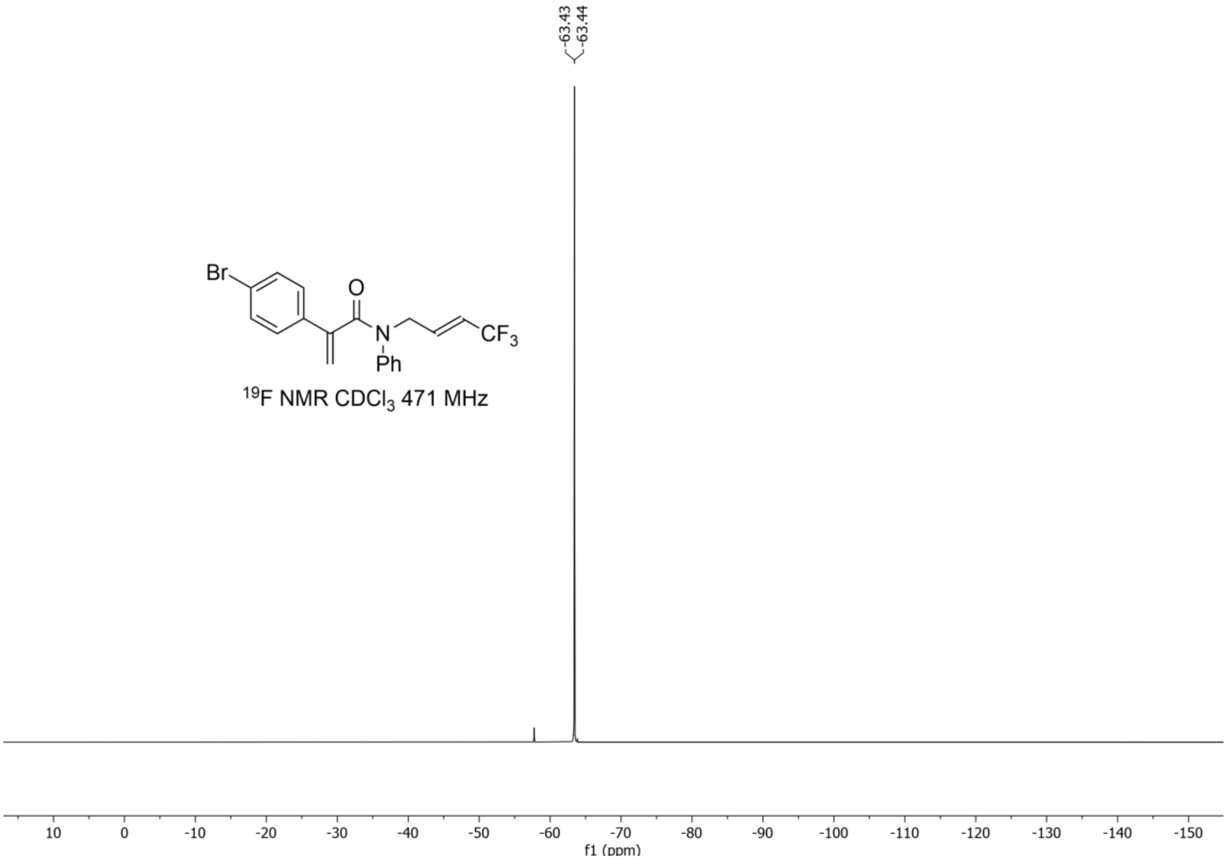

(*E*)-2-(2-bromophenyl)-*N*-phenyl-*N*-(4,4,4-trifluorobut-2-en-1-yl)acrylamide (2h)

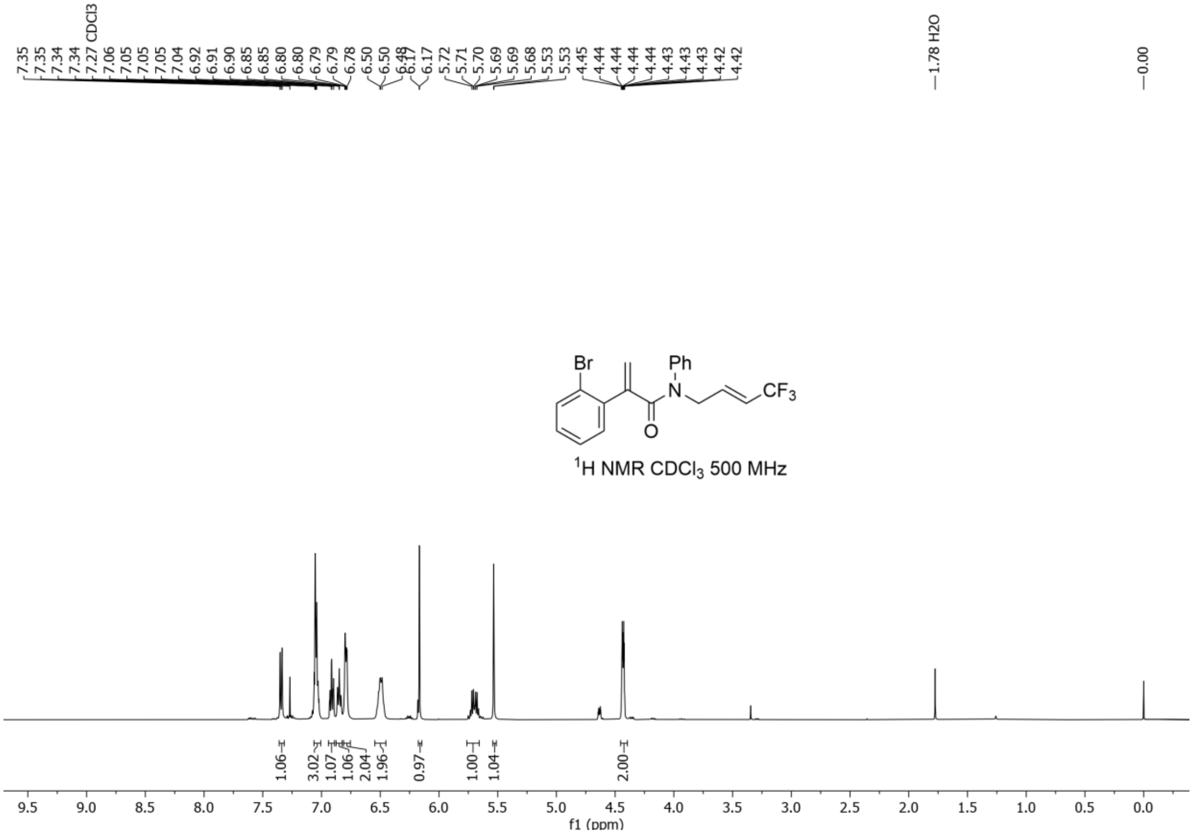

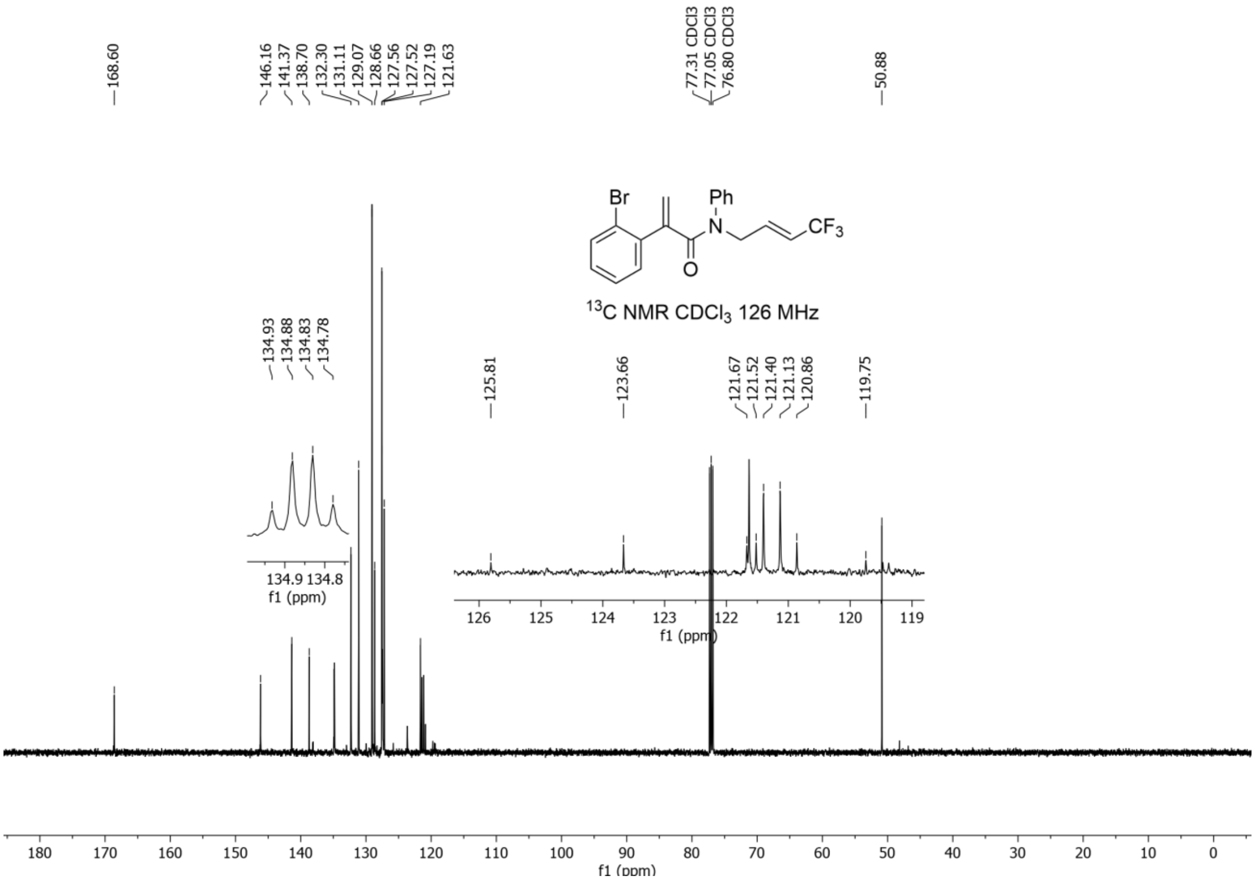

168.60
146.16
141.37
138.70
132.30
131.11
129.07
128.66
127.56
127.52
127.19
121.63
77.31 CDCl3
77.05 CDCl3
76.80 CDCl3
50.88
Br
Ph
N
CF3
O
13C NMR CDCl3 126 MHz
134.93
134.88
134.83
134.78
125.81
123.66
121.67
121.52
121.40
121.13
120.86
119.75
f1 (ppm)

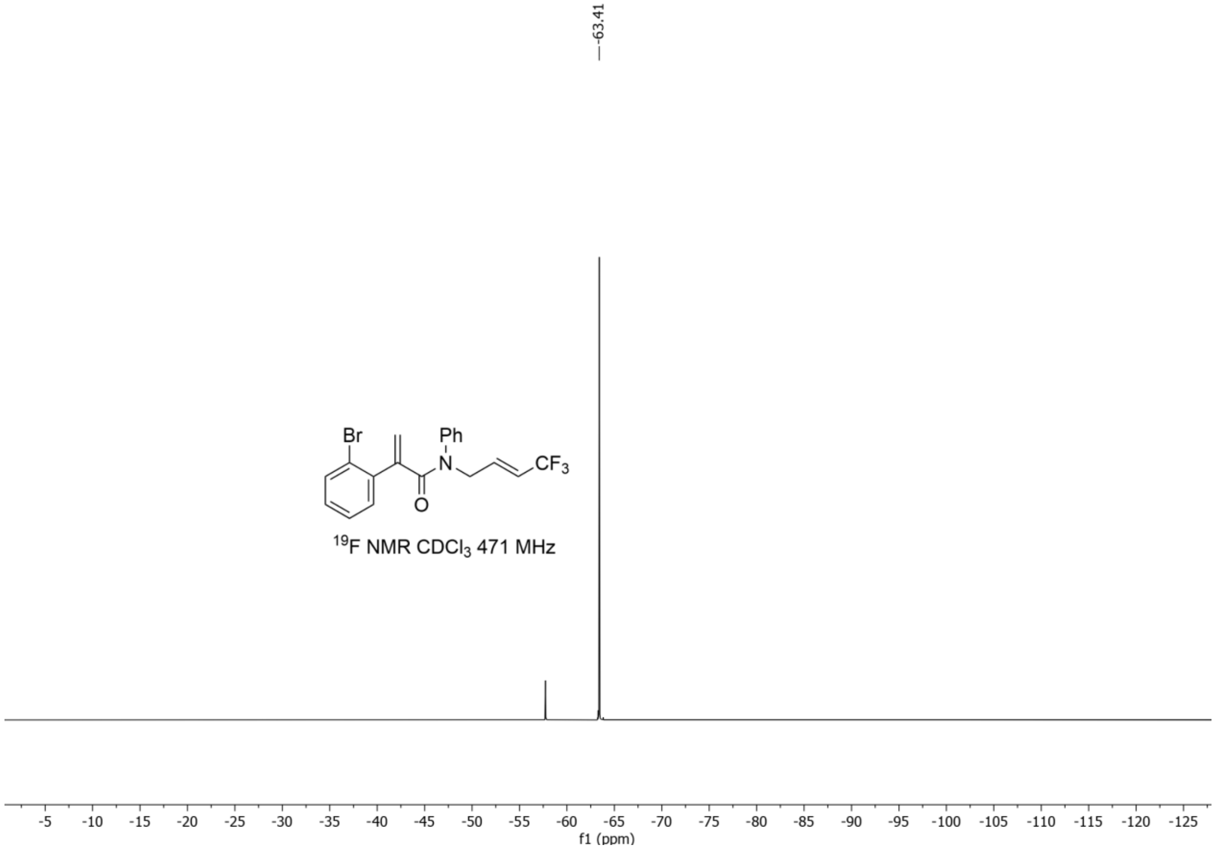

-63.41
Br
Ph
N
CF3
O
19F NMR CDCl3 471 MHz
f1 (ppm)

(*E*)-*N*-phenyl-*N*-(4,4,4-trifluorobut-2-en-1-yl)-2-(4-(trifluoromethyl)phenyl)acrylamide (2i)

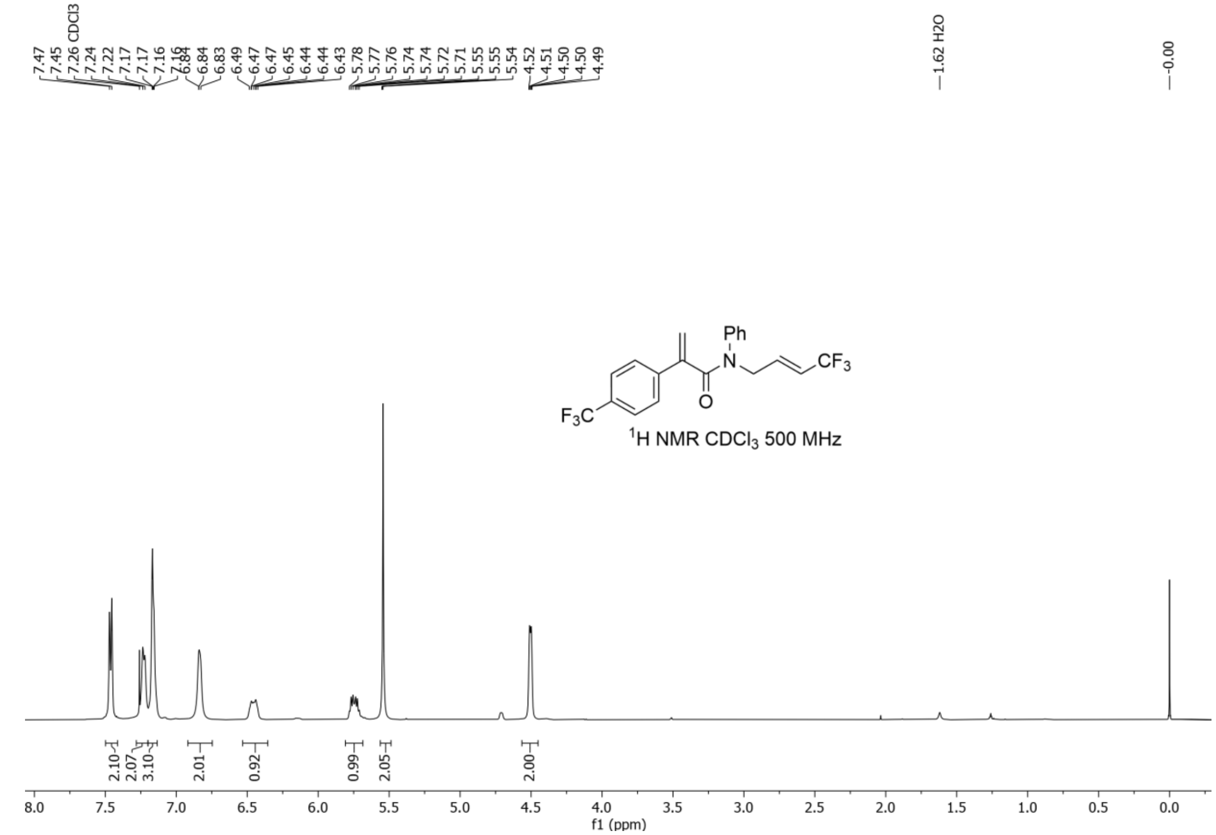

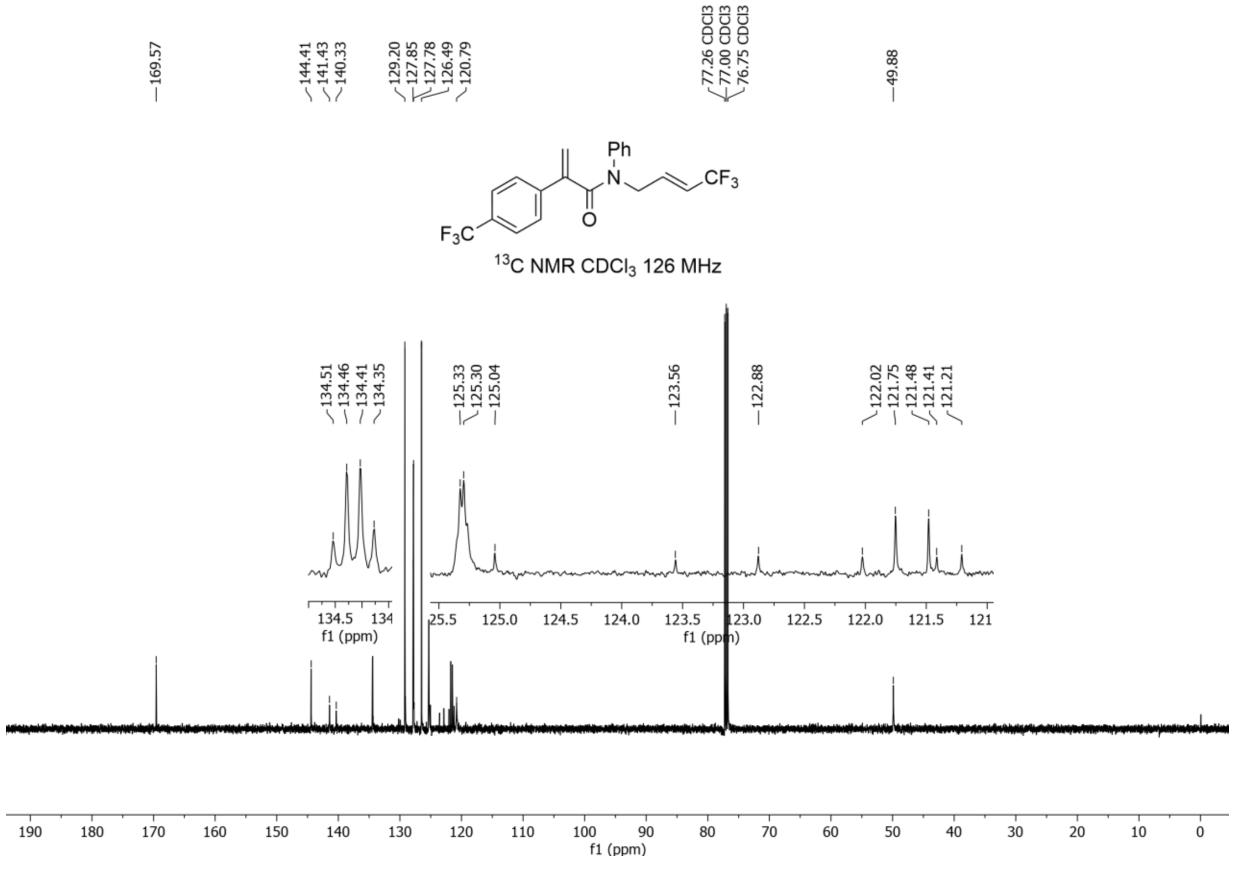

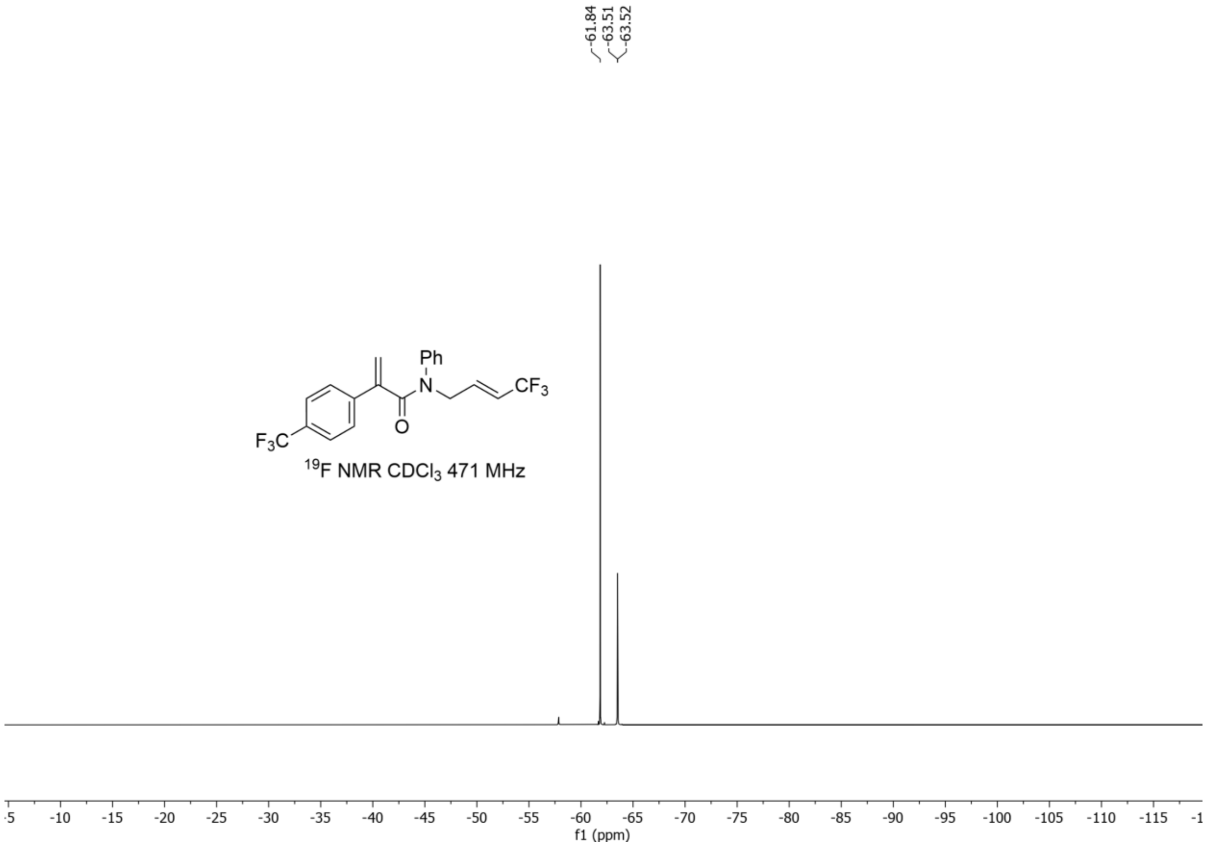

(*E*)-*N*-phenyl-*N*-(4,4,4-trifluorobut-2-en-1-yl)-2-(3-(trifluoromethyl)phenyl)acrylamide (2j)

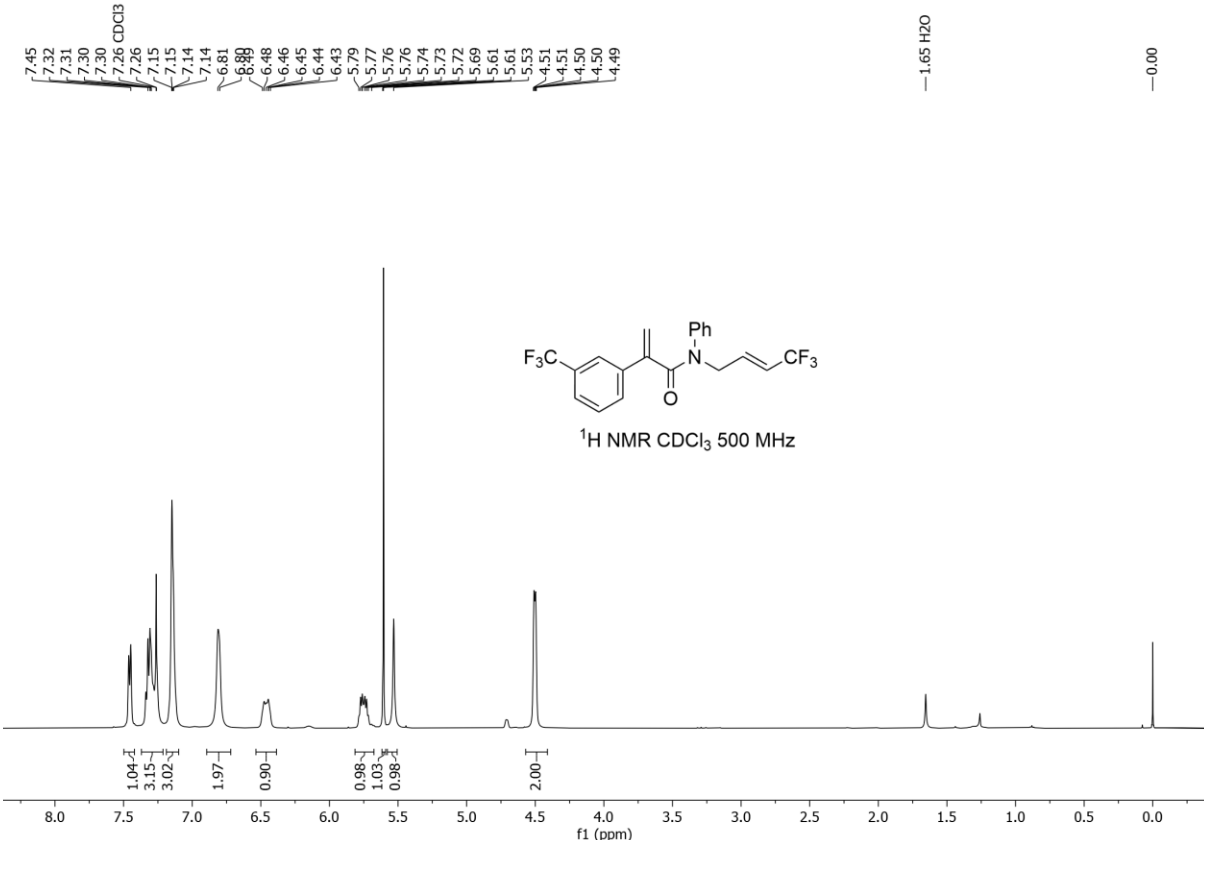

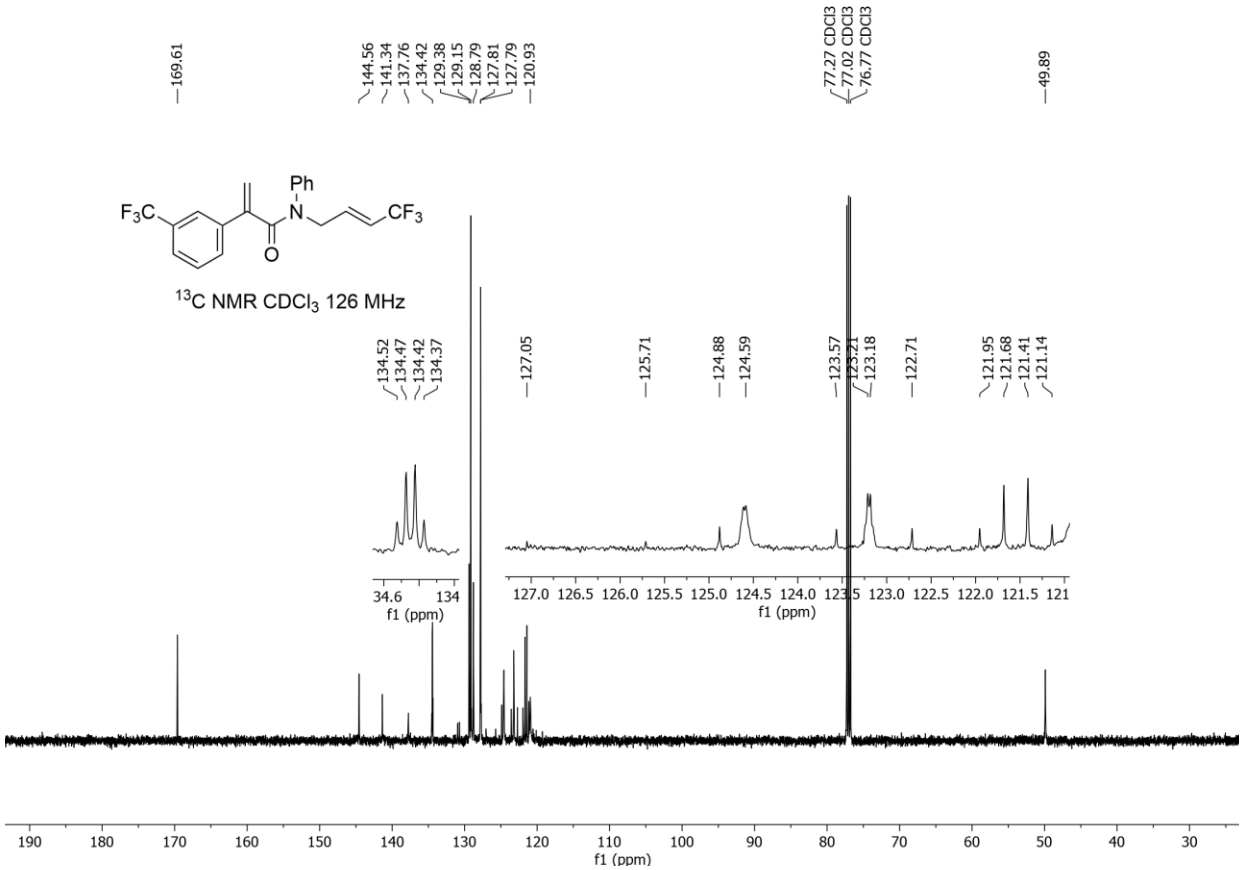
169.61
144.56
141.34
137.76
134.42
129.38
129.15
128.79
127.81
127.79
120.93
77.27 CDCl3
77.02 CDCl3
76.77 CDCl3
49.89
Ph
N
F_3C
CF_3
O
^{13}C NMR $CDCl_3$ 126 MHz
134.52
134.47
134.42
134.37
127.05
125.71
124.88
124.59
123.57
123.21
123.18
122.71
121.95
121.68
121.41
121.14
f1 (ppm)
f1 (ppm)
f1 (ppm)

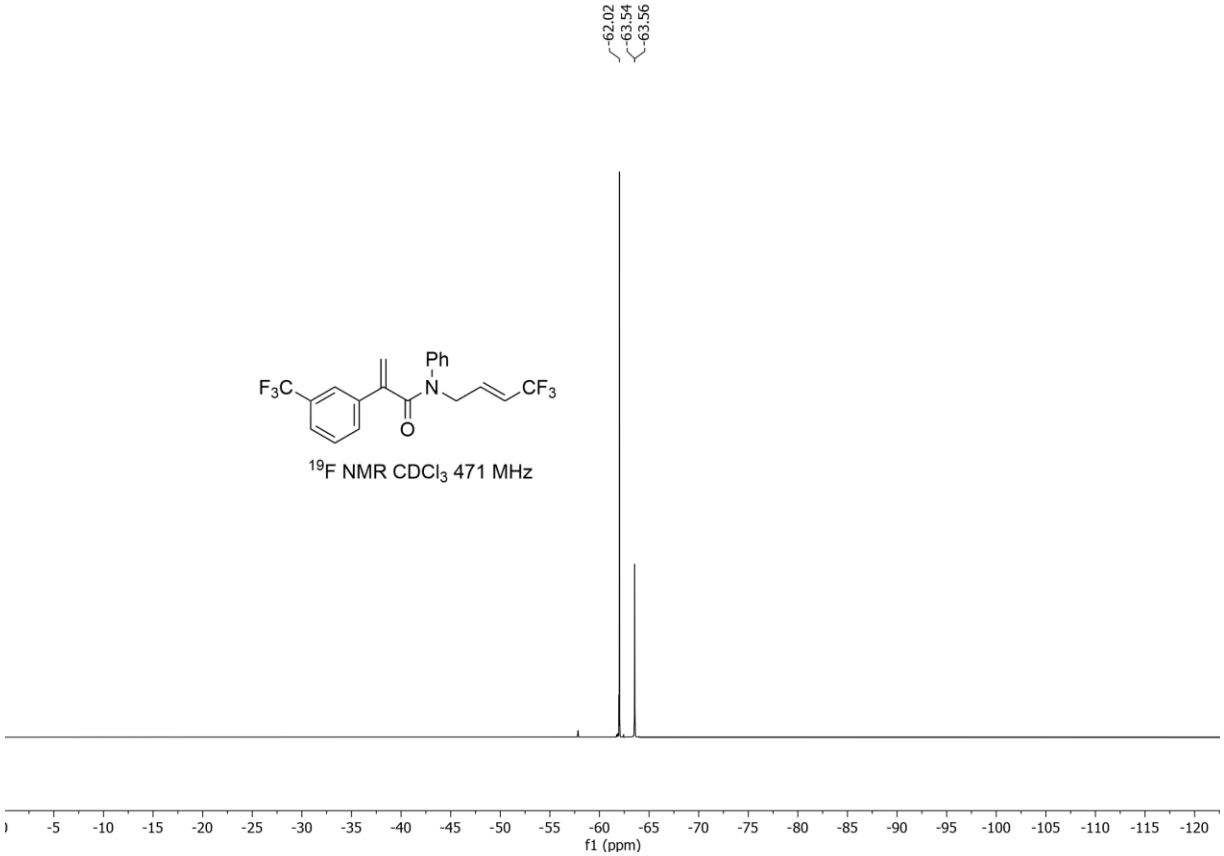
-62.02
-63.54
-63.56
Ph
N
F_3C
CF_3
O
^{19}F NMR $CDCl_3$ 471 MHz
f1 (ppm)

(*E*)-*N*-phenyl-2-(*o*-tolyl)-*N*-(4,4,4-trifluorobut-2-en-1-yl)acrylamide (2k)

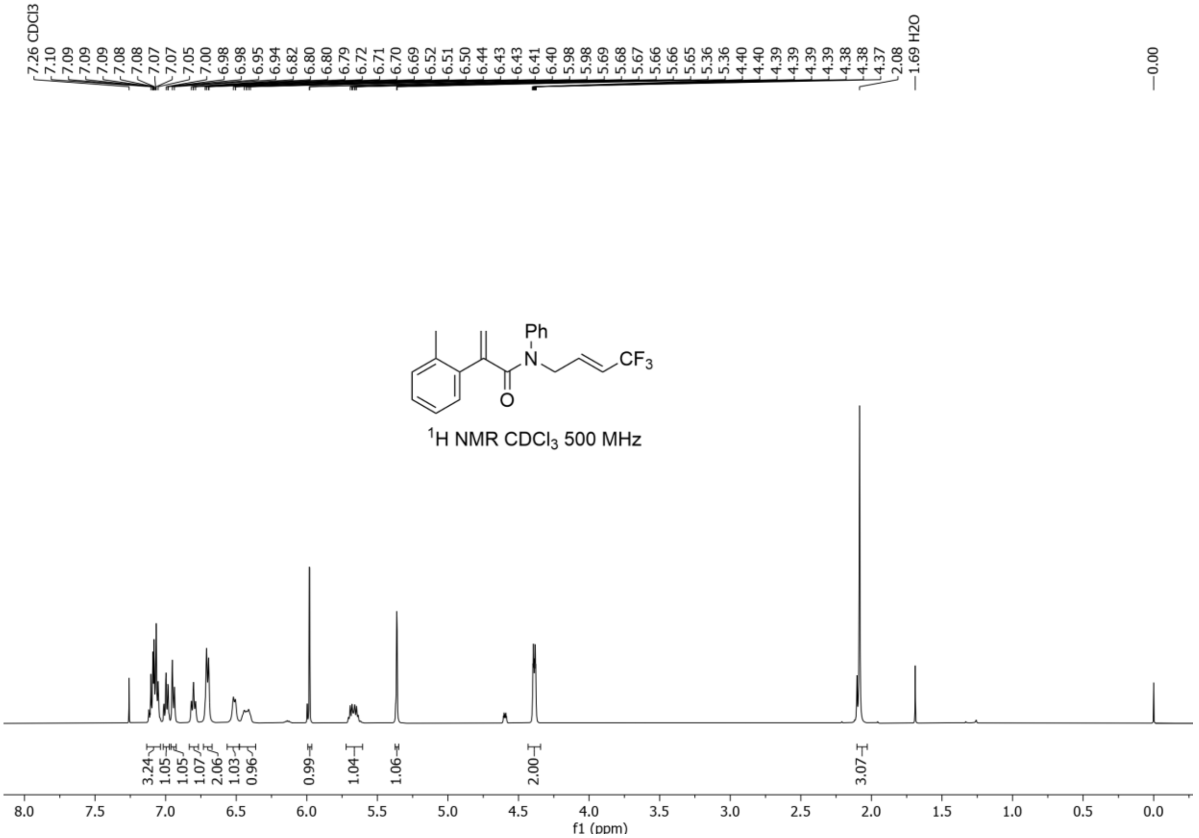

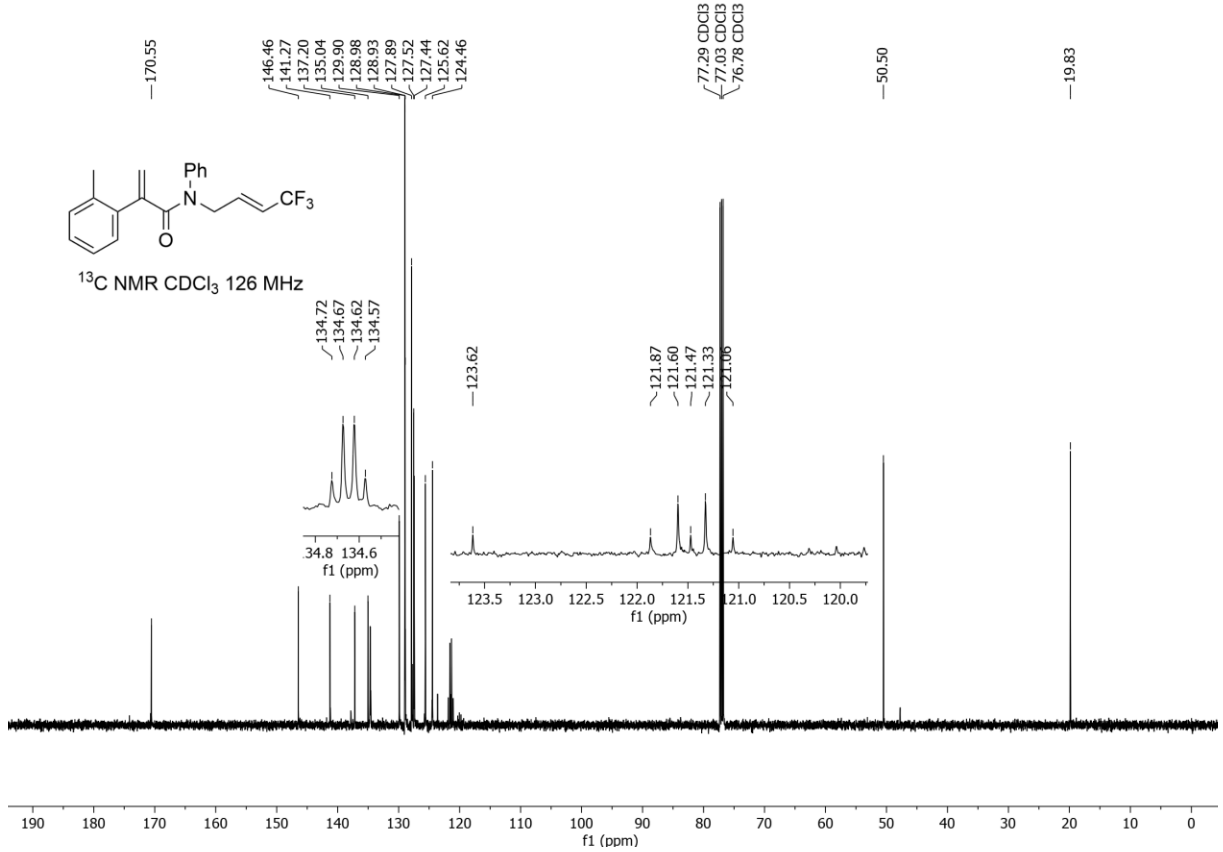

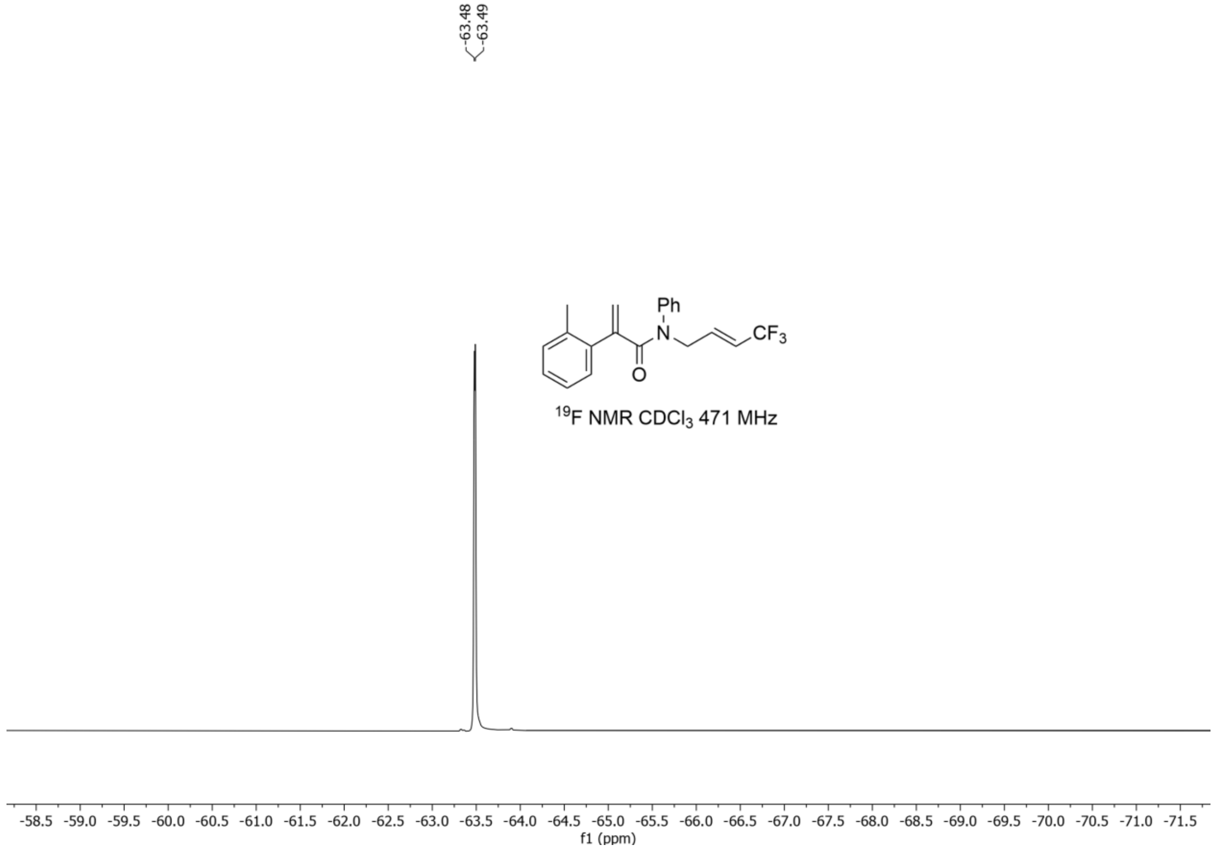

(*E*)-2-(naphthalen-1-yl)-*N*-phenyl-*N*-(4,4,4-trifluorobut-2-en-1-yl)acrylamide (2l)

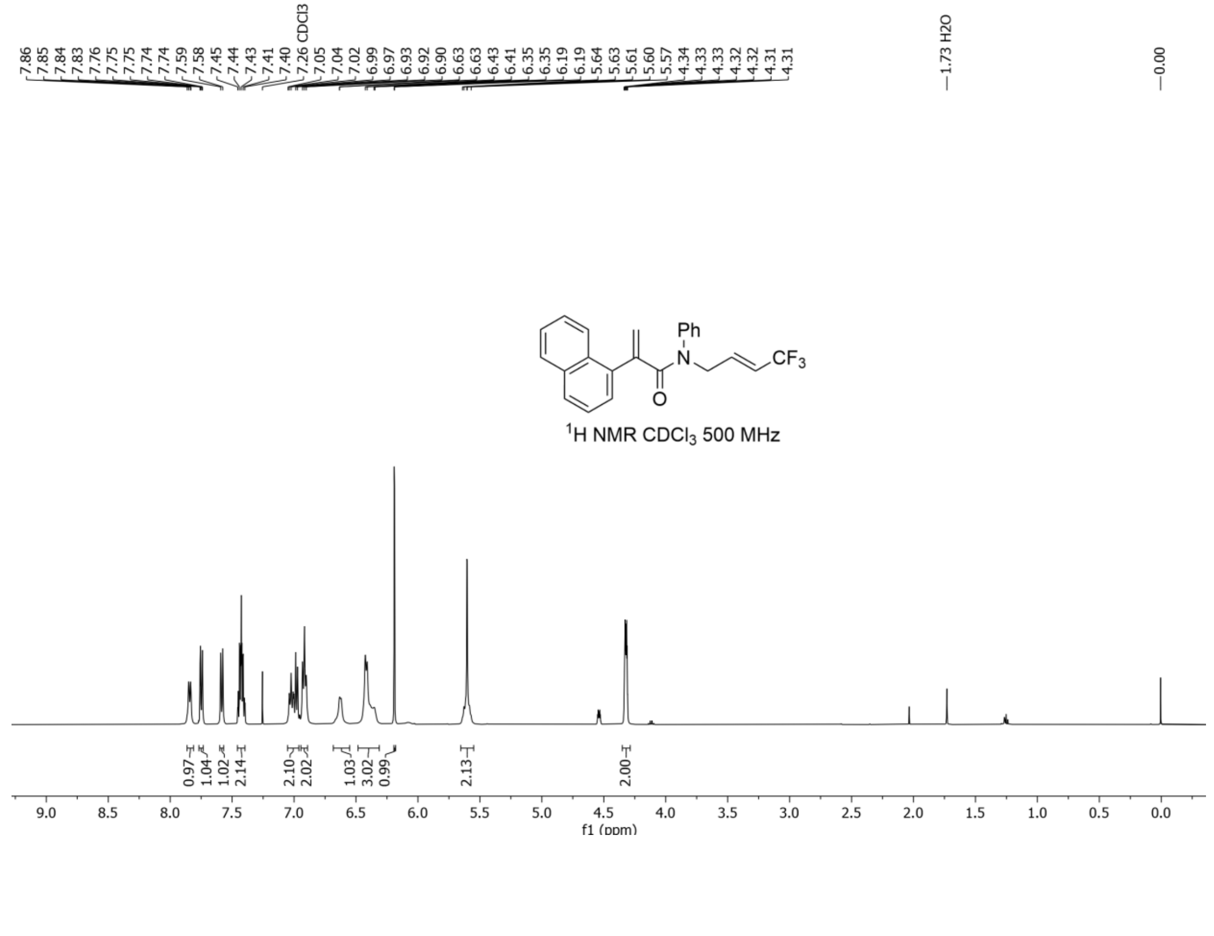

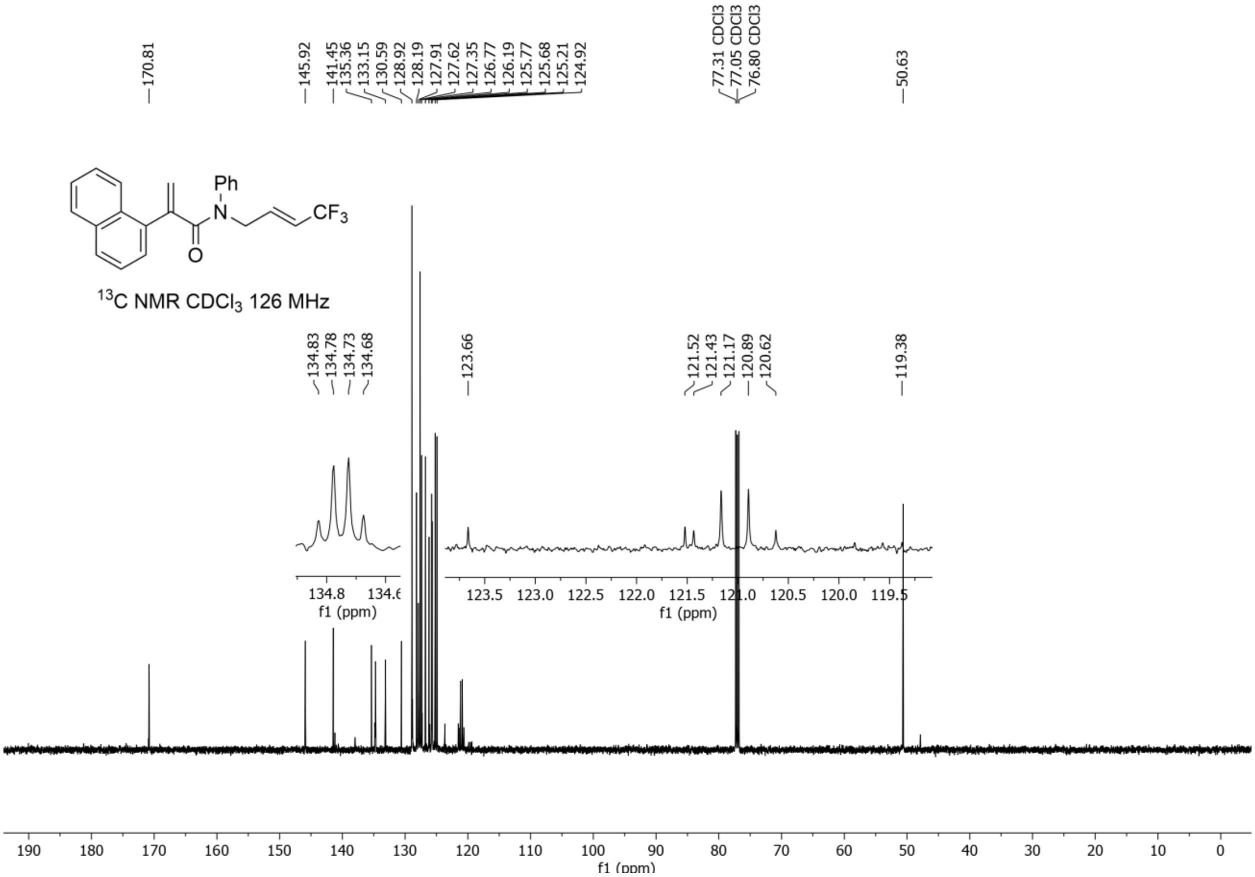

170.81
145.92
141.45
135.36
133.15
130.59
128.92
128.19
127.91
127.62
127.35
126.77
126.19
125.77
125.68
125.21
124.92
77.31 CDCl3
77.05 CDCl3
76.80 CDCl3
50.63
Ph
N
CF3
O
13C NMR CDCl3 126 MHz
134.83
134.78
134.73
134.68
123.66
121.52
121.43
121.17
120.89
120.62
119.38
f1 (ppm)

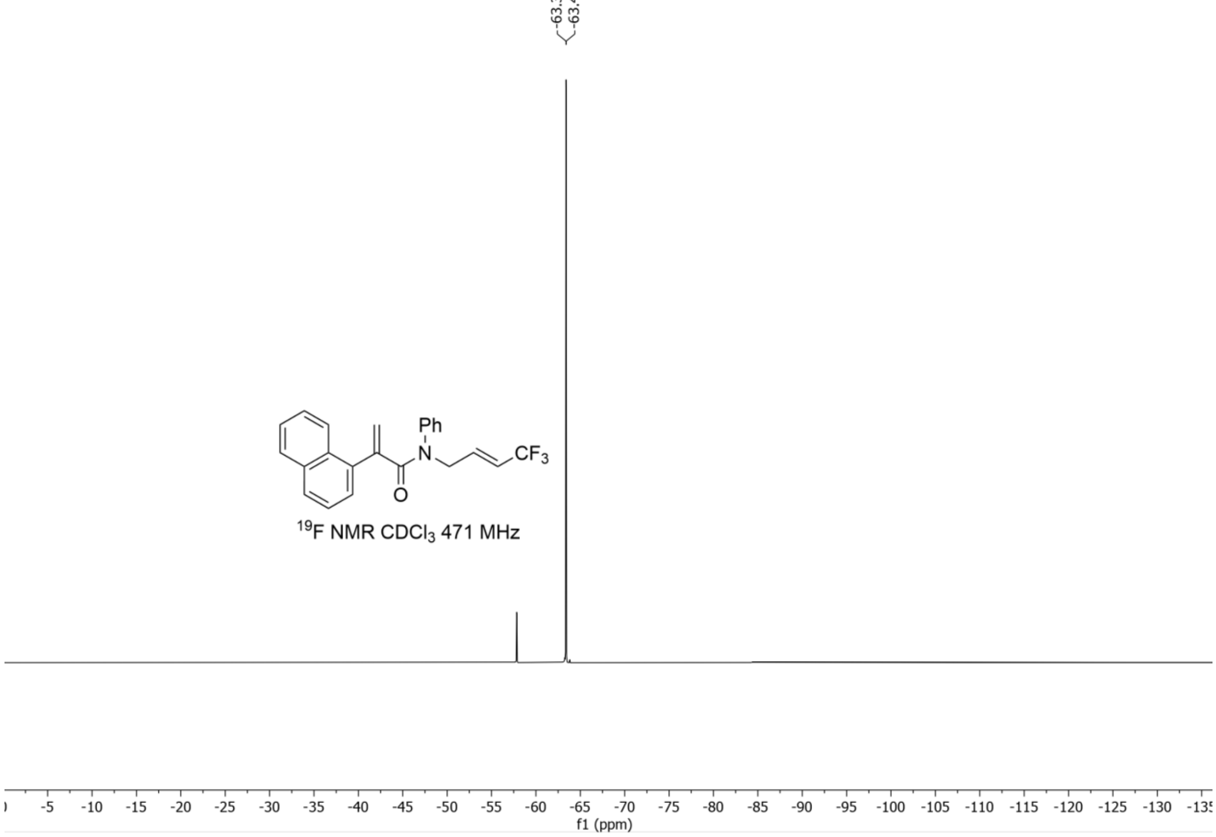

Ph
N
CF3
O
19F NMR CDCl3 471 MHz
f1 (ppm)

(*E*)-2-(2-fluoro-[1,1'-biphenyl]-4-yl)-*N*-phenyl-*N*-(4,4,4-trifluorobut-2-en-1-yl)acrylamide (2m)

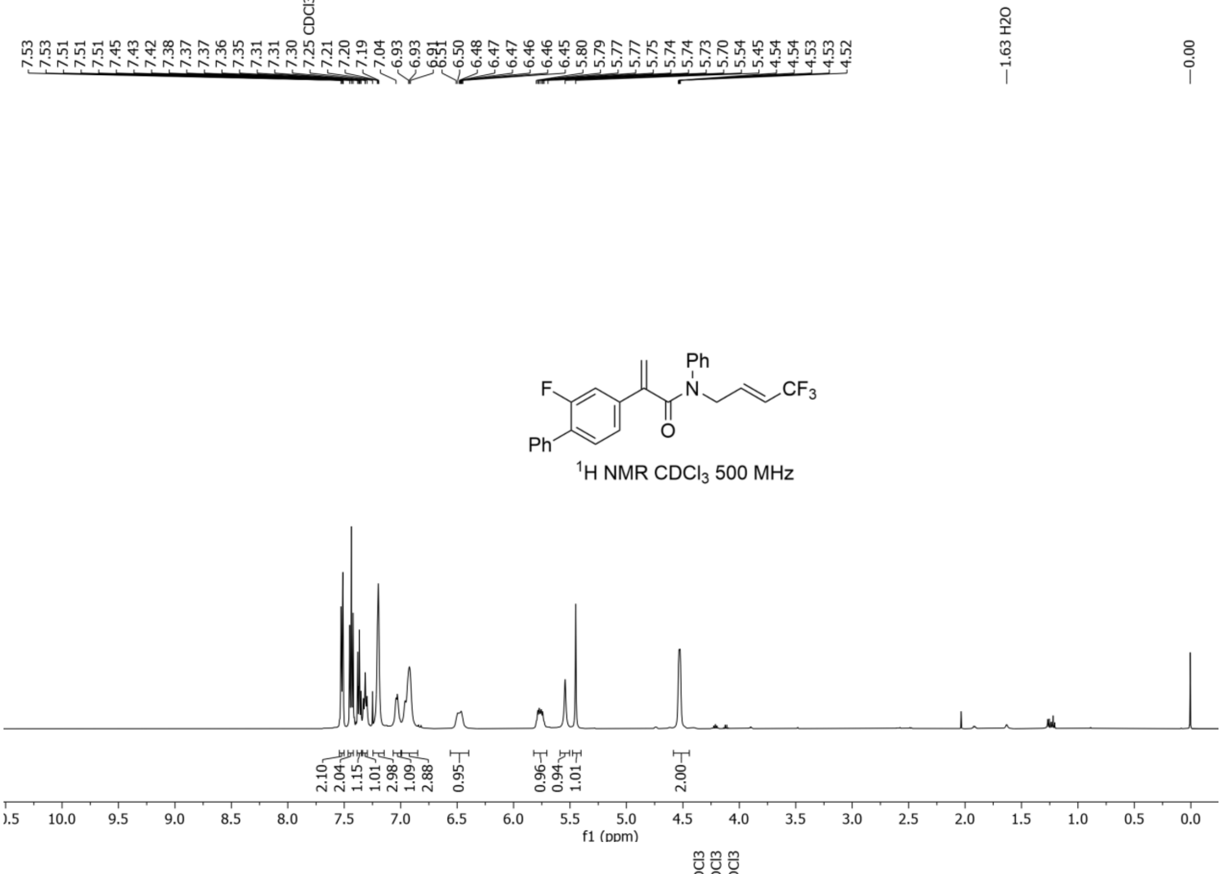

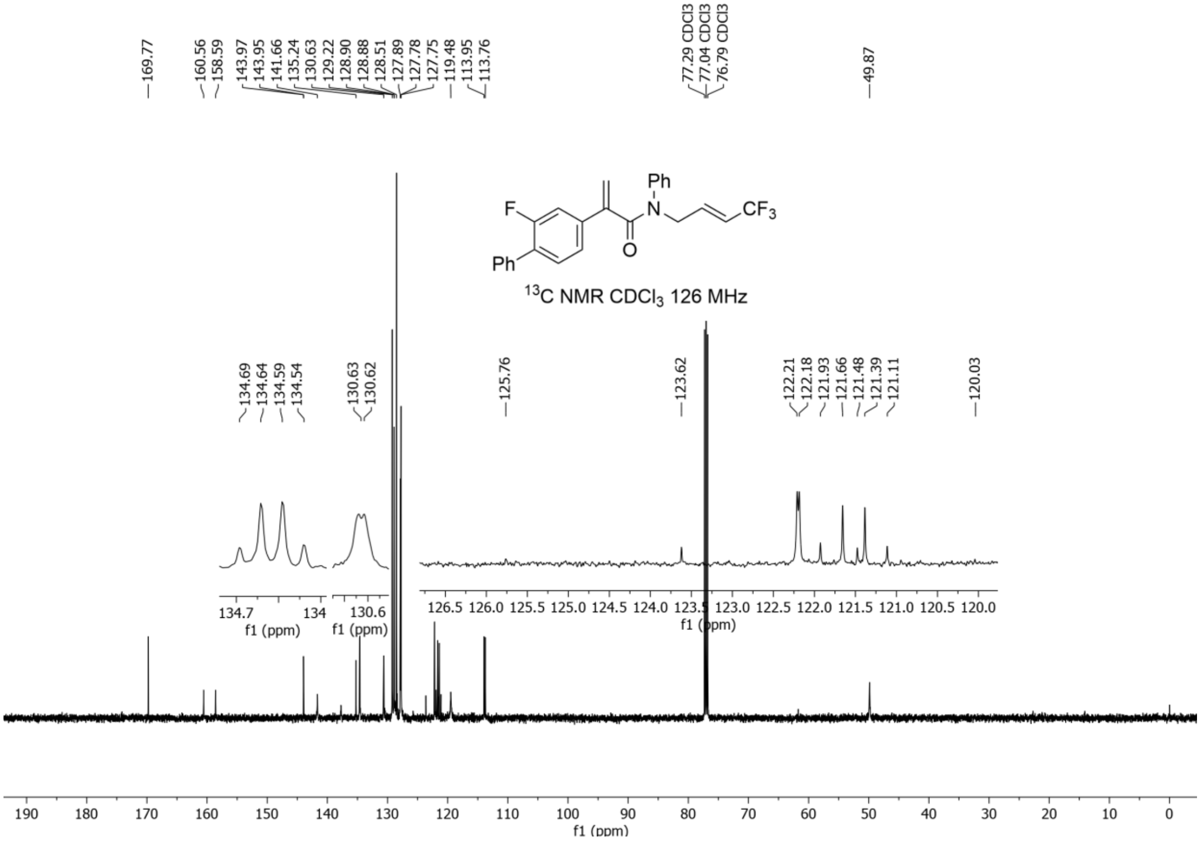

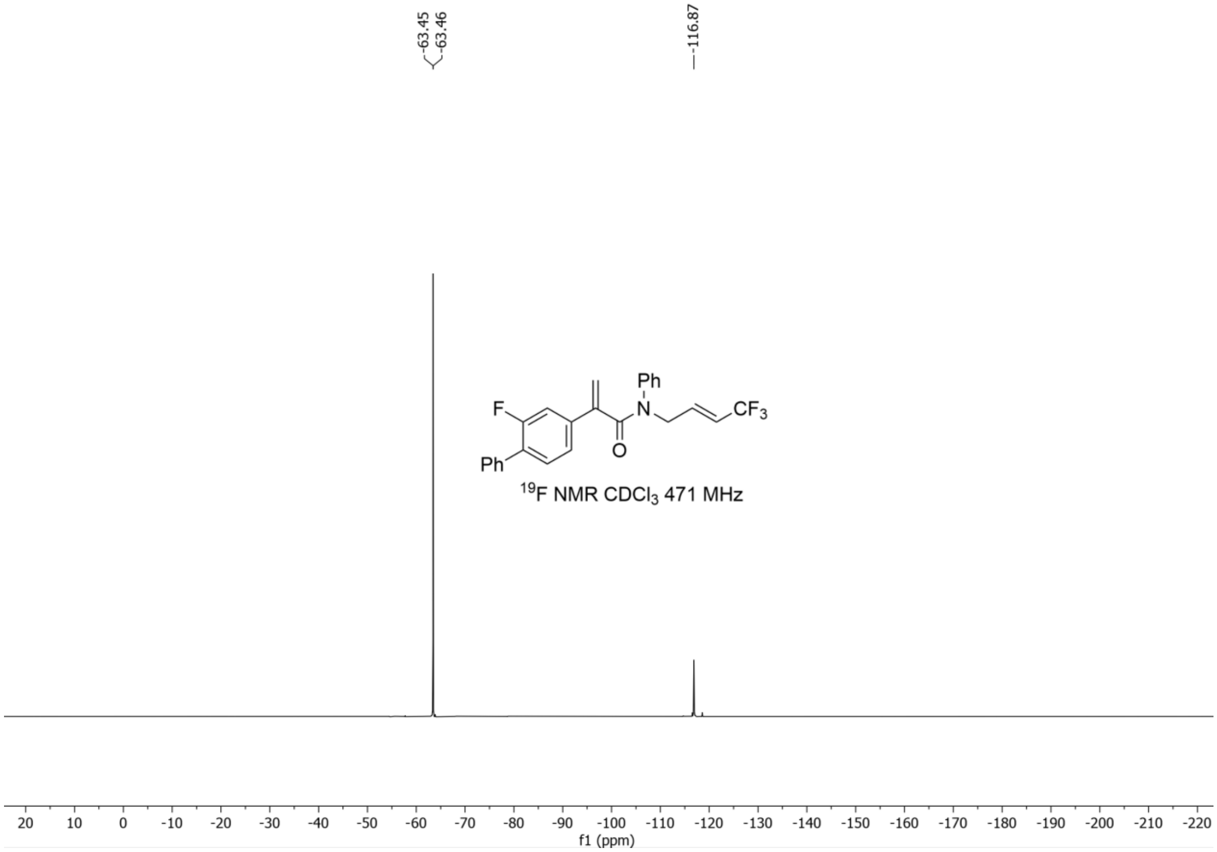

(*E*)-*N*-phenyl-*N*-(4,4,4-trifluorobut-2-en-1-yl)acrylamide (2n)

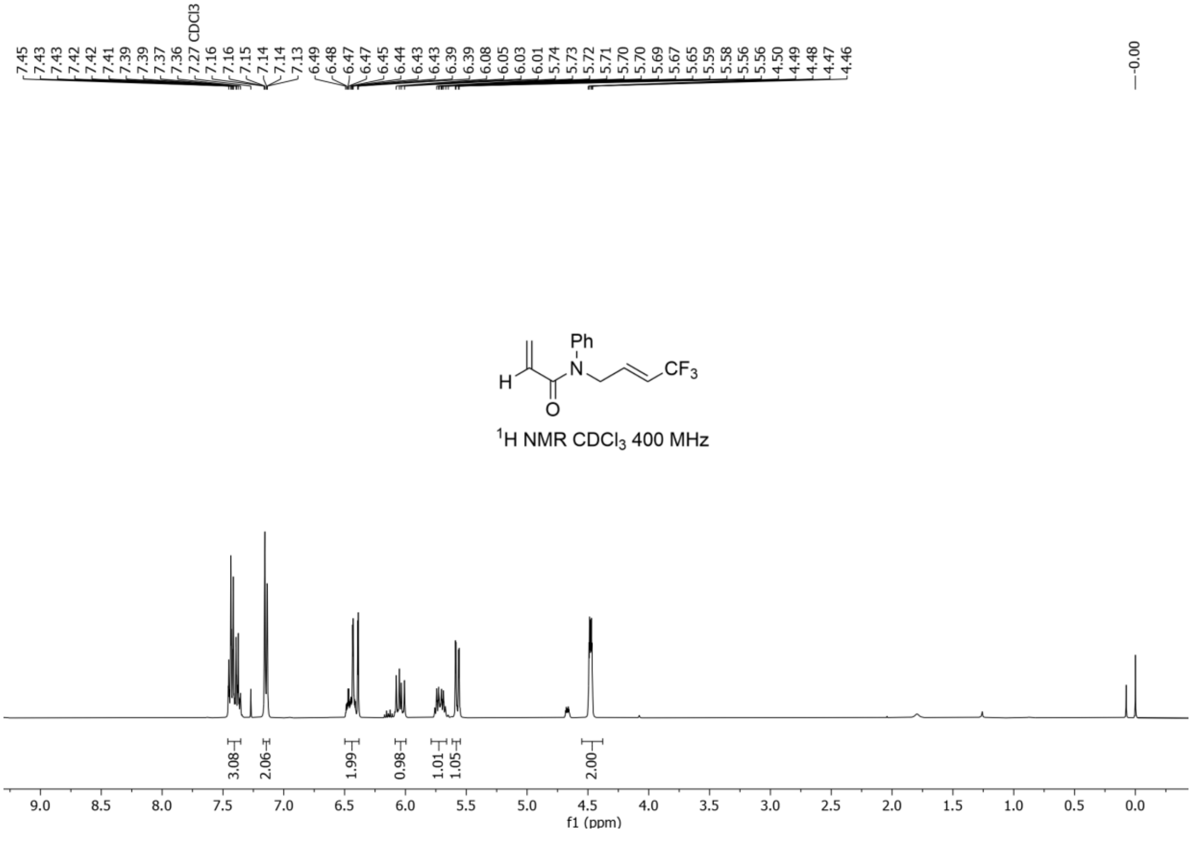

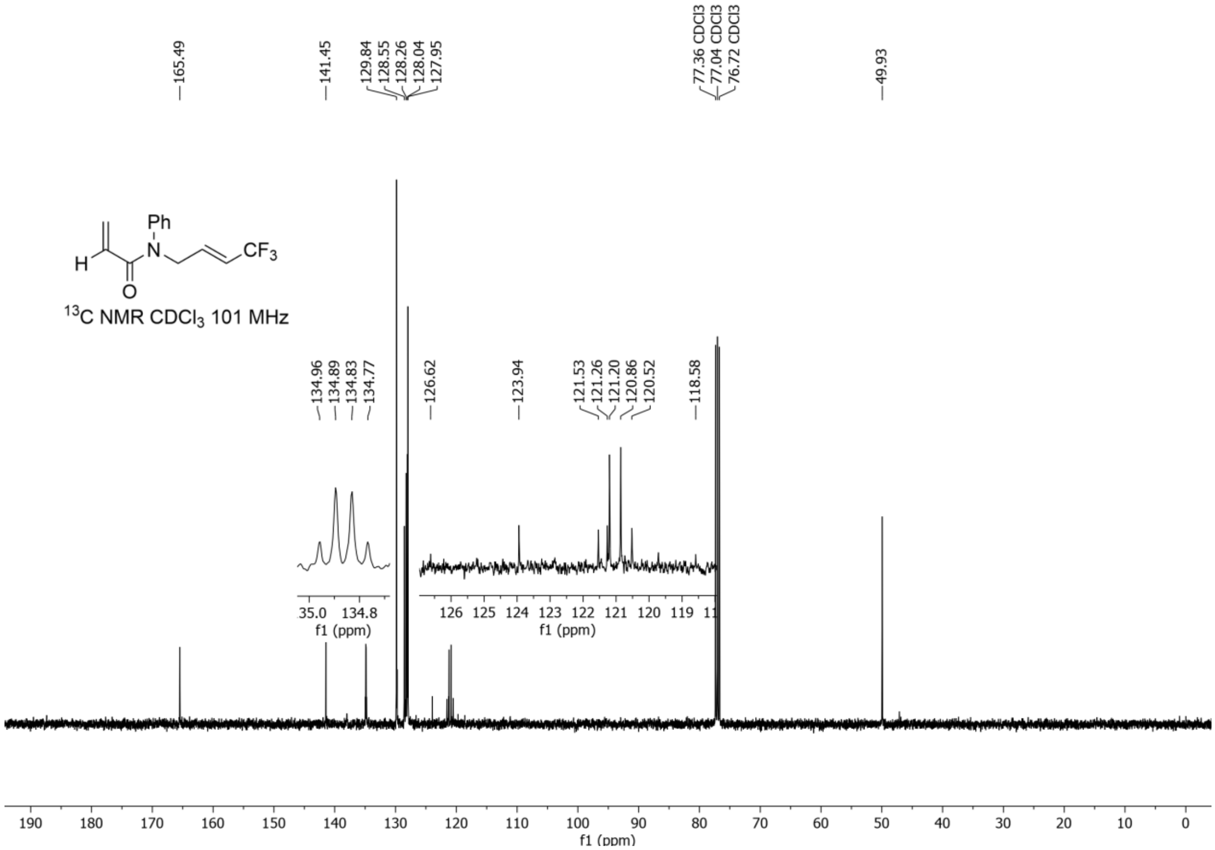

165.49
141.45
129.84
128.55
128.26
128.04
127.95
77.36 CDCl3
77.04 CDCl3
76.72 CDCl3
49.93
Ph
N
H
O
CF3
13C NMR CDCl3 101 MHz
134.96
134.89
134.83
134.77
126.62
123.94
121.53
121.26
121.20
120.86
120.52
118.58
35.0
134.8
f1 (ppm)
126 125 124 123 122 121 120 119
f1 (ppm)
190 180 170 160 150 140 130 120 110 100 90 80 70 60 50 40 30 20 10 0
f1 (ppm)

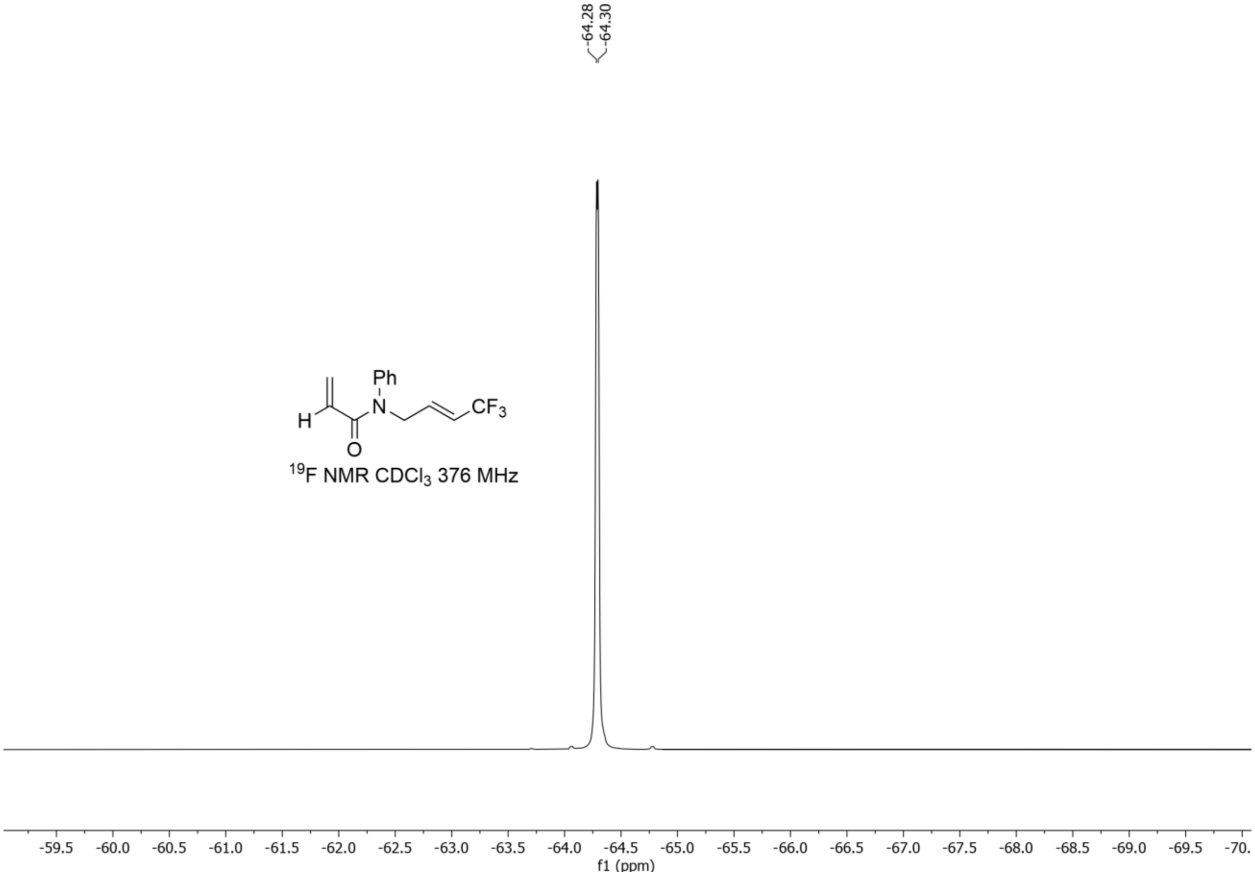

-64.28
-64.30
Ph
N
H
O
CF3
19F NMR CDCl3 376 MHz
-59.5 -60.0 -60.5 -61.0 -61.5 -62.0 -62.5 -63.0 -63.5 -64.0 -64.5 -65.0 -65.5 -66.0 -66.5 -67.0 -67.5 -68.0 -68.5 -69.0 -69.5
f1 (ppm)

(*E*)-*N*-phenyl-*N*-(4,4,4-trifluorobut-2-en-1-yl)methacrylamide (2o)

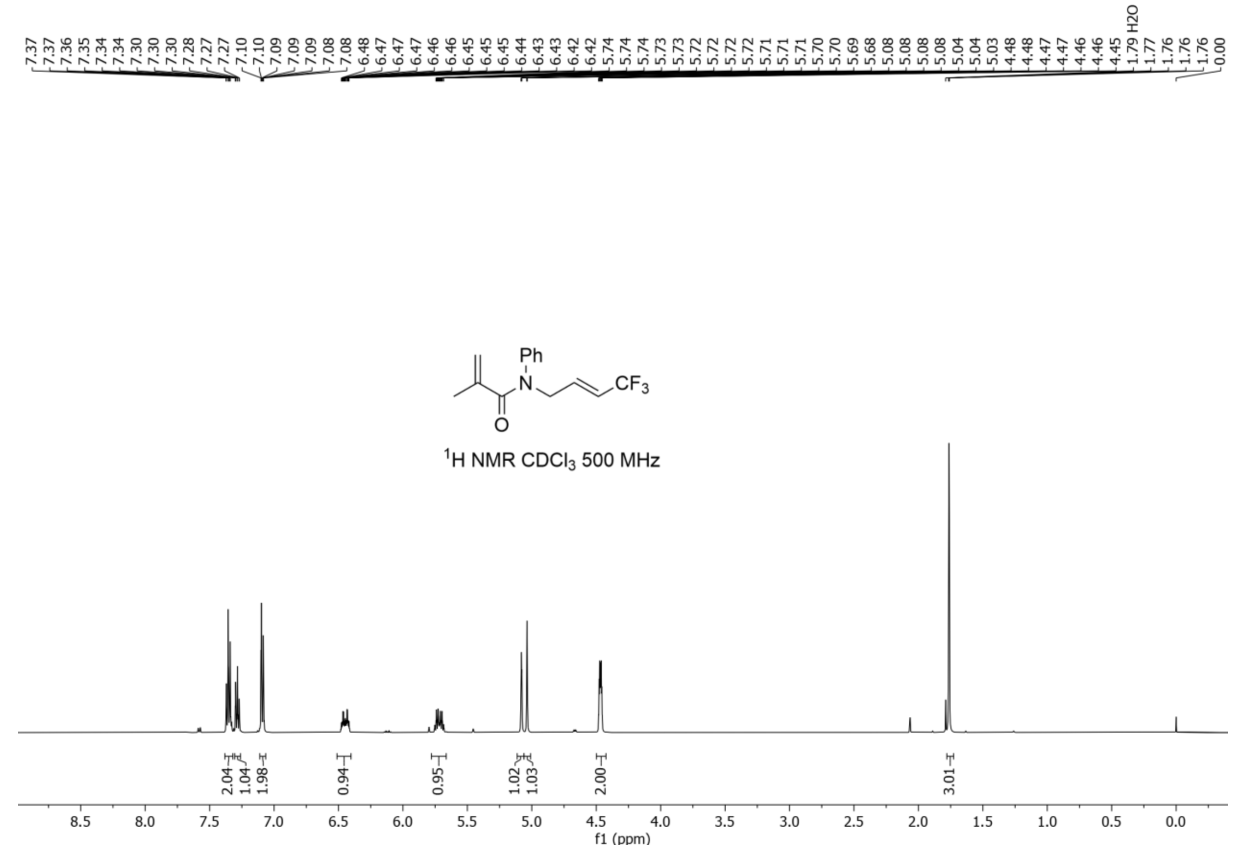

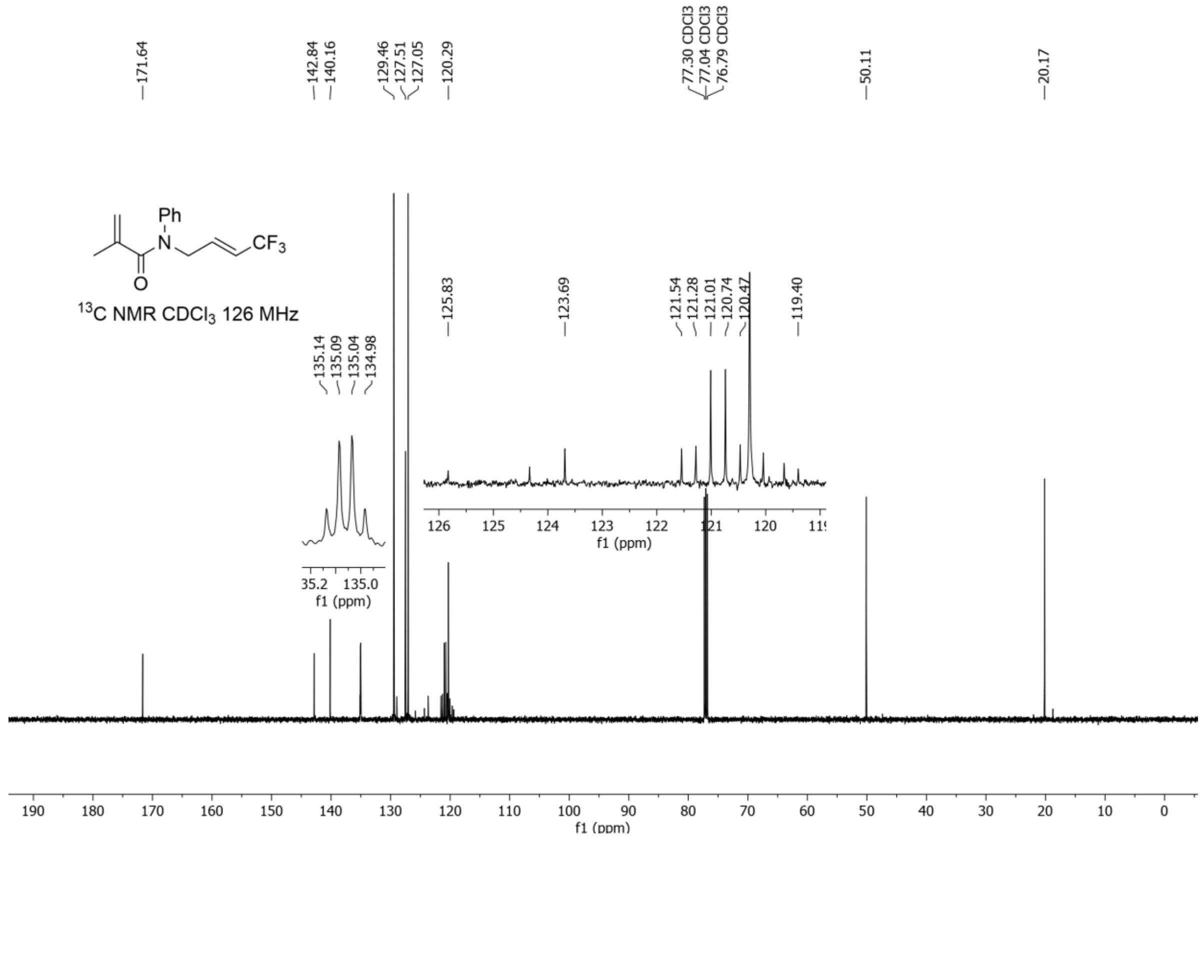

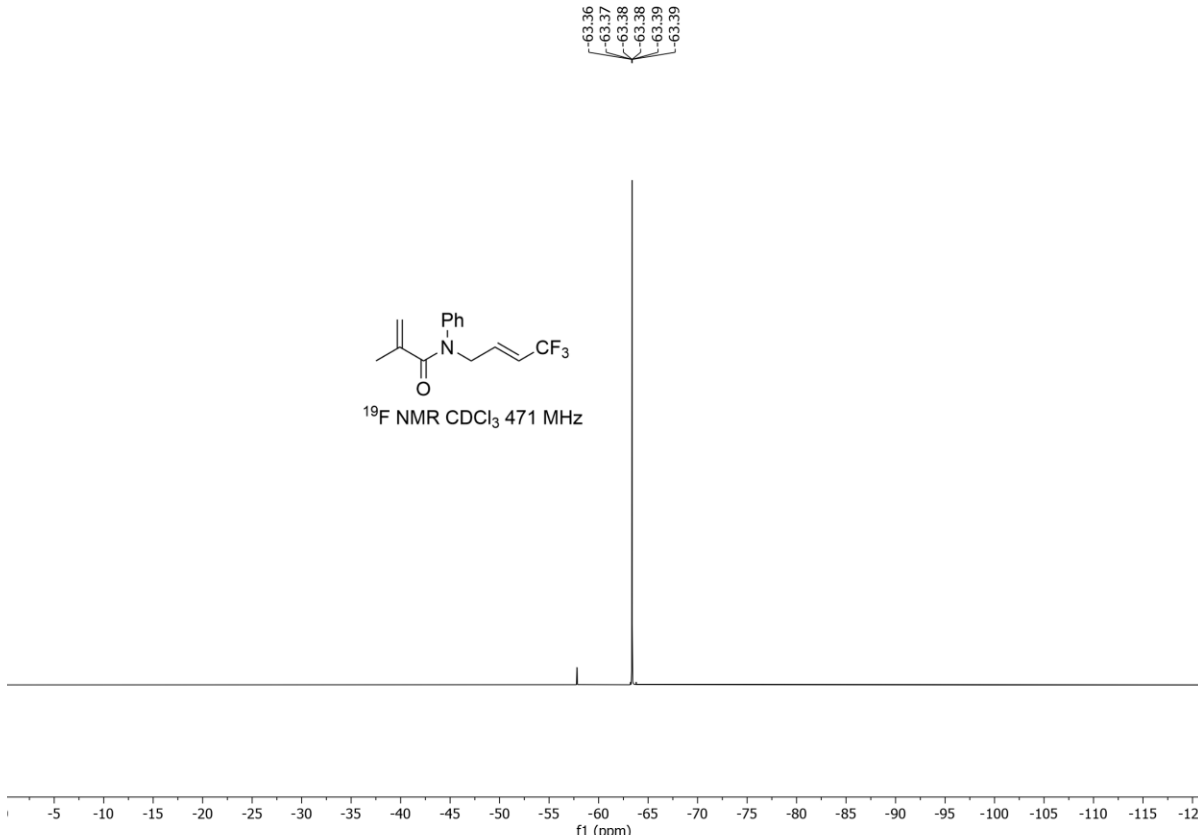

(*E*)-2-benzyl-*N*-phenyl-*N*-(4,4,4-trifluorobut-2-en-1-yl)acrylamide (2p)

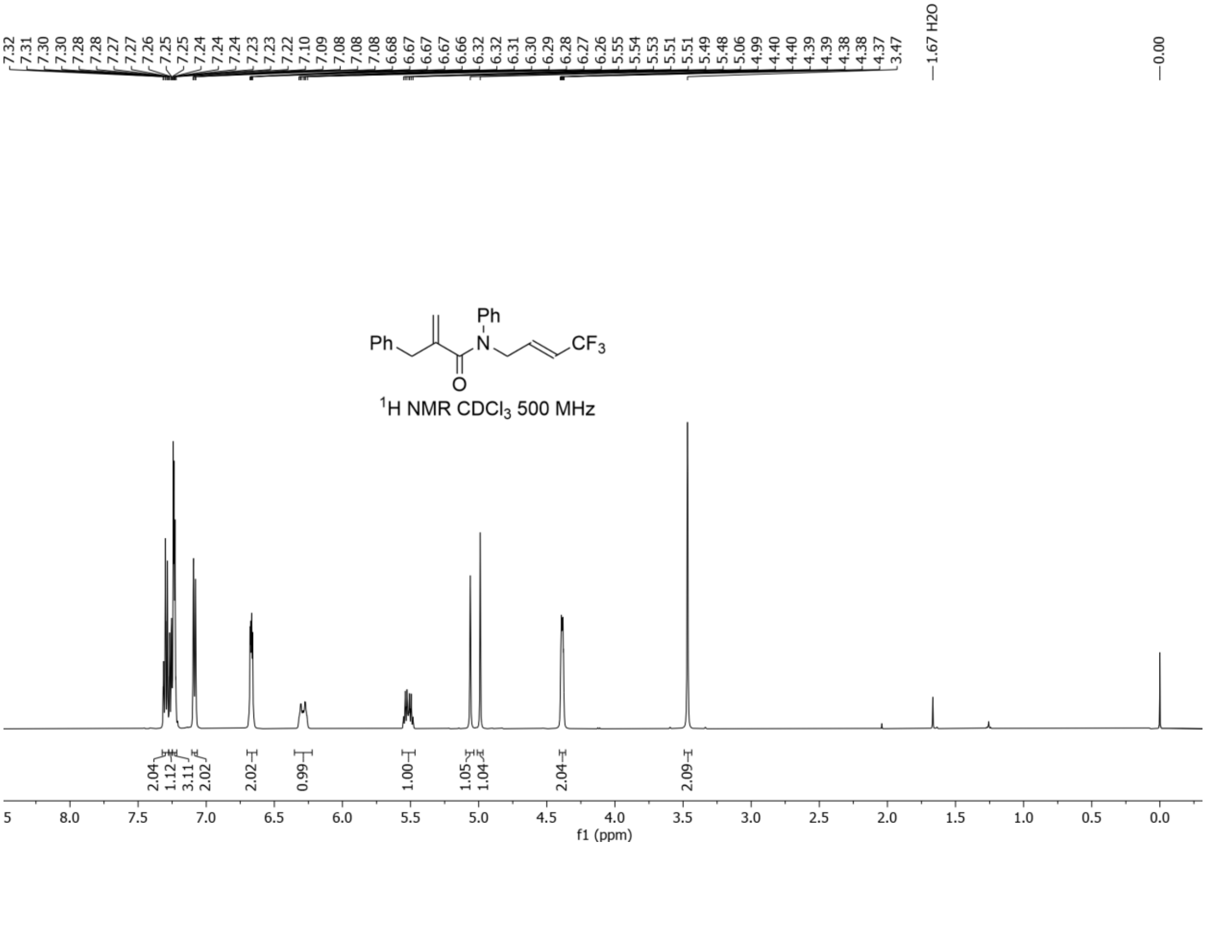

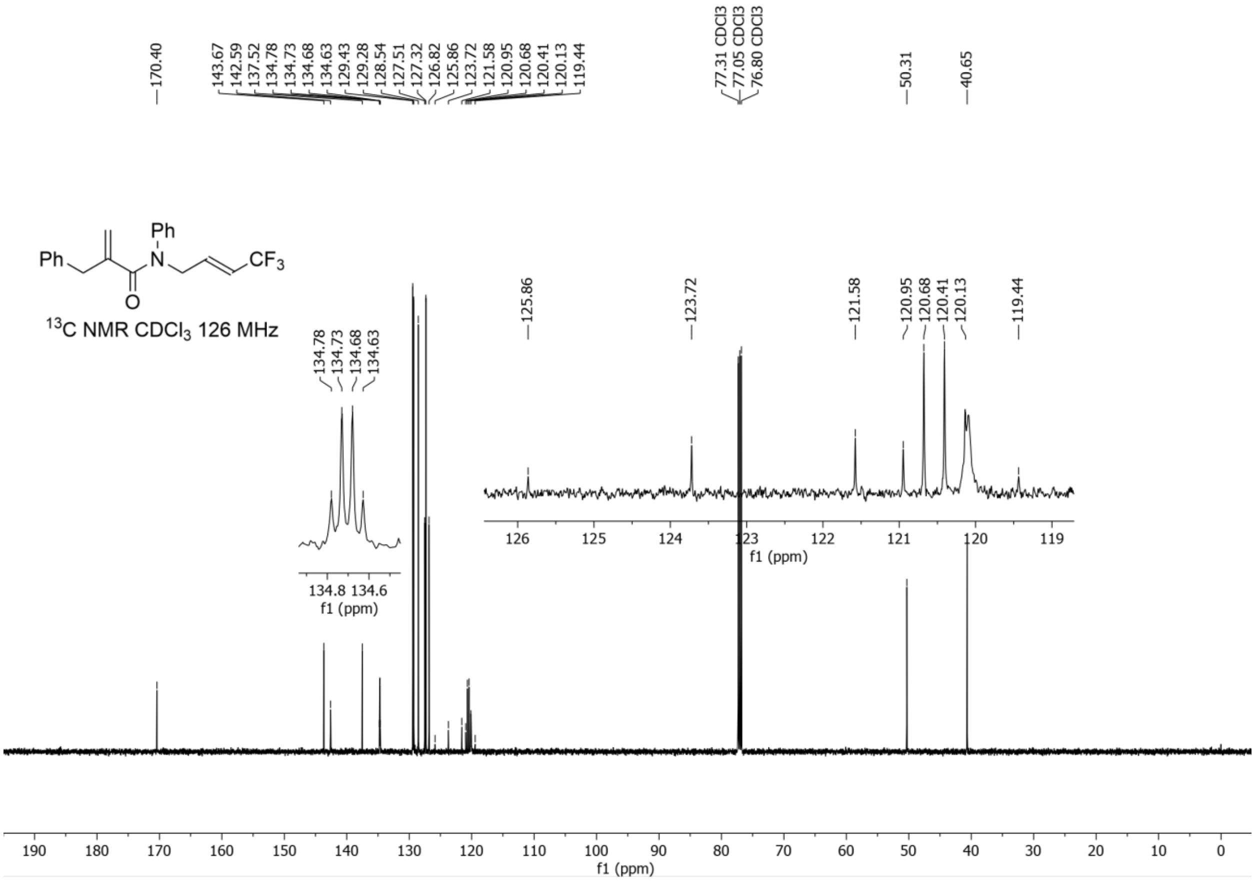

170.40
143.67
142.59
137.52
134.78
134.73
134.68
134.63
129.43
129.28
128.54
127.51
127.32
126.82
125.86
123.72
121.58
120.95
120.68
120.41
120.13
119.44
77.31 CDCl3
77.05 CDCl3
76.80 CDCl3
50.31
40.65
Ph
Ph
N
O
CF3
13C NMR CDCl3 126 MHz
f1 (ppm)

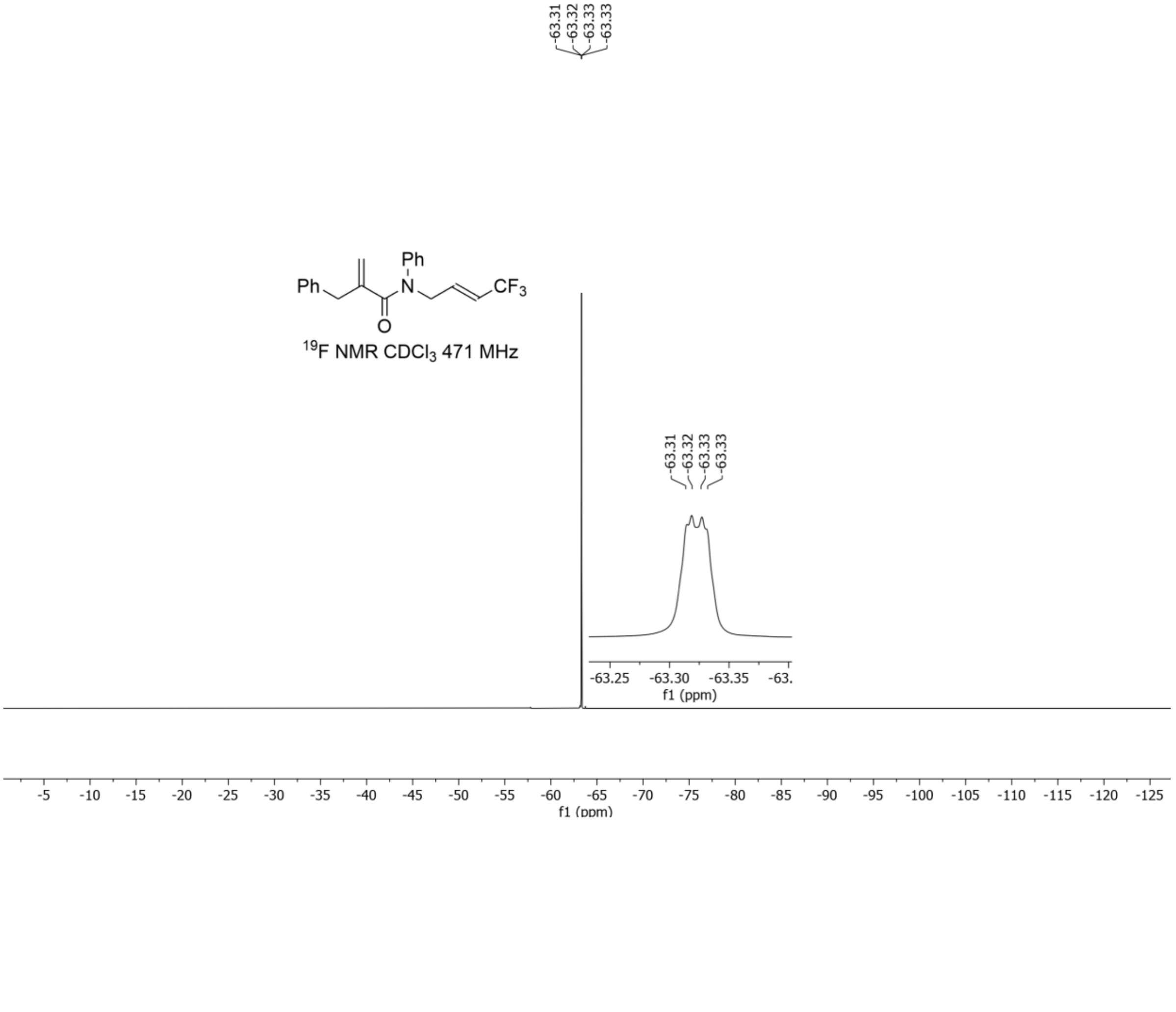

-63.31
-63.32
-63.33
-63.33
Ph
Ph
N
O
CF3
19F NMR CDCl3 471 MHz
f1 (ppm)

(*E*)-2-methylene-*N*-phenyl-*N*-(4,4,4-trifluorobut-2-en-1-yl)pentanamide (2q)

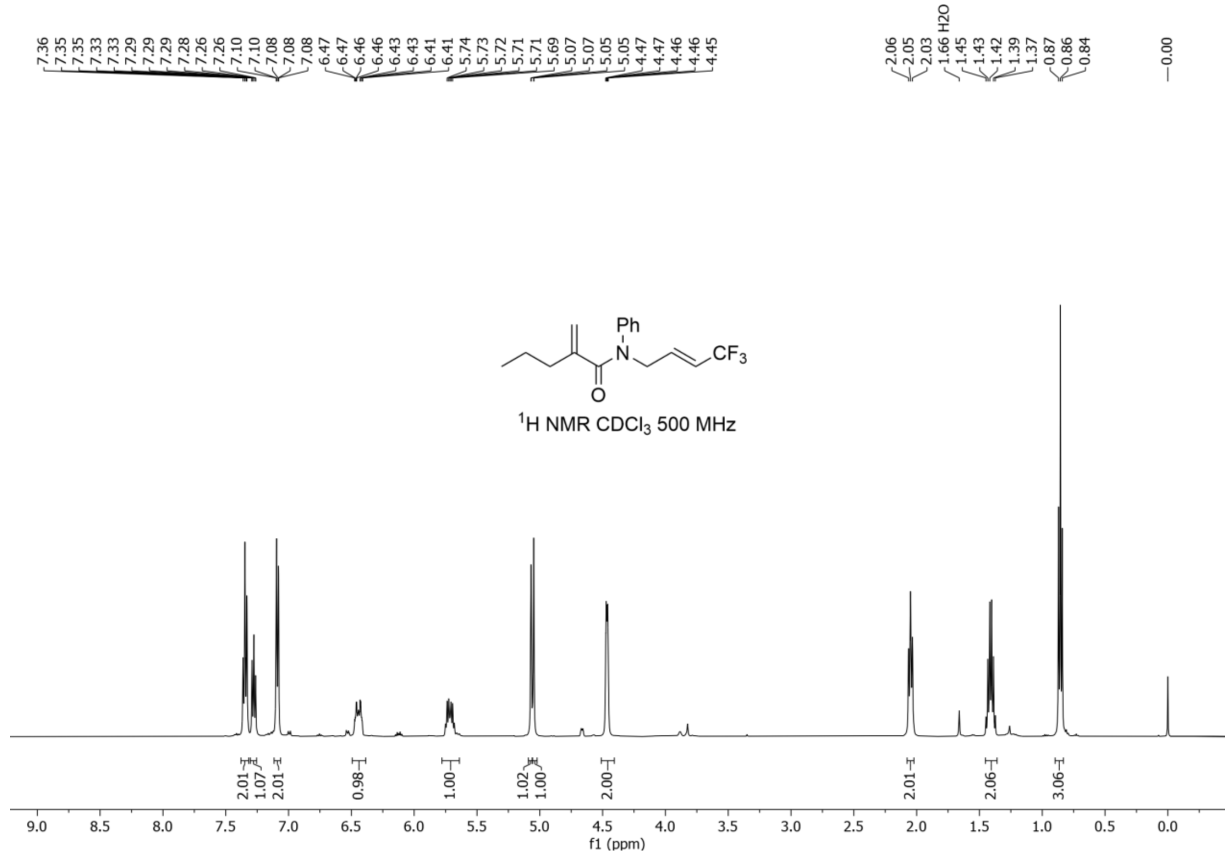

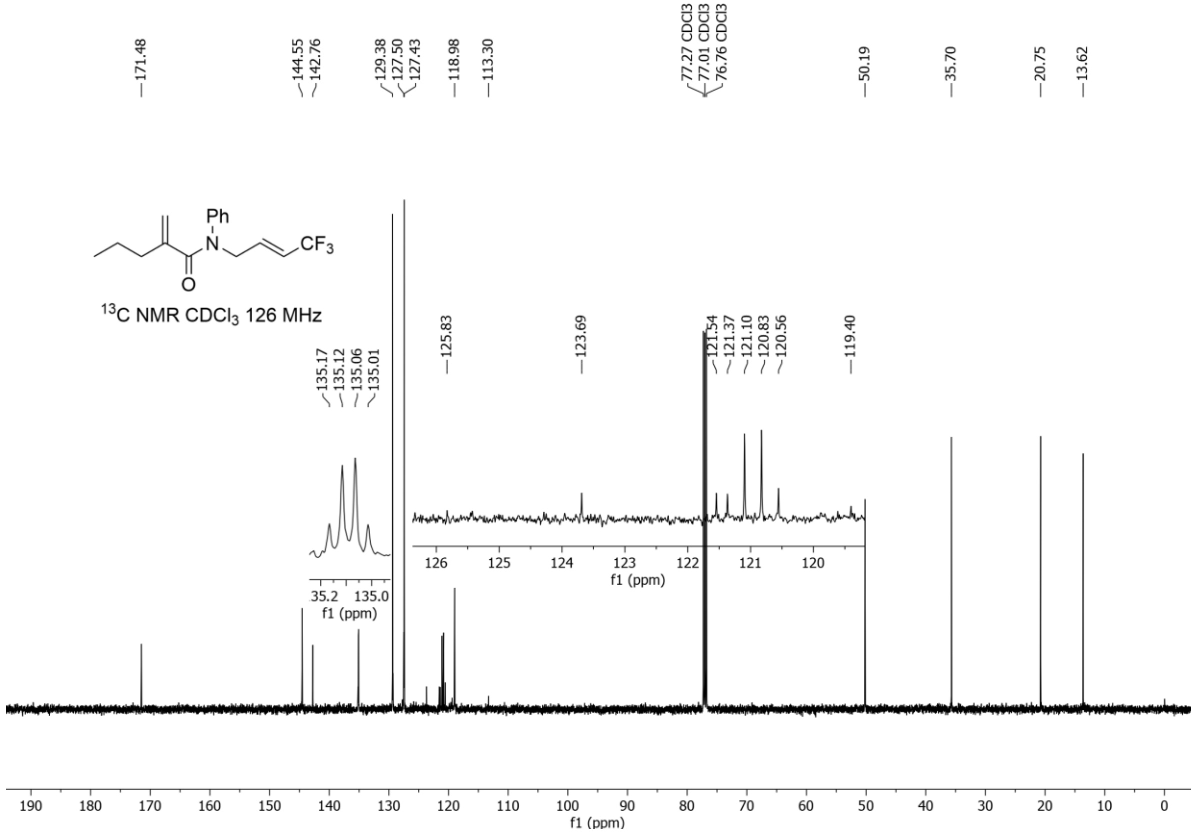

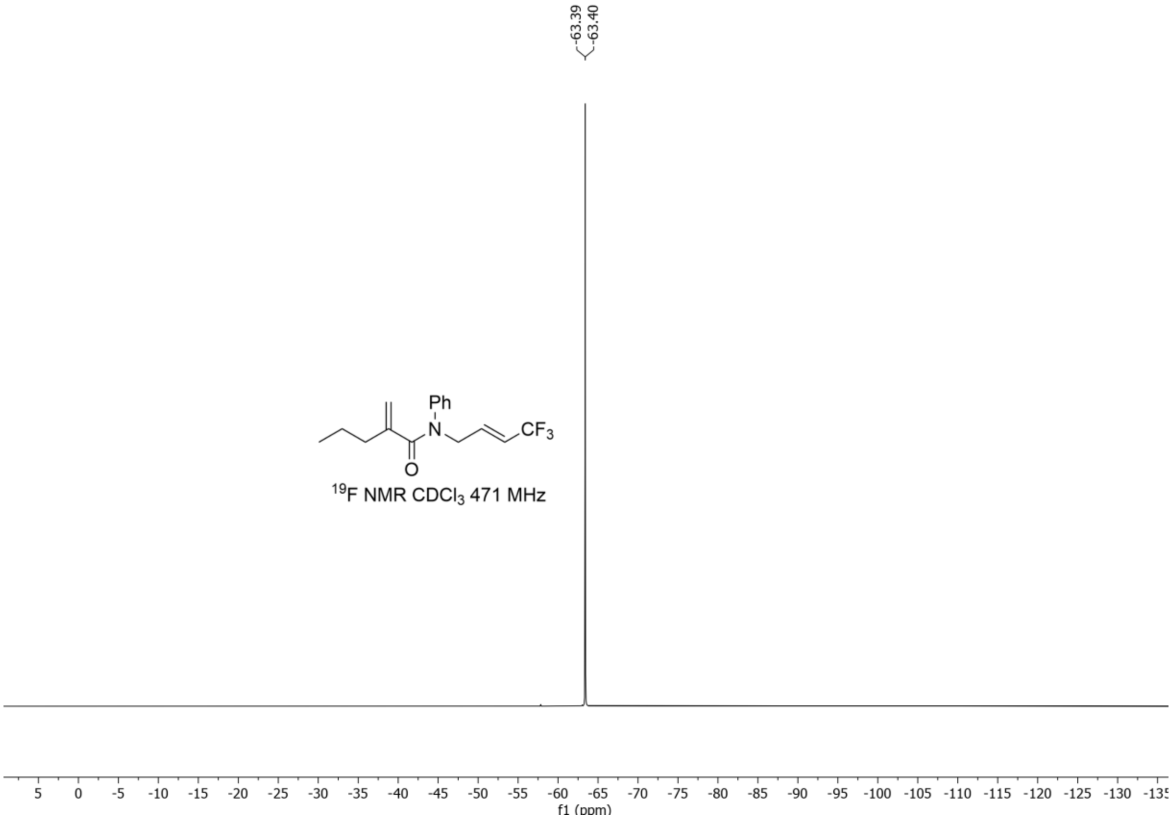

(*E*)-4-methyl-2-methylene-*N*-phenyl-*N*-(4,4,4-trifluorobut-2-en-1-yl)pentanamide (2r)

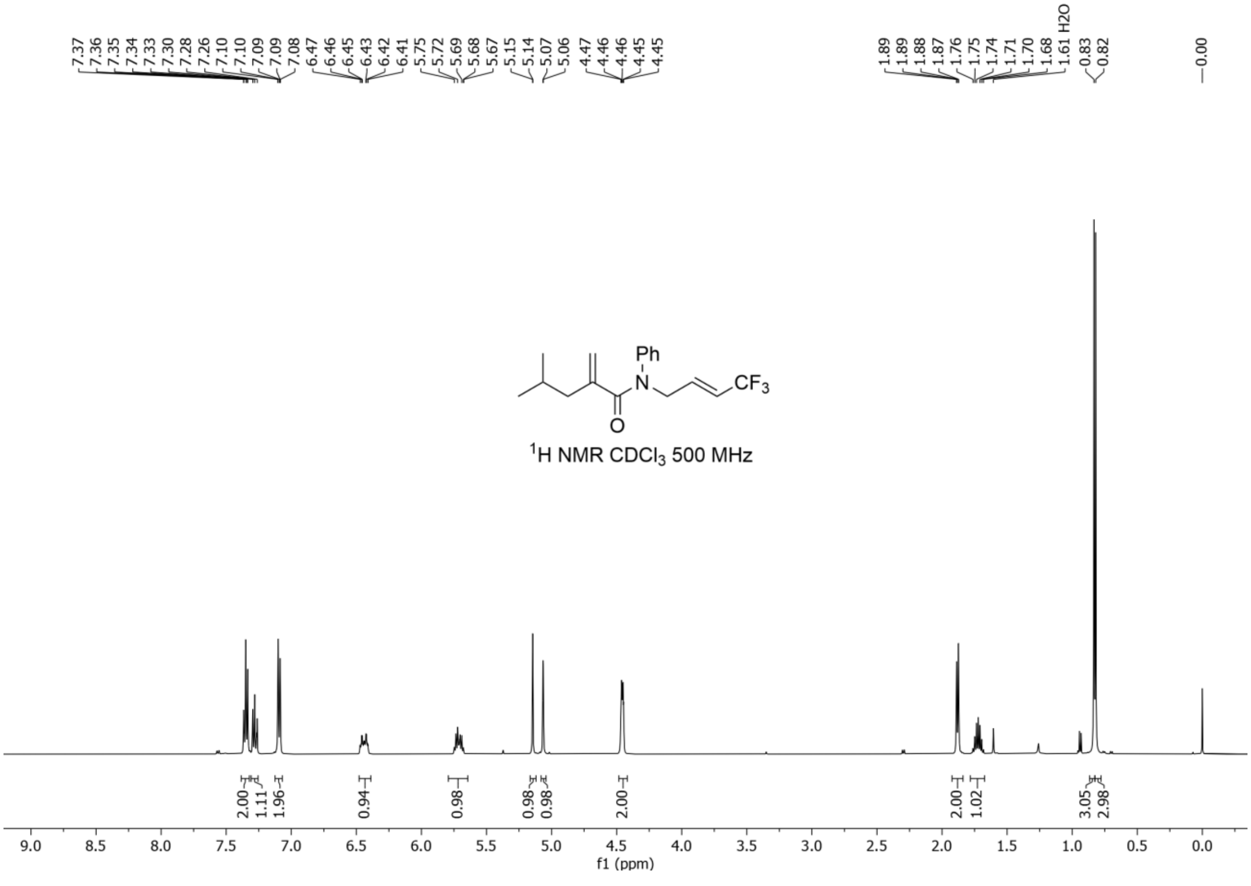

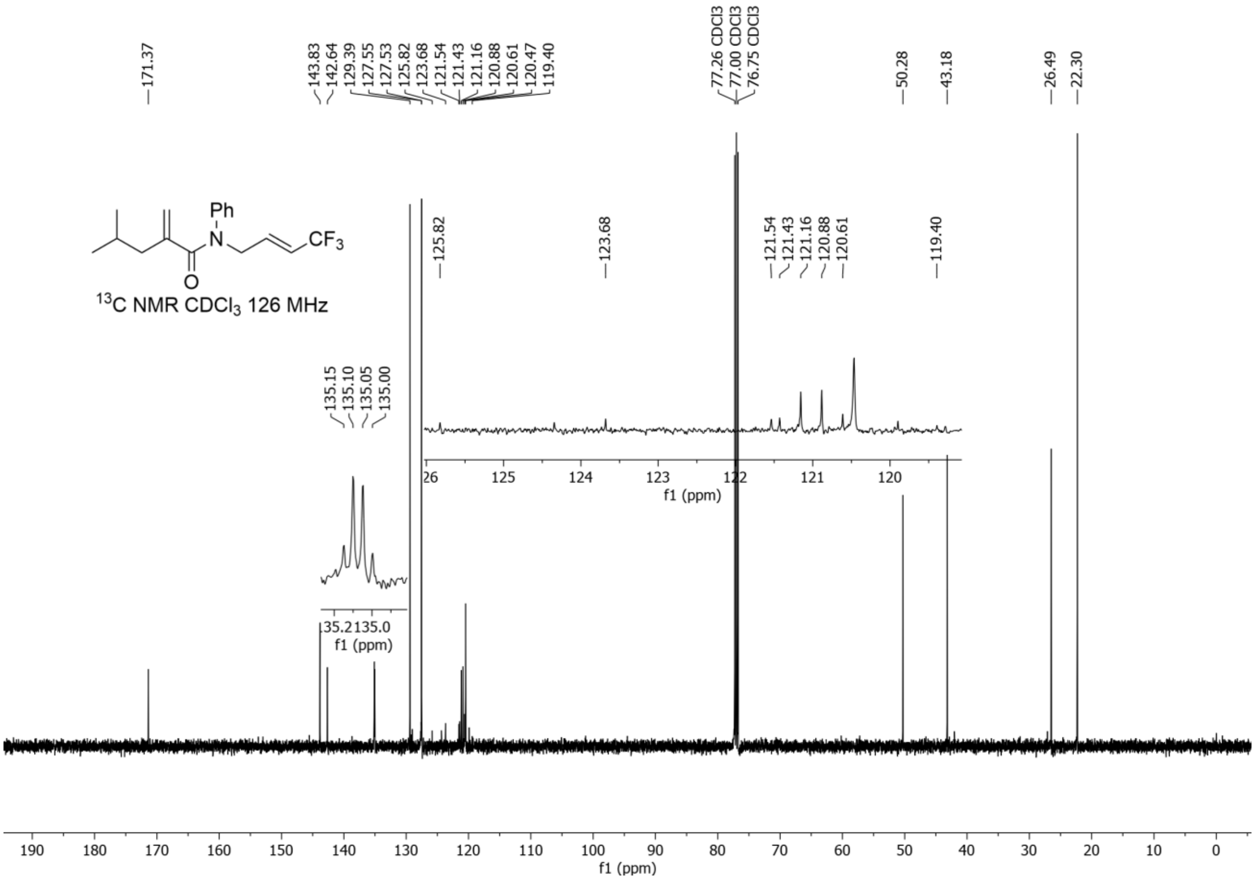
171.37
143.83
142.64
129.39
127.55
127.53
125.82
123.68
121.54
121.43
121.16
120.88
120.61
120.47
119.40
77.26 CDCl3
77.00 CDCl3
76.75 CDCl3
50.28
43.18
26.49
22.30
Ph
N
CF3
O
13C NMR CDCl3 126 MHz
135.15
135.10
135.05
135.00
f1 (ppm)
f1 (ppm)
f1 (ppm)

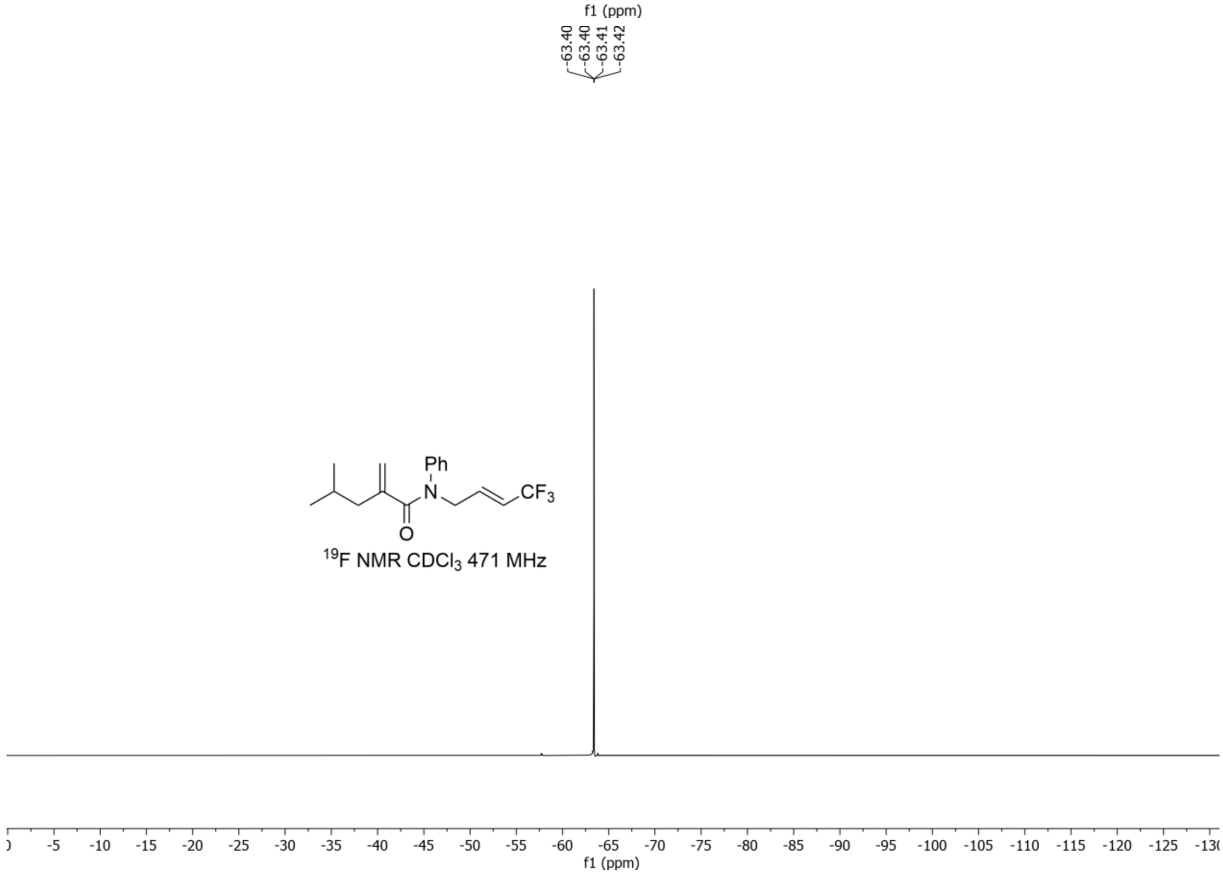
-63.40
-63.40
-63.41
-63.42
Ph
N
CF3
O
19F NMR CDCl3 471 MHz
f1 (ppm)

(*E*)-2-cyclopropyl-*N*-phenyl-N-(4,4,4-trifluorobut-2-en-1-yl)acrylamide (2s)

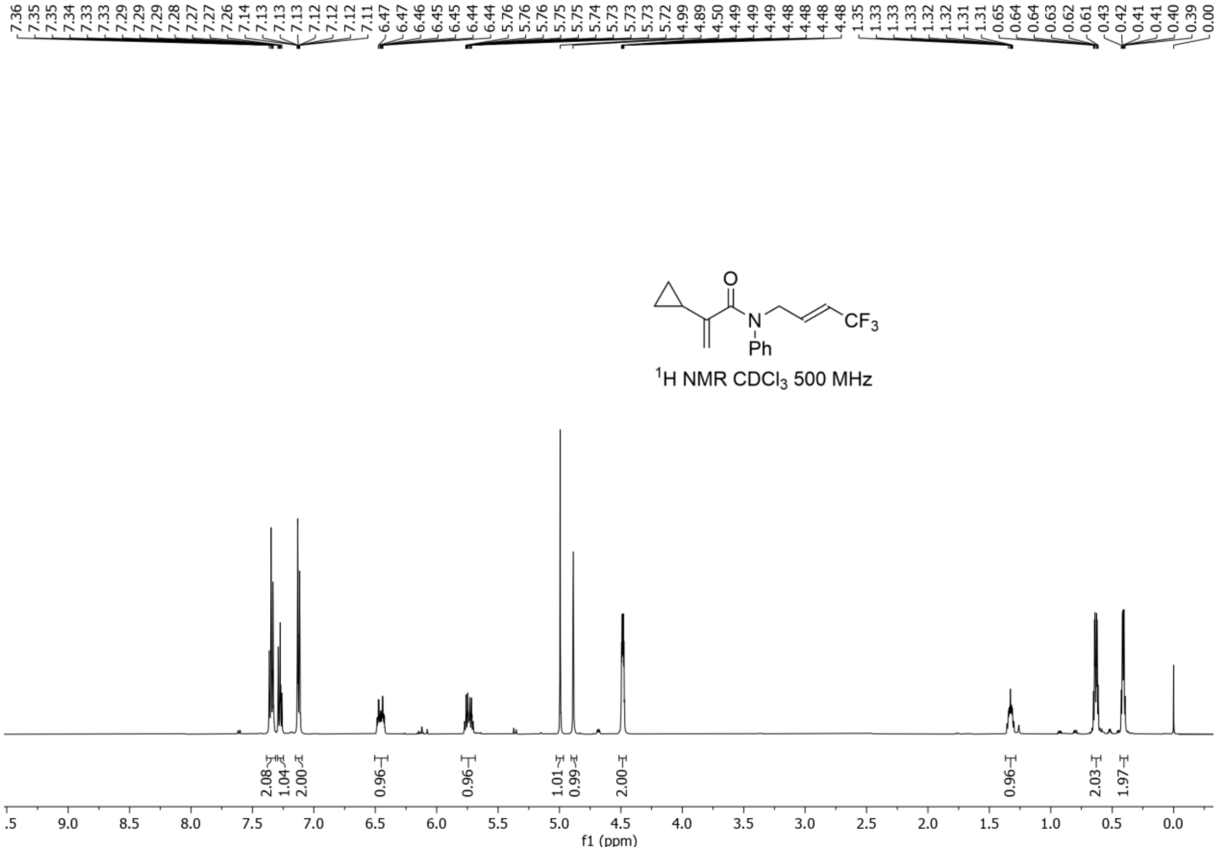

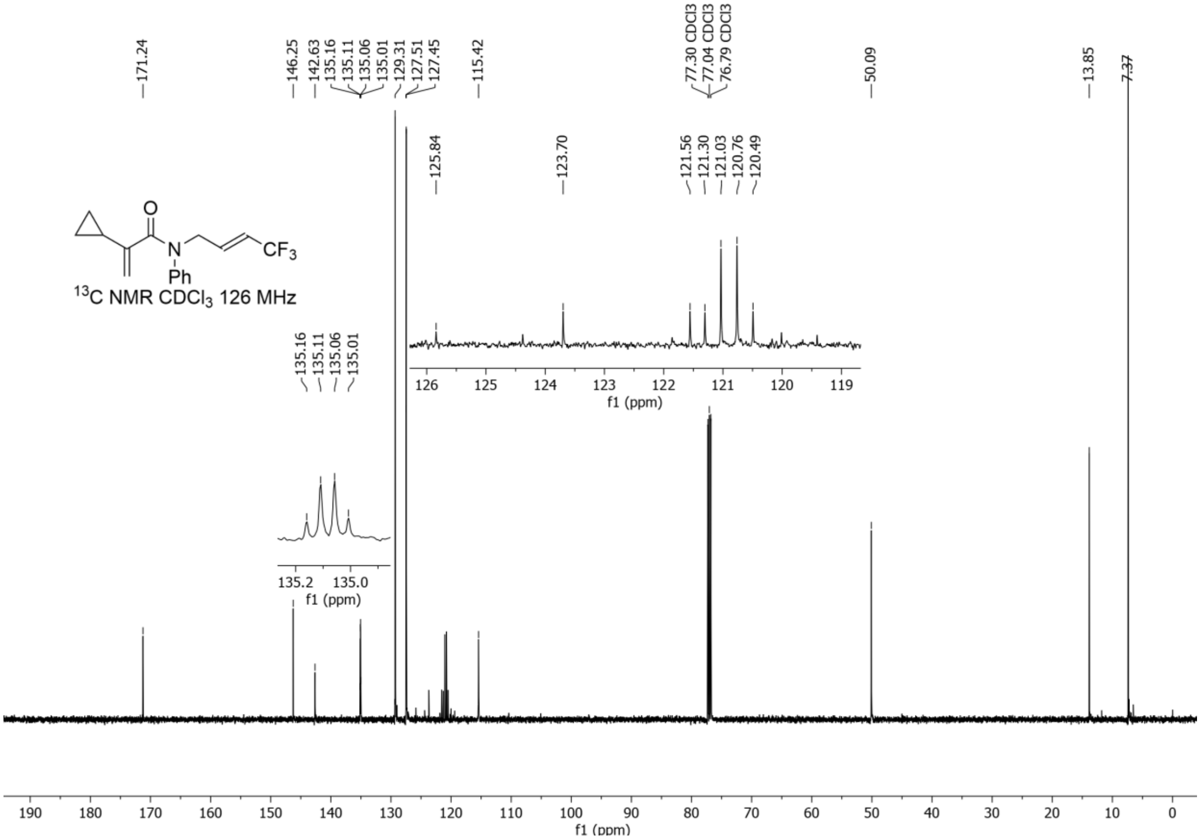

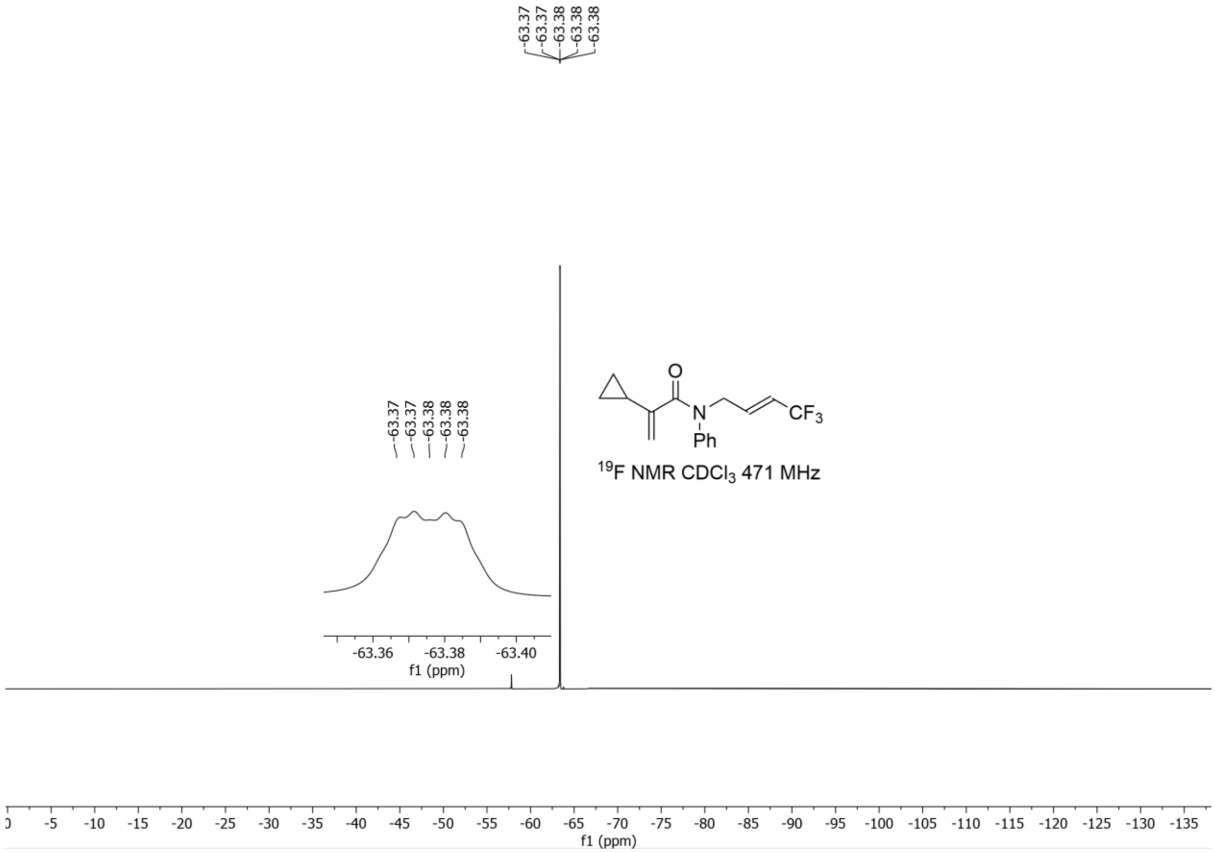

(*E*)-2-cyclopentyl-*N*-phenyl-*N*-(4,4,4-trifluorobut-2-en-1-yl)acrylamide (2t)

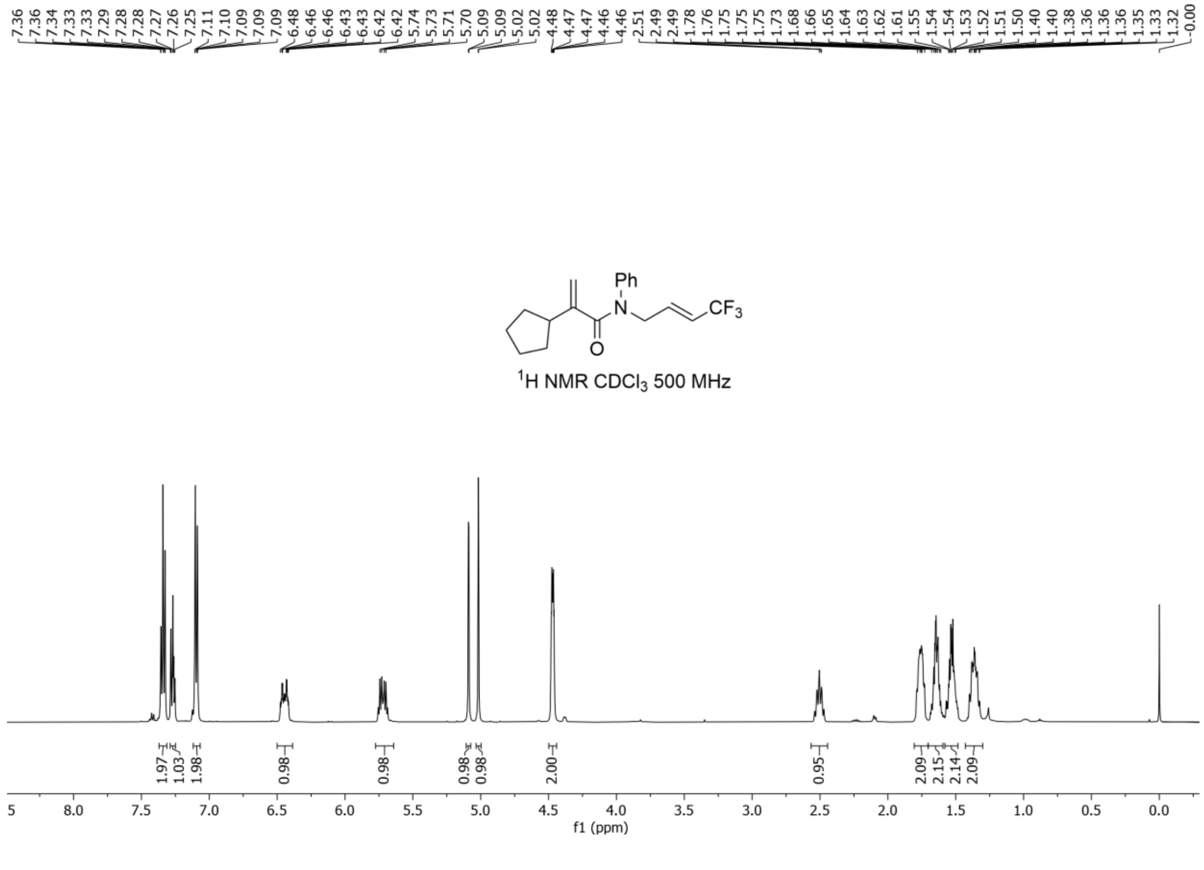

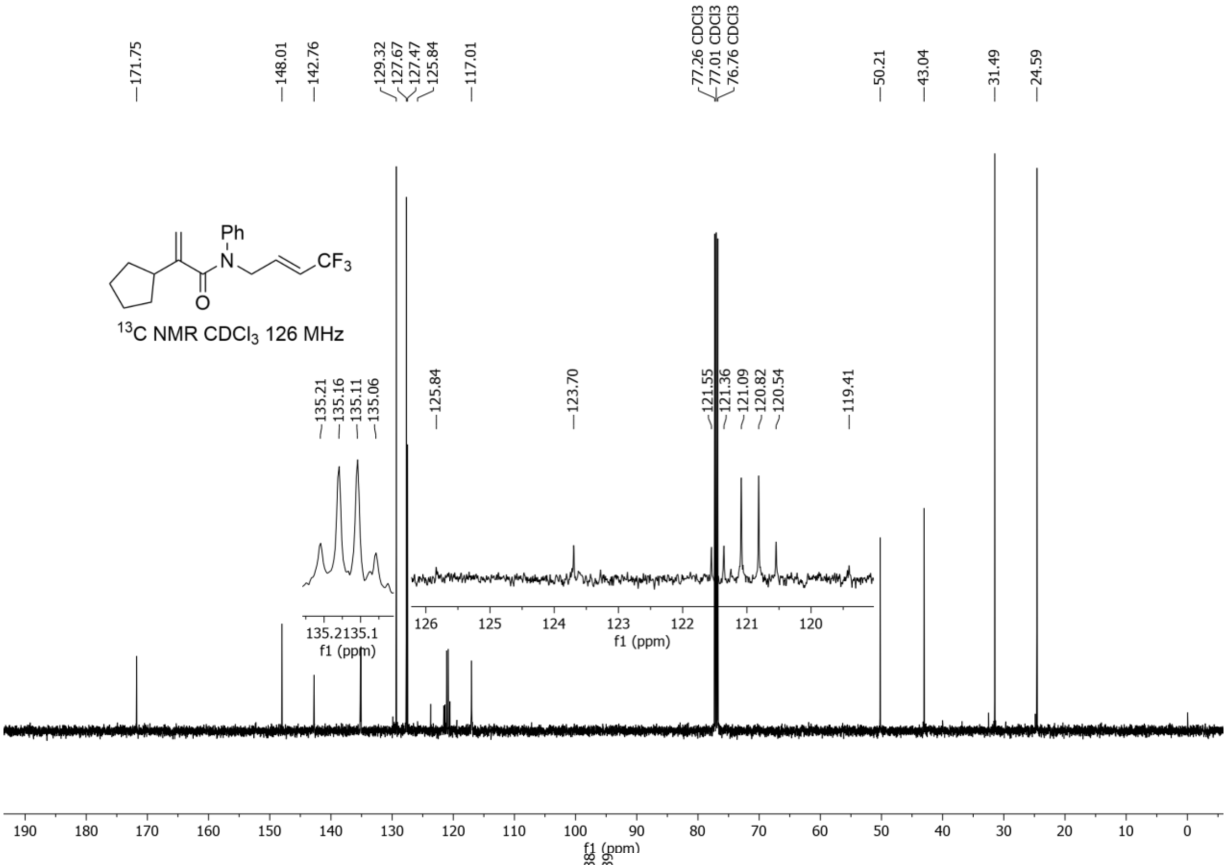

^{13}C NMR $CDCl_3$ 126 MHz

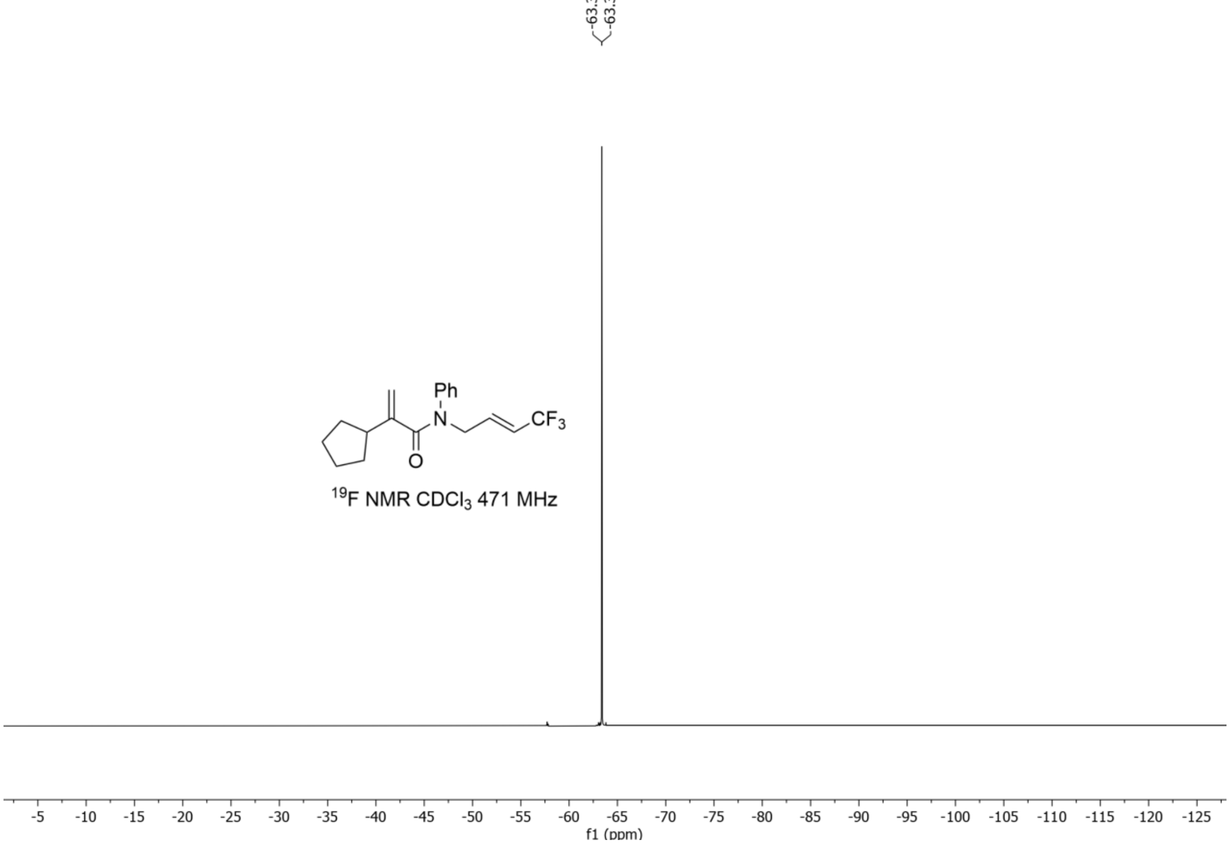

^{19}F NMR $CDCl_3$ 471 MHz

(*E*)-*N*-(4-(*tert*-butyl)phenyl)-2-phenyl-*N*-(4,4,4-trifluorobut-2-en-1-yl)acrylamide (2u)

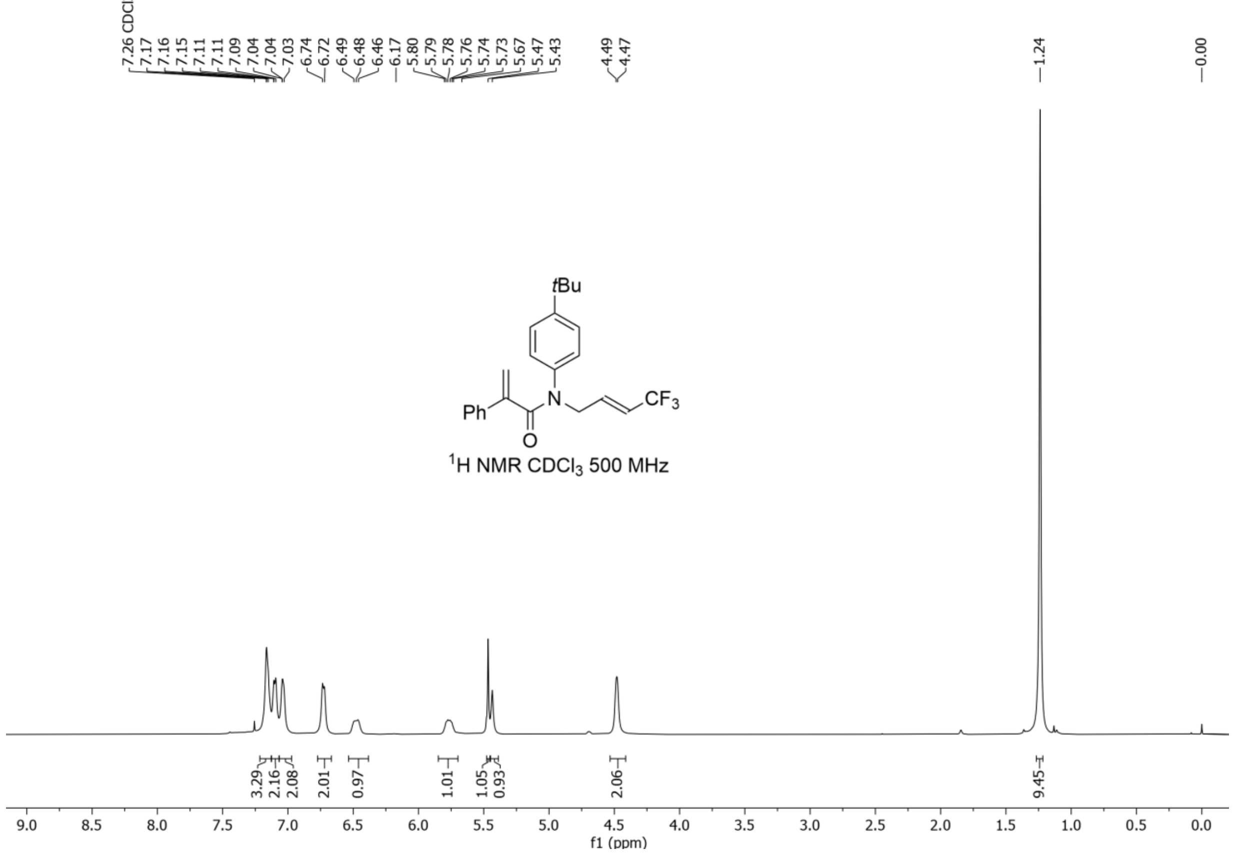

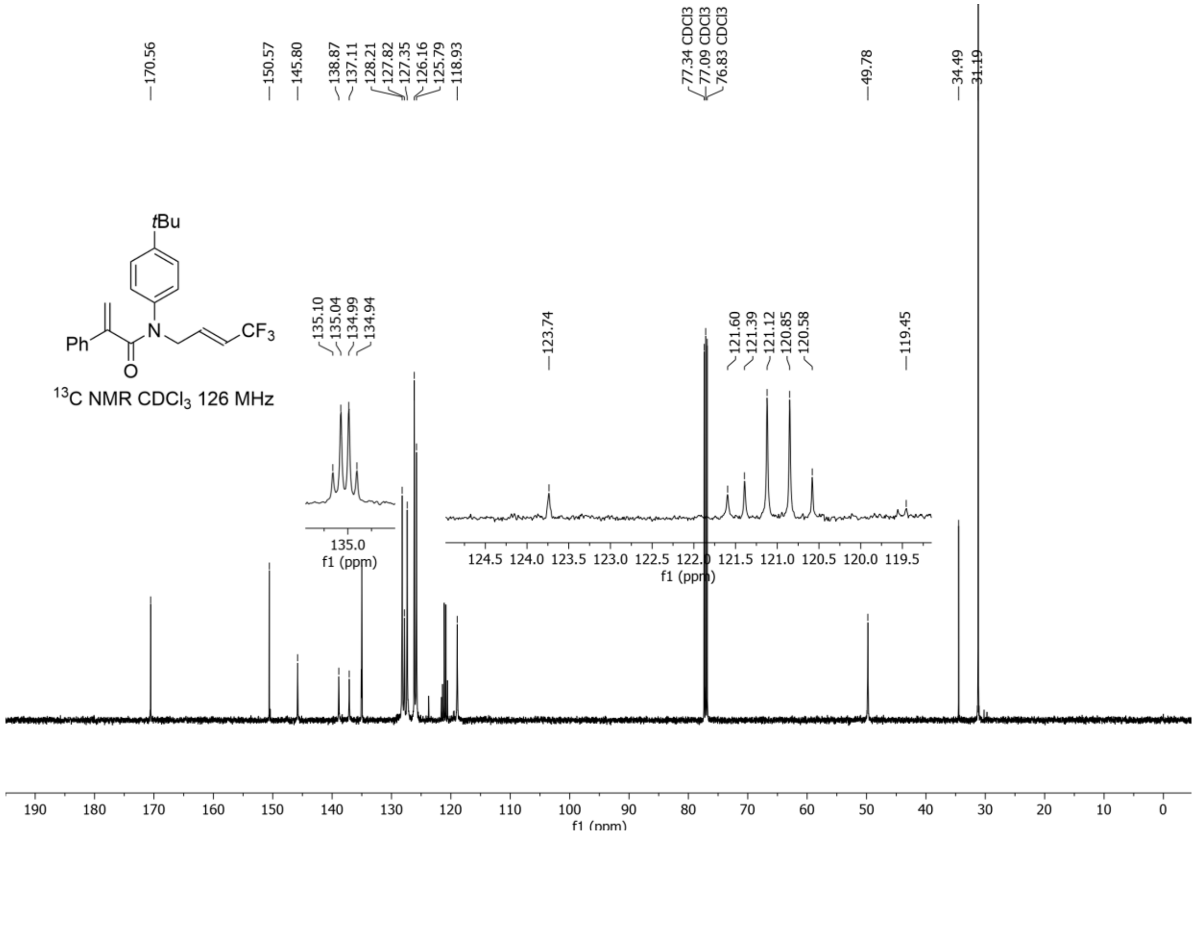

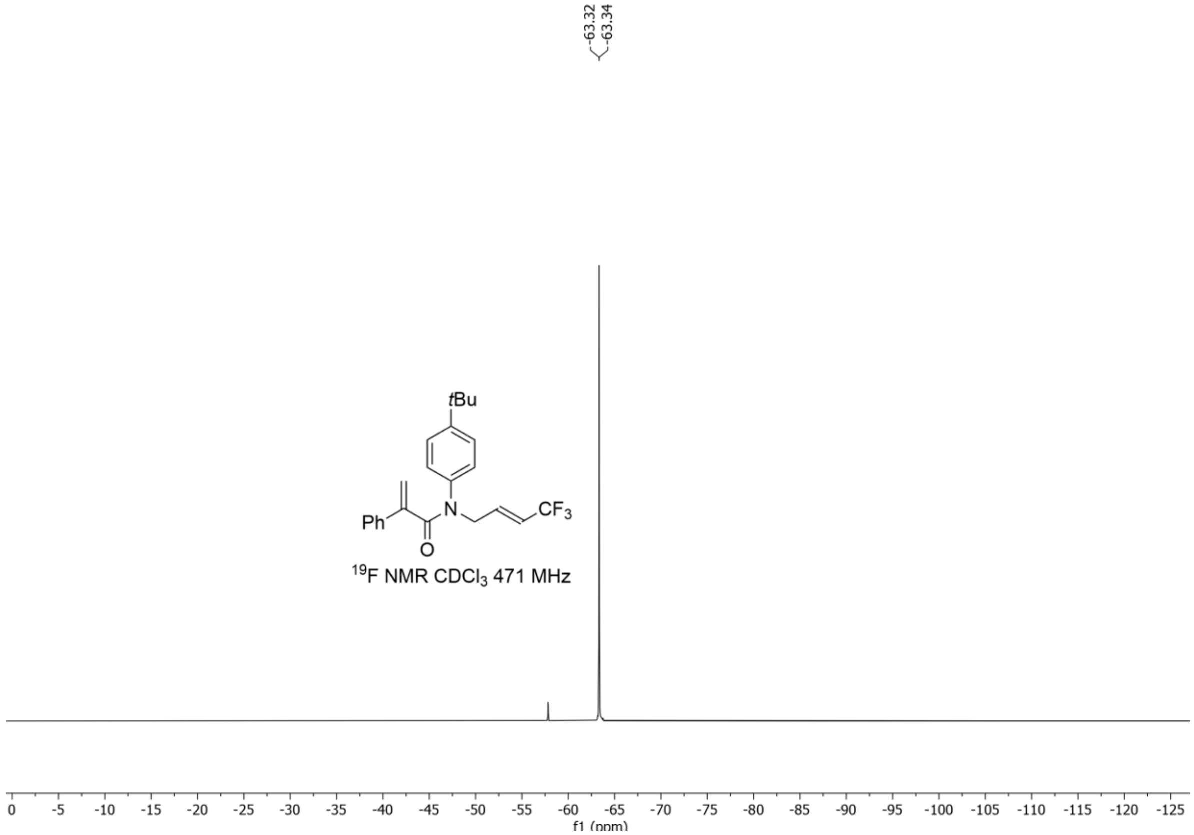

(*E*)-*N*-benzyl-2-phenyl-*N*-(4,4,4-trifluorobut-2-en-1-yl)acrylamide (2v)

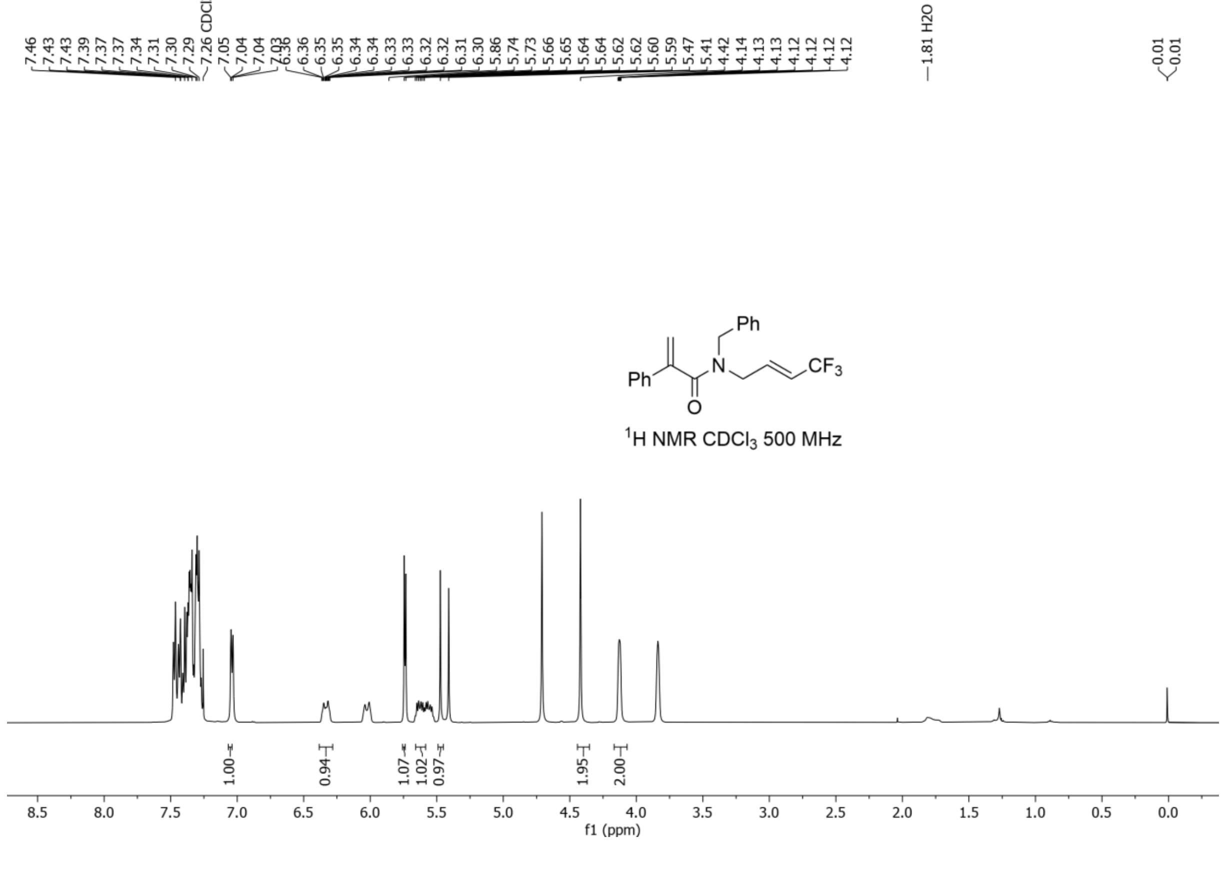

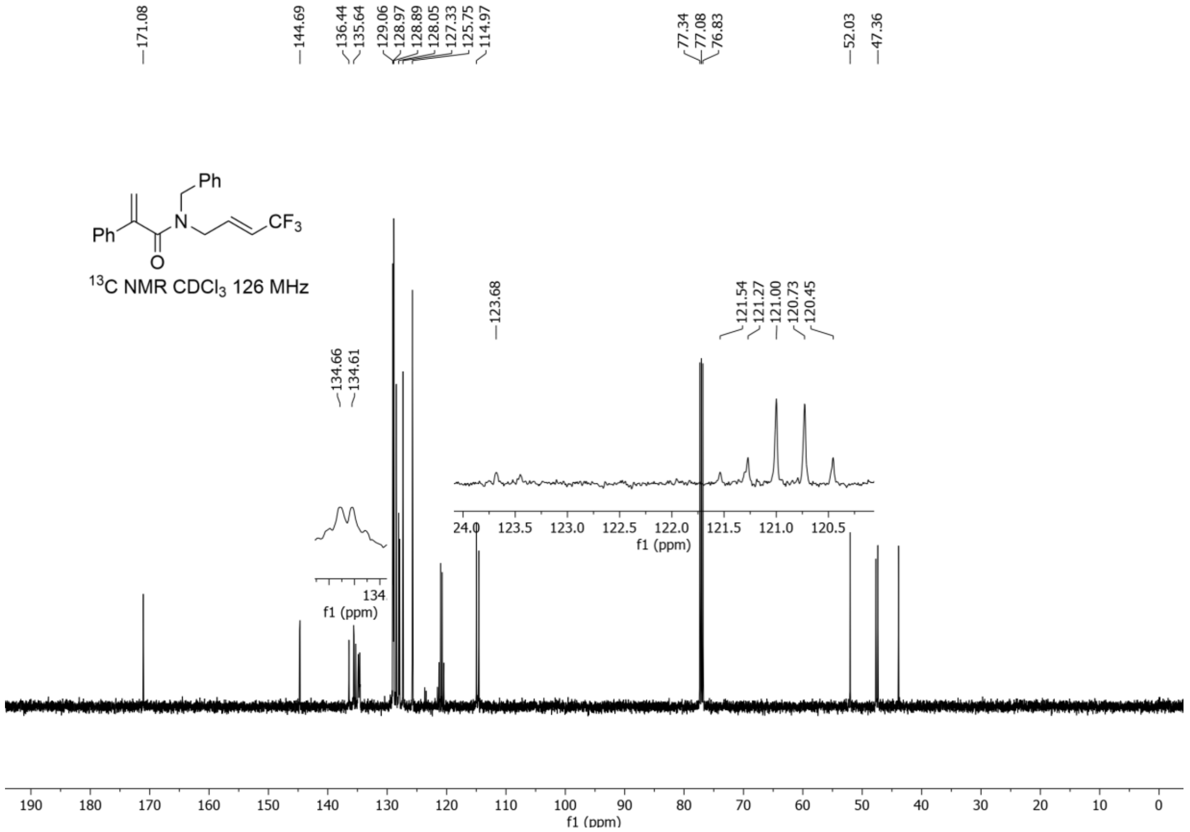
Ph
N
CF3
Ph
O
13C NMR CDCl3 126 MHz
171.08
144.69
136.44
135.64
129.06
128.97
128.89
128.05
127.33
125.75
114.97
77.34
77.08
76.83
52.03
47.36
134.66
134.61
123.68
121.54
121.27
121.00
120.73
120.45
f1 (ppm)

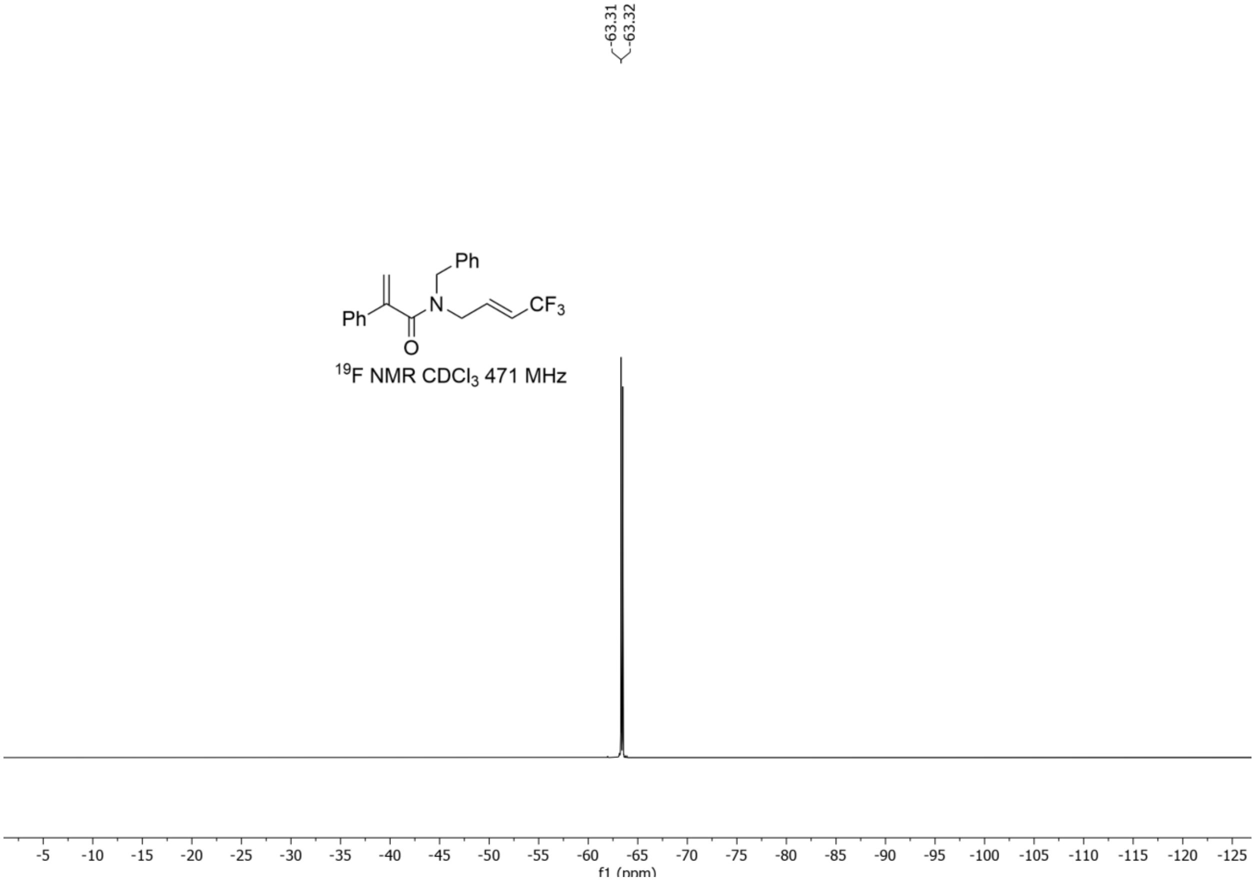
-63.31
-63.32
Ph
N
CF3
Ph
O
19F NMR CDCl3 471 MHz
f1 (ppm)

(*E*)-2-phenyl-*N*-(3-phenylpropyl)-*N*-(4,4,4-trifluorobut-2-en-1-yl)acrylamide (2w)

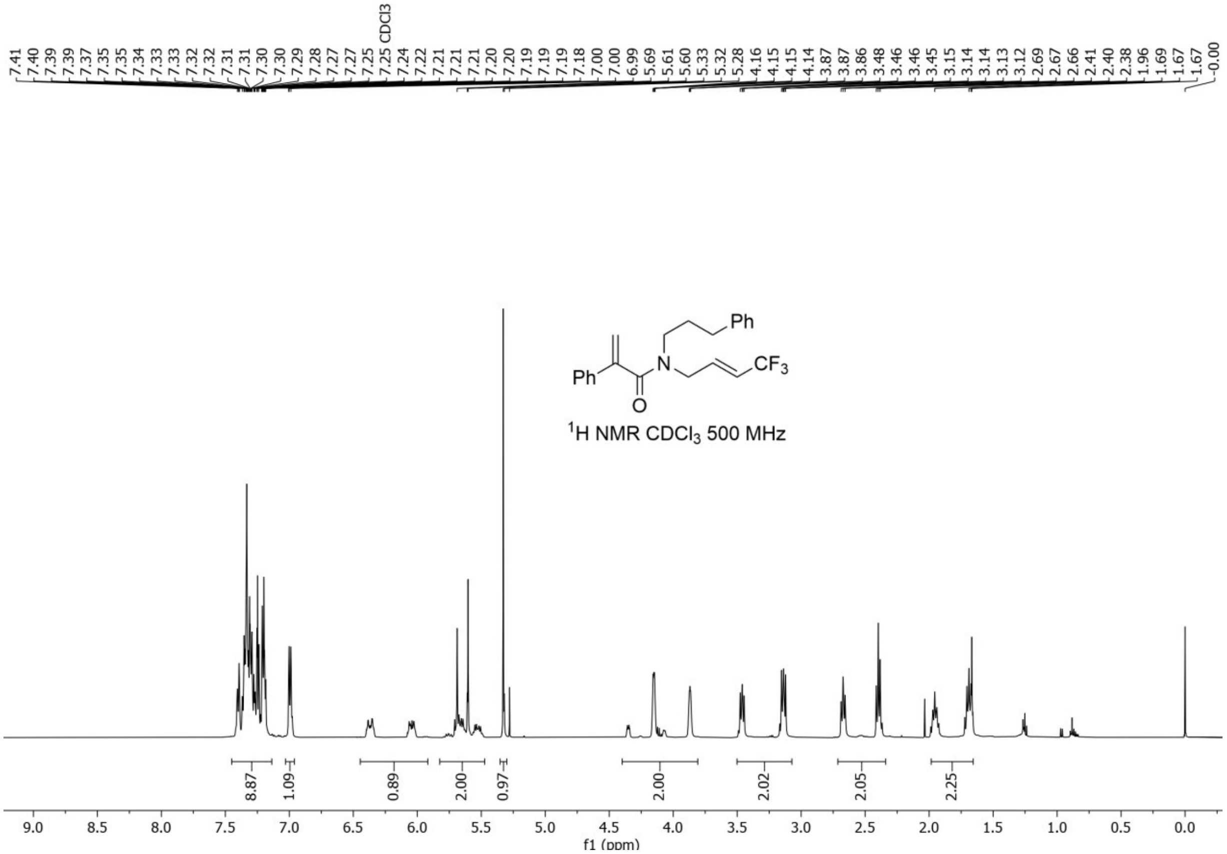

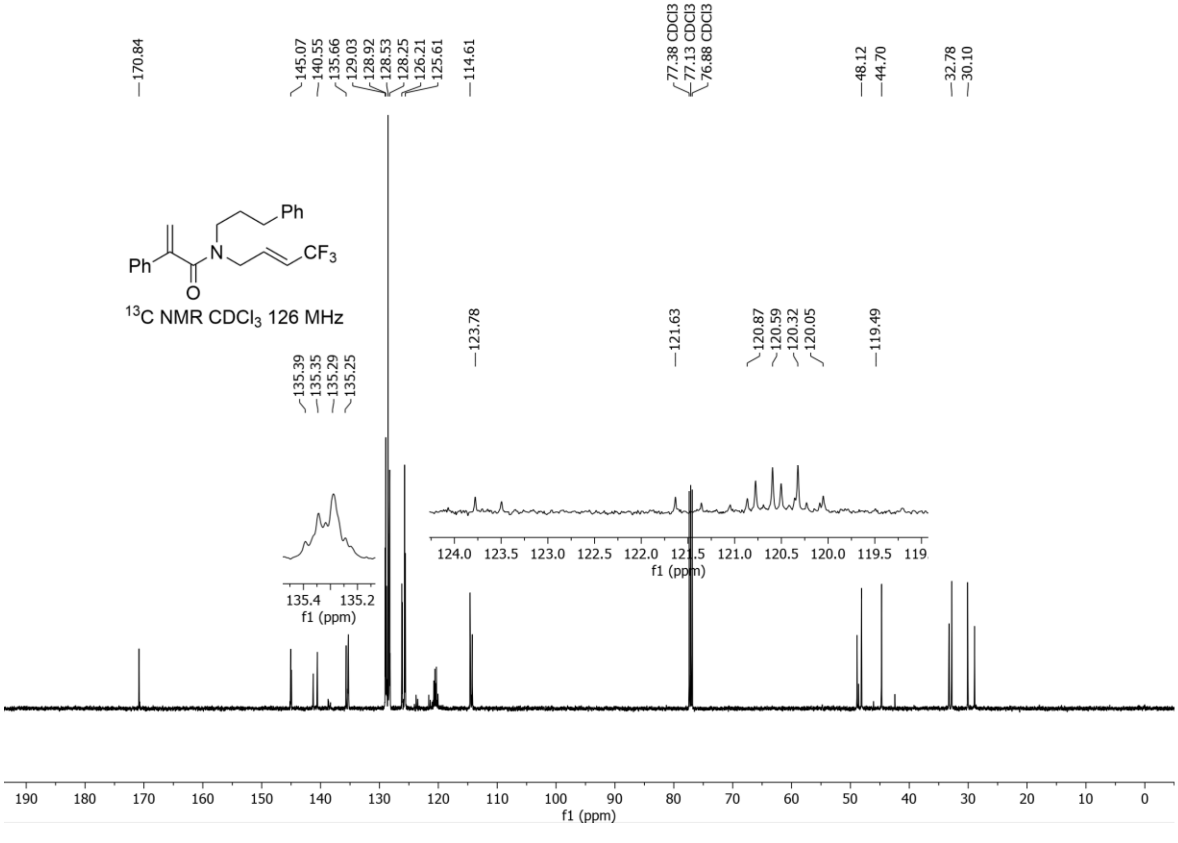

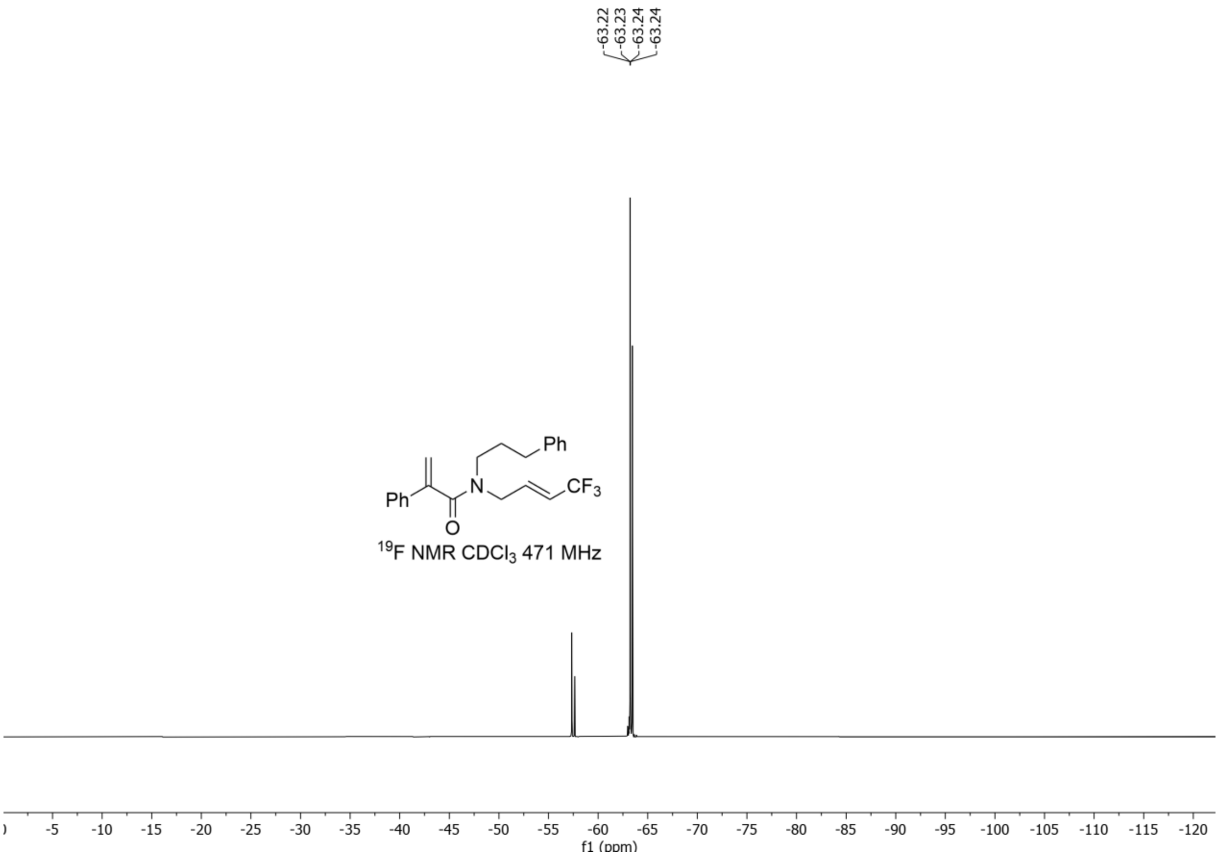

(*E*)-*N*-hexyl-2-phenyl-*N*-(4,4,4-trifluorobut-2-en-1-yl)acrylamide (2x)

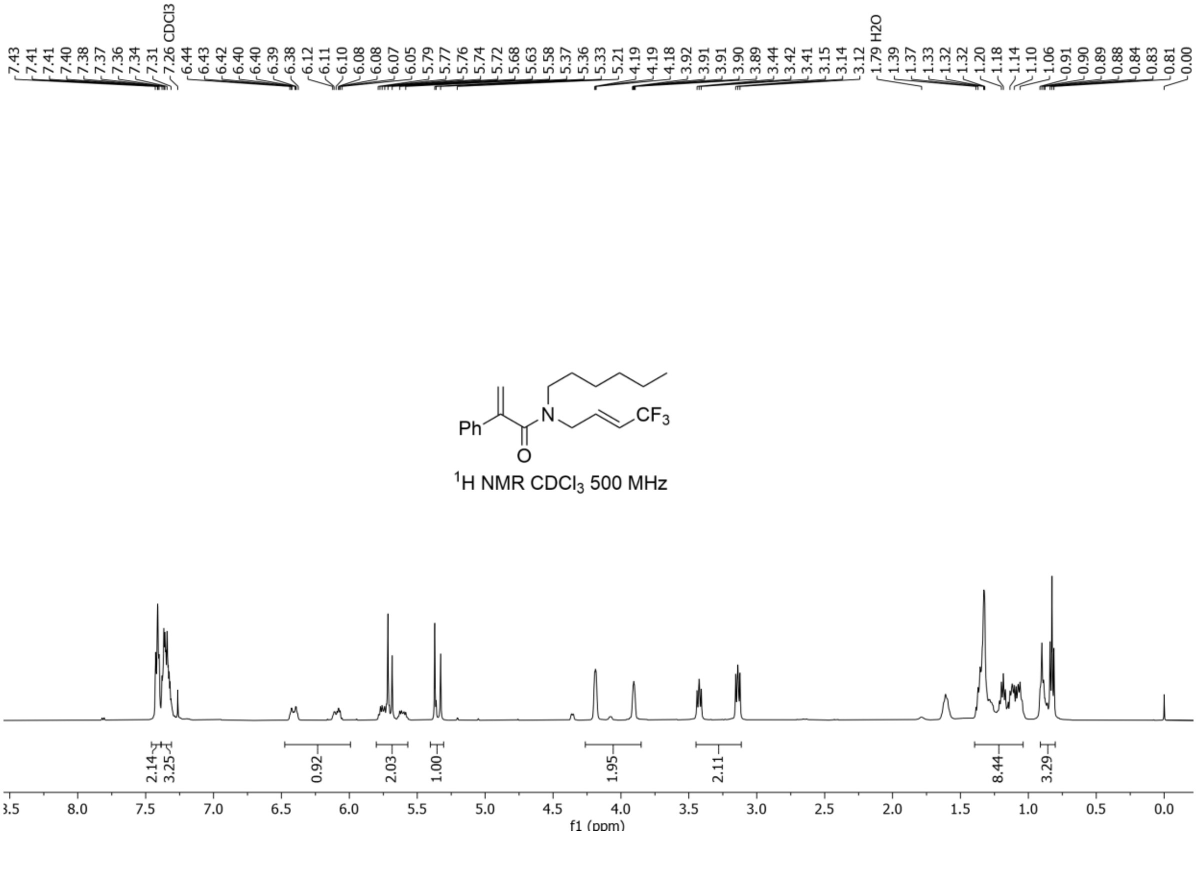

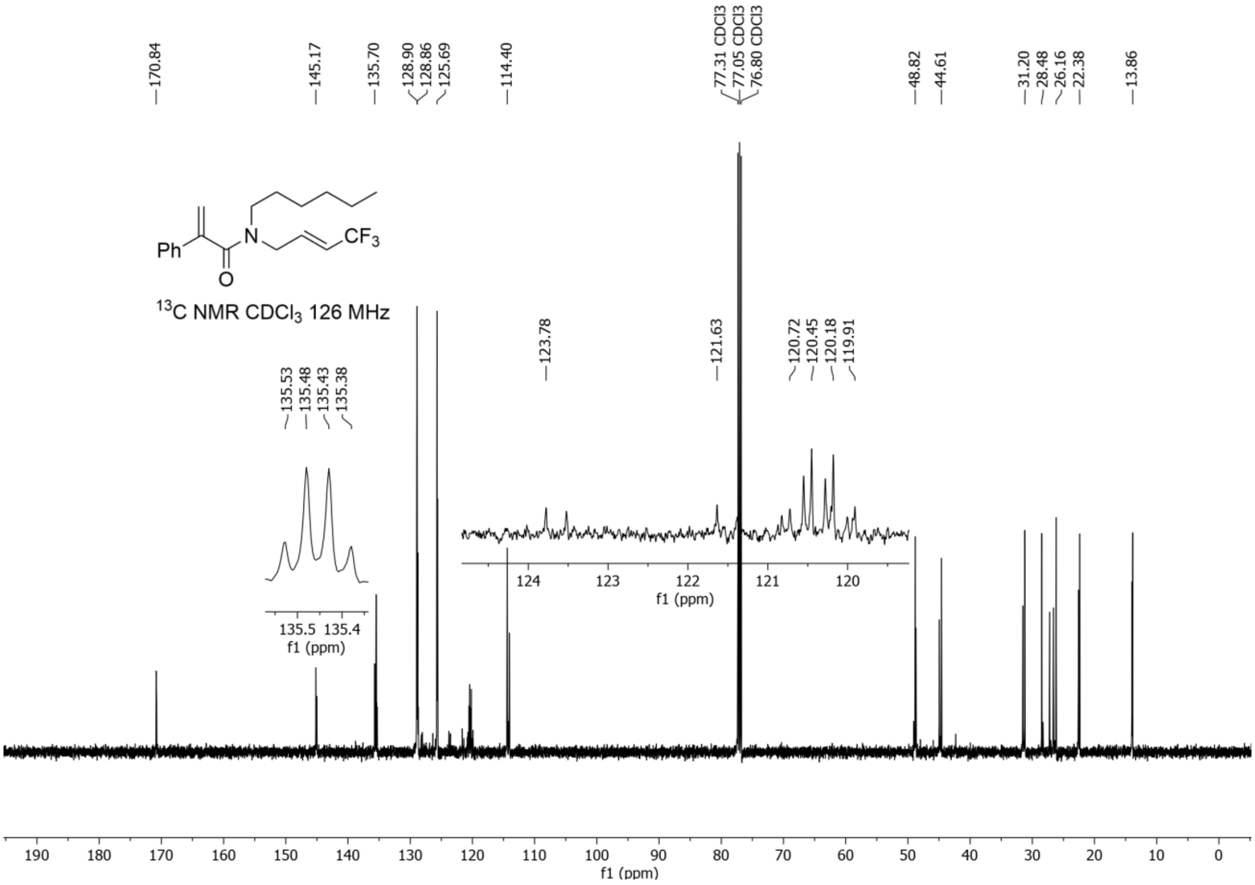

170.84
145.17
135.70
128.90
128.86
125.69
114.40
77.31 CDCl3
77.05 CDCl3
76.80 CDCl3
48.82
44.61
31.20
28.48
26.16
22.38
13.86
Ph
N
CF3
O
13C NMR CDCl3 126 MHz
135.53
135.48
135.43
135.38
123.78
121.63
120.72
120.45
120.18
119.91
f1 (ppm)

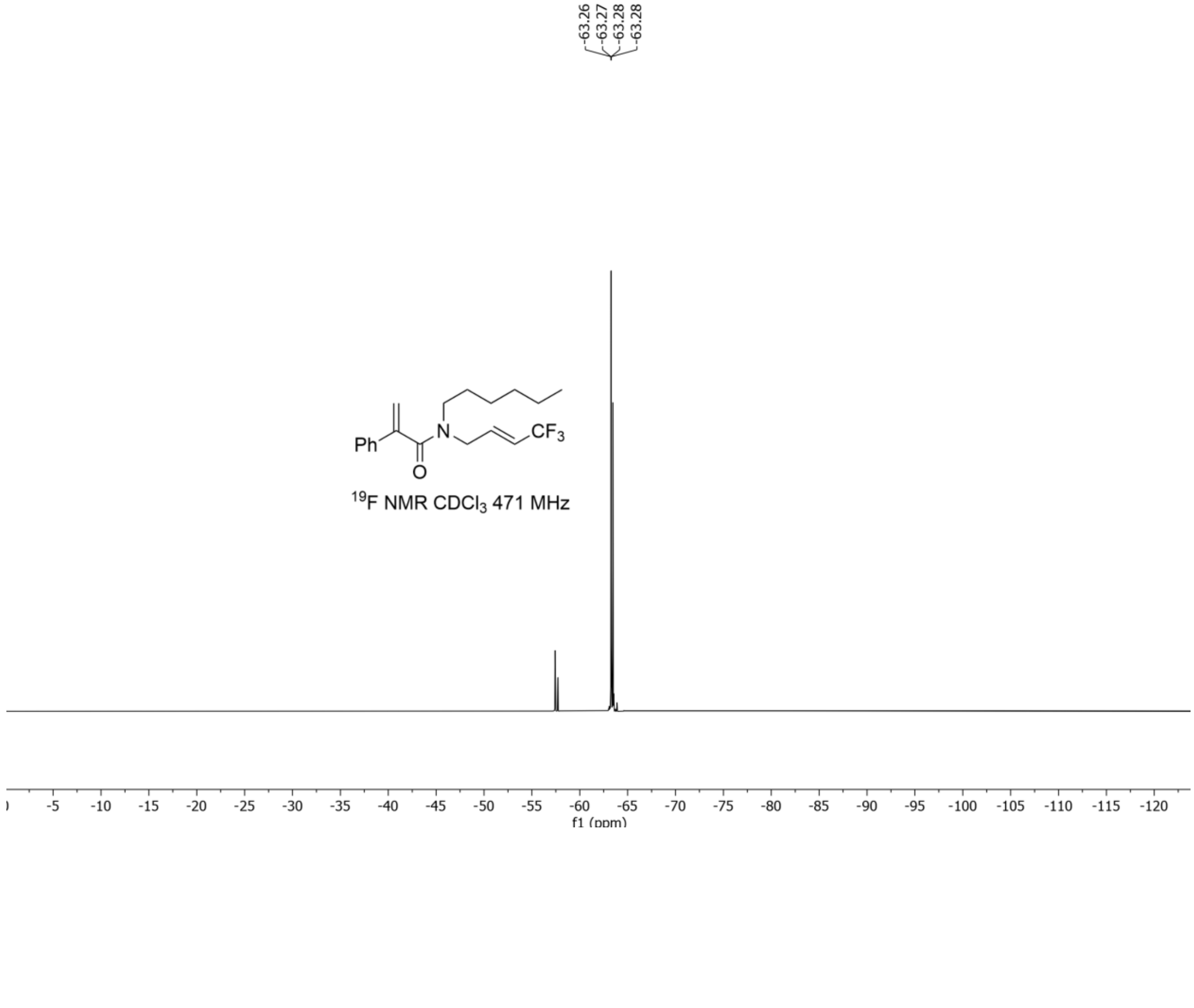

-63.26
-63.27
-63.28
-63.28
Ph
N
CF3
O
19F NMR CDCl3 471 MHz
f1 (ppm)

(*E*)-*N*-neopentyl-2-phenyl-*N*-(4,4,4-trifluorobut-2-en-1-yl)acrylamide (2y)

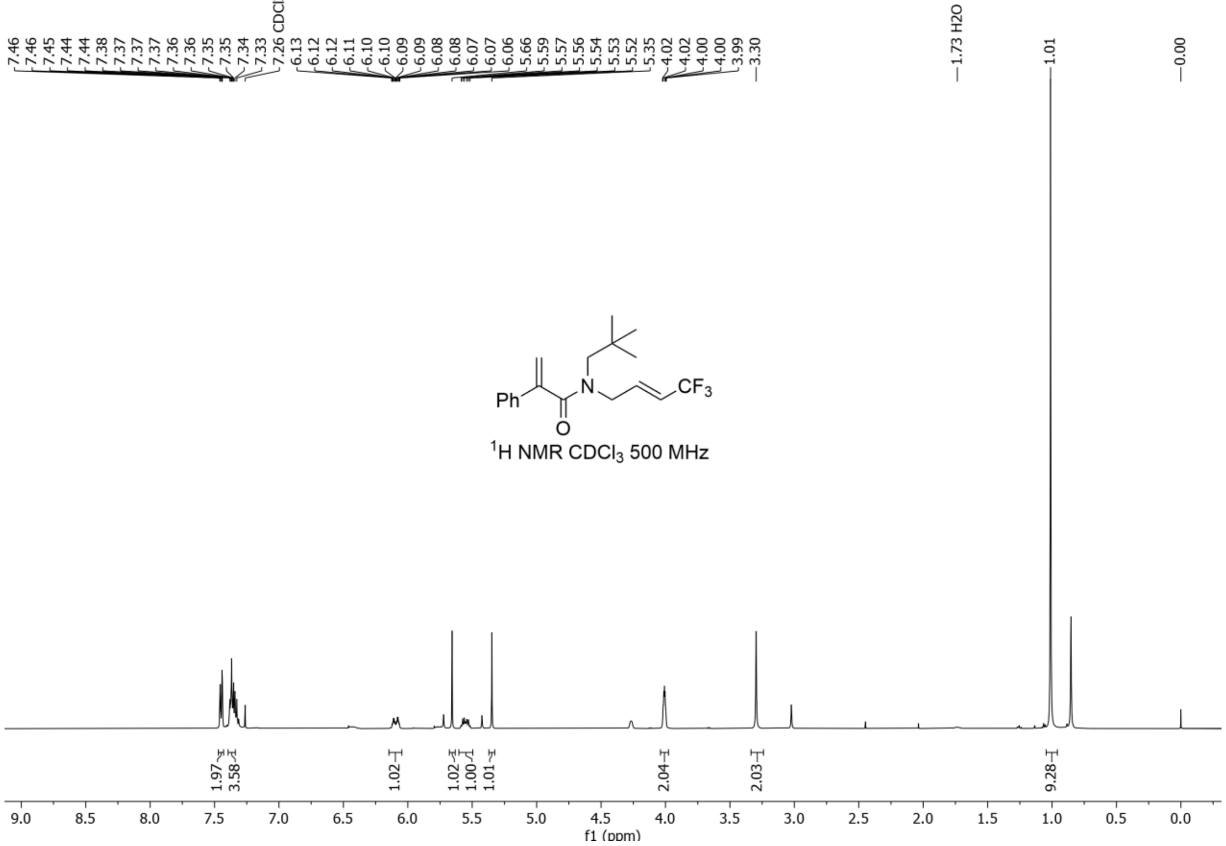

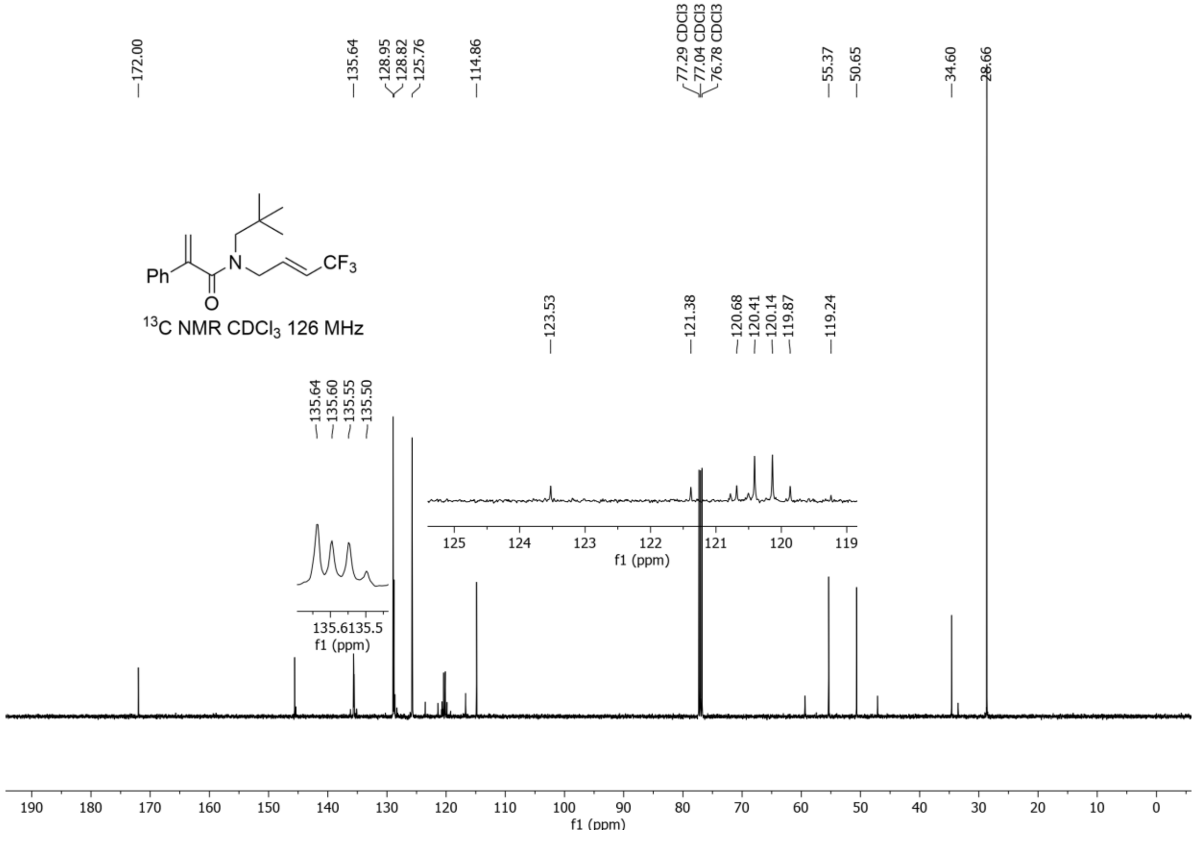

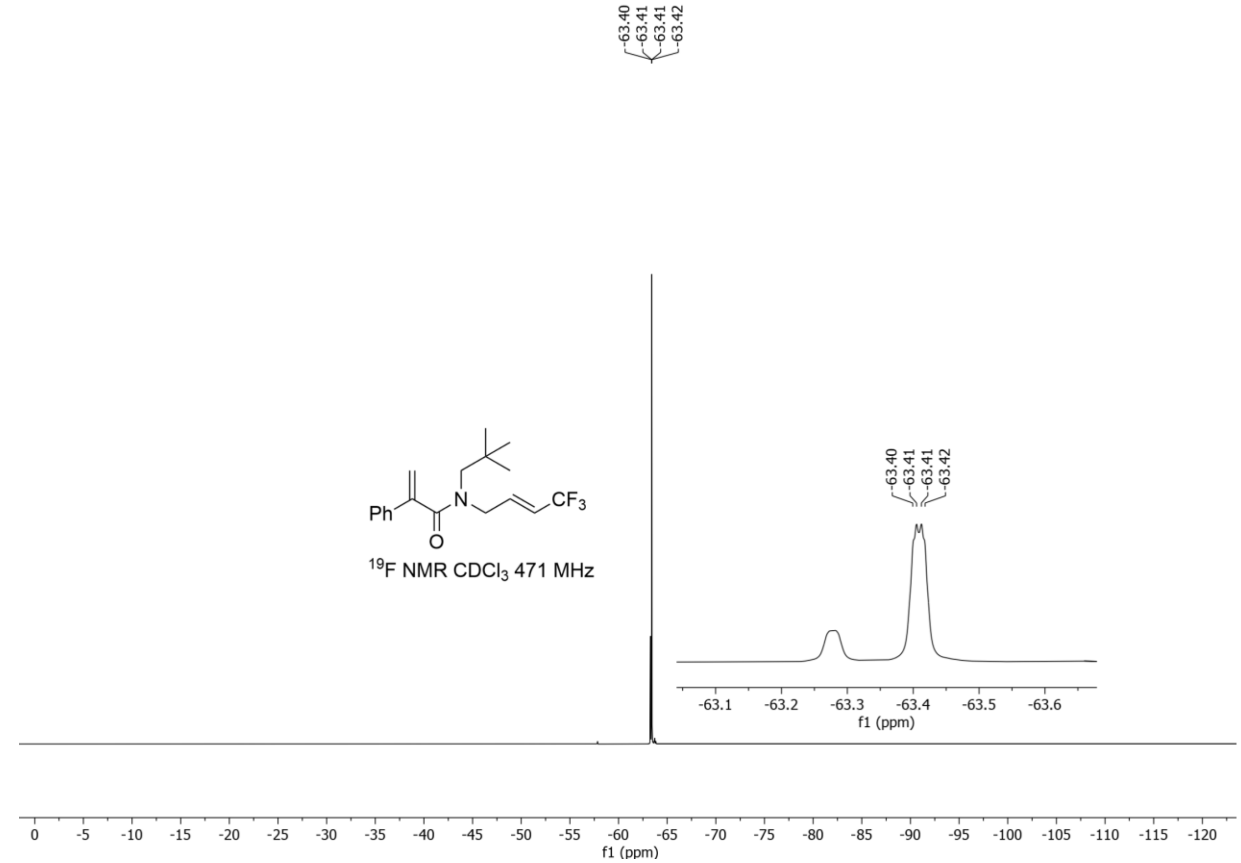

***N*-phenyl-*N*-((*E*)-4,4,4-trifluorobut-2-en-1-yl)cinnamamide (S1)**

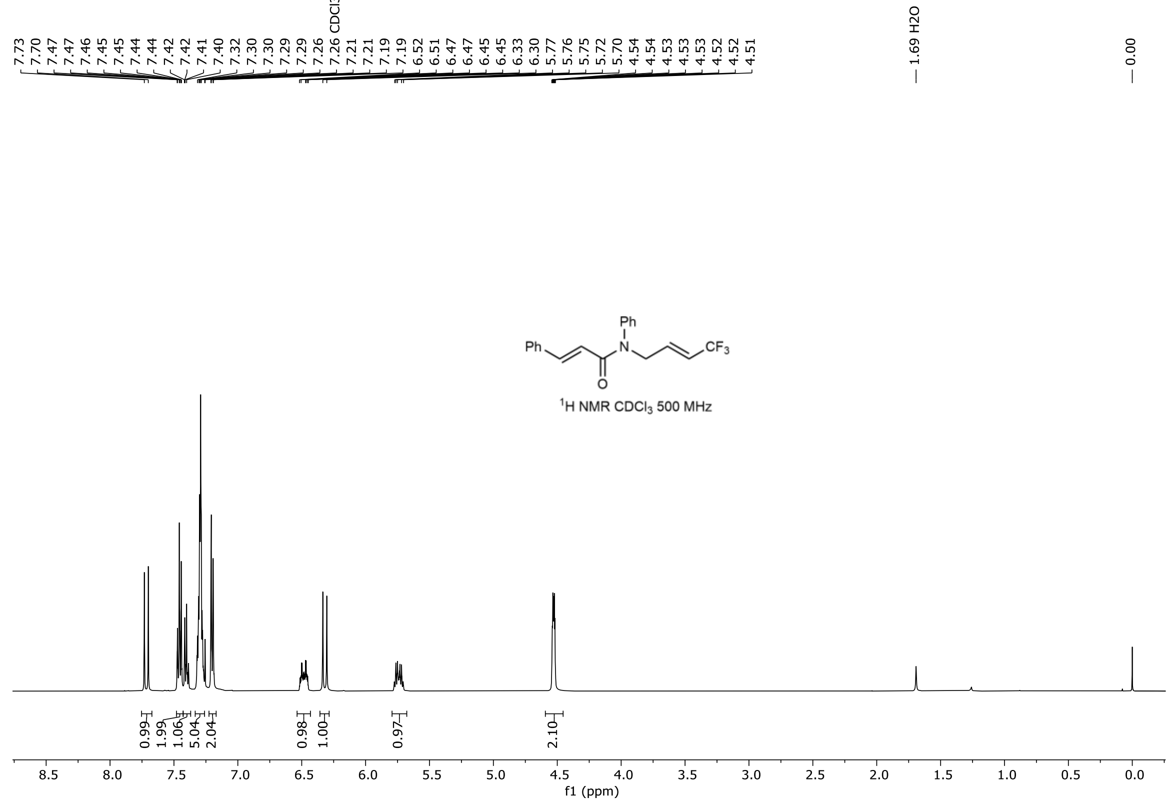

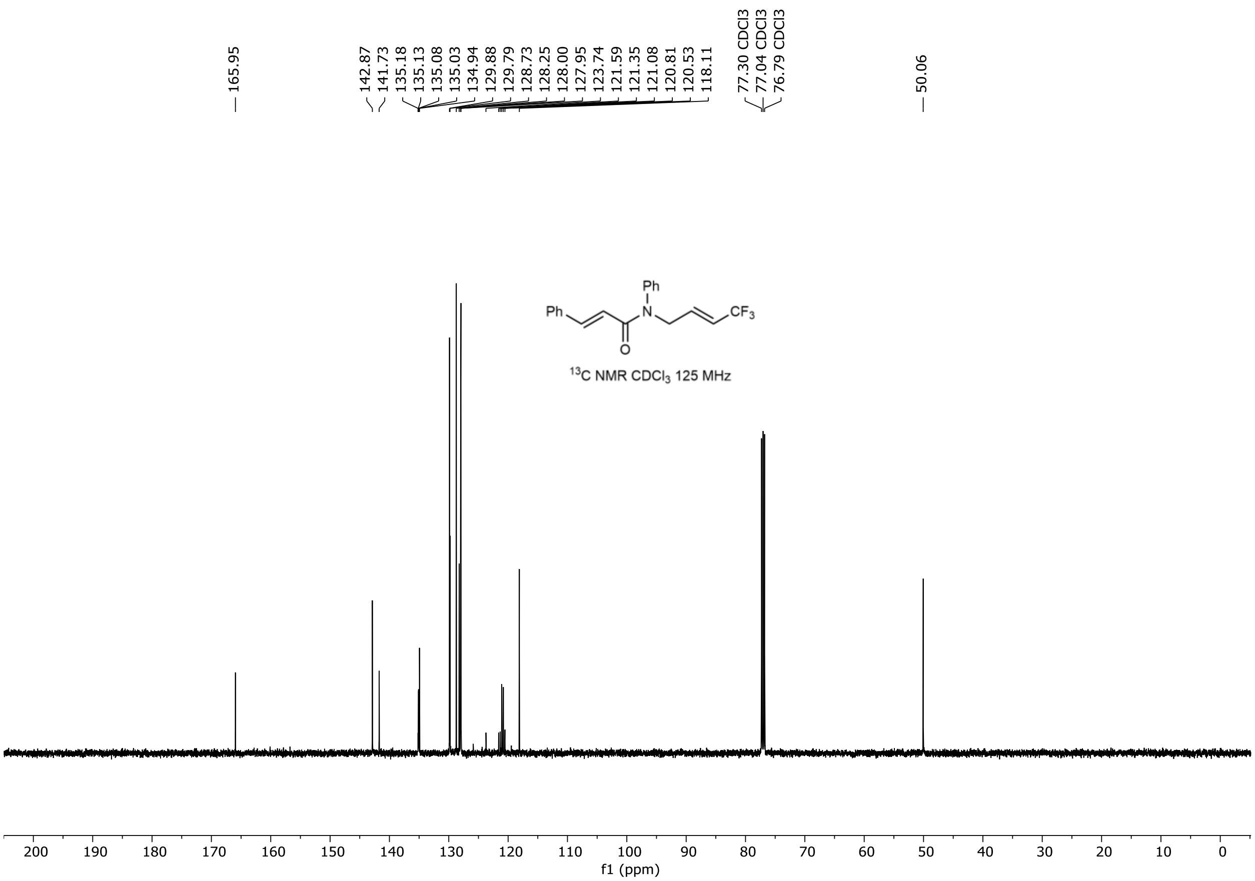

165.95
142.87
141.73
135.18
135.13
135.08
135.03
134.94
129.88
129.79
128.73
128.25
128.00
127.95
123.74
121.59
121.35
121.08
120.81
120.53
118.11
77.30 CDCl3
77.04 CDCl3
76.79 CDCl3
50.06
Ph
Ph
N
CF3
O
13C NMR CDCl3 125 MHz
200 190 180 170 160 150 140 130 120 110 100 90 80 70 60 50 40 30 20 10 0
f1 (ppm)

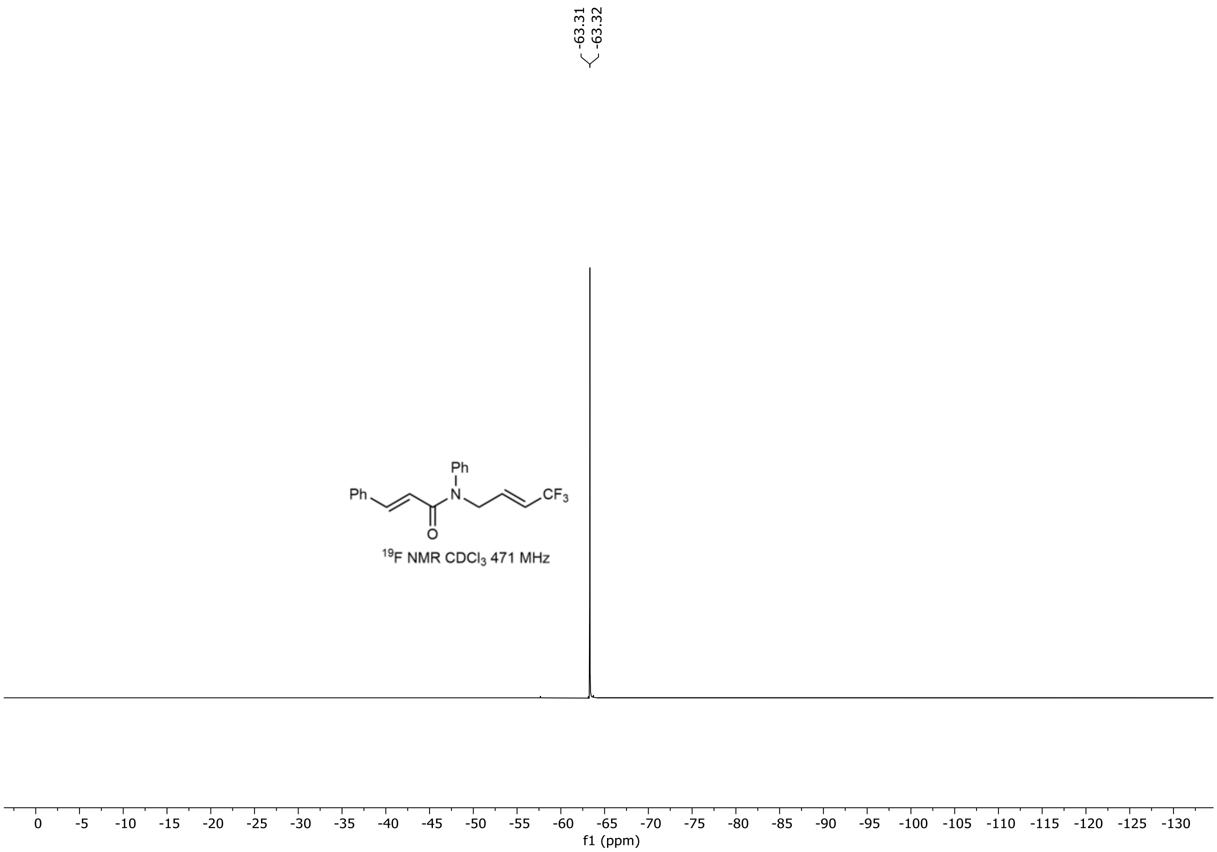

-63.31
-63.32
Ph
Ph
N
CF3
O
19F NMR CDCl3 471 MHz
0 -5 -10 -15 -20 -25 -30 -35 -40 -45 -50 -55 -60 -65 -70 -75 -80 -85 -90 -95 -100 -105 -110 -115 -120 -125 -130
f1 (ppm)

(*E*)-*N*,3-diphenyl-*N*-(4,4,4-trifluorobut-2-en-1-yl)but-3-enamide (S2)

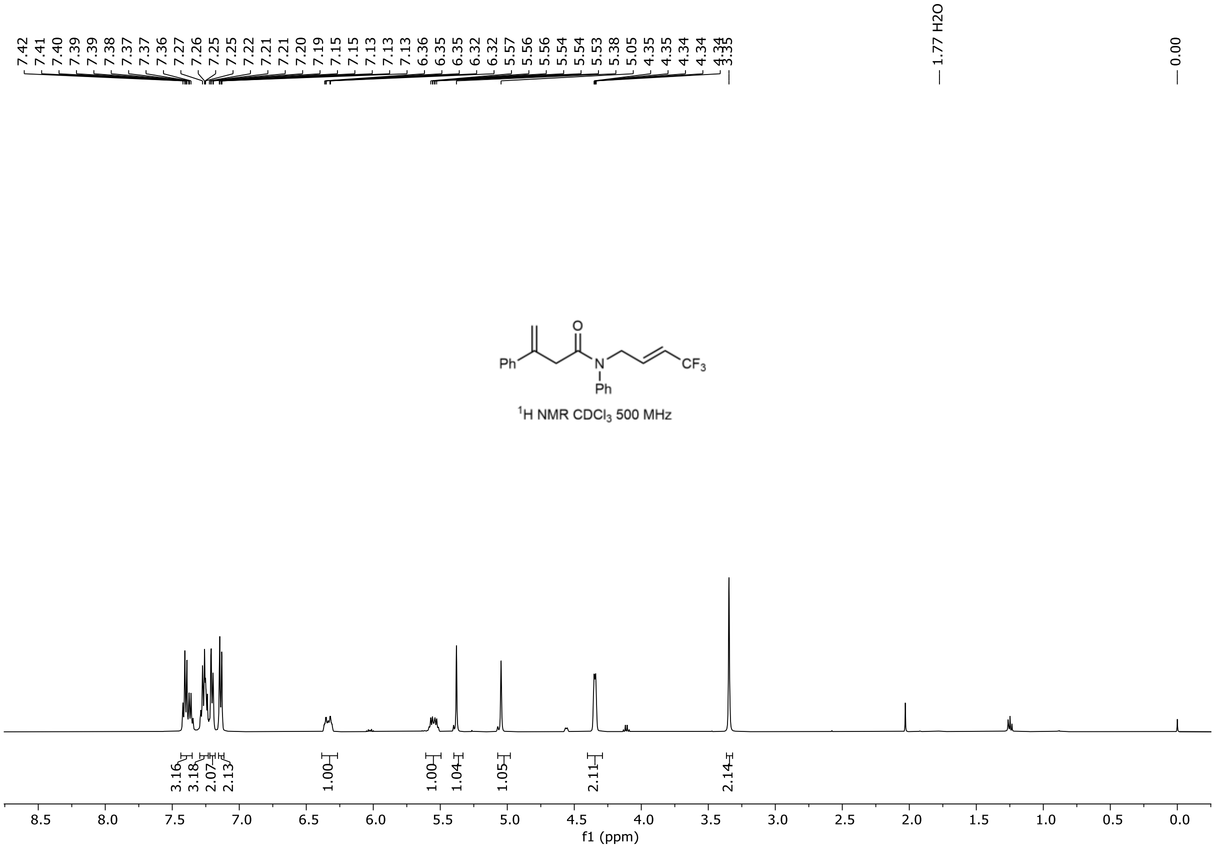

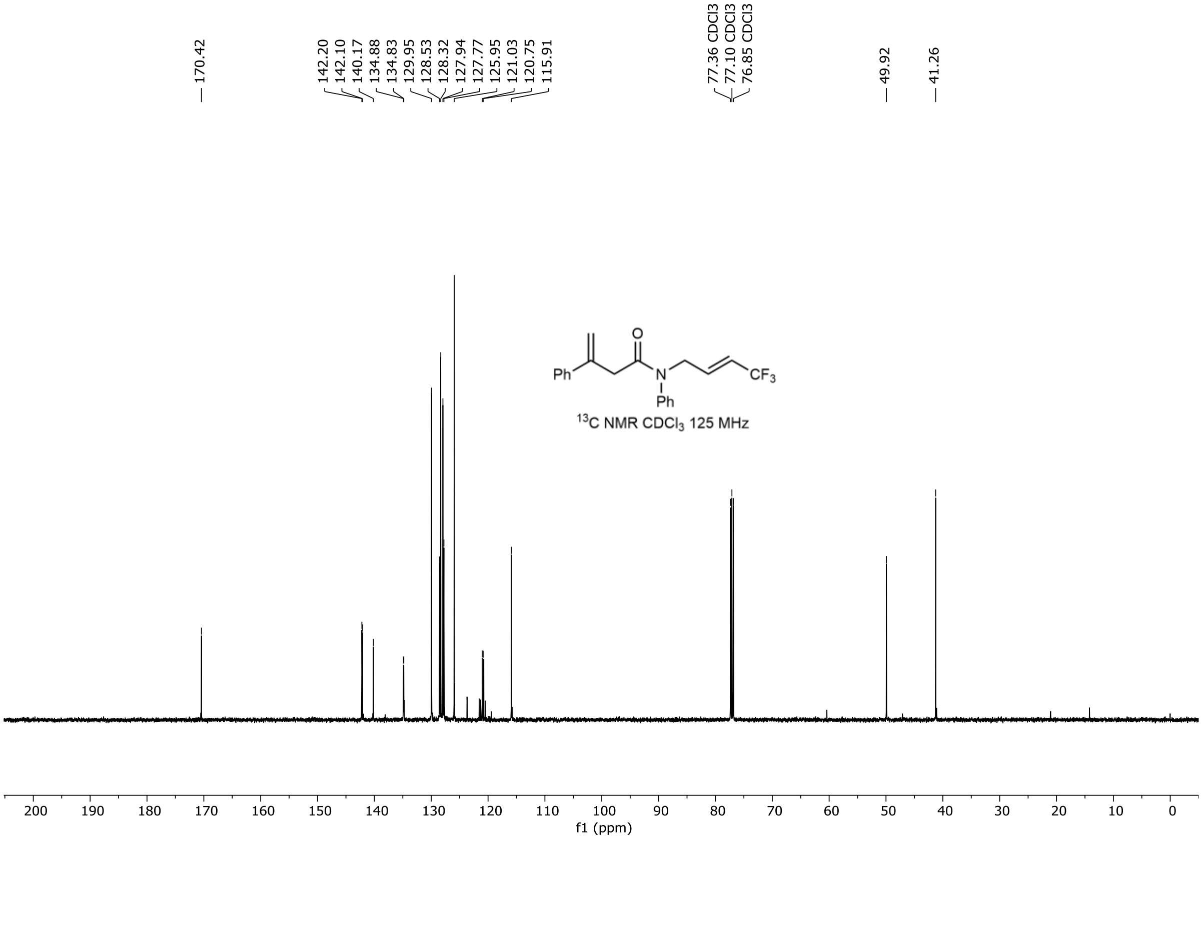

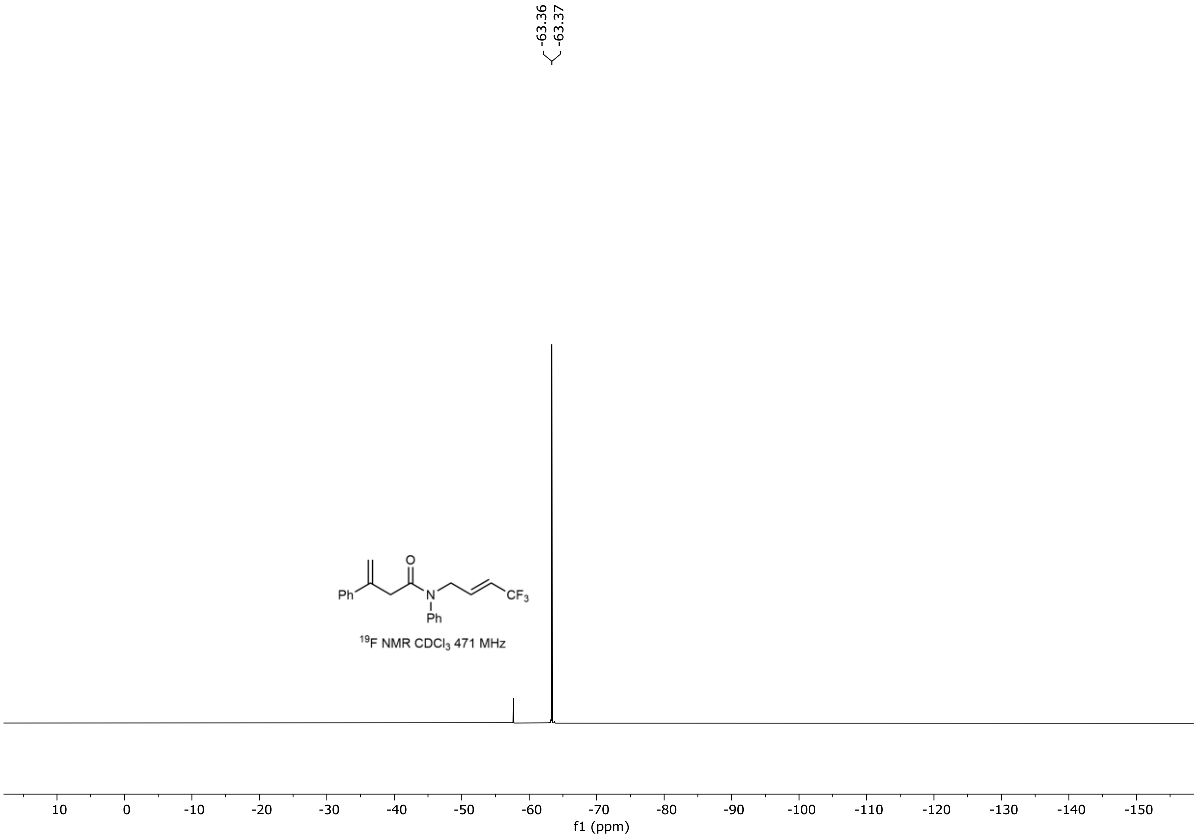

(*E*)-4-phenyl-*N*-tosyl-*N*-(4,4,4-trifluorobut-2-en-1-yl)pent-4-enamide (S3)

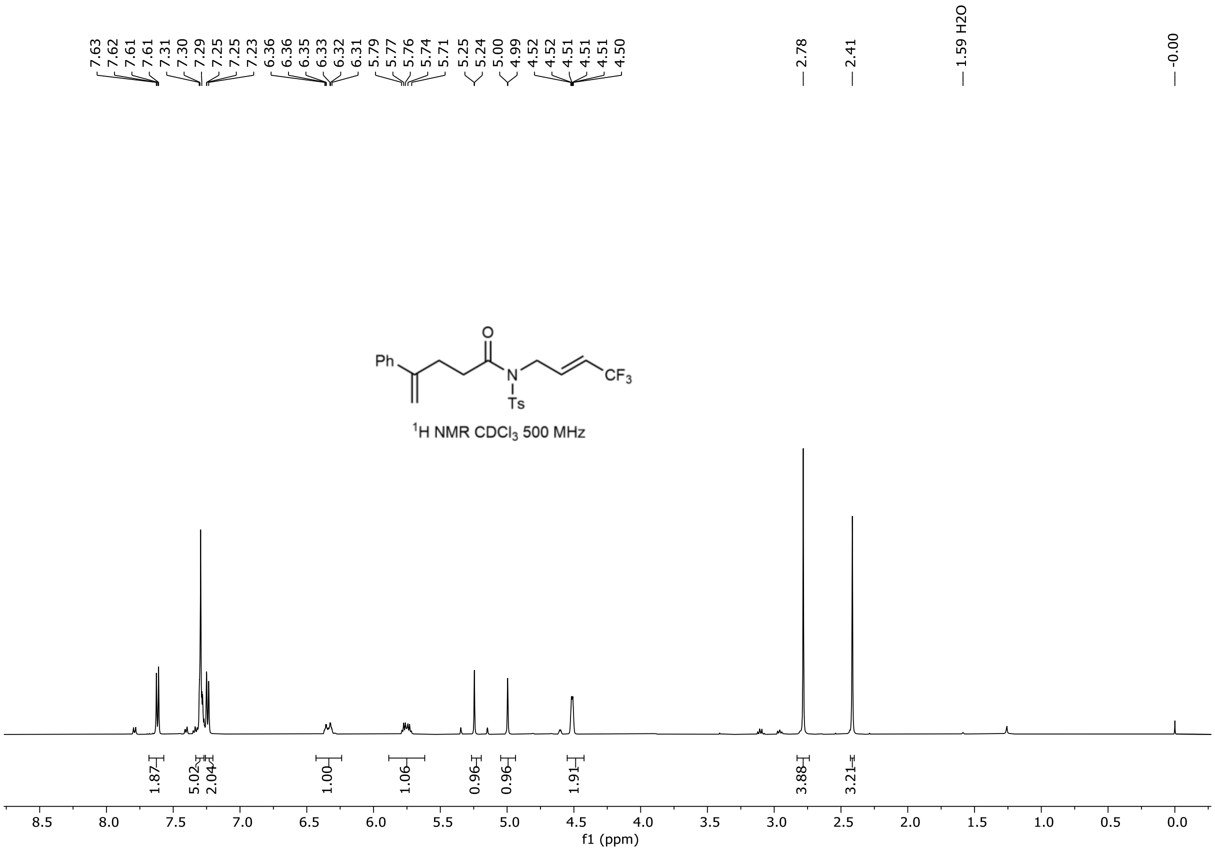

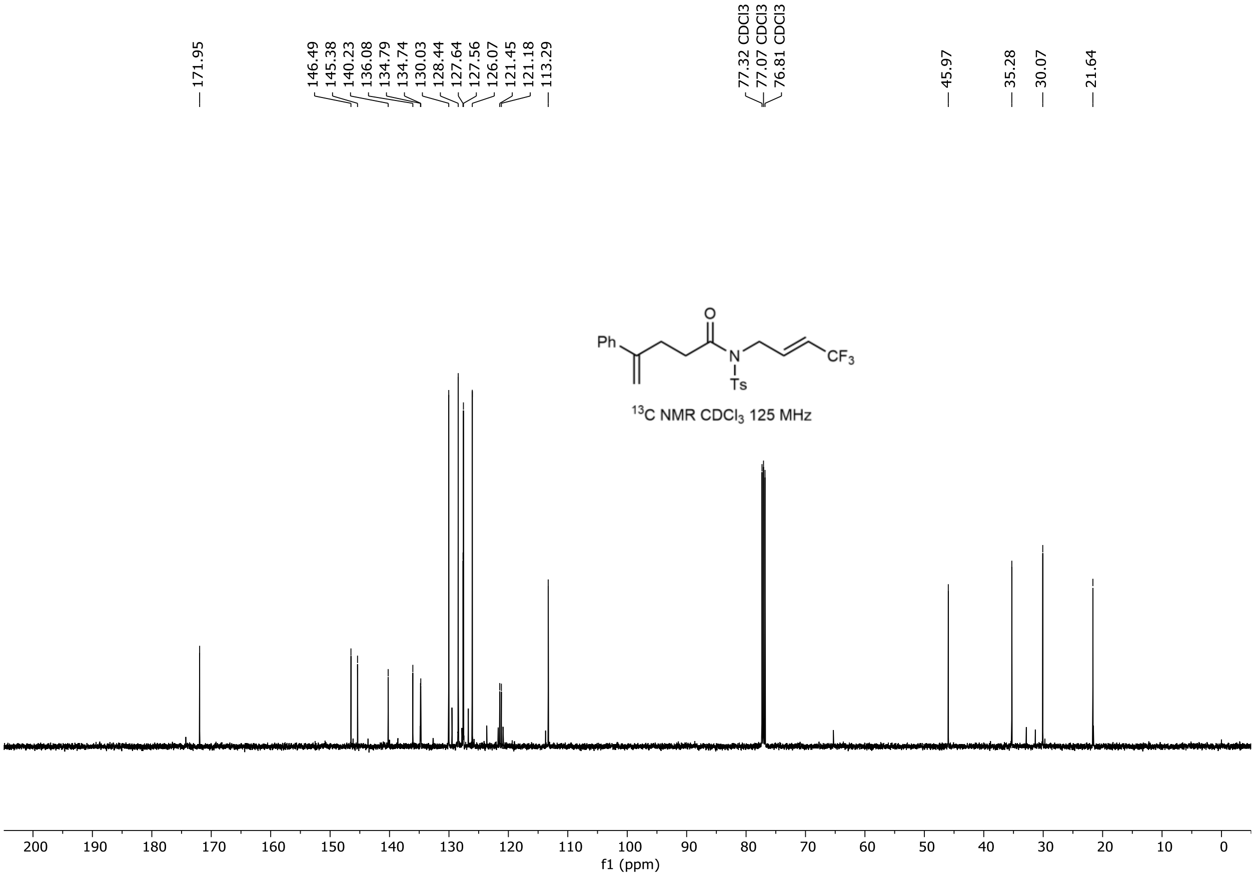
171.95
146.49
145.38
140.23
136.08
134.79
134.74
130.03
128.44
127.64
127.56
126.07
121.45
121.18
113.29
77.32 CDCl3
77.07 CDCl3
76.81 CDCl3
45.97
35.28
30.07
21.64
Ph
O
N
Ts
CF3
13C NMR CDCl3 125 MHz
200 190 180 170 160 150 140 130 120 110 100 90 80 70 60 50 40 30 20 10 0
f1 (ppm)

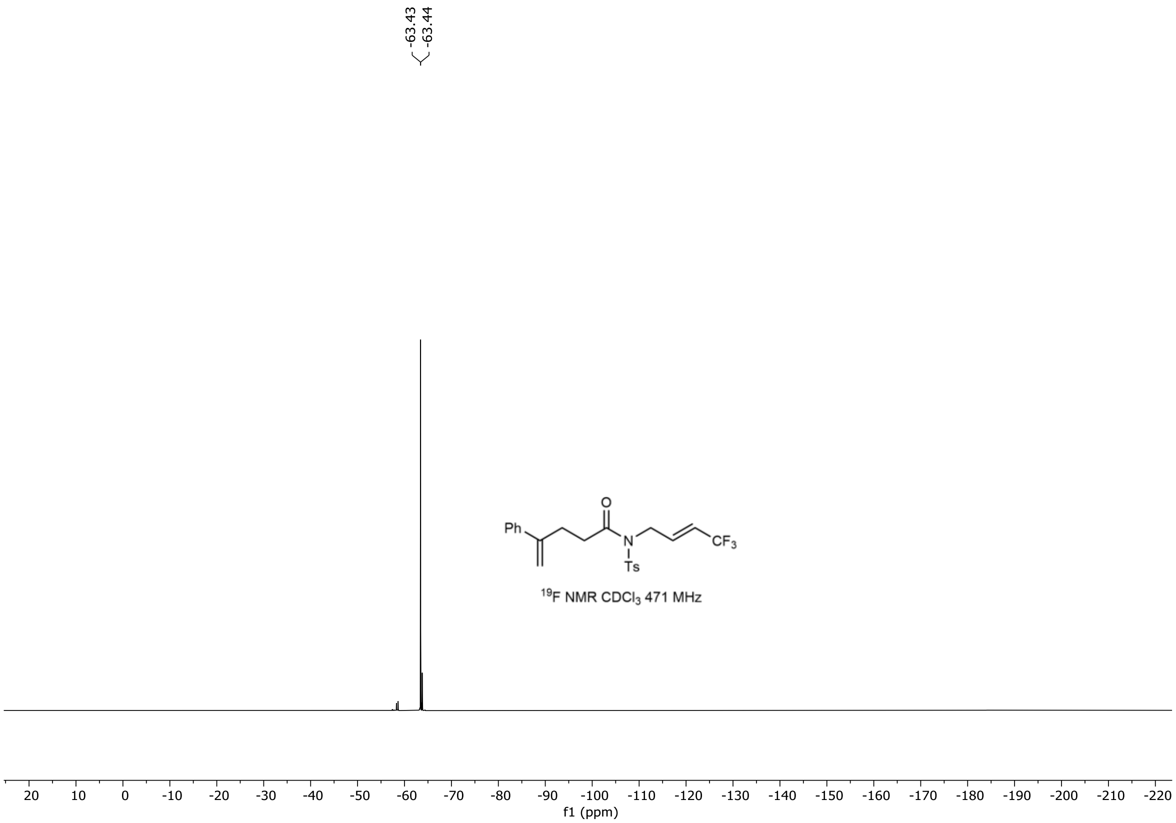
-63.43
-63.44
Ph
O
N
Ts
CF3
19F NMR CDCl3 471 MHz
20 10 0 -10 -20 -30 -40 -50 -60 -70 -80 -90 -100 -110 -120 -130 -140 -150 -160 -170 -180 -190 -200 -210 -220
f1 (ppm)

***cis*-4-(2,2-difluorovinyl)-3-methyl-1,3-diphenylpyrrolidin-2-one (*cis*-3a)**

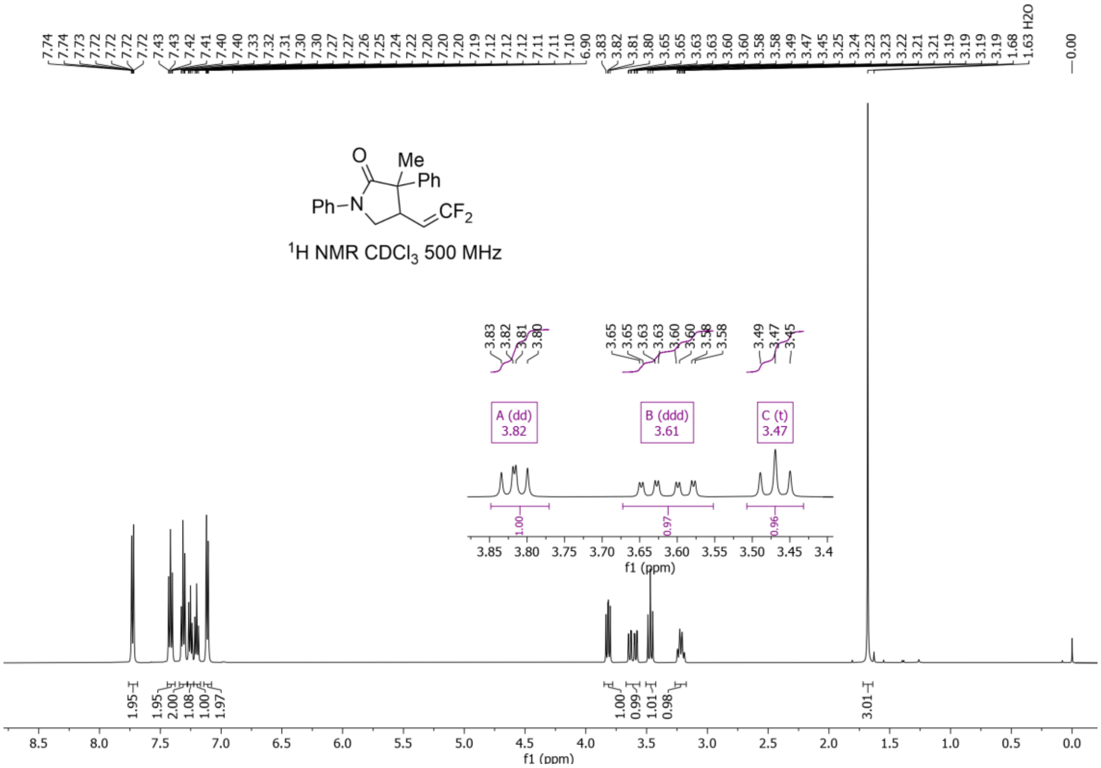

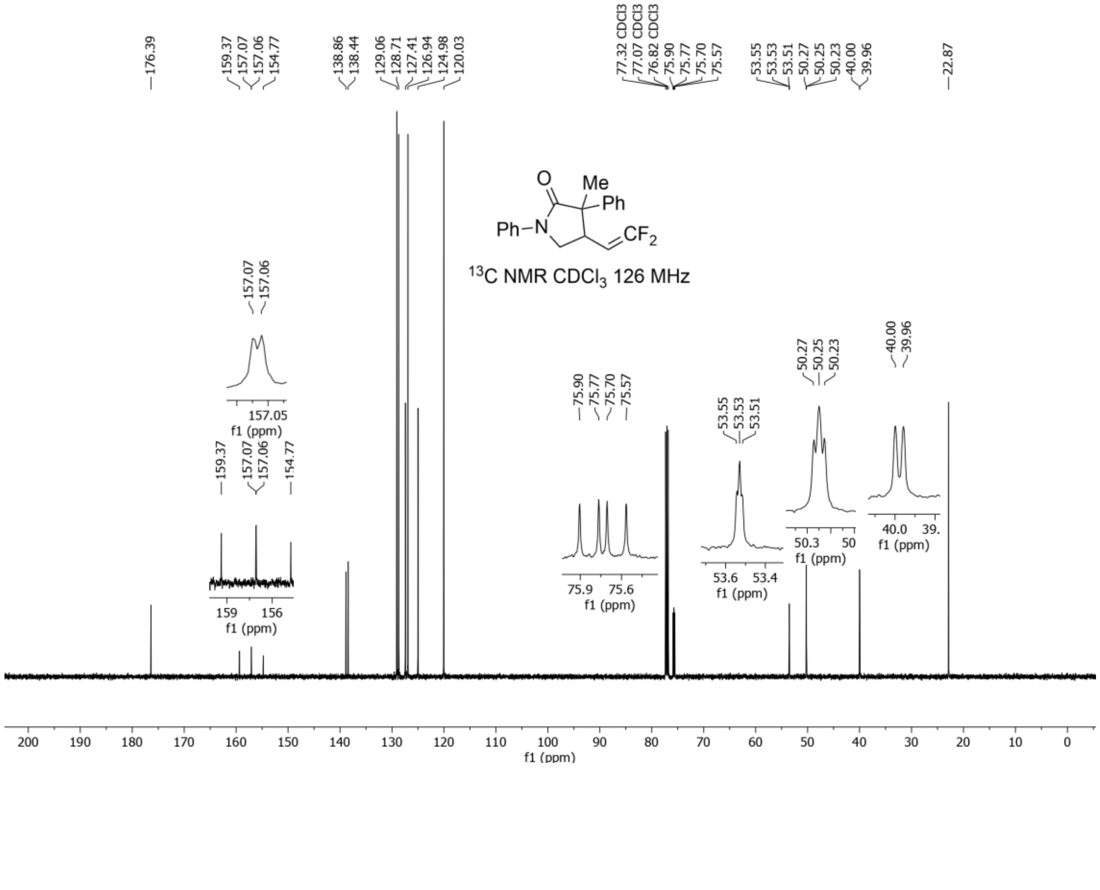

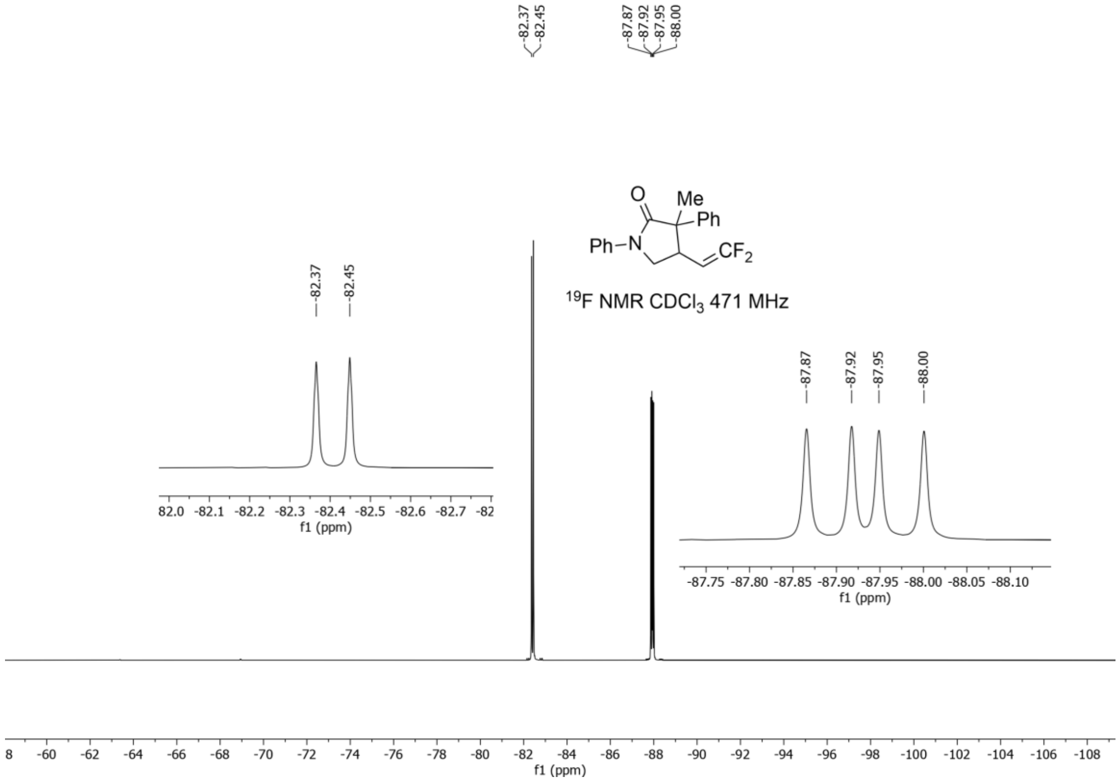

cis-4-(2,2-difluorovinyl)-3-(4-methoxyphenyl)-3-methyl-1-phenylpyrrolidin-2-one (_cis_-3b)

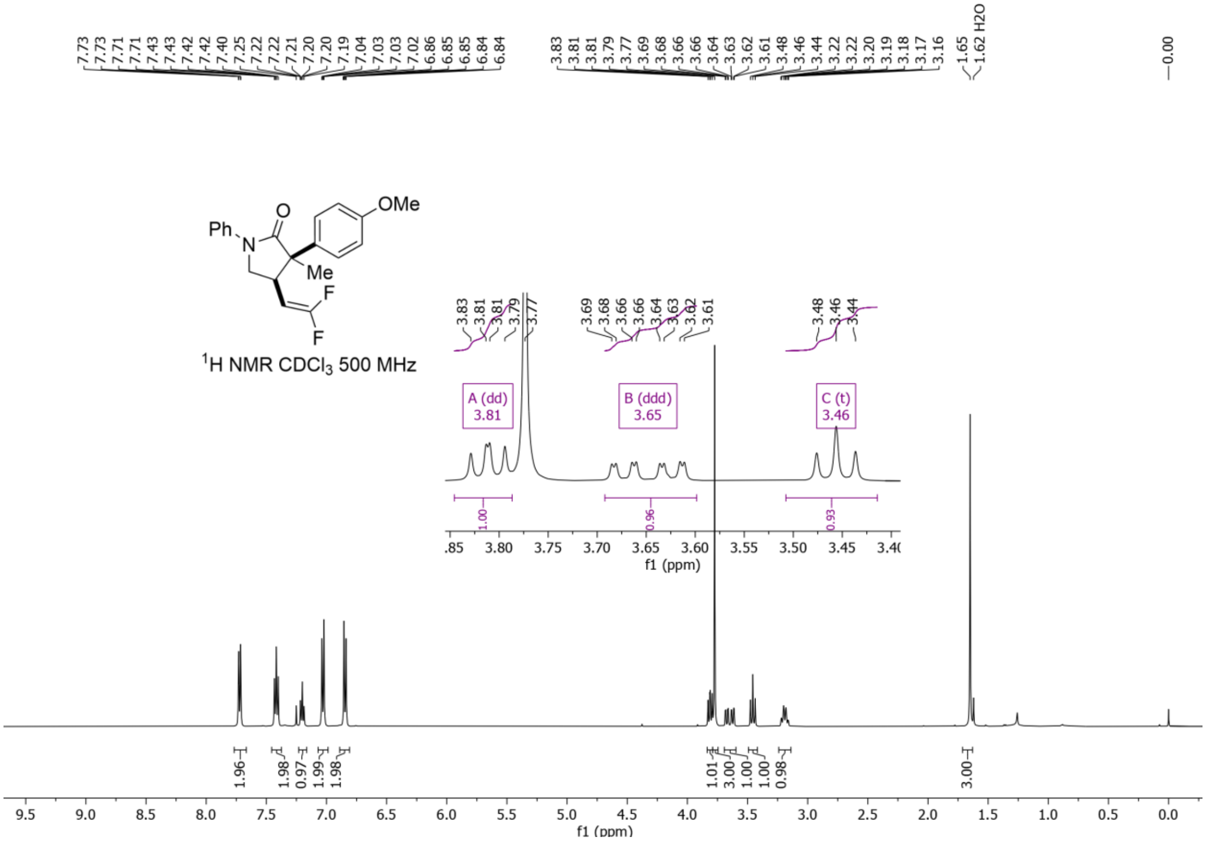

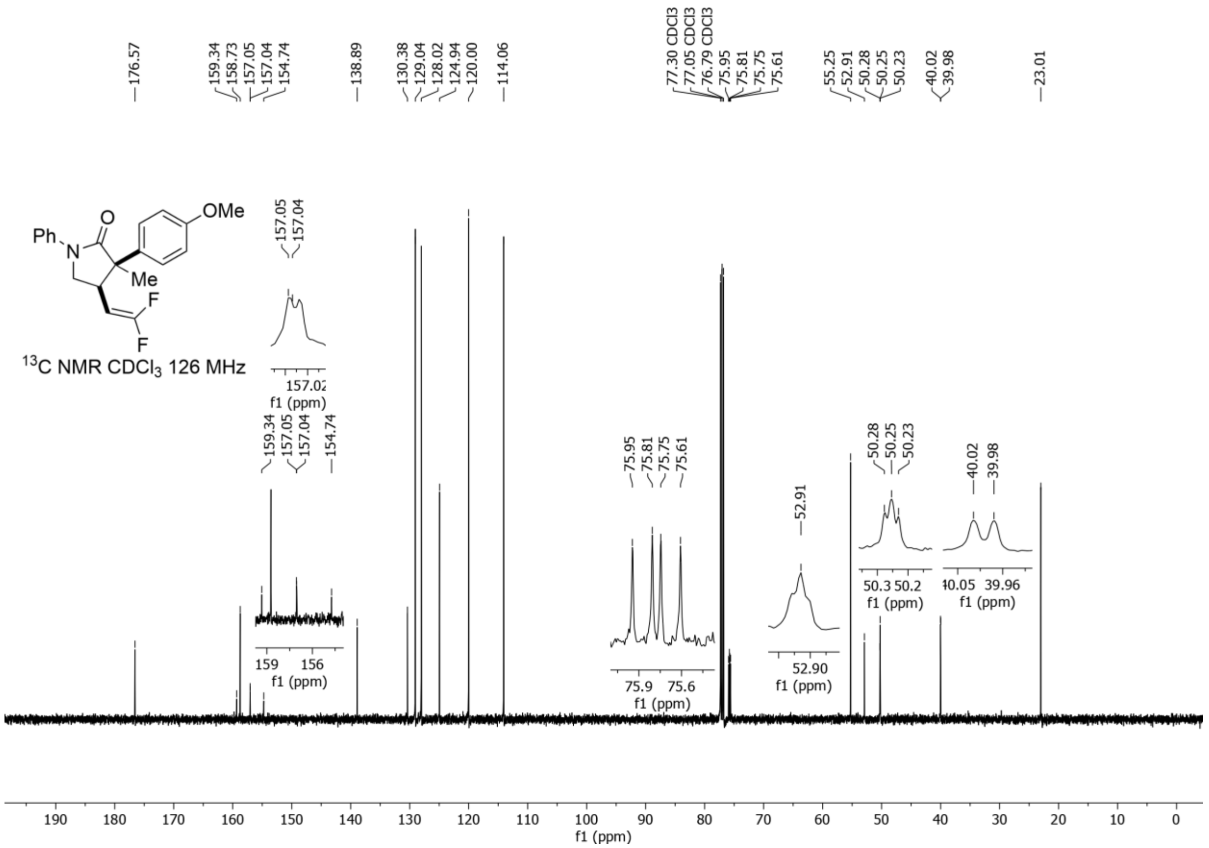

176.57
159.34
158.73
157.05
157.04
154.74
138.89
130.38
129.04
128.02
124.94
120.00
114.06
77.30 CDCl3
77.05 CDCl3
76.79 CDCl3
75.95
75.81
75.75
75.61
55.25
52.91
50.28
50.25
50.23
40.02
39.98
23.01
Ph
N
O
OMe
Me
F
F
^{13}C NMR CDCl$_3$ 126 MHz
f1 (ppm)

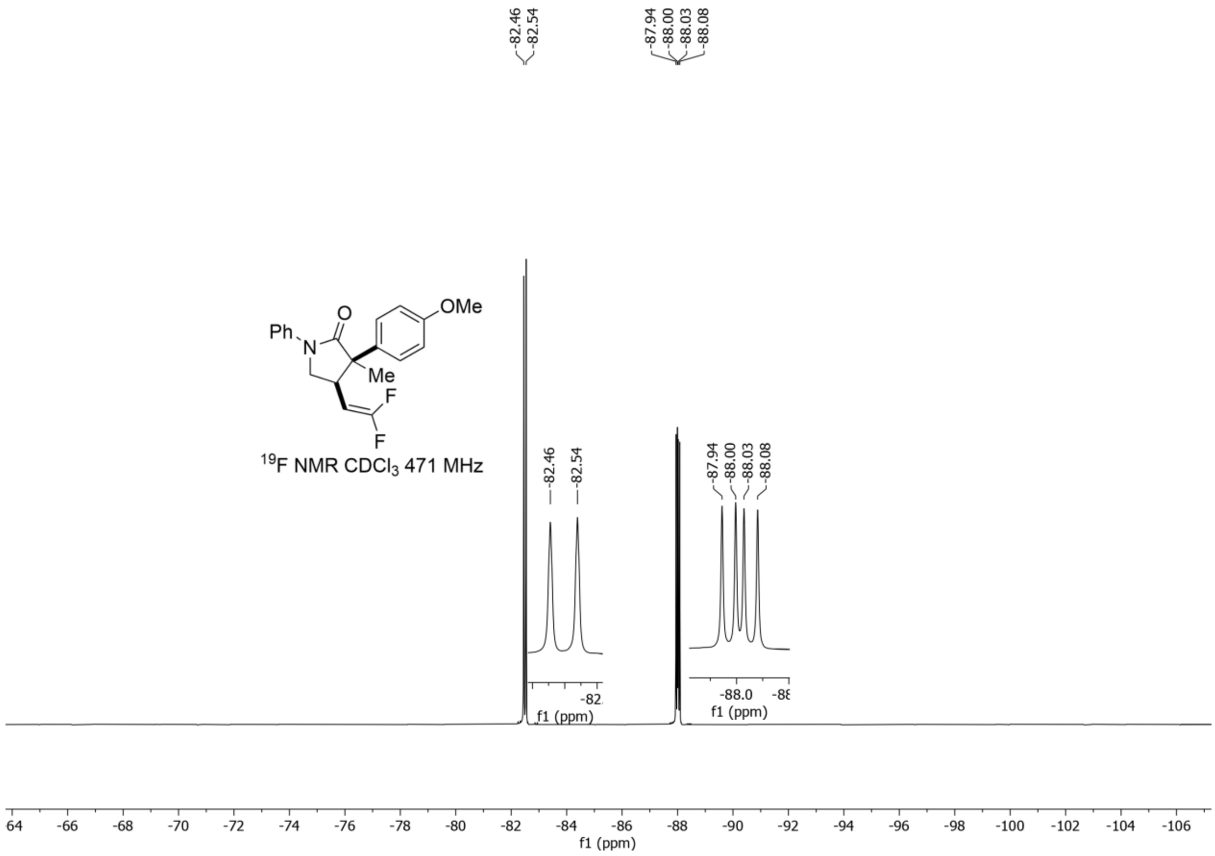

-82.46
-82.54
-87.94
-88.00
-88.03
-88.08
Ph
N
O
OMe
Me
F
F
^{19}F NMR CDCl$_3$ 471 MHz
f1 (ppm)

cis-4-(2,2-difluorovinyl)-3-methyl-1-phenyl-3-(p-tolyl)pyrrolidin-2-one (cis-3c)

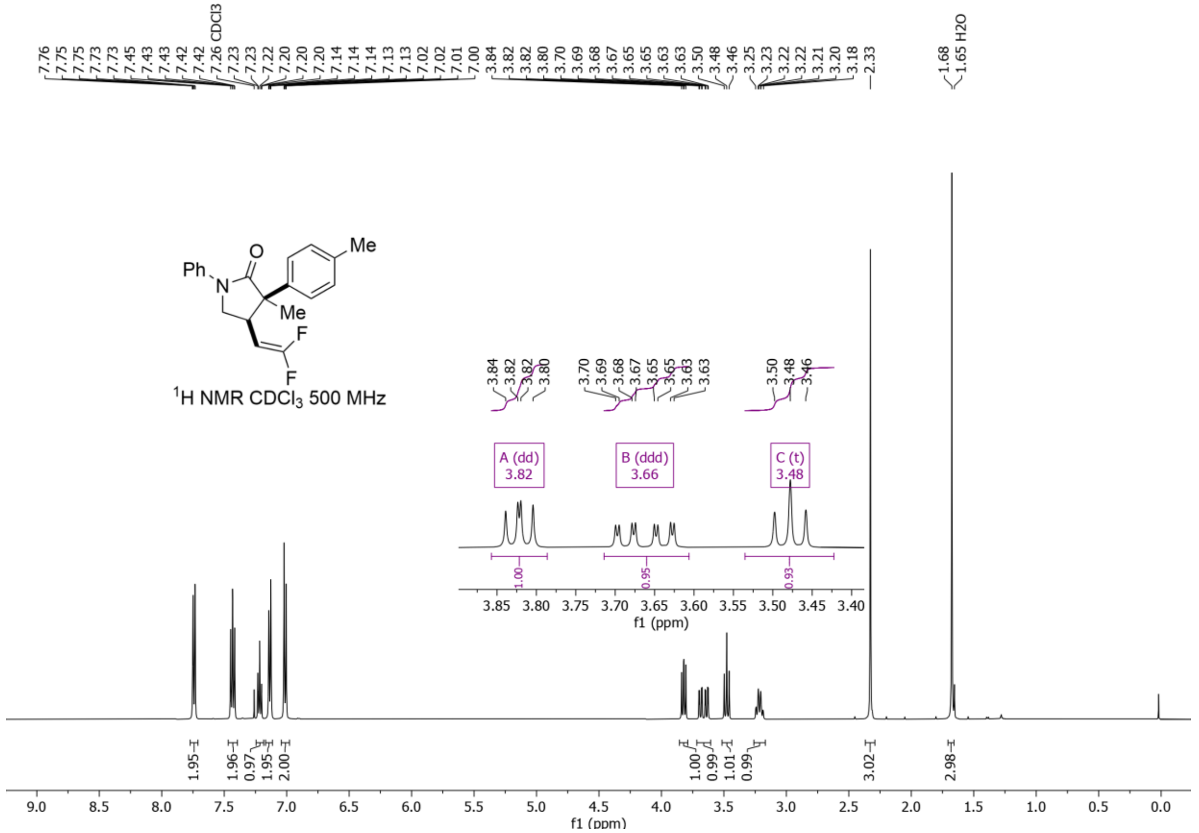

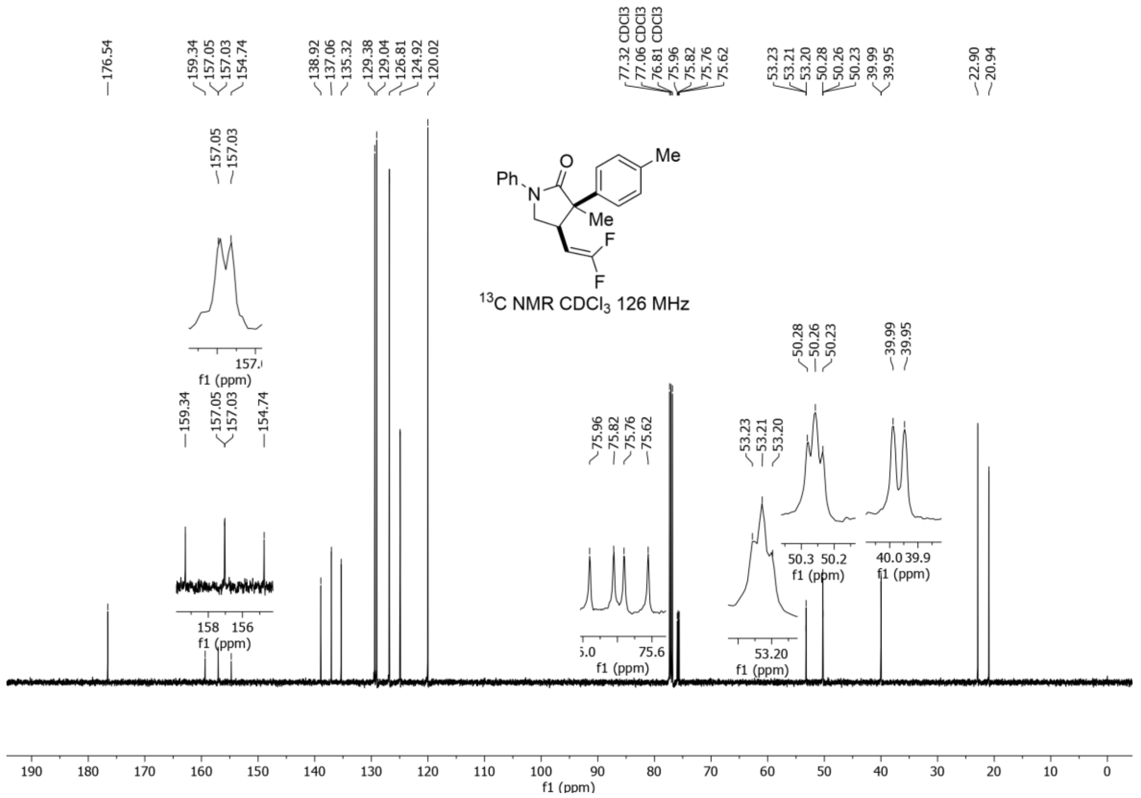

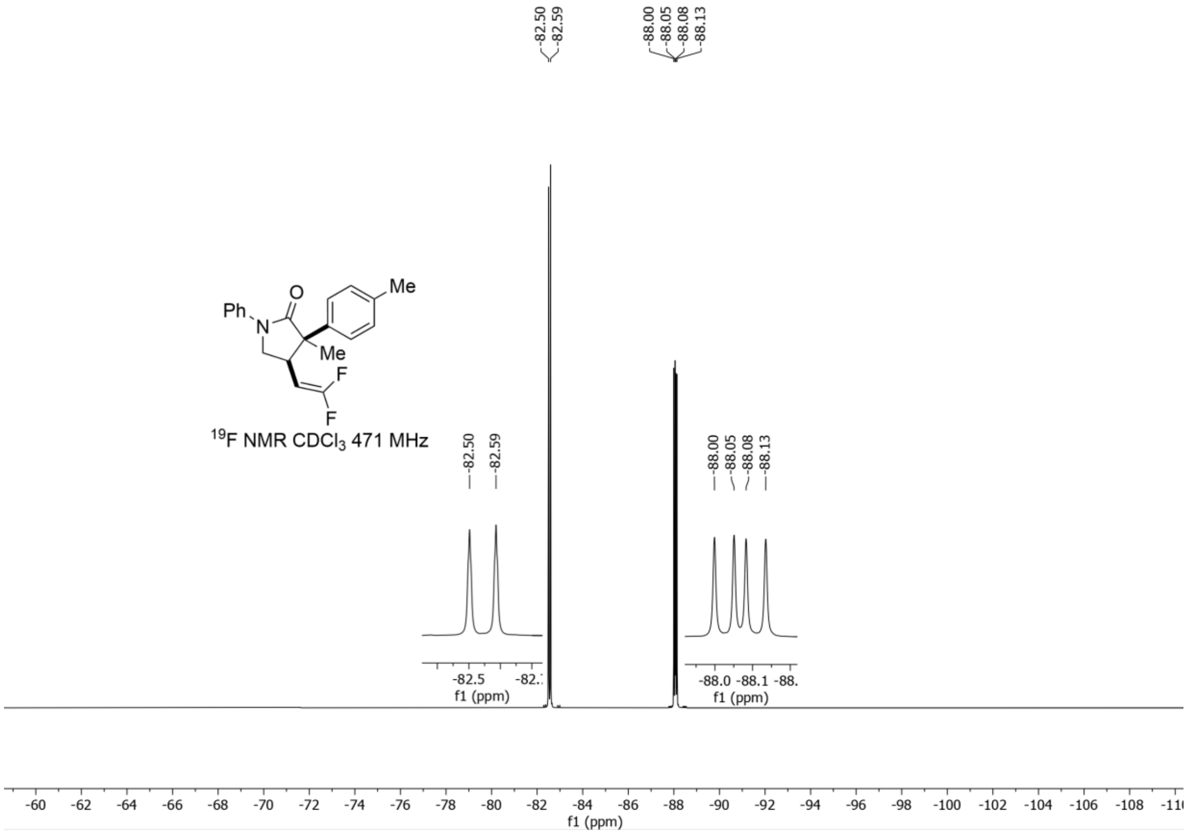

cis-3-(4-(tert-butyl)phenyl)-4-(2,2-difluorovinyl)-3-methyl-1-phenylpyrrolidin-2-one (cis-3d)

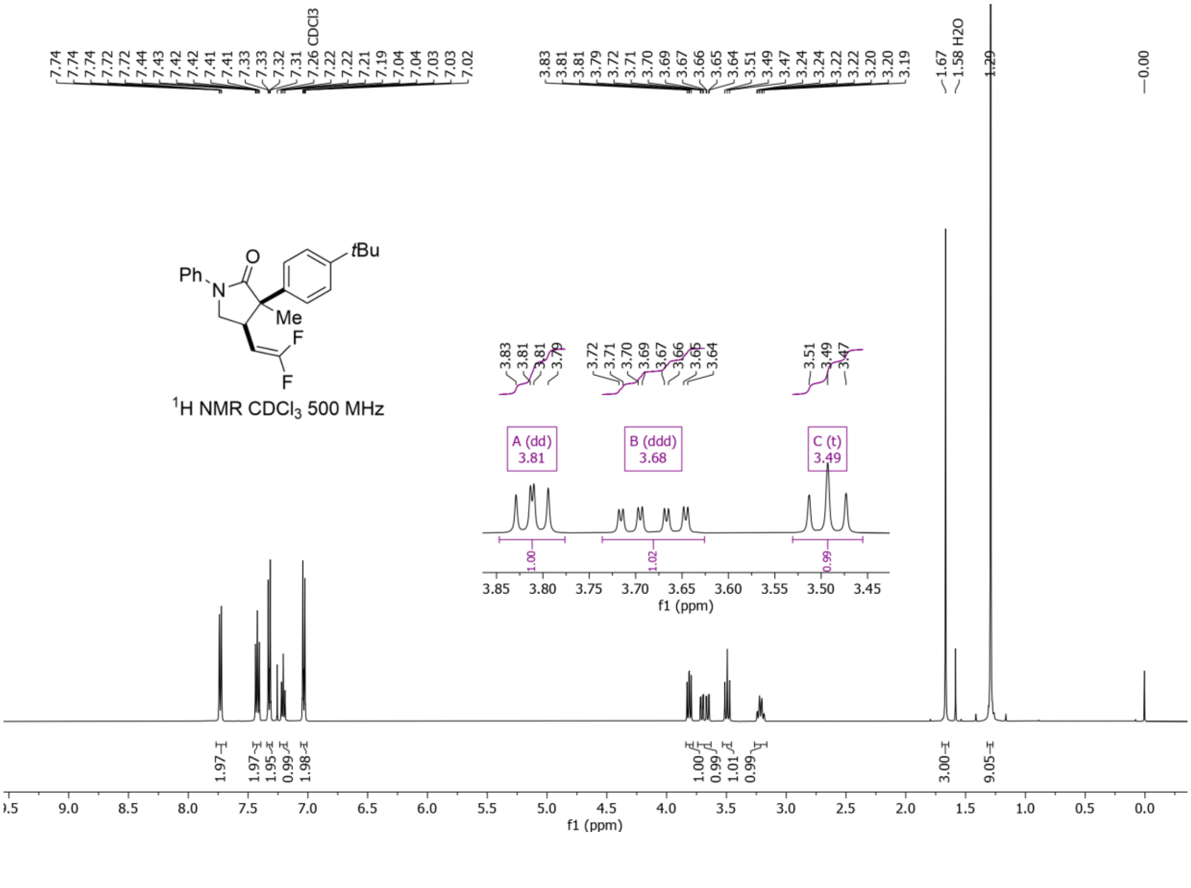

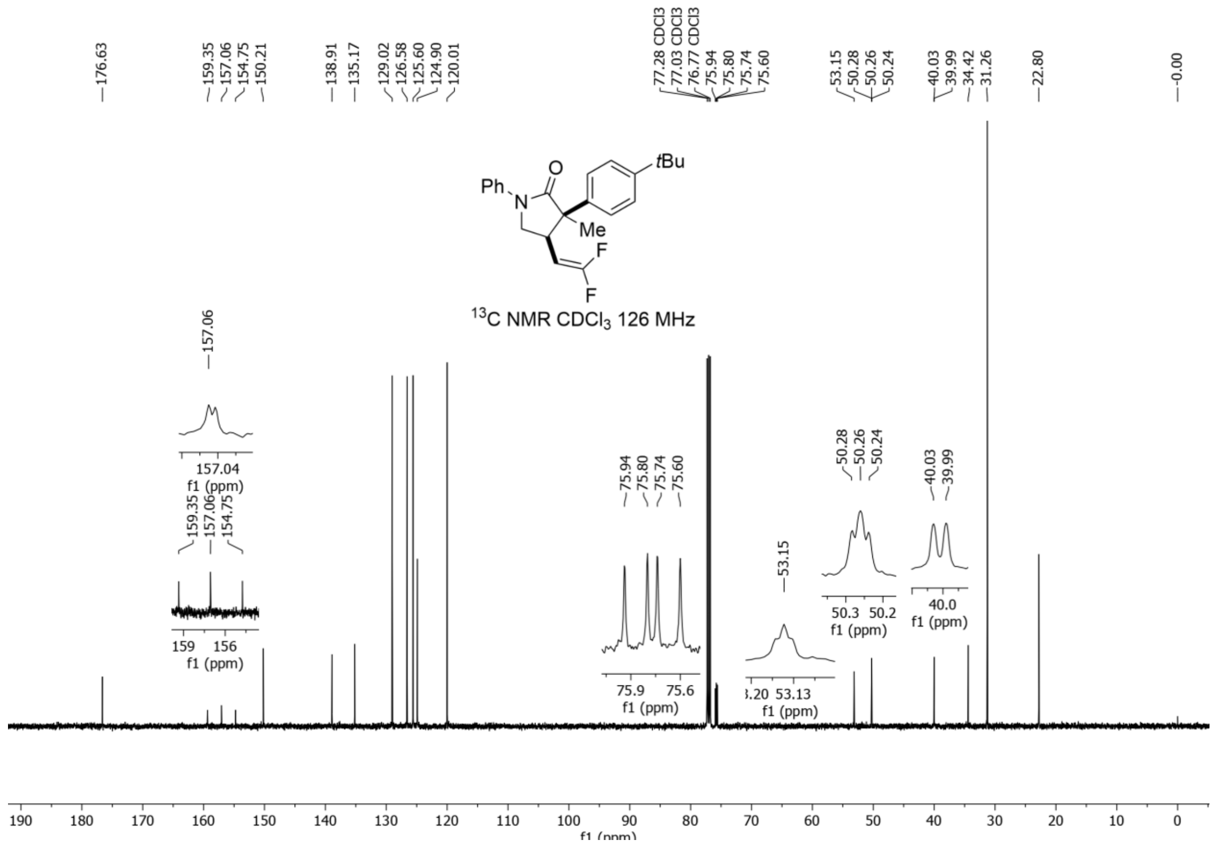

13C NMR CDCl3 126 MHz
Ph
N
O
tBu
Me
F
F
f1 (ppm)

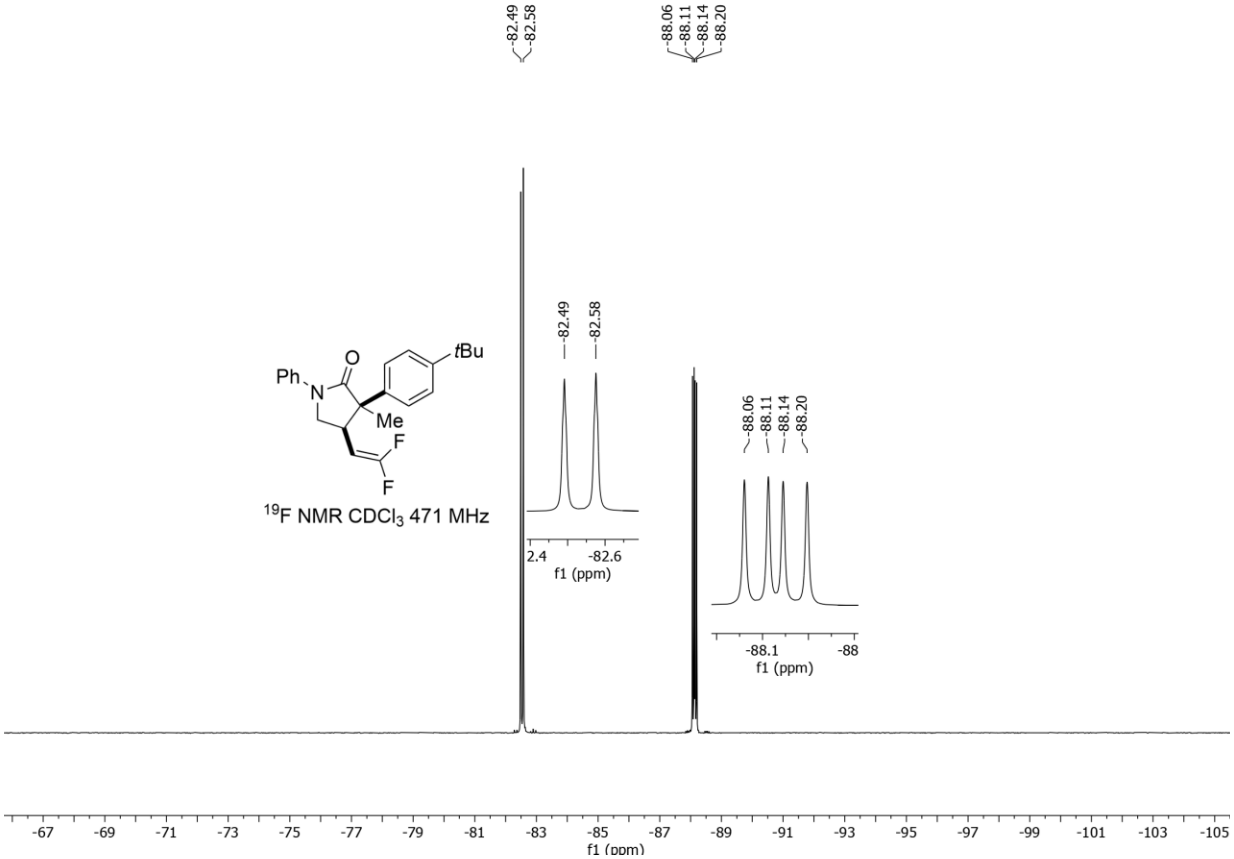

19F NMR CDCl3 471 MHz
Ph
N
O
tBu
Me
F
F
f1 (ppm)

cis-4-(2,2-difluorovinyl)-3-(4-fluorophenyl)-3-methyl-1-phenylpyrrolidin-2-one (_cis_-3e)

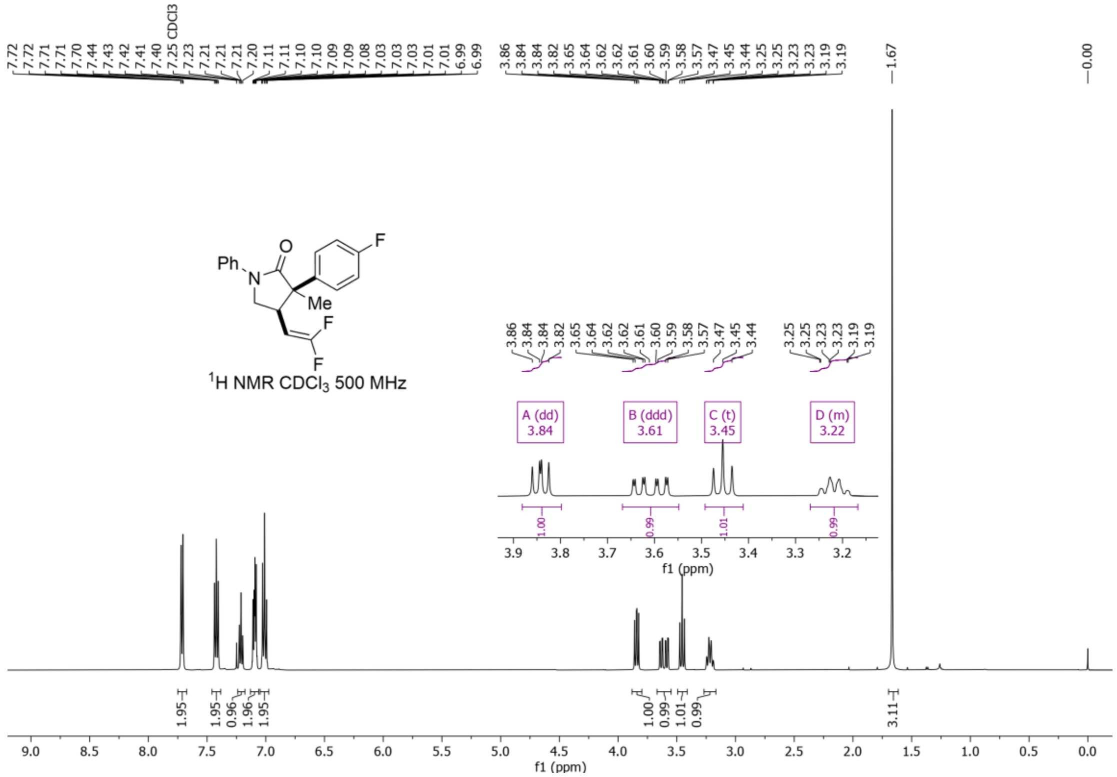

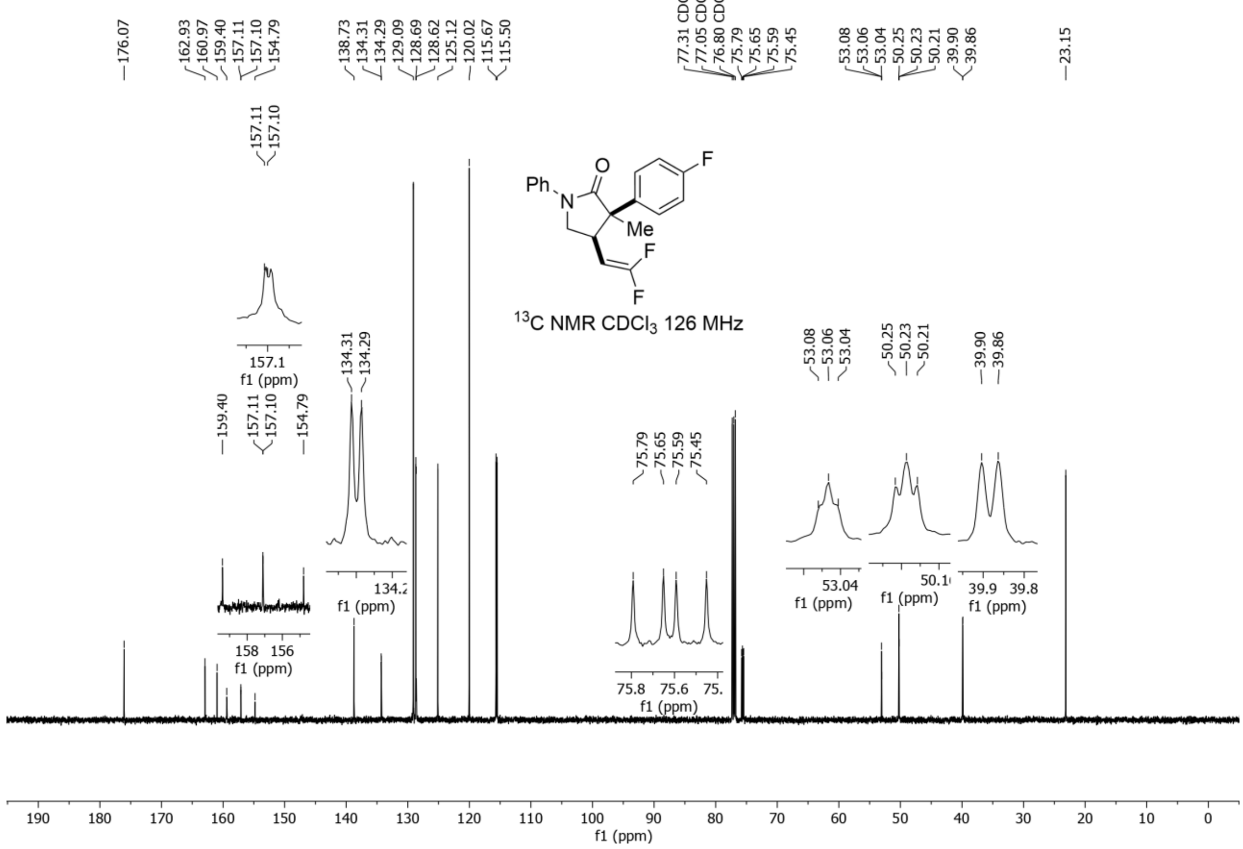

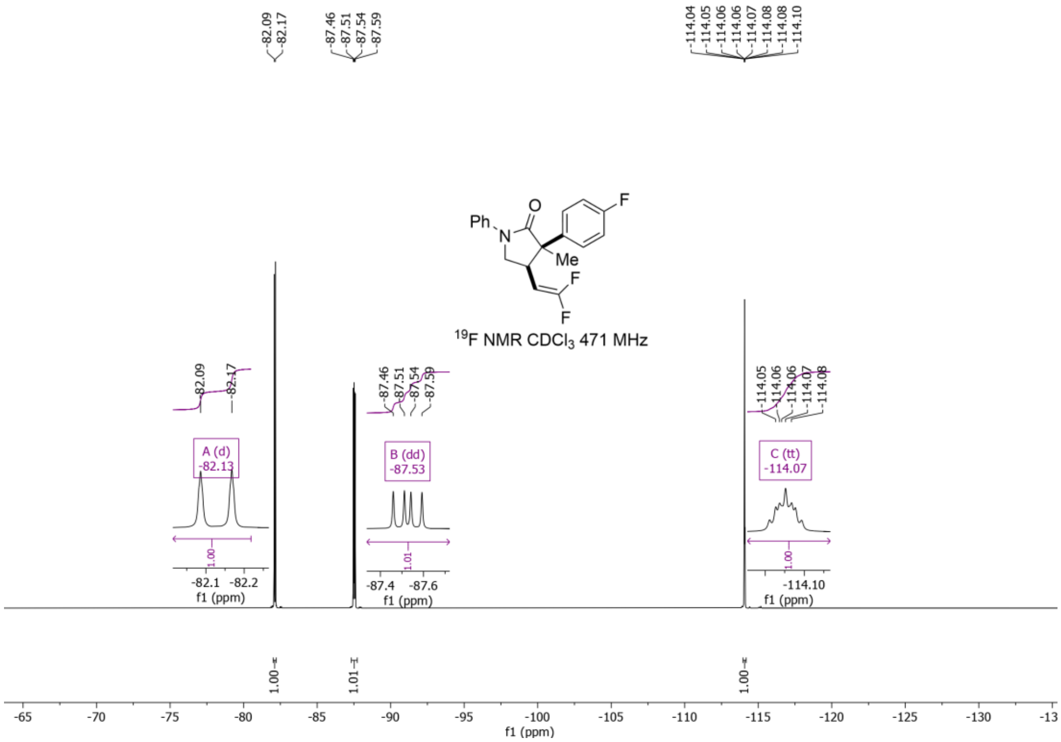

cis-3-(4-chlorophenyl)-4-(2,2-difluorovinyl)-3-methyl-1-phenylpyrrolidin-2-one (cis-3f)

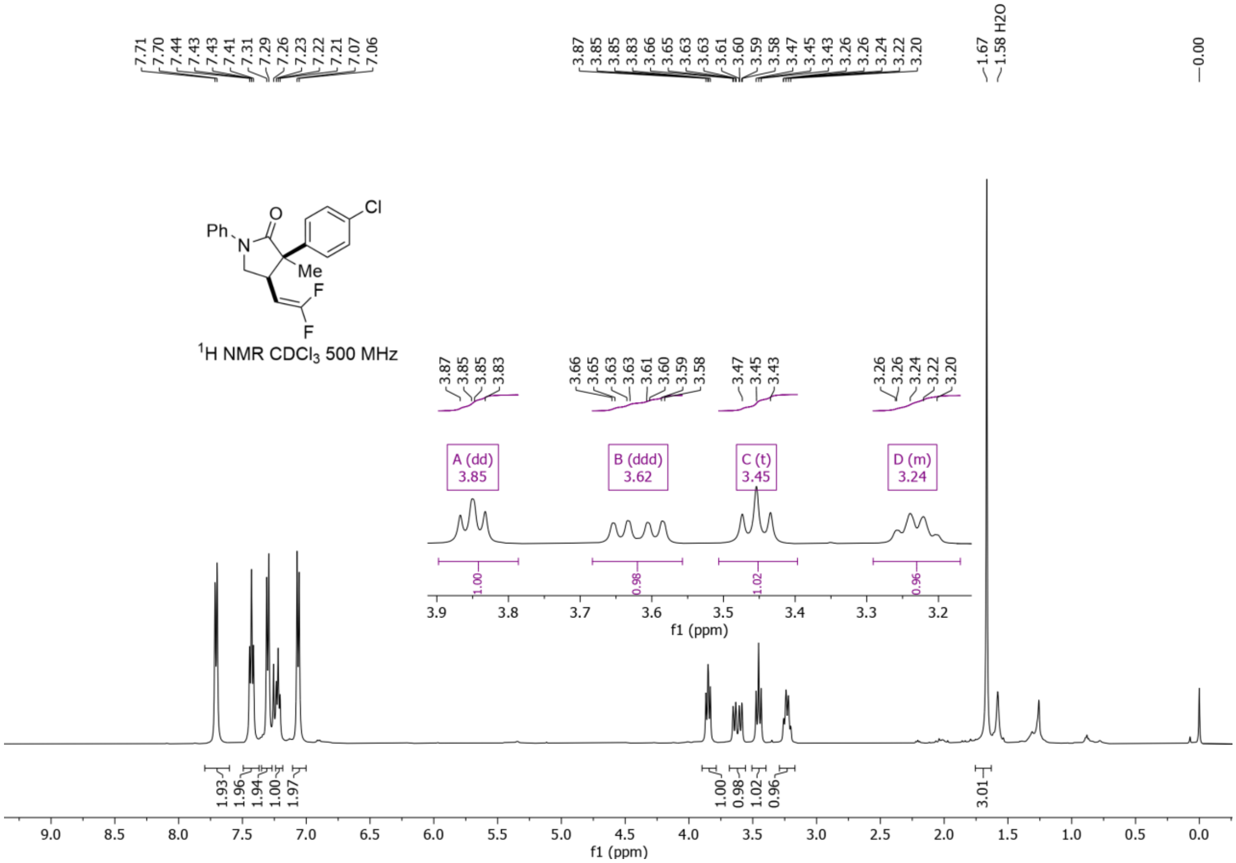

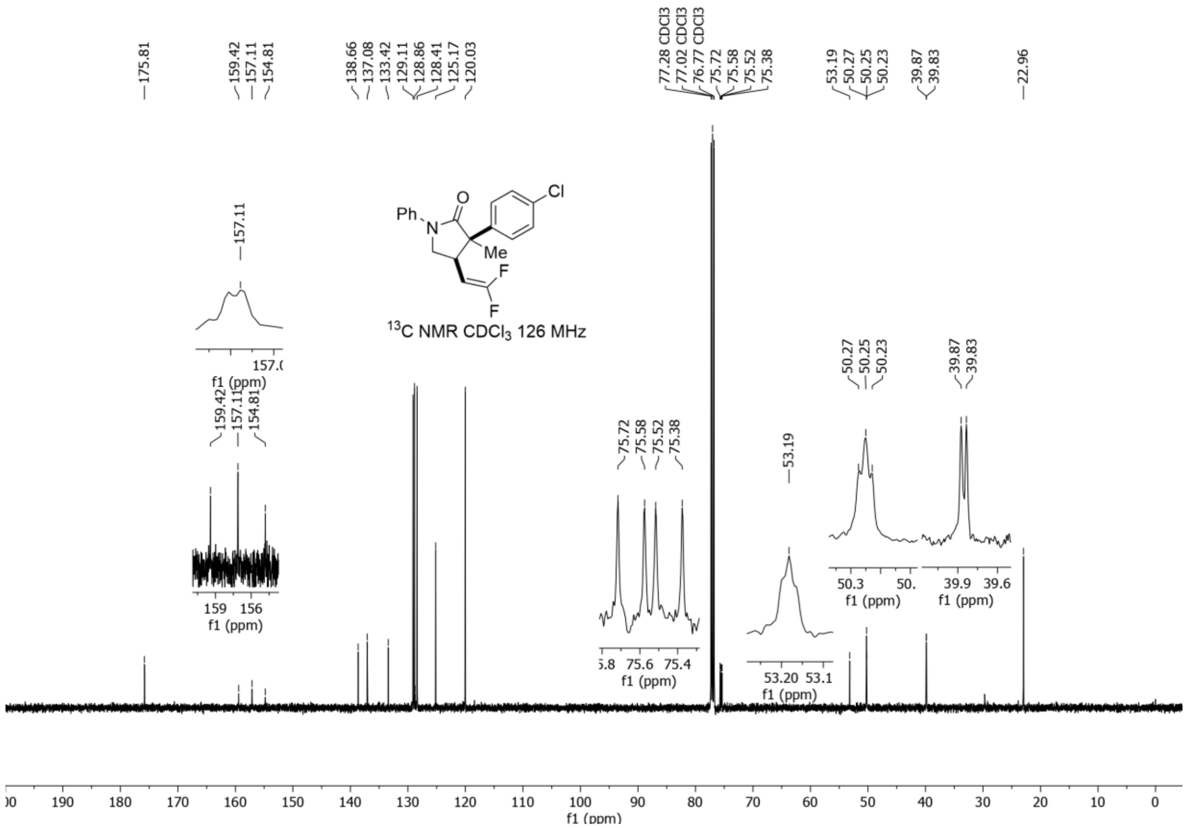

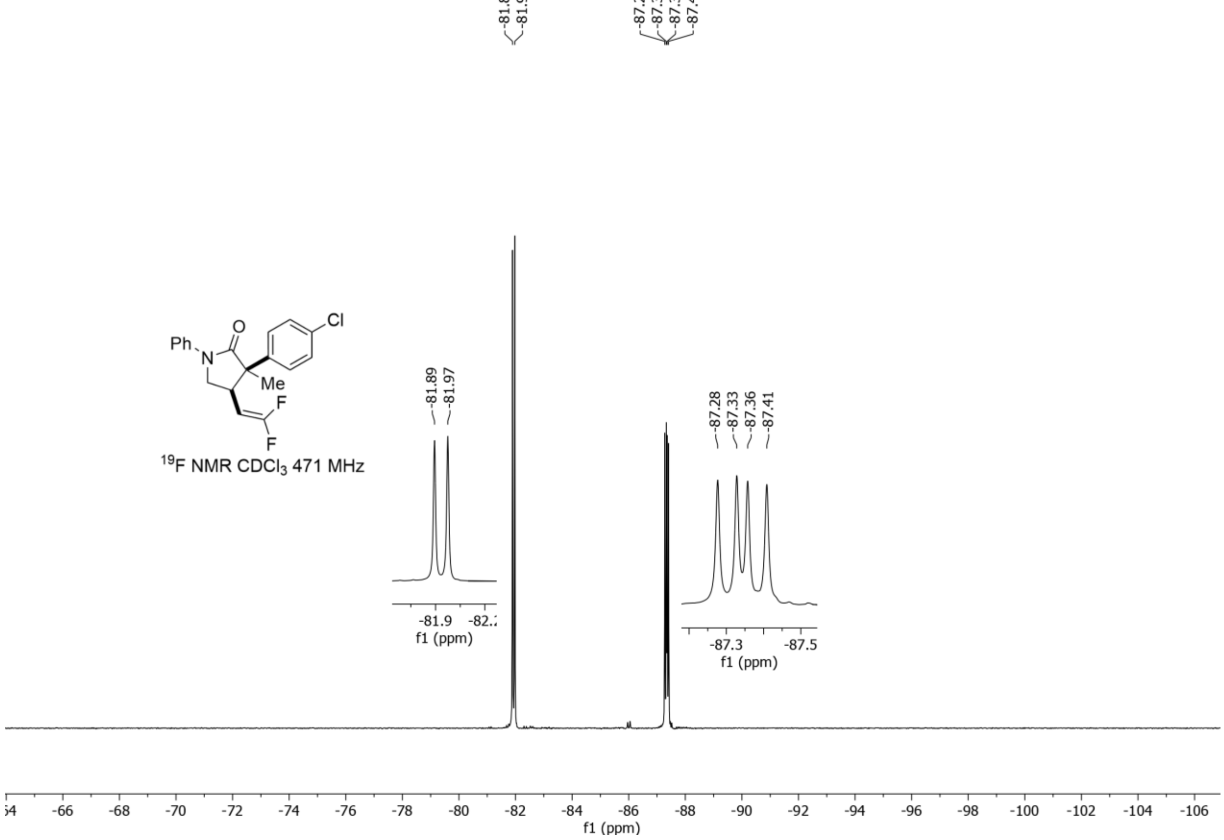

***cis*-3-(4-bromophenyl)-4-(2,2-difluorovinyl)-3-methyl-1-phenylpyrrolidin-2-one (*cis*-3g)**

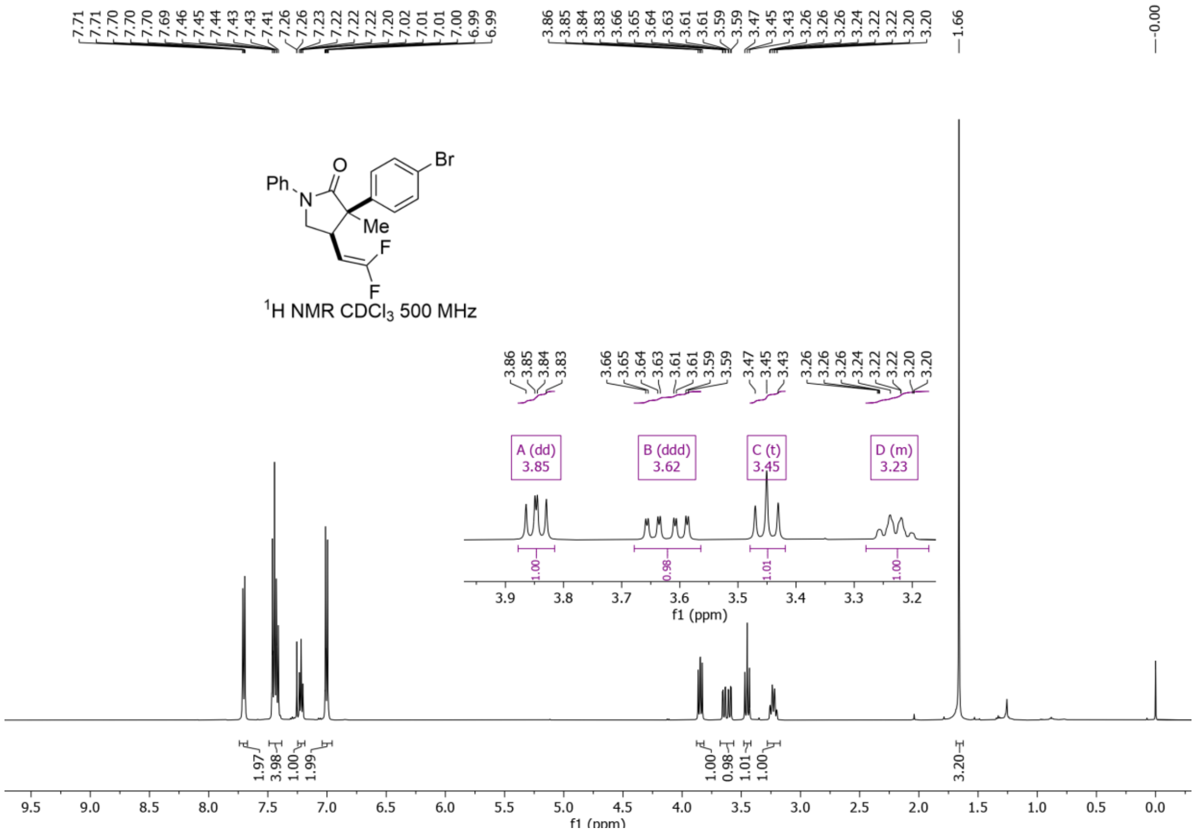

^{1}H NMR CDCl$_3$ 500 MHz
A (dd) 3.85
B (ddd) 3.62
C (t) 3.45
D (m) 3.23
f1 (ppm)

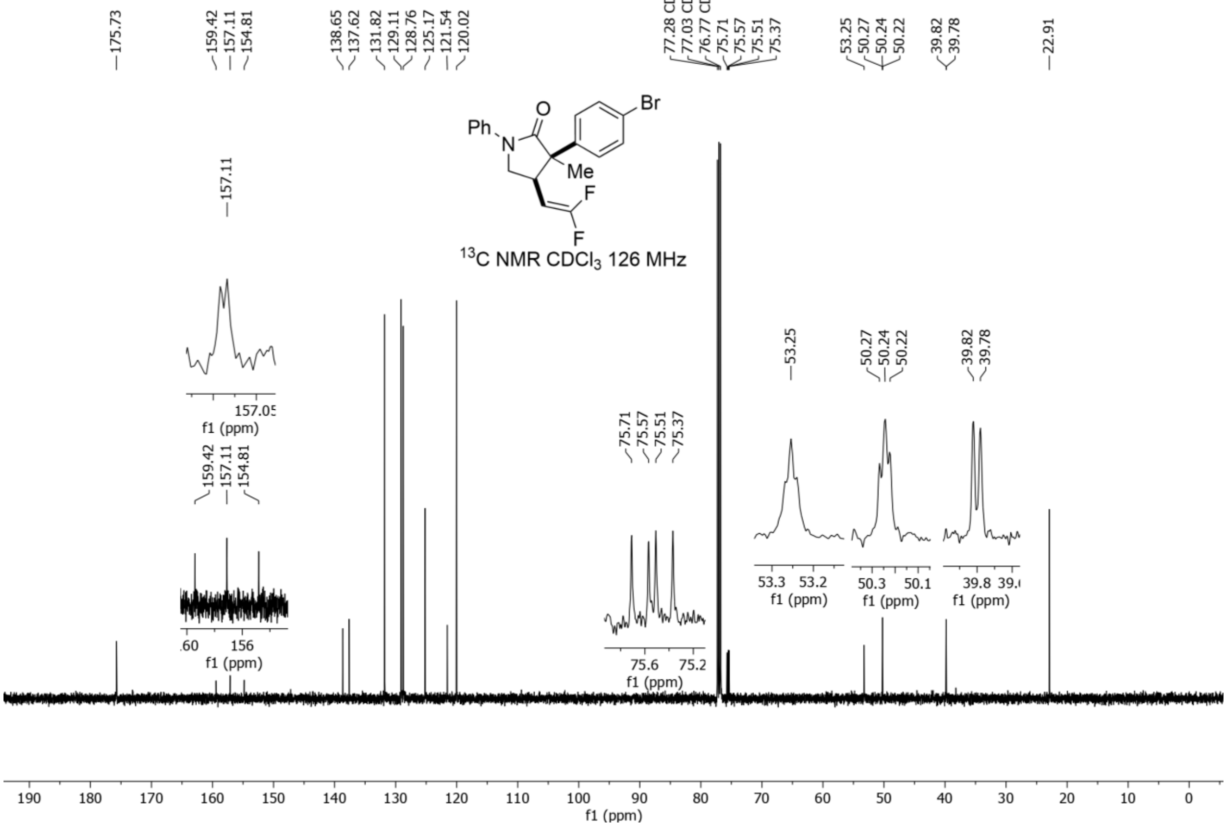

^{13}C NMR CDCl$_3$ 126 MHz
77.28 CDCl3
77.03 CDCl3
76.77 CDCl3
f1 (ppm)

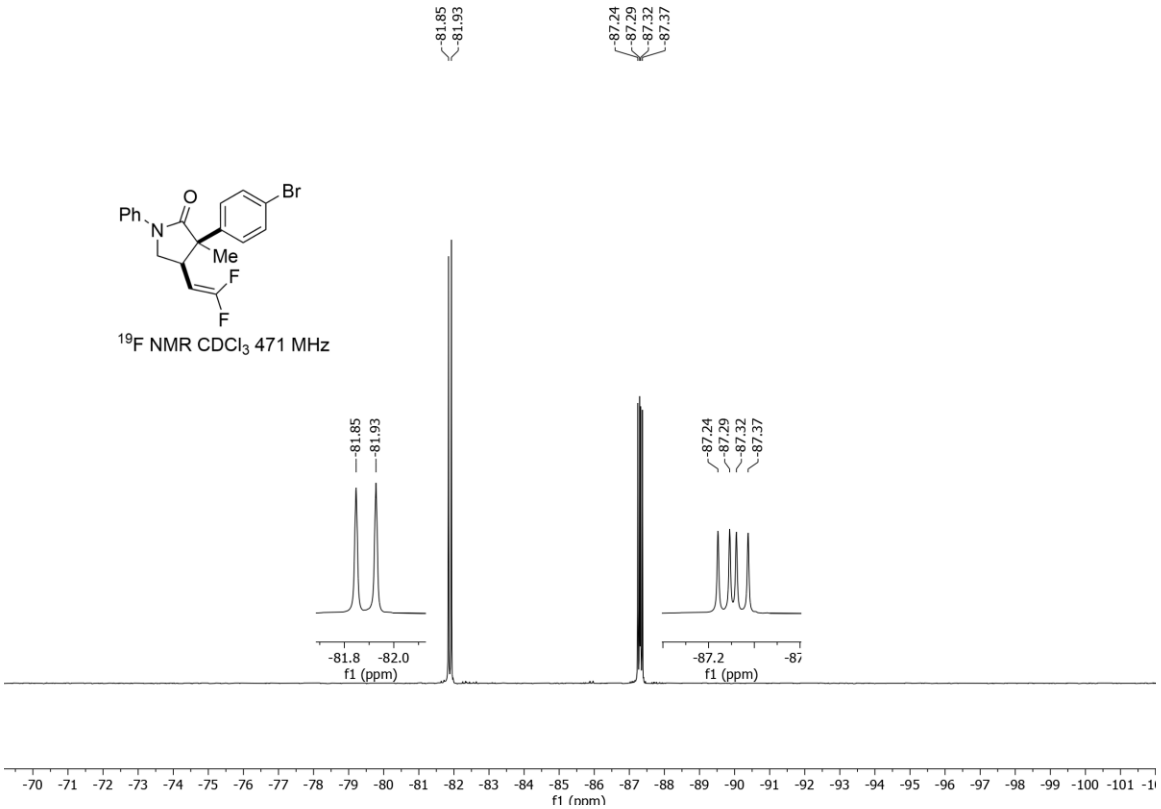

***cis*-3-(2-bromophenyl)-4-(2,2-difluorovinyl)-3-methyl-1-phenylpyrrolidin-2-one (*cis*-3h)**

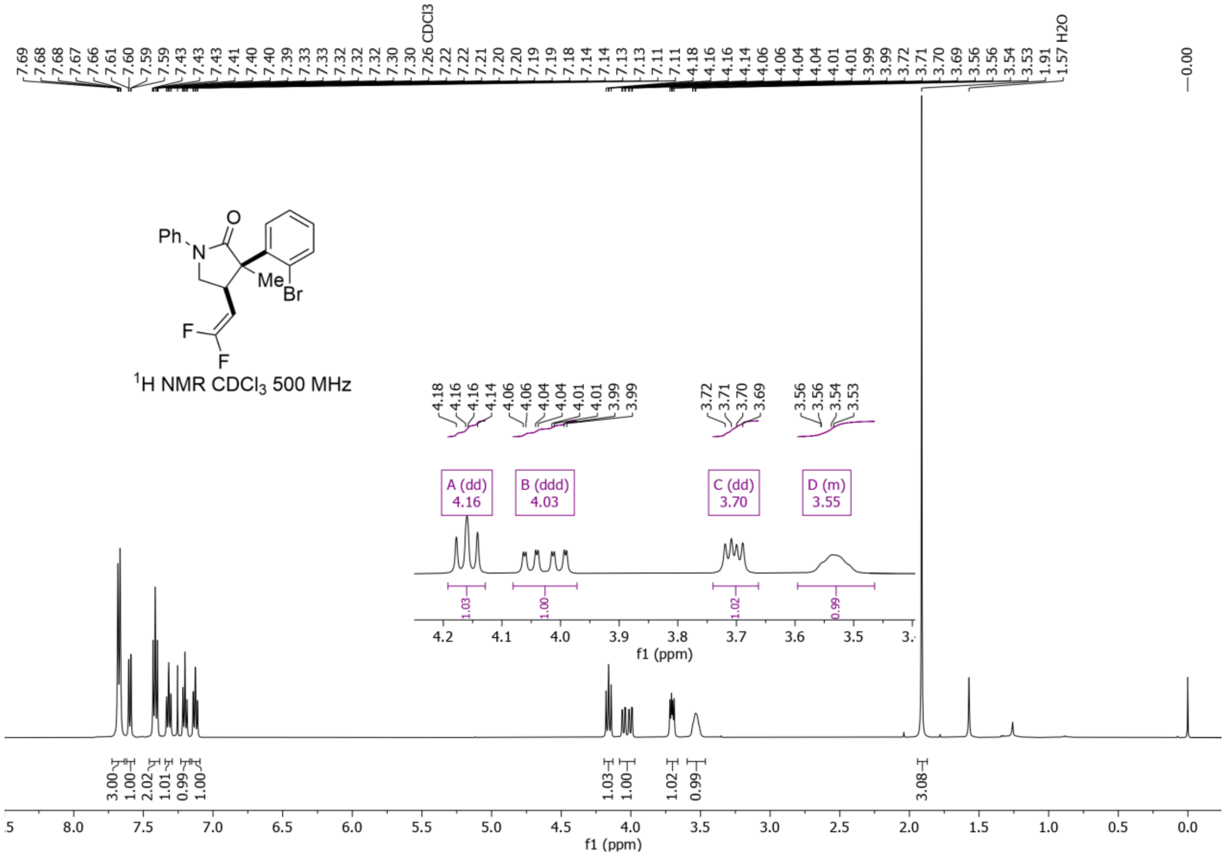

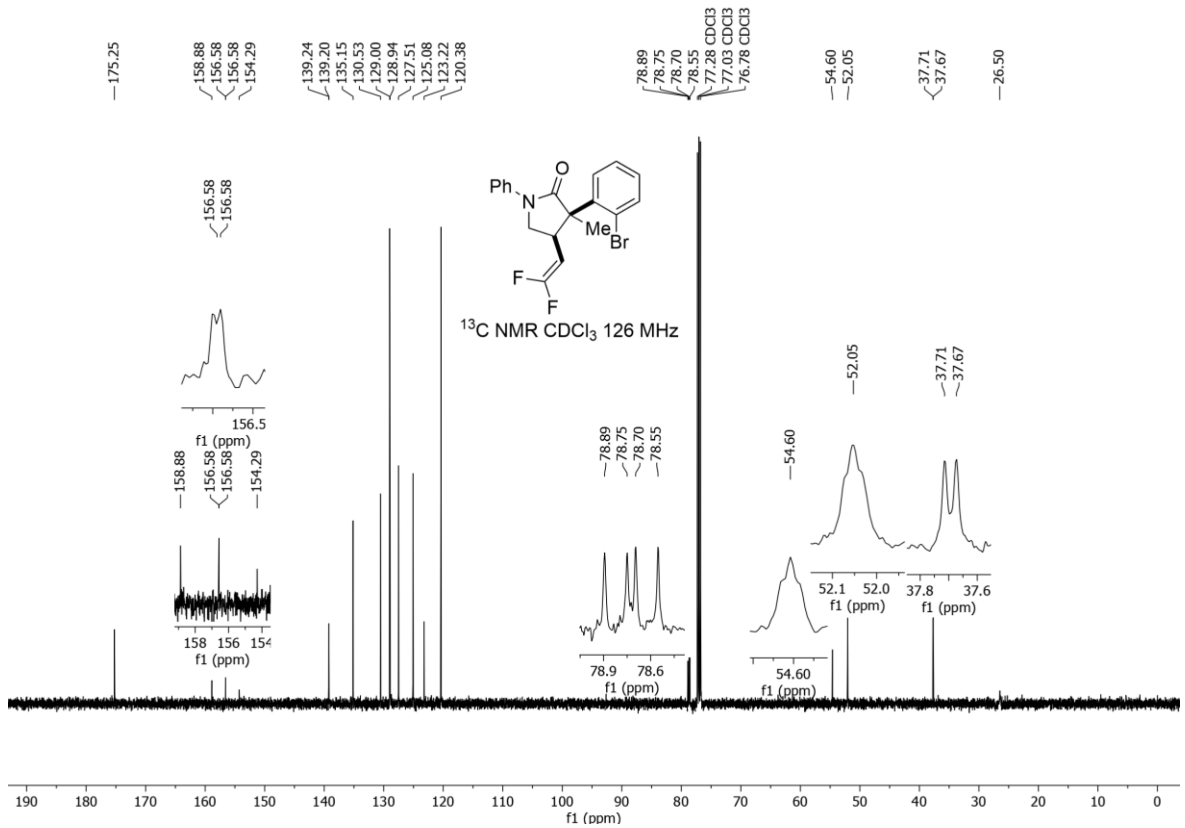

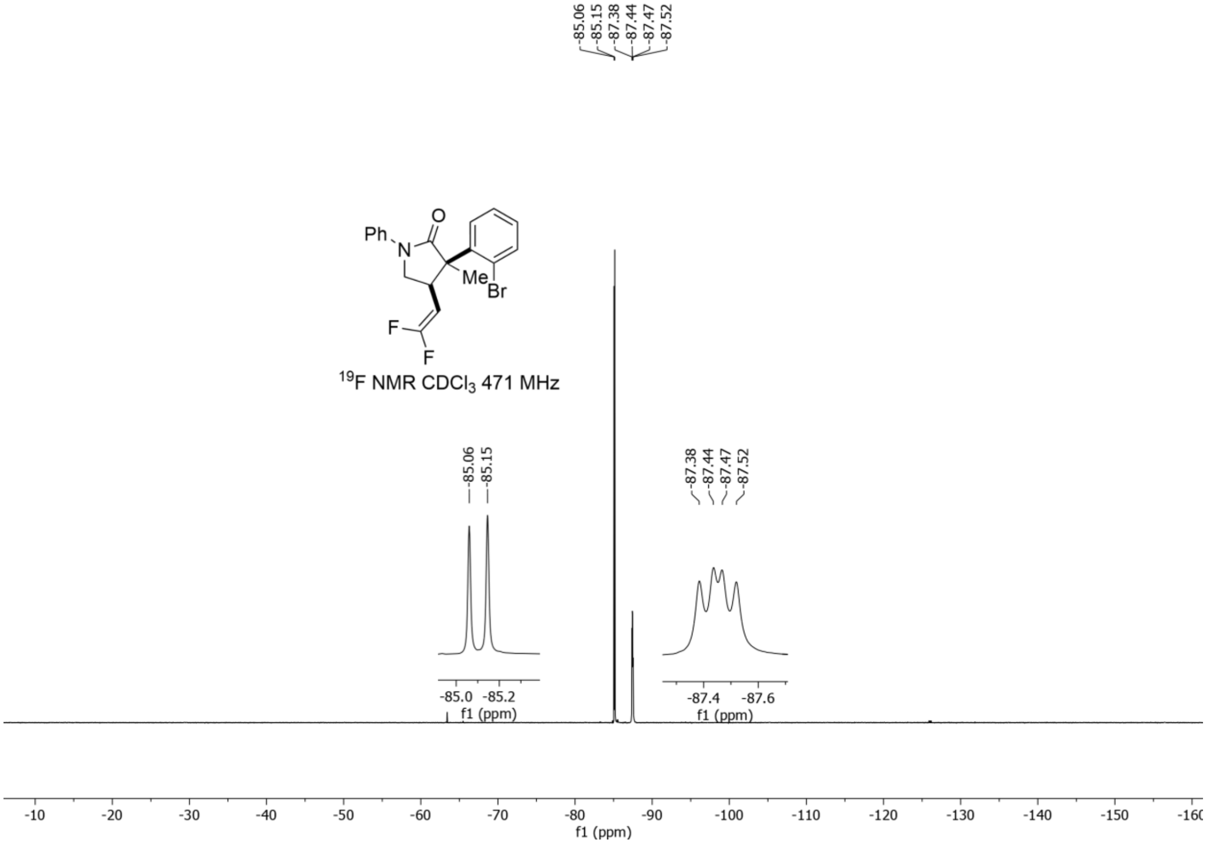

***cis*-4-(2,2-difluorovinyl)-3-methyl-1-phenyl-3-(4-(trifluoromethyl)phenyl)pyrrolidin-2-one (*cis*-3i)**

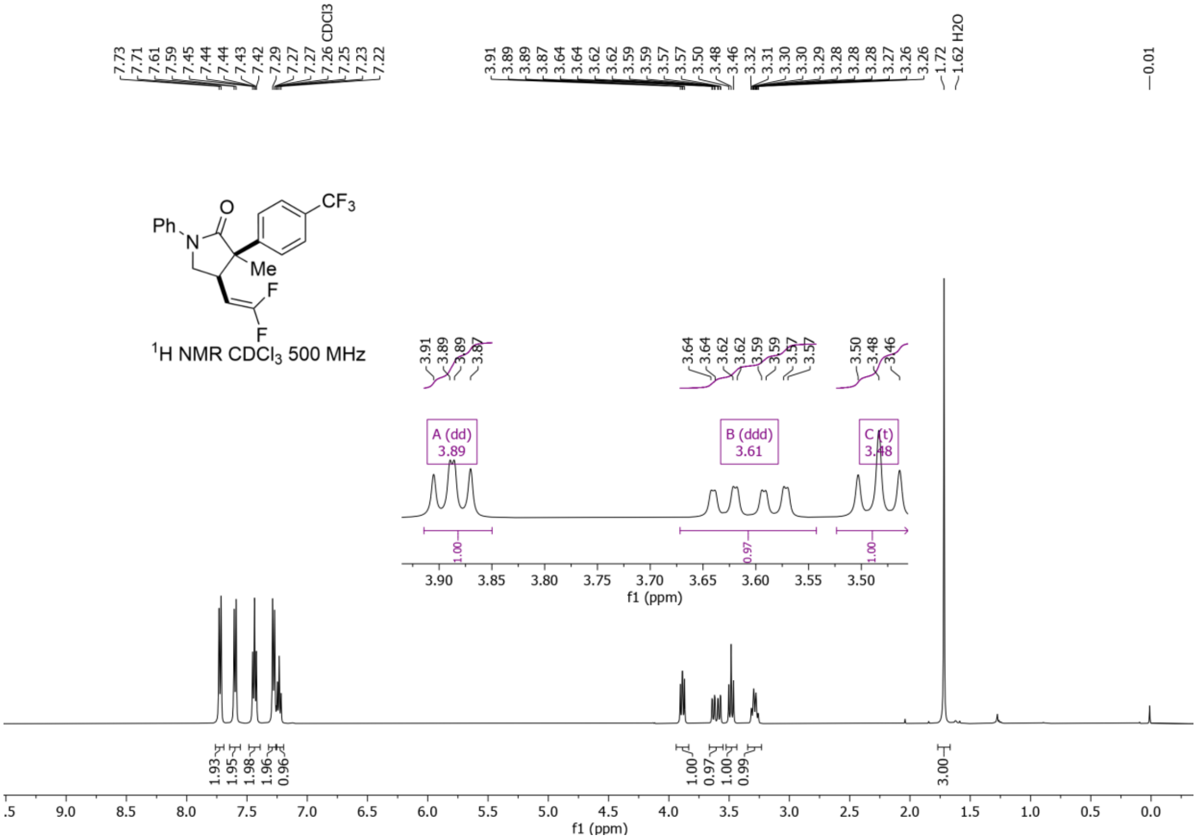

^{1}H NMR CDCl$_3$ 500 MHz
A (dd) 3.89
B (ddd) 3.61
C (t) 3.48
f1 (ppm)

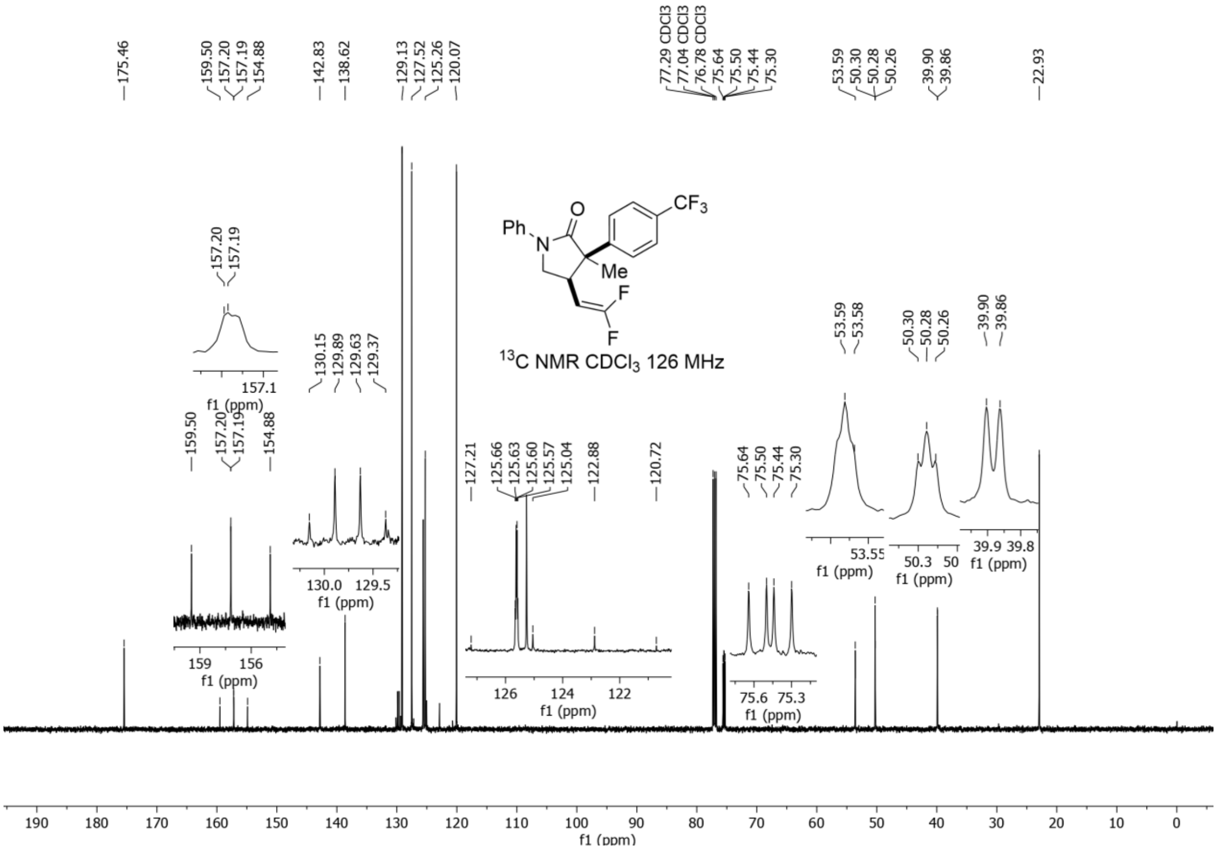

^{13}C NMR CDCl$_3$ 126 MHz
f1 (ppm)

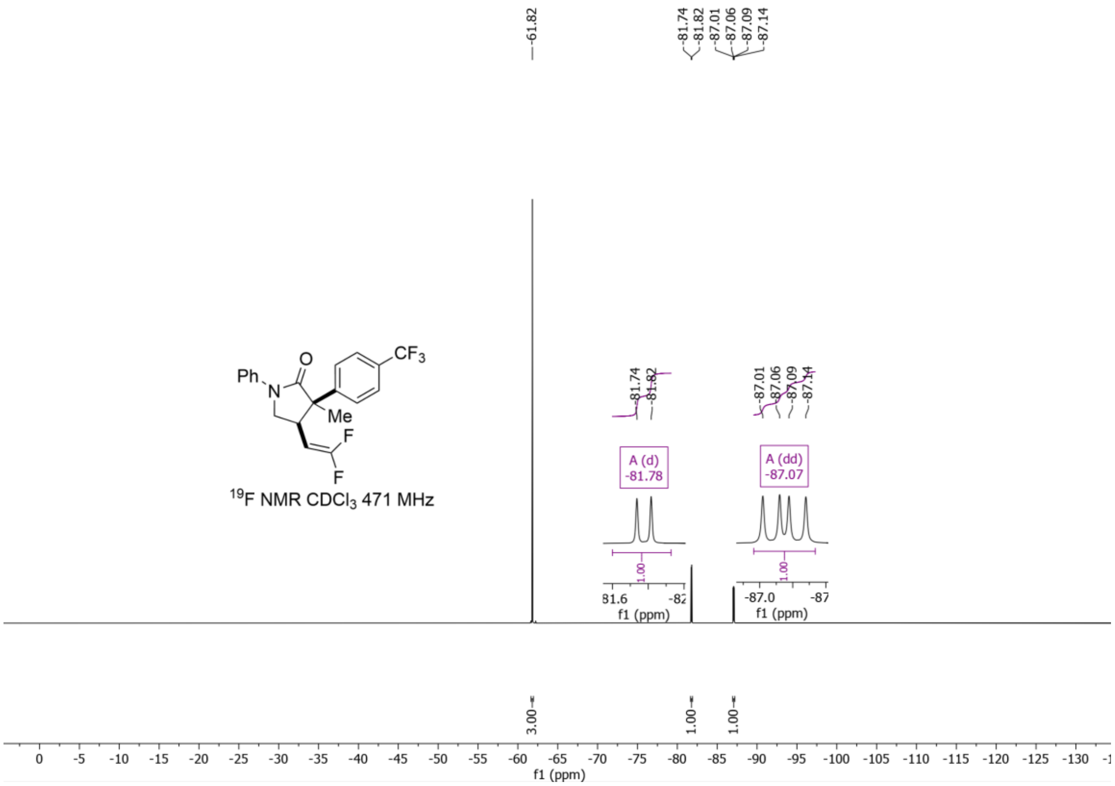

cis-4-(2,2-difluorovinyl)-3-methyl-1-phenyl-3-(3-(trifluoromethyl)phenyl)pyrrolidin-2-one (_cis_-3j)

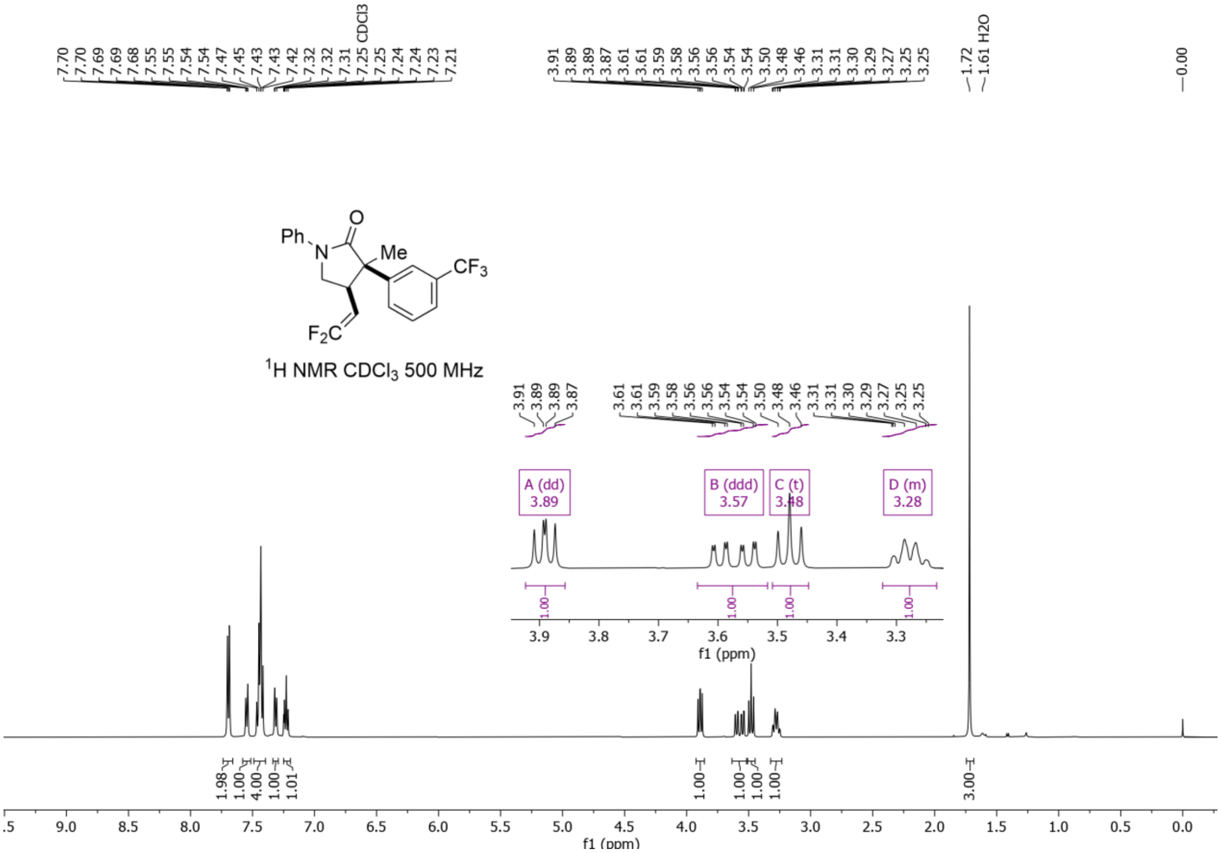

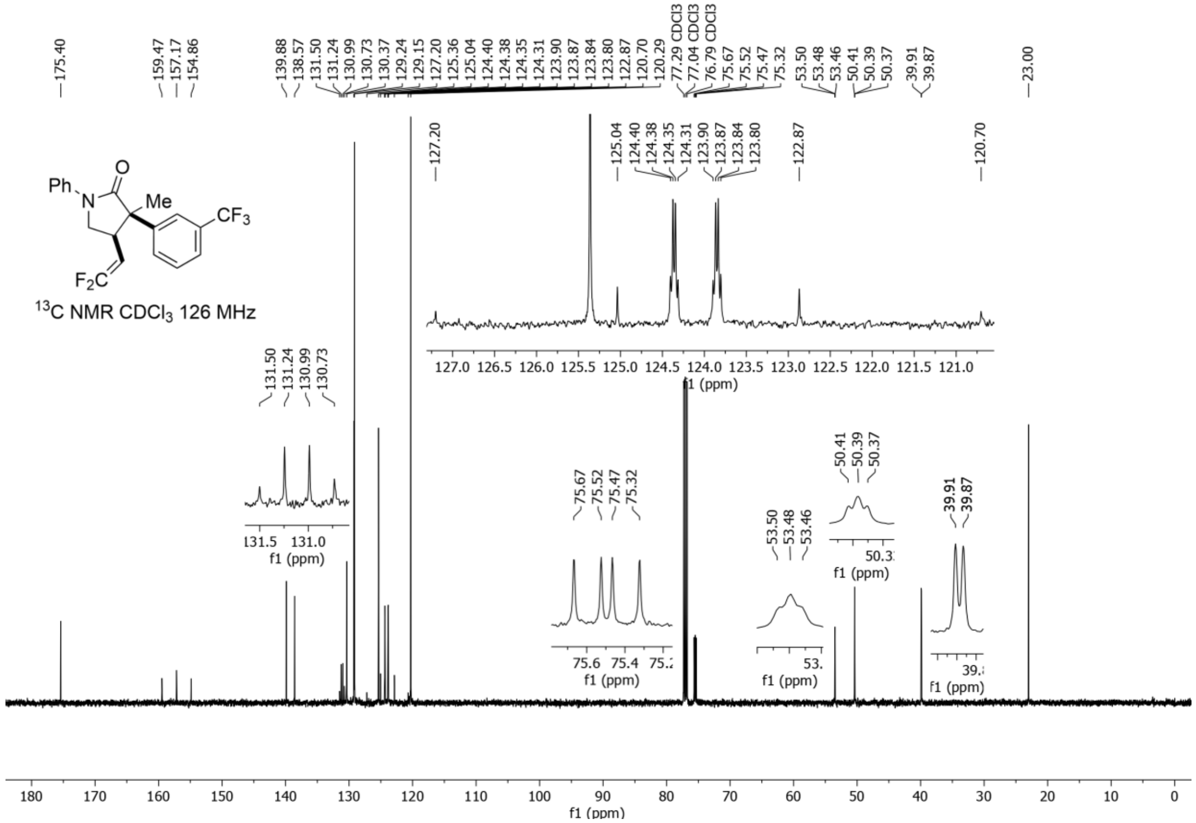

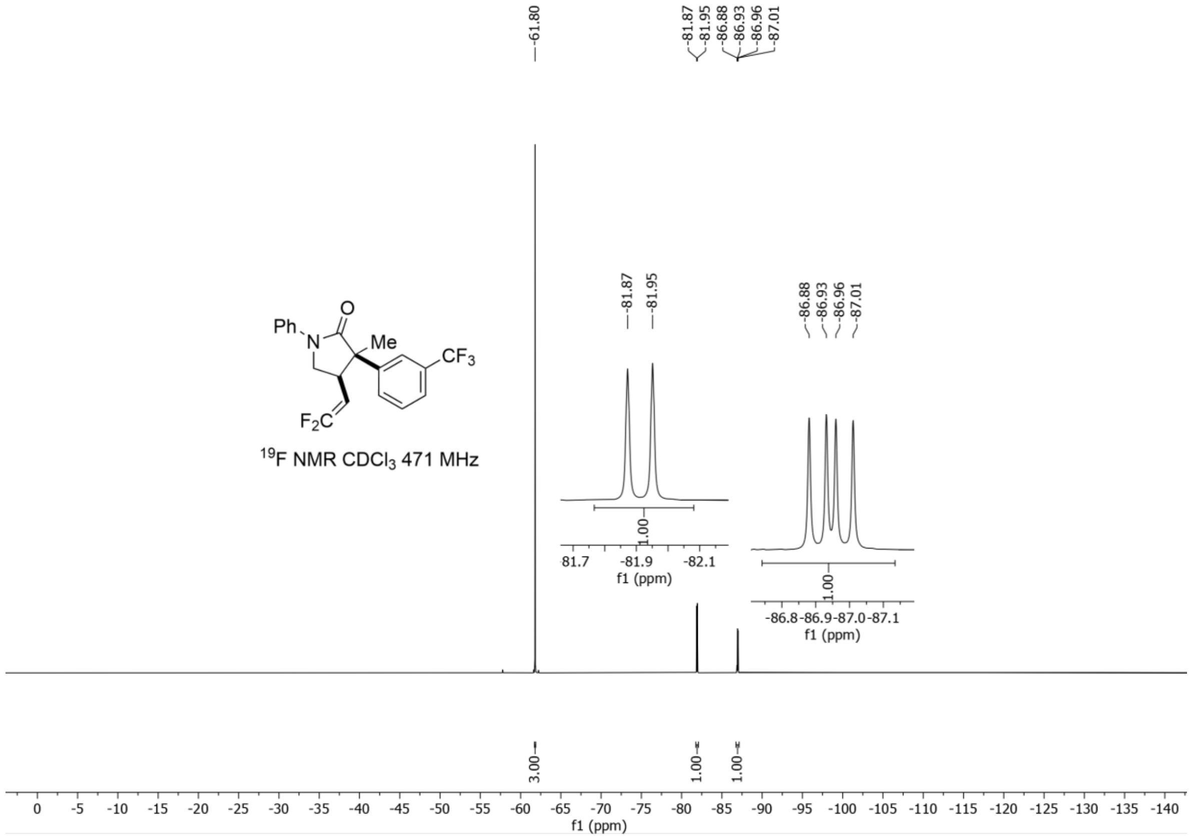

***cis*-4-(2,2-difluorovinyl)-3-methyl-1,3-diphenylpyrrolidin-2-one (*cis*-3k)**

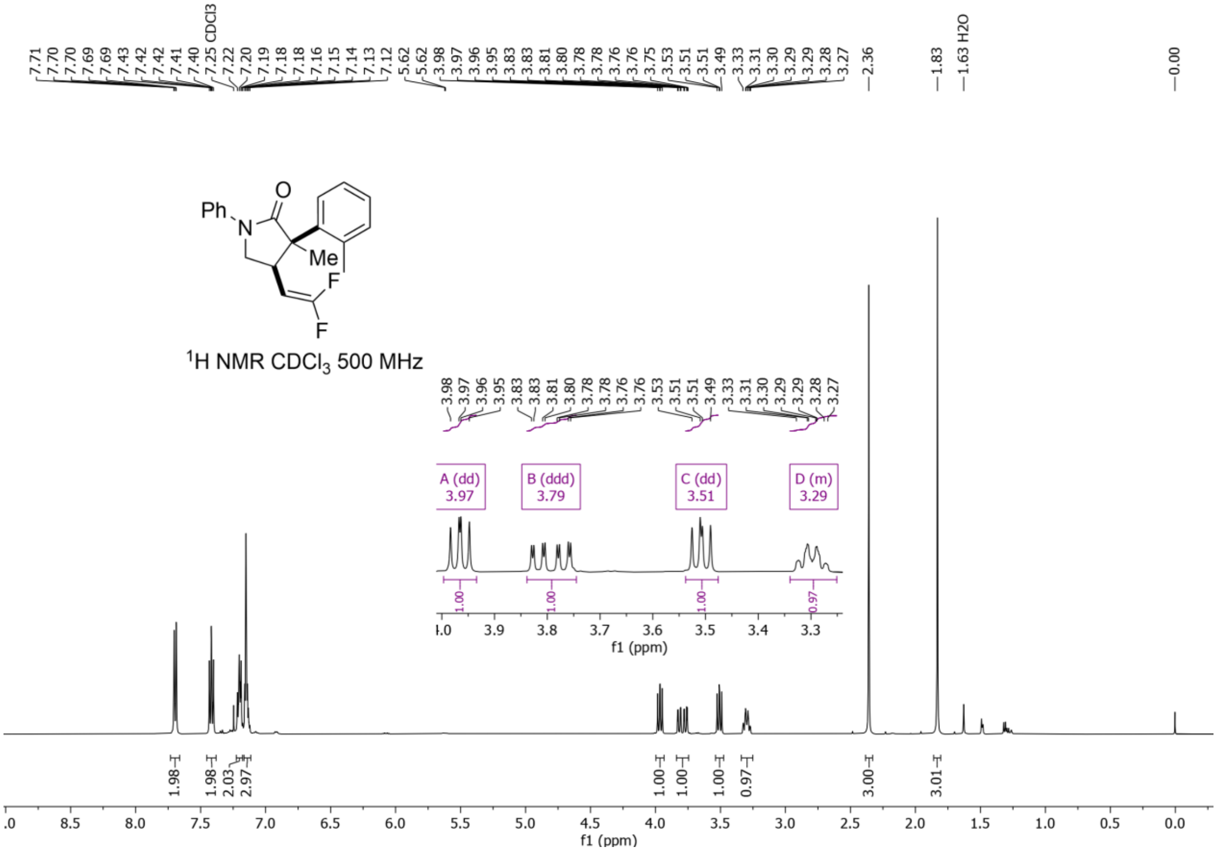

1H NMR CDCl3 500 MHz
Ph
N
O
Me
F
F
A (dd) 3.97
B (ddd) 3.79
C (dd) 3.51
D (m) 3.29
f1 (ppm)

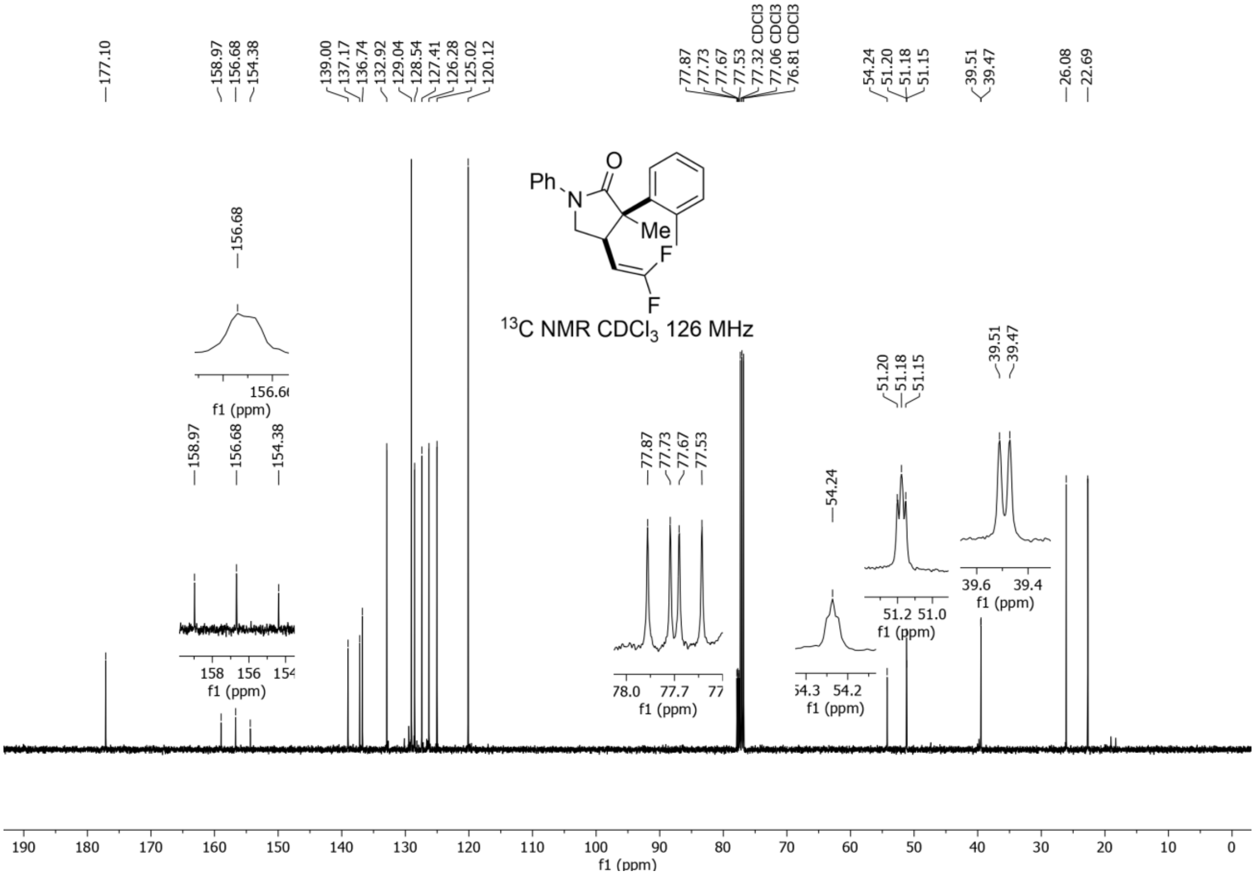

13C NMR CDCl3 126 MHz
Ph
N
O
Me
F
F
f1 (ppm)

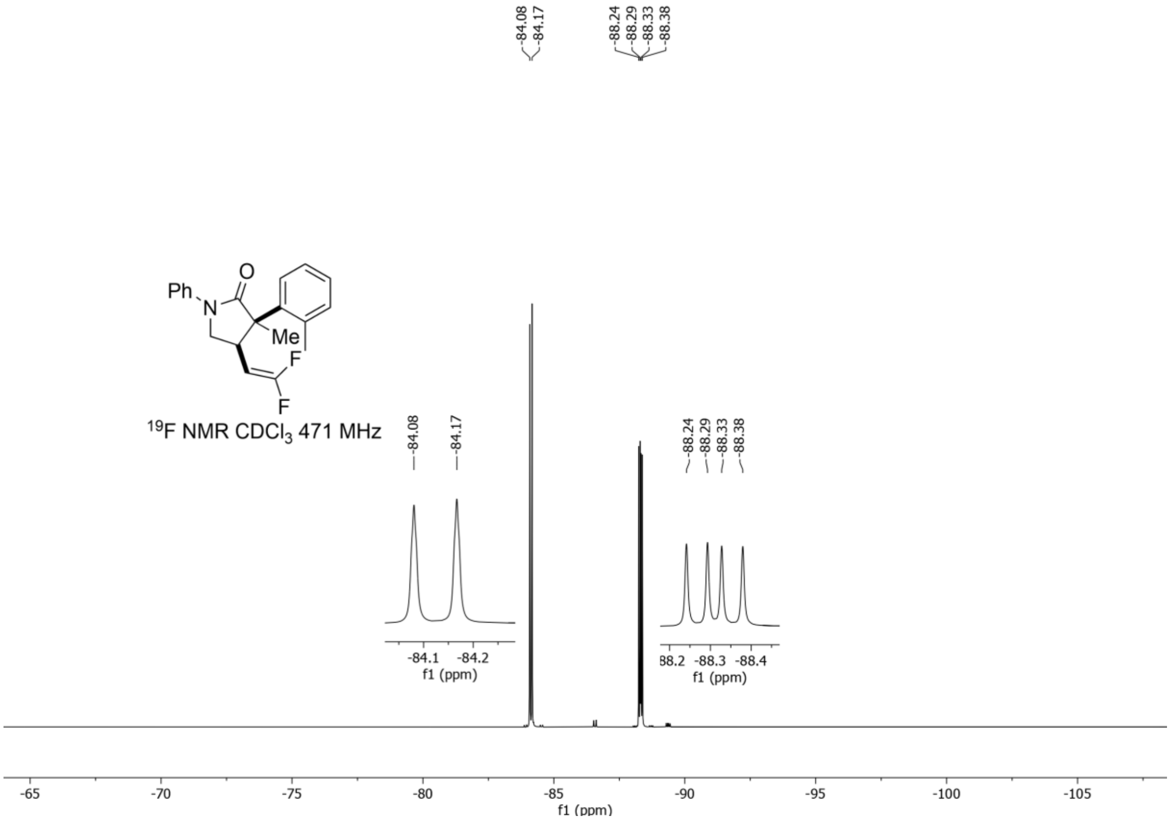

***cis*-4-(2,2-difluorovinyl)-3-methyl-3-(naphthalen-1-yl)-1-phenylpyrrolidin-2-one (*cis*-3l)**

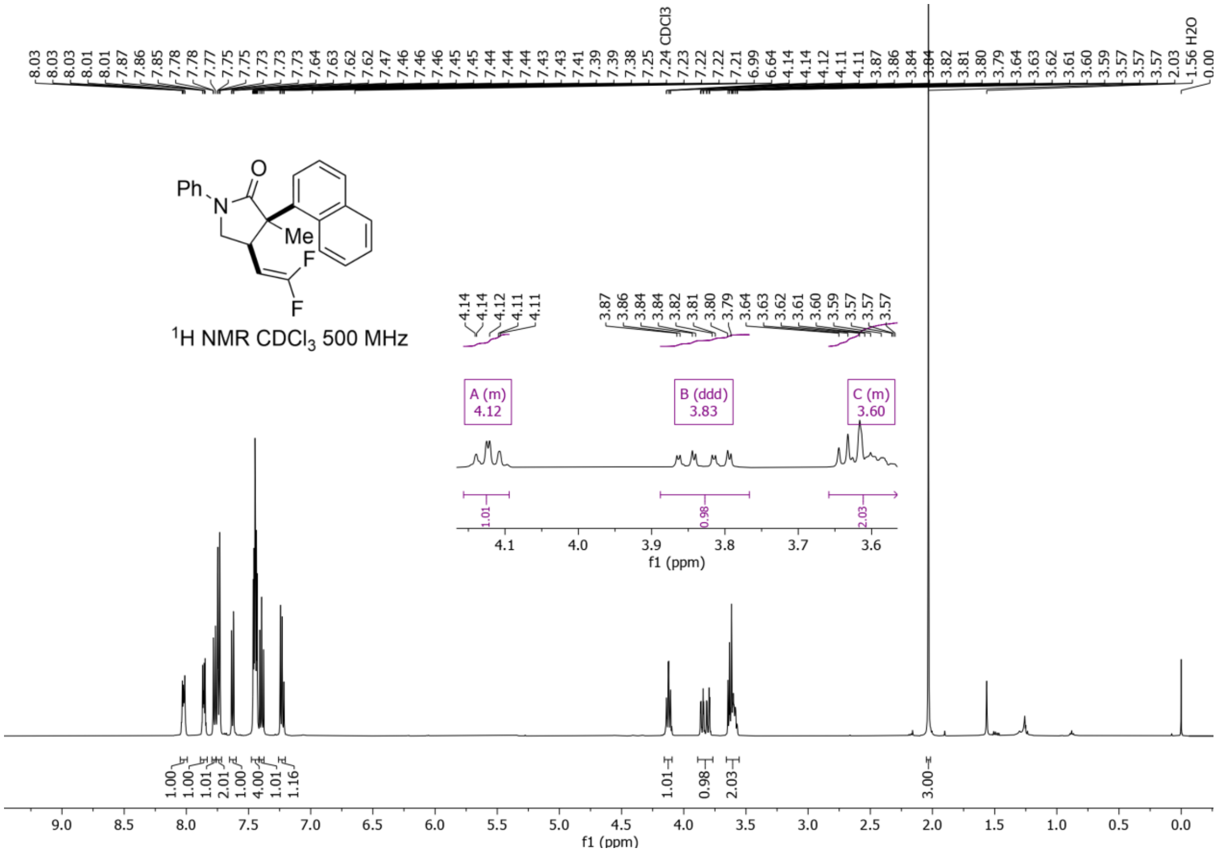

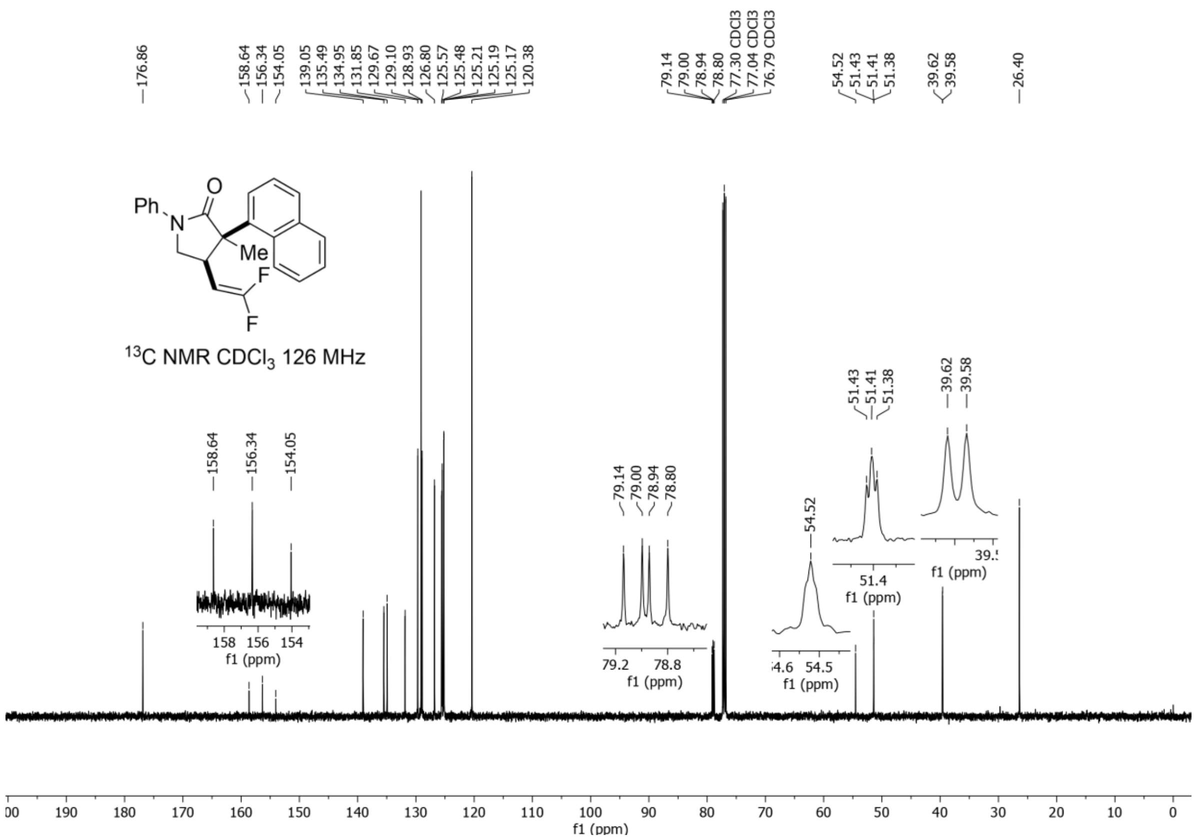

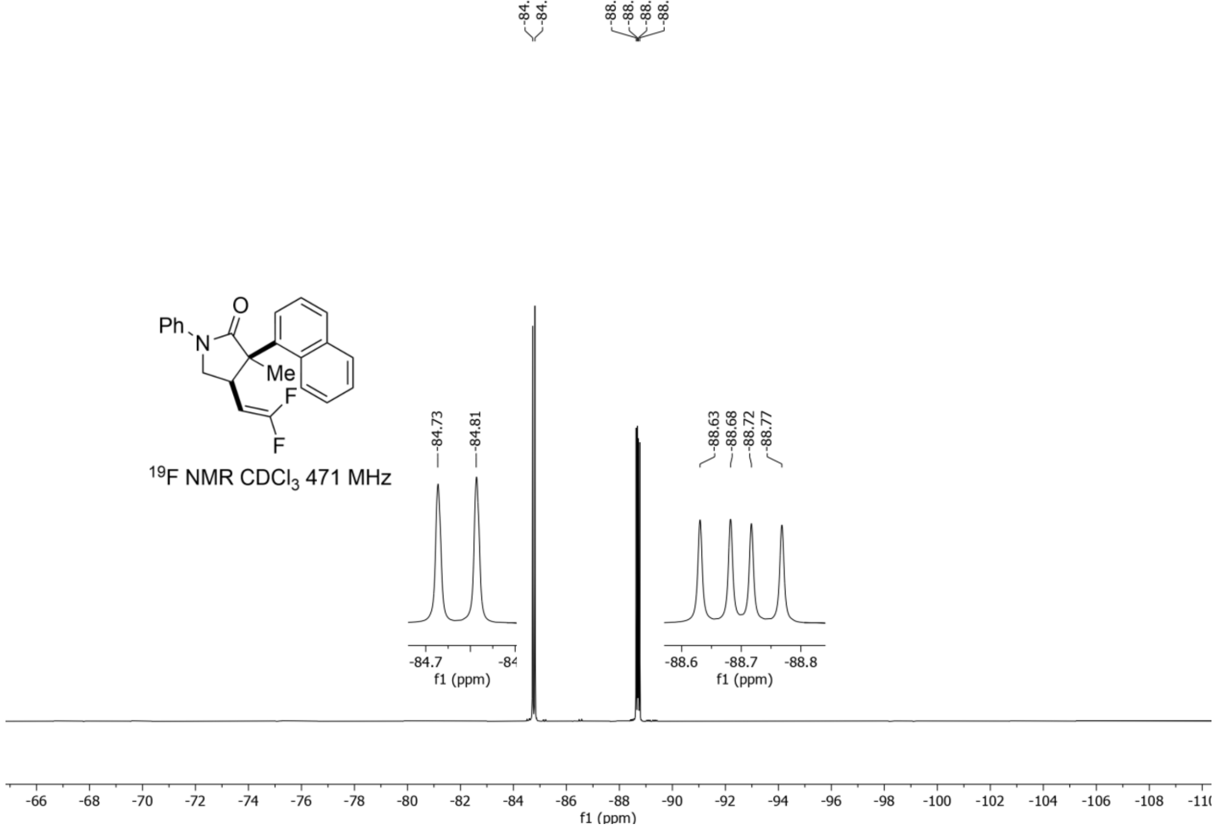

***cis*-4-(2,2-difluorovinyl)-3-(2-fluoro-[1,1'-biphenyl]-4-yl)-3-methyl-1-phenylpyrrolidin-2-one (*cis*-3m)**

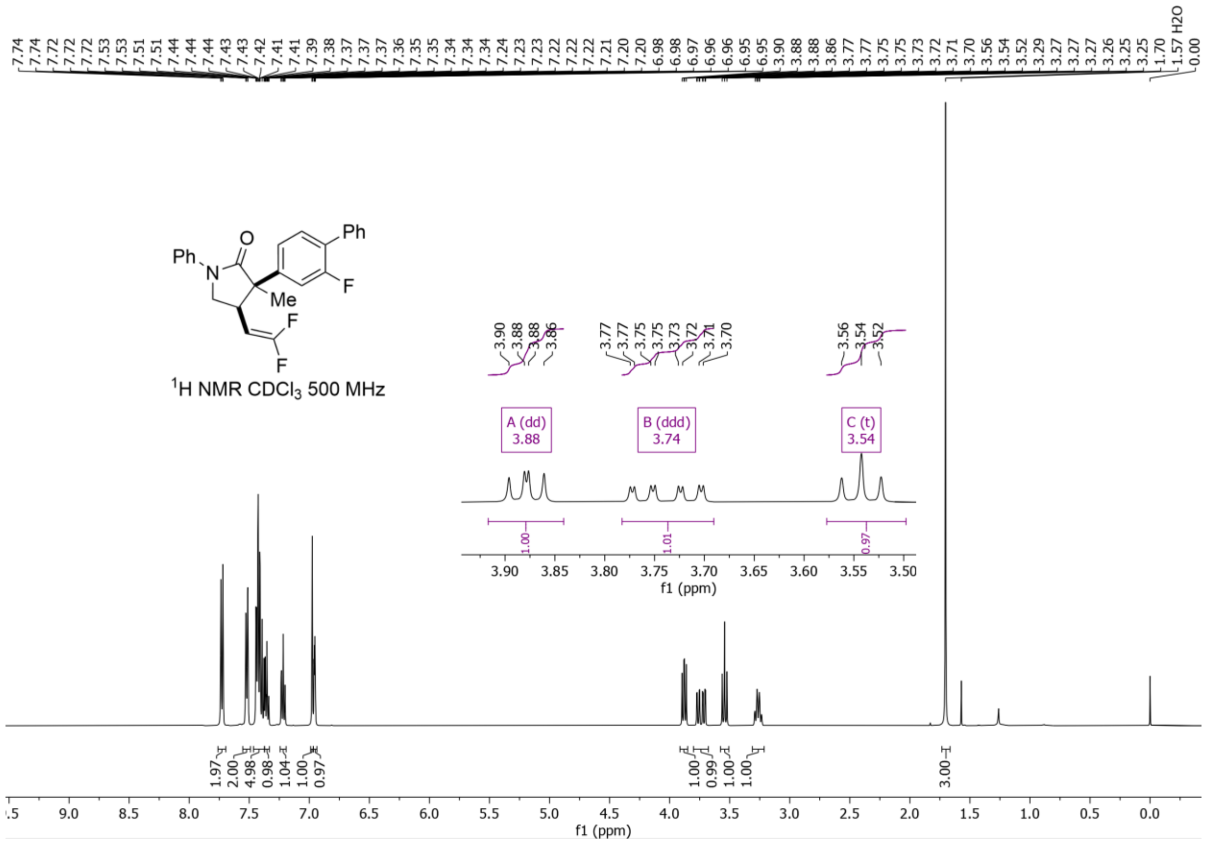

¹H NMR CDCl₃ 500 MHz
A (dd) 3.88
B (ddd) 3.74
C (t) 3.54
f1 (ppm)

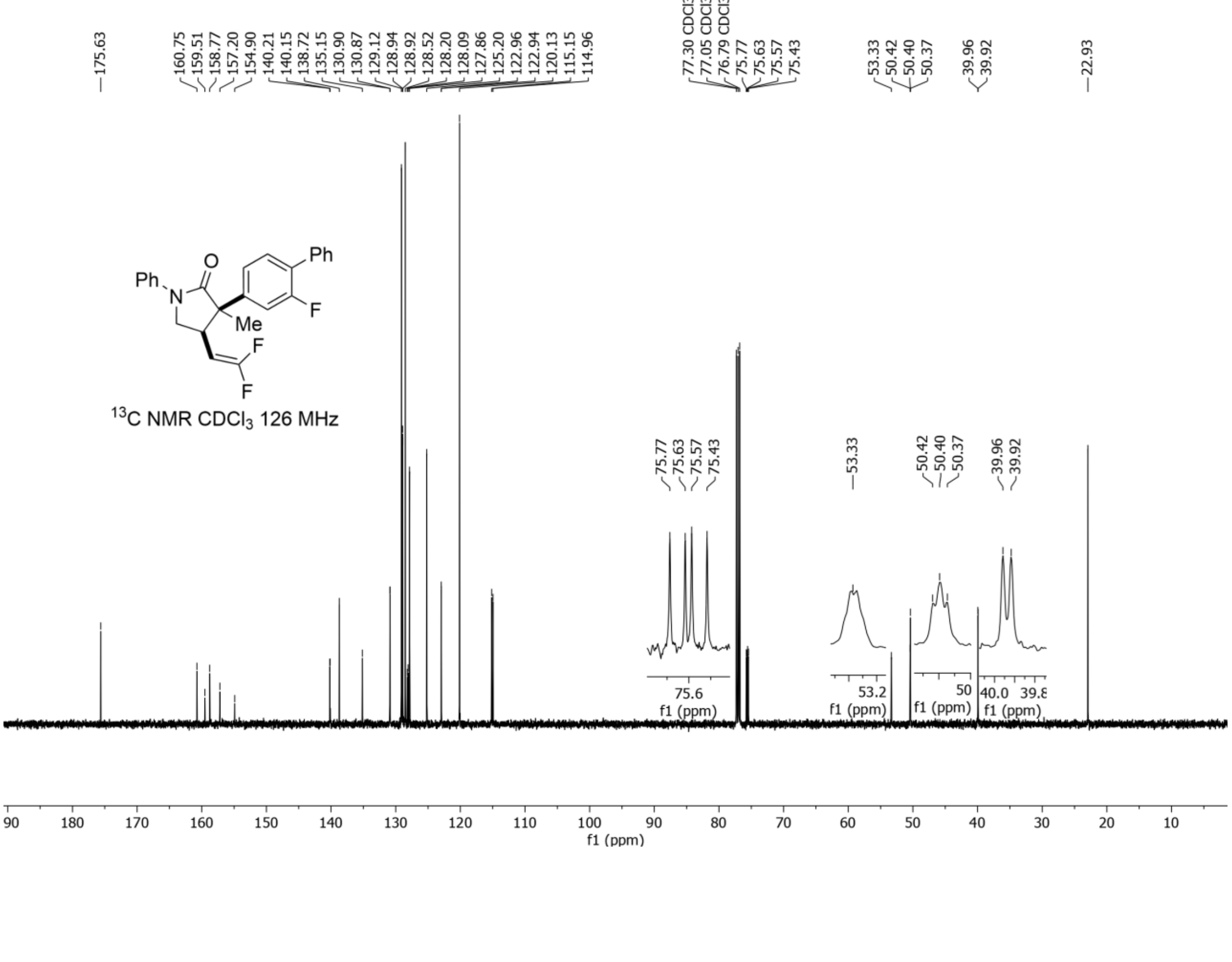

¹³C NMR CDCl₃ 126 MHz
175.63
160.75 159.51 158.77 157.20 154.90
140.21 140.15 138.72 135.15 130.90 130.87 129.12 128.94 128.92 128.52 128.20 128.09 127.86 125.20 122.96 122.94 120.13 115.15 114.96
77.30 CDCl3 77.05 CDCl3 76.79 CDCl3
75.77 75.63 75.57 75.43
53.33
50.42 50.40 50.37
39.96 39.92
22.93
f1 (ppm)

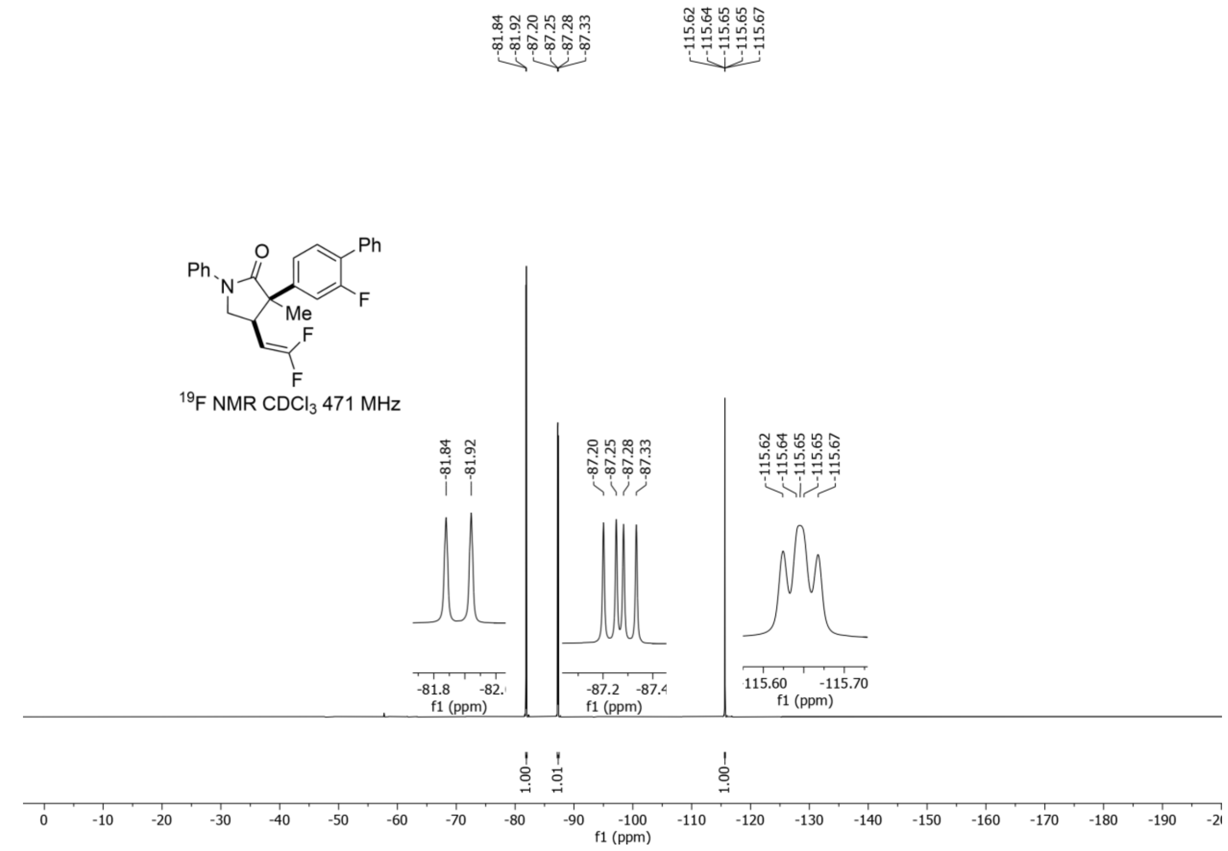

***cis*-4-(2,2-difluorovinyl)-3-methyl-1-phenylpyrrolidin-2-one (*cis*-3n)**

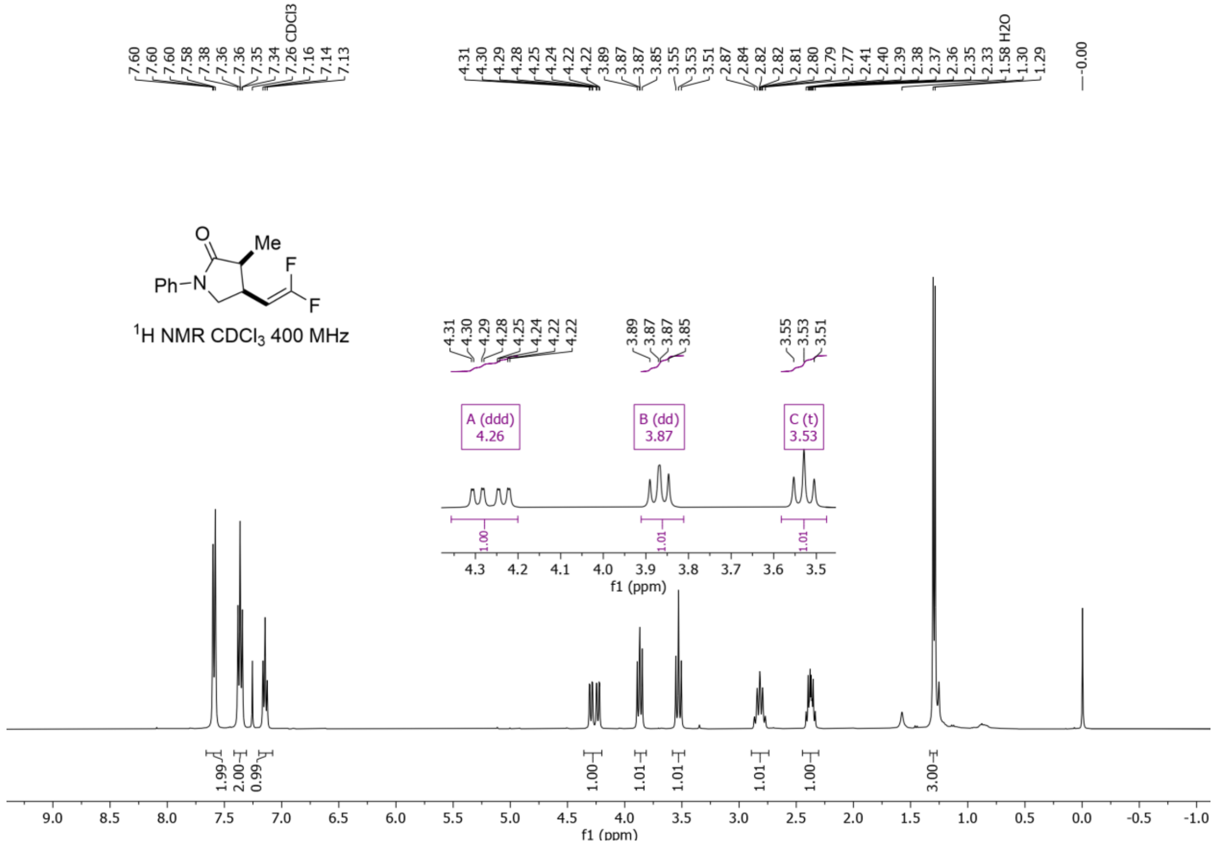

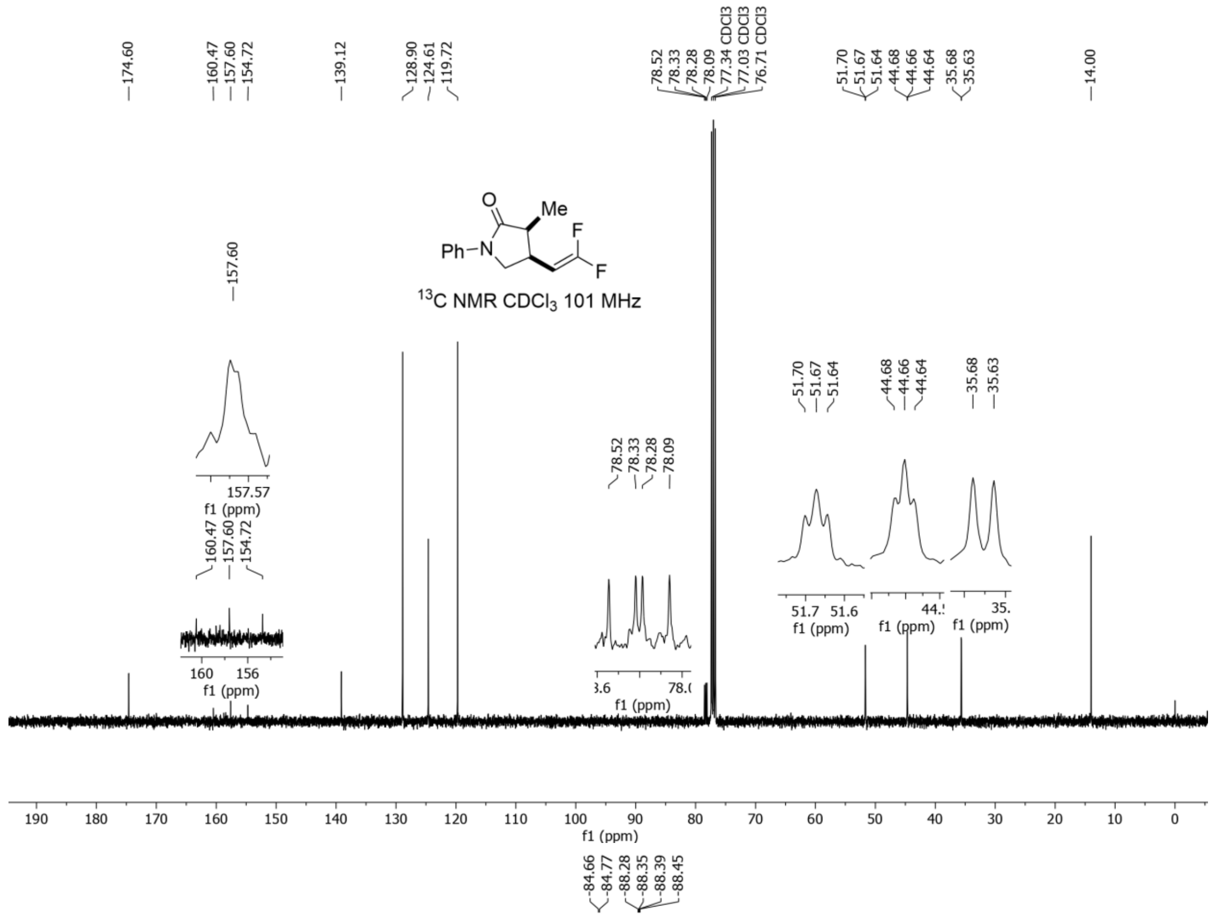
Me
F
Ph–N
F
O
^{13}C NMR CDCl$_3$ 101 MHz
f1 (ppm)

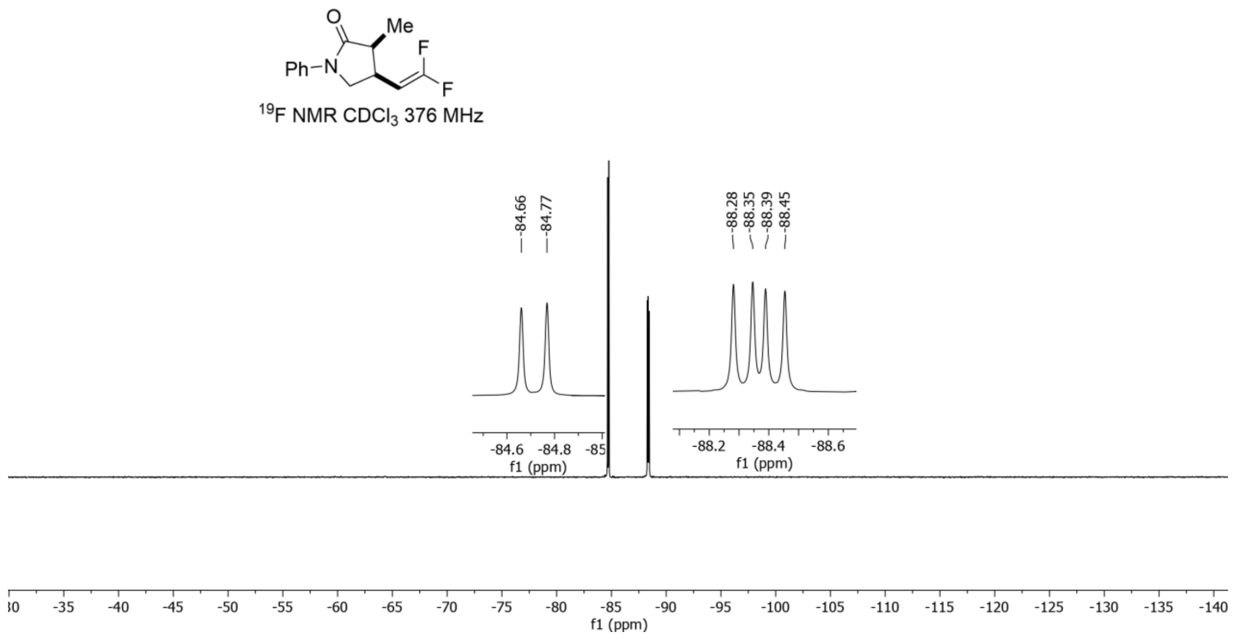
Me
F
Ph–N
F
O
^{19}F NMR CDCl$_3$ 376 MHz
f1 (ppm)

4-(2,2-difluorovinyl)-3,3-dimethyl-1-phenylpyrrolidin-2-one (3o)

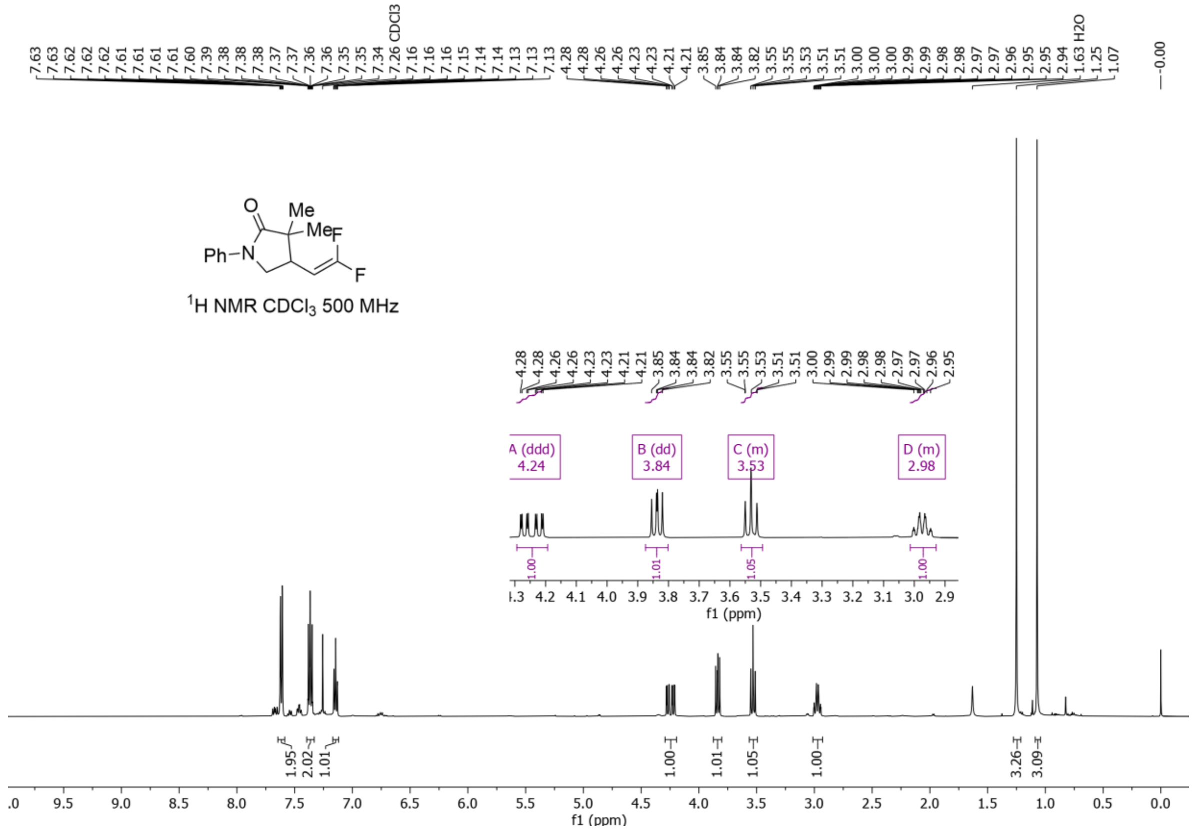

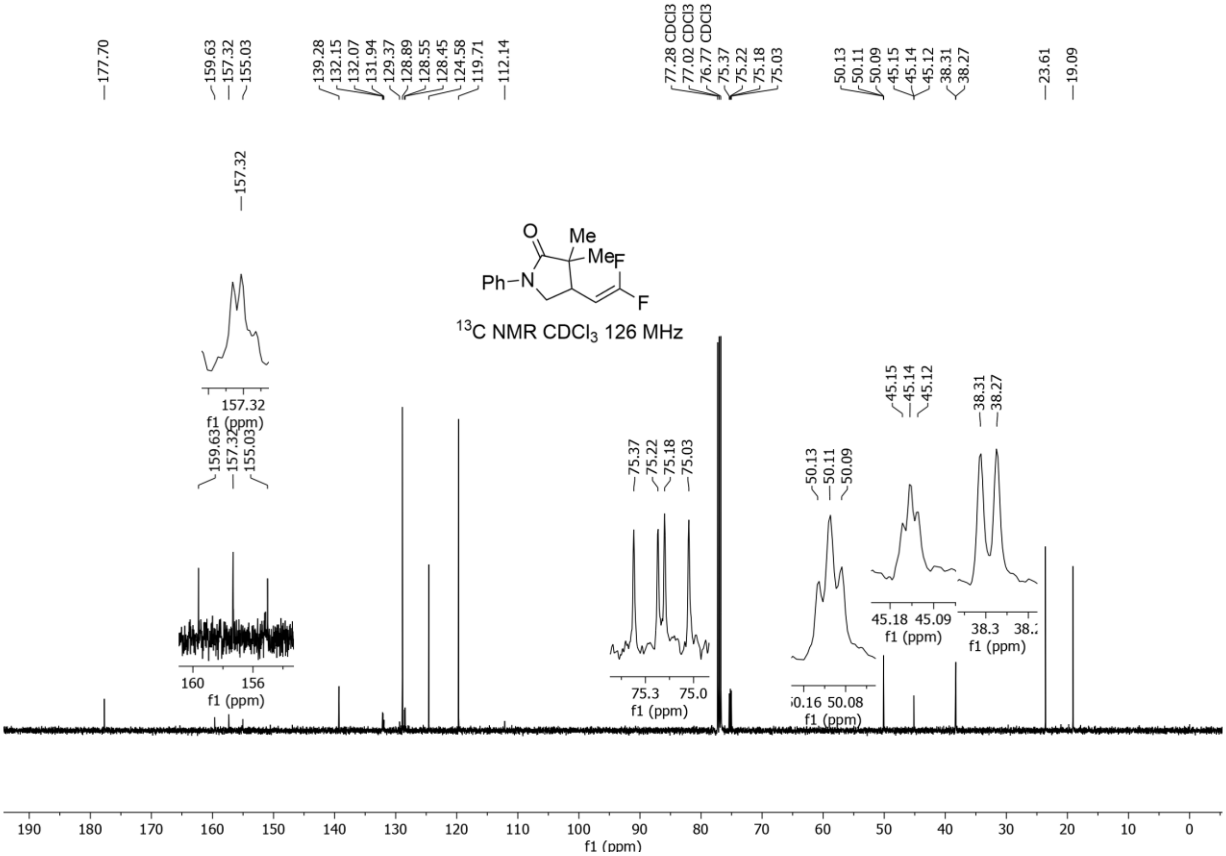

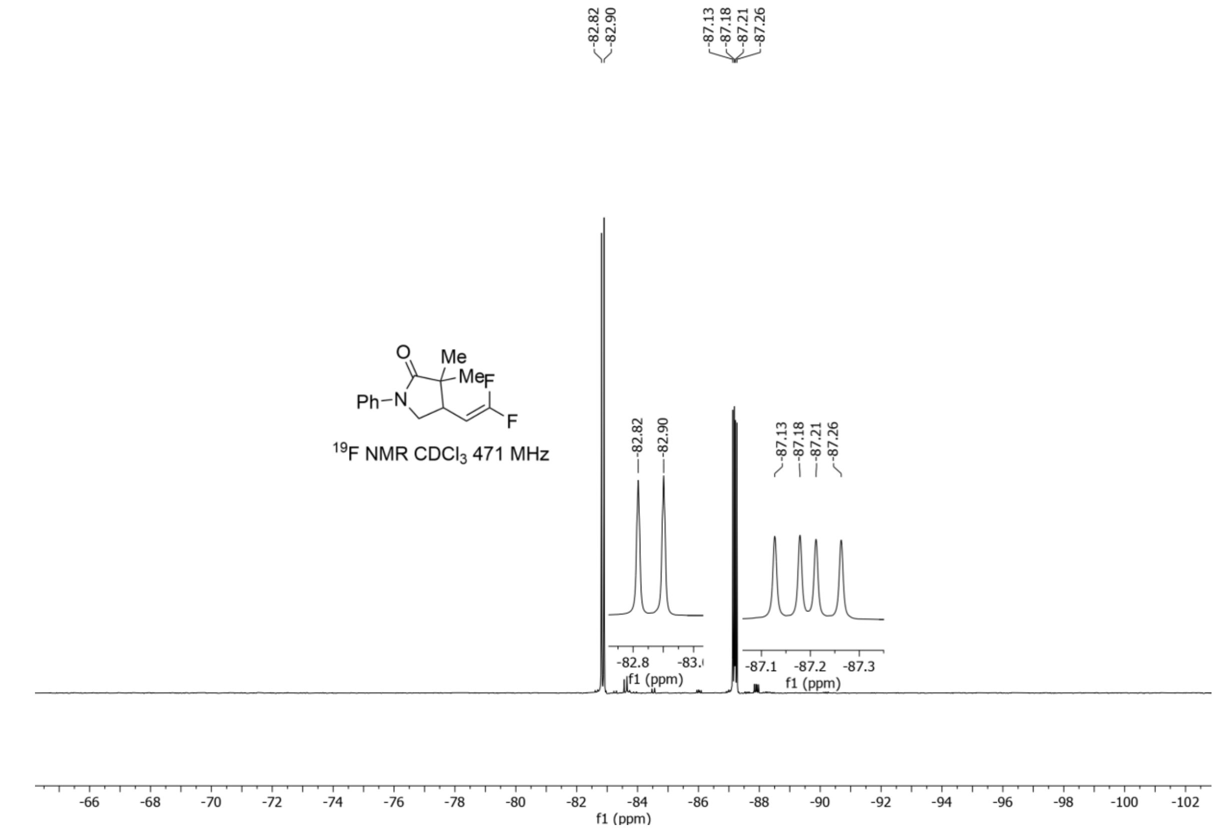

***cis*-3-benzyl-4-(2,2-difluorovinyl)-3-methyl-1-phenylpyrrolidin-2-one (*cis*-3p)**

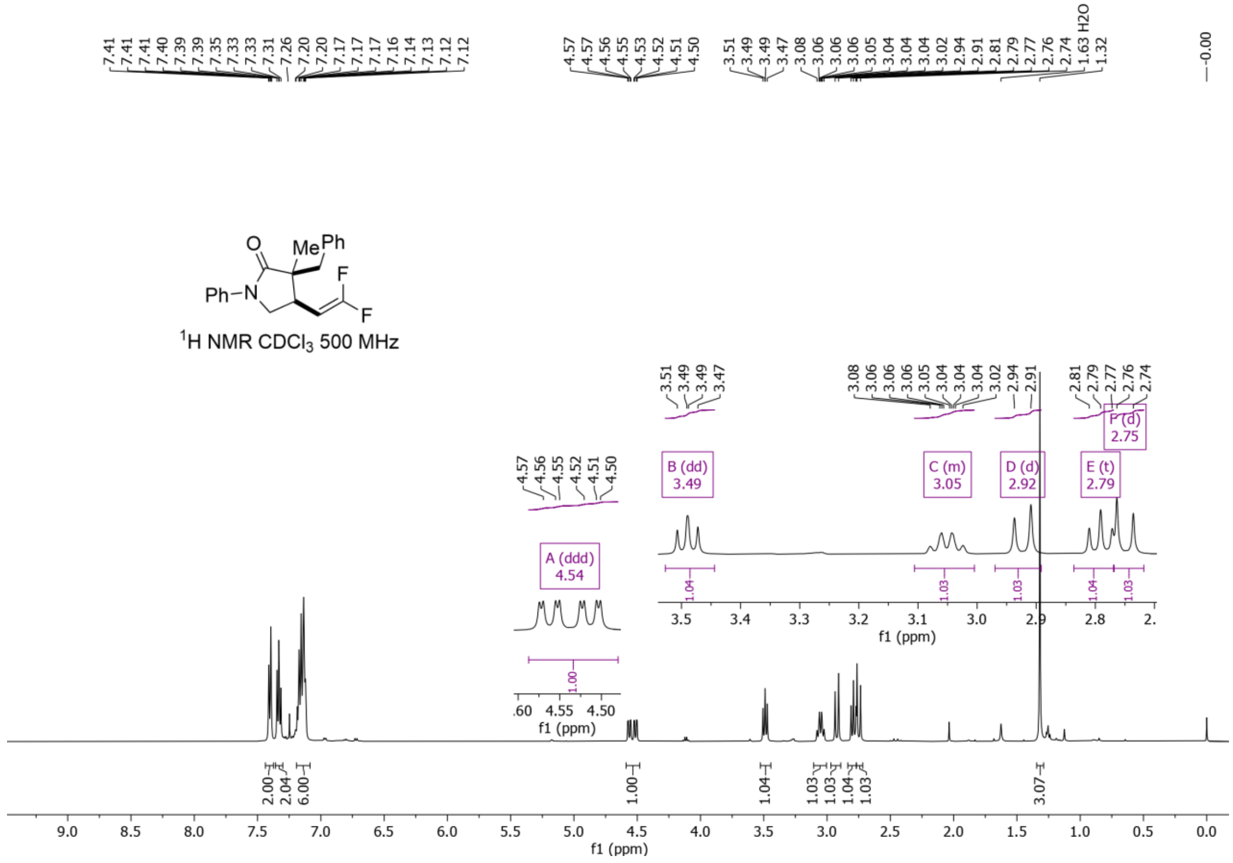

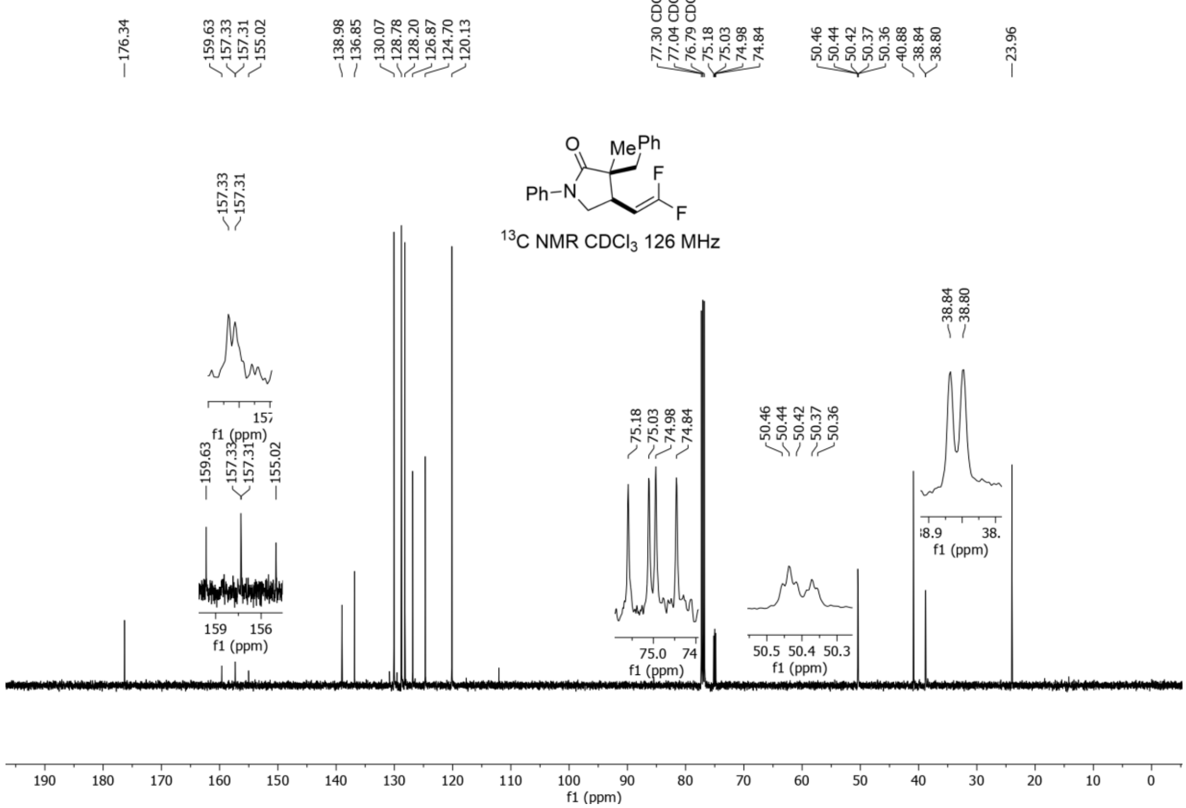

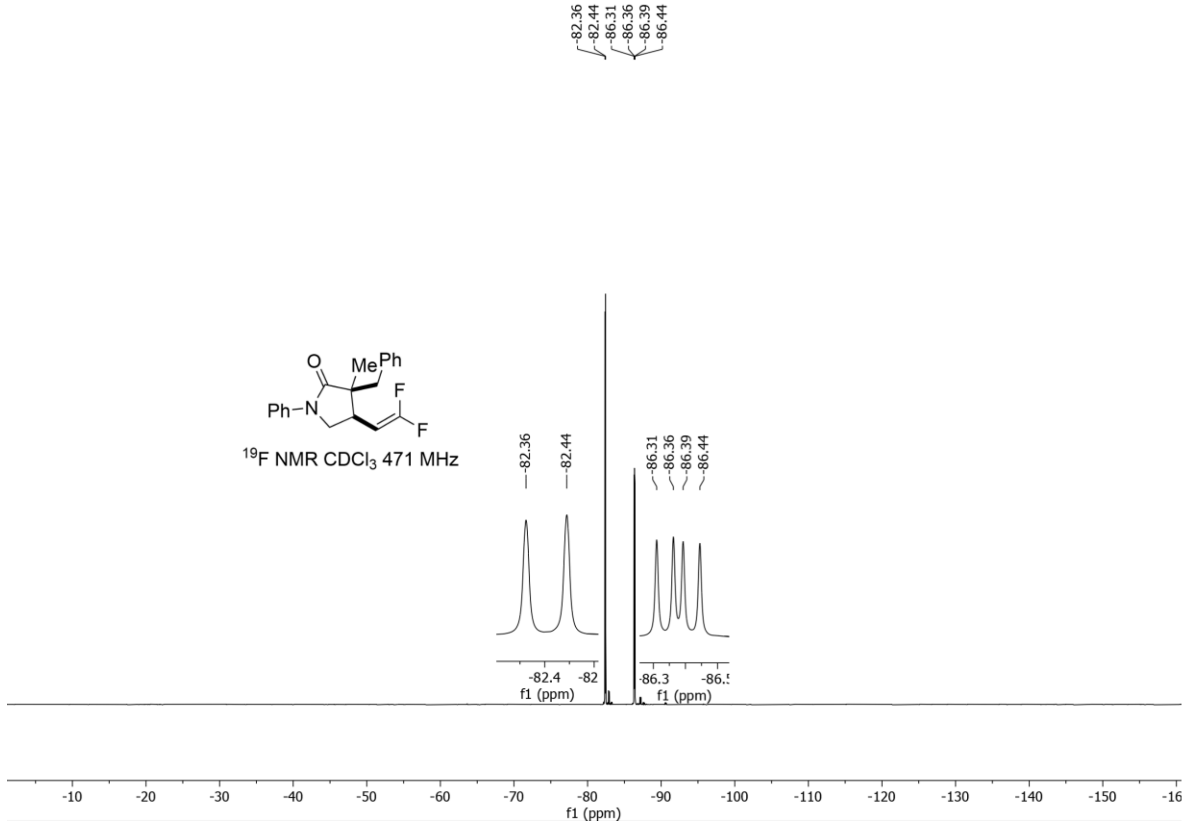

***cis*-3-cyclopentyl-4-(2,2-difluorovinyl)-3-methyl-1-phenylpyrrolidin-2-one (*cis*-3q)**

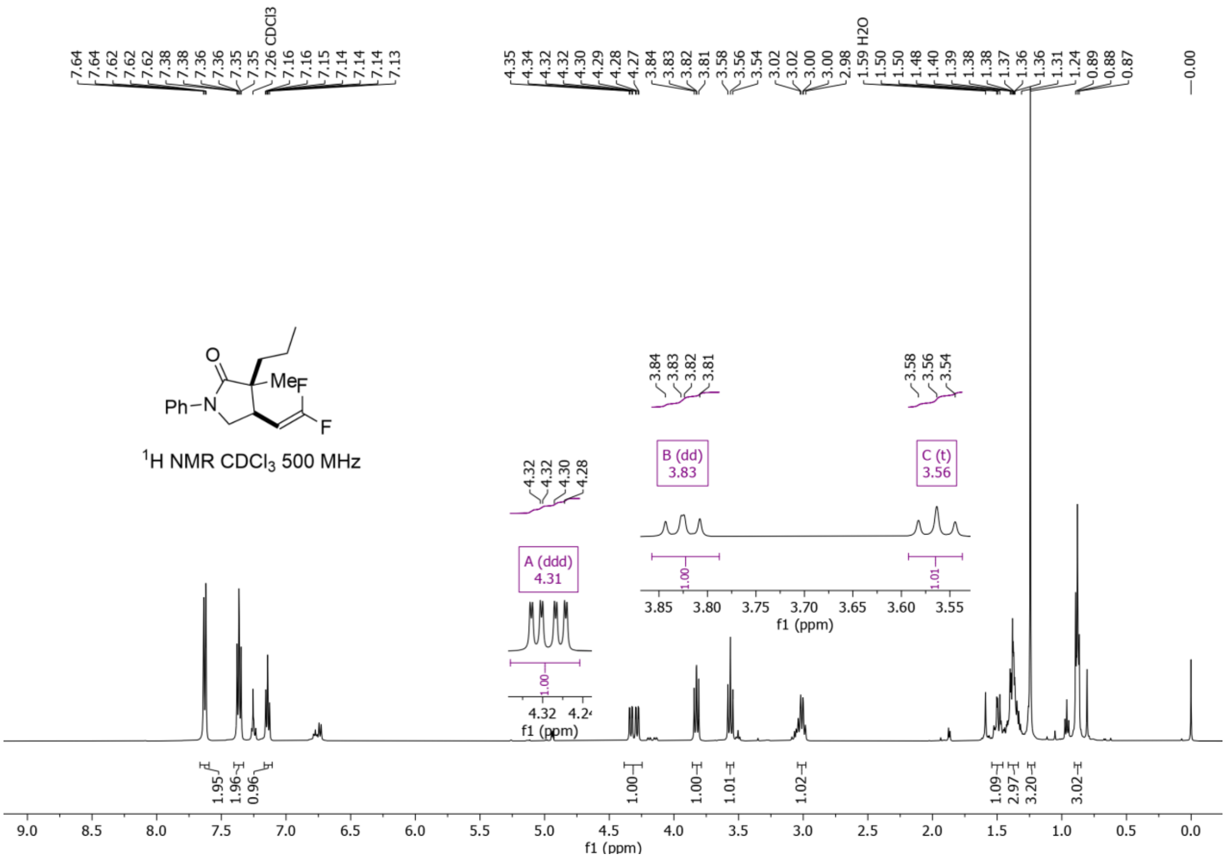

¹H NMR CDCl₃ 500 MHz
7.64 7.64 7.62 7.62 7.38 7.38 7.36 7.36 7.35 7.35 7.26 CDCl3 7.16 7.16 7.15 7.14 7.14 7.14 7.13
4.35 4.34 4.32 4.32 4.30 4.29 4.28 4.27 3.84 3.83 3.82 3.81 3.58 3.56 3.54 3.02 3.02 3.00 3.00 2.98
1.59 H2O 1.50 1.50 1.48 1.40 1.39 1.38 1.38 1.37 1.36 1.36 1.31 1.24 0.89 0.88 0.87
0.00
A (ddd) 4.31
B (dd) 3.83
C (t) 3.56
1.95 1.96 0.96 1.00 1.00 1.01 1.02 1.09 2.97 3.20 3.02
f1 (ppm)

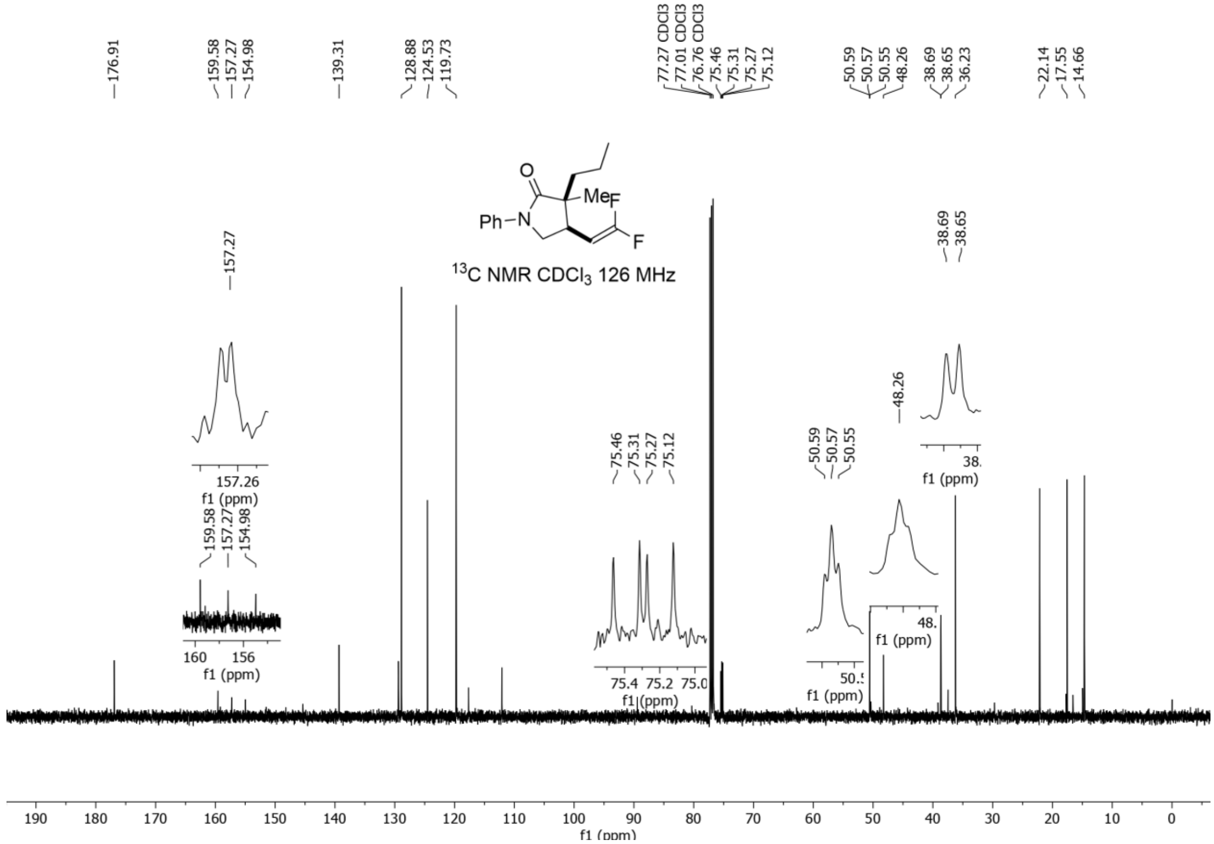

¹³C NMR CDCl₃ 126 MHz
176.91
159.58 157.27 154.98
139.31
128.88 124.53 119.73
77.27 CDCl3 77.01 CDCl3 76.76 CDCl3 75.46 75.31 75.27 75.12
50.59 50.57 50.55 48.26
38.69 38.65 36.23
22.14 17.55 14.66
f1 (ppm)

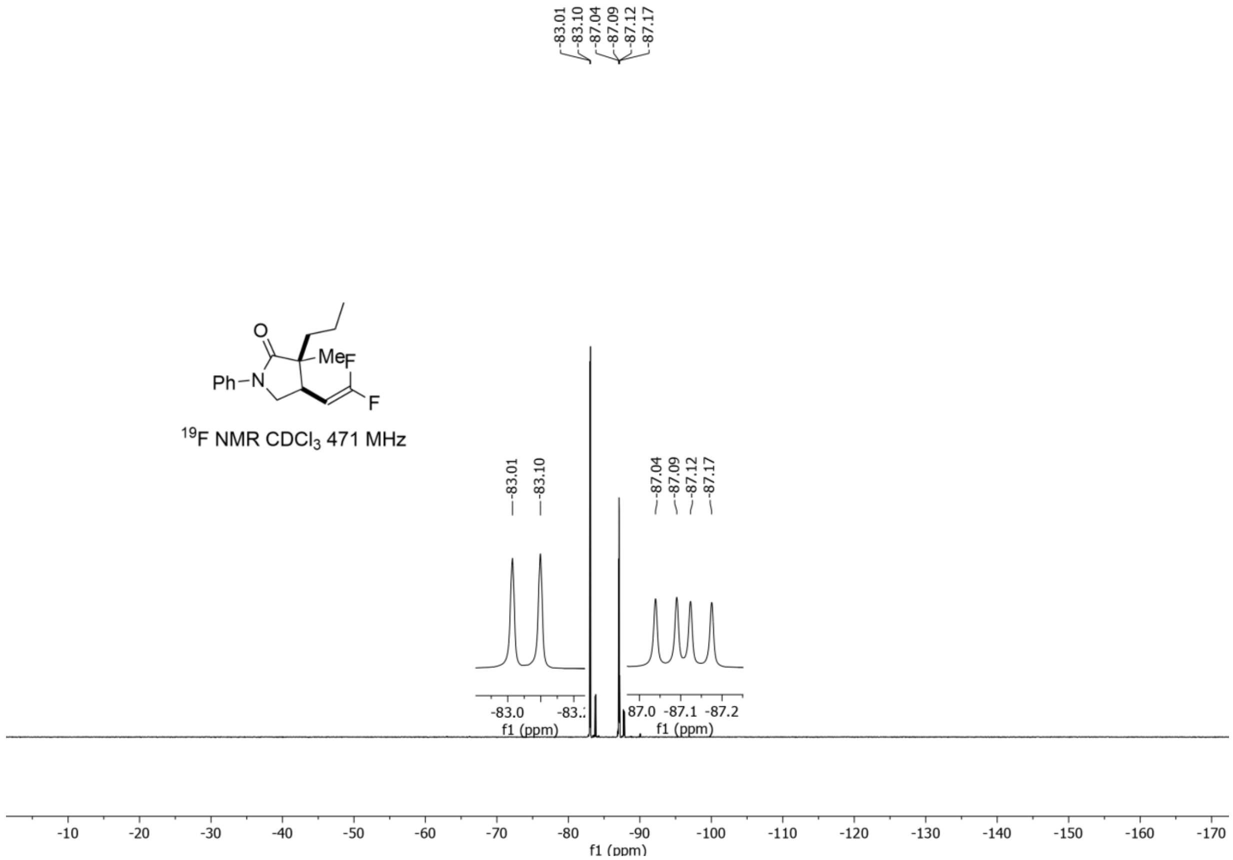

cis-4-(2,2-difluorovinyl)-3-isobutyl-3-methyl-1-phenylpyrrolidin-2-one (_cis_-3r)

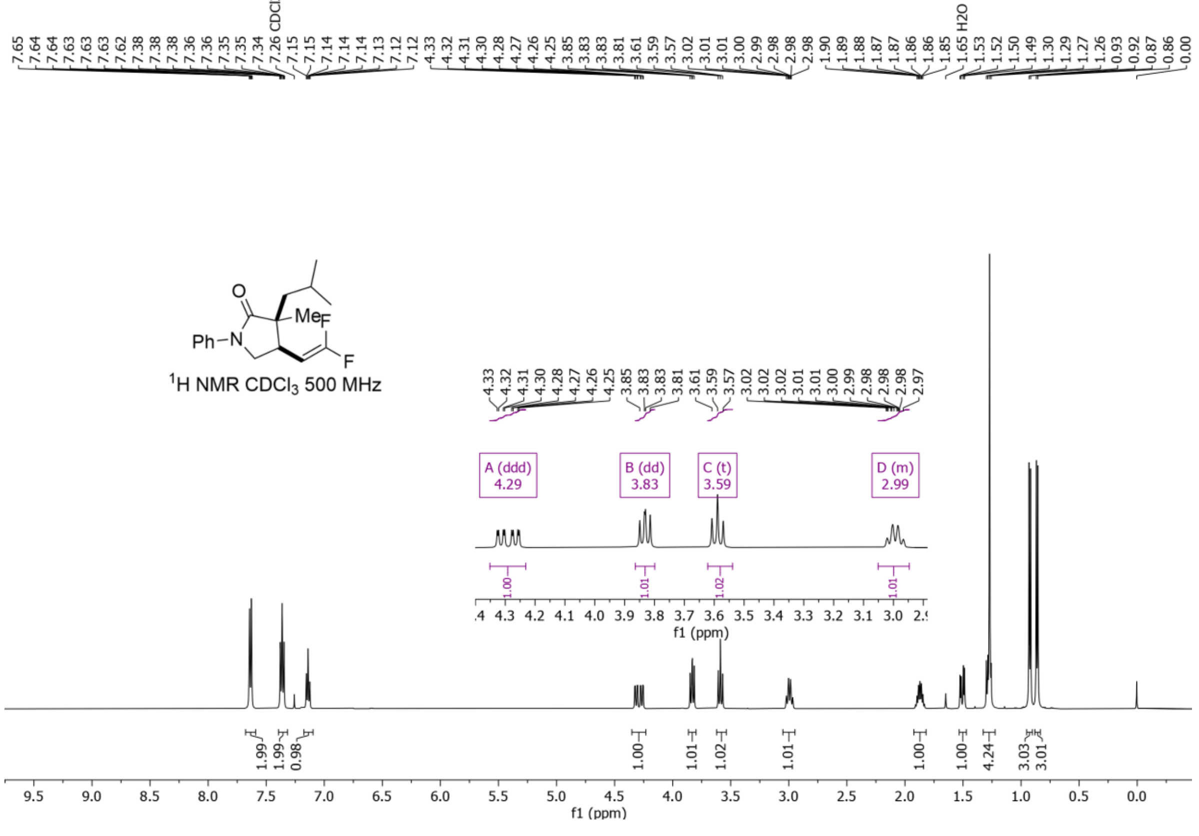

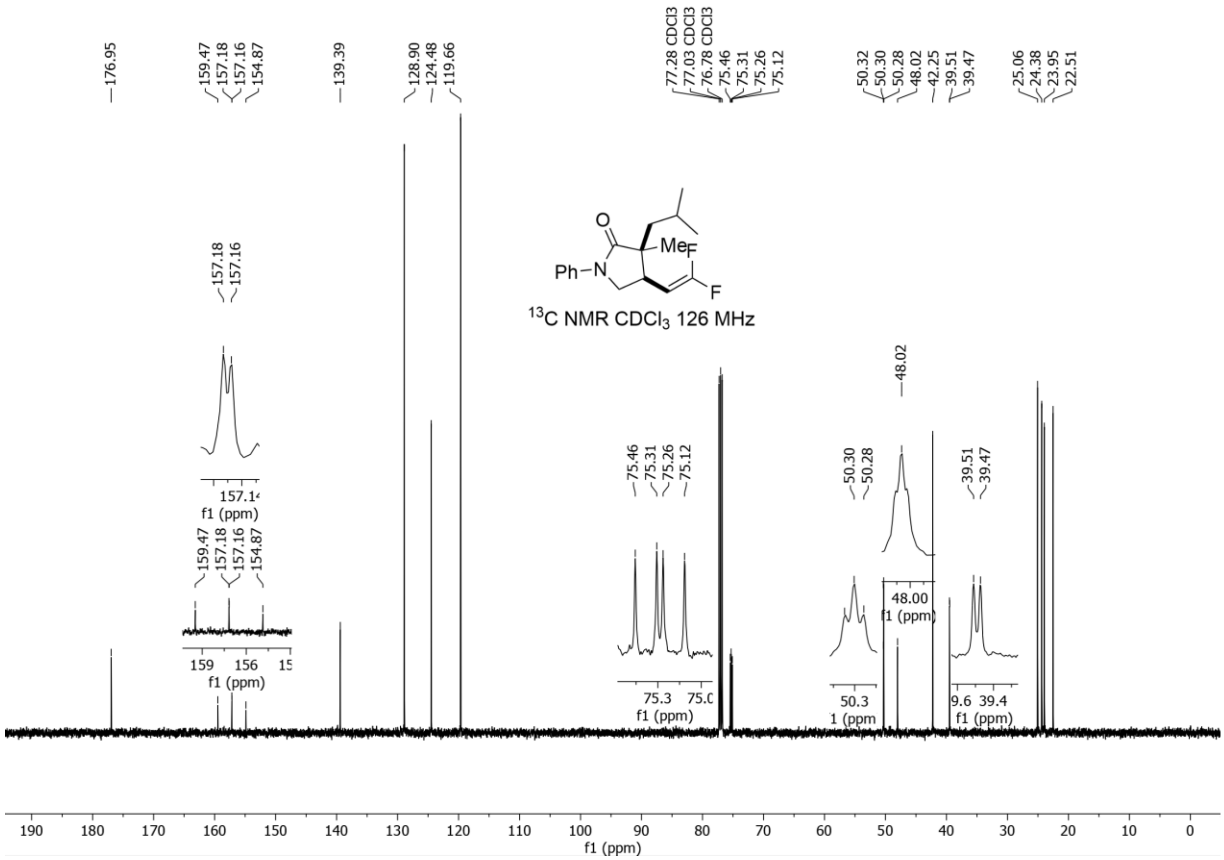
176.95
159.47
157.18
157.16
154.87
139.39
128.90
124.48
119.66
77.28 CDCl3
77.03 CDCl3
76.78 CDCl3
75.46
75.31
75.26
75.12
50.32
50.30
50.28
48.02
42.25
39.51
39.47
25.06
24.38
23.95
22.51
O
Me
F
F
Ph
N
13C NMR CDCl3 126 MHz
f1 (ppm)

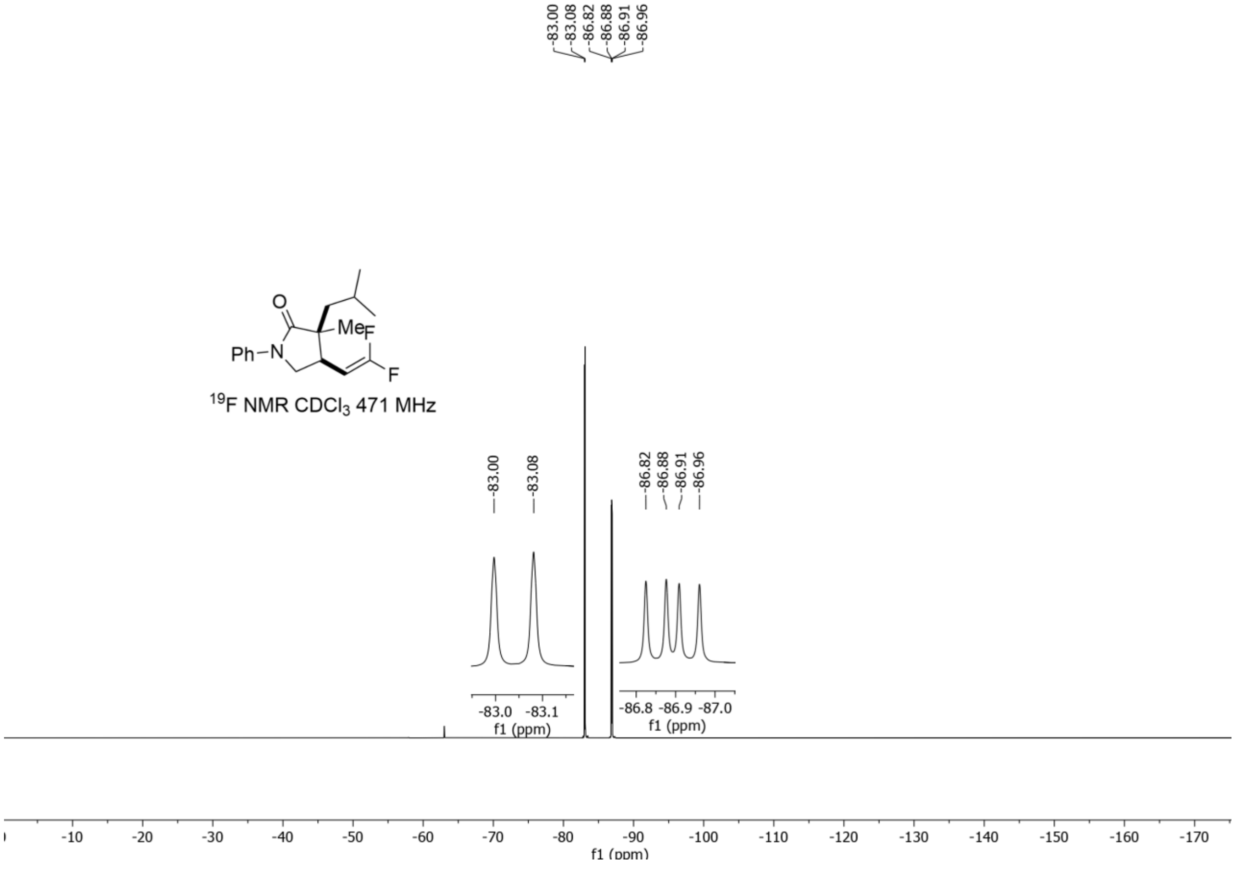
-83.00
-83.08
-86.82
-86.88
-86.91
-86.96
O
Me
F
F
Ph
N
19F NMR CDCl3 471 MHz
f1 (ppm)

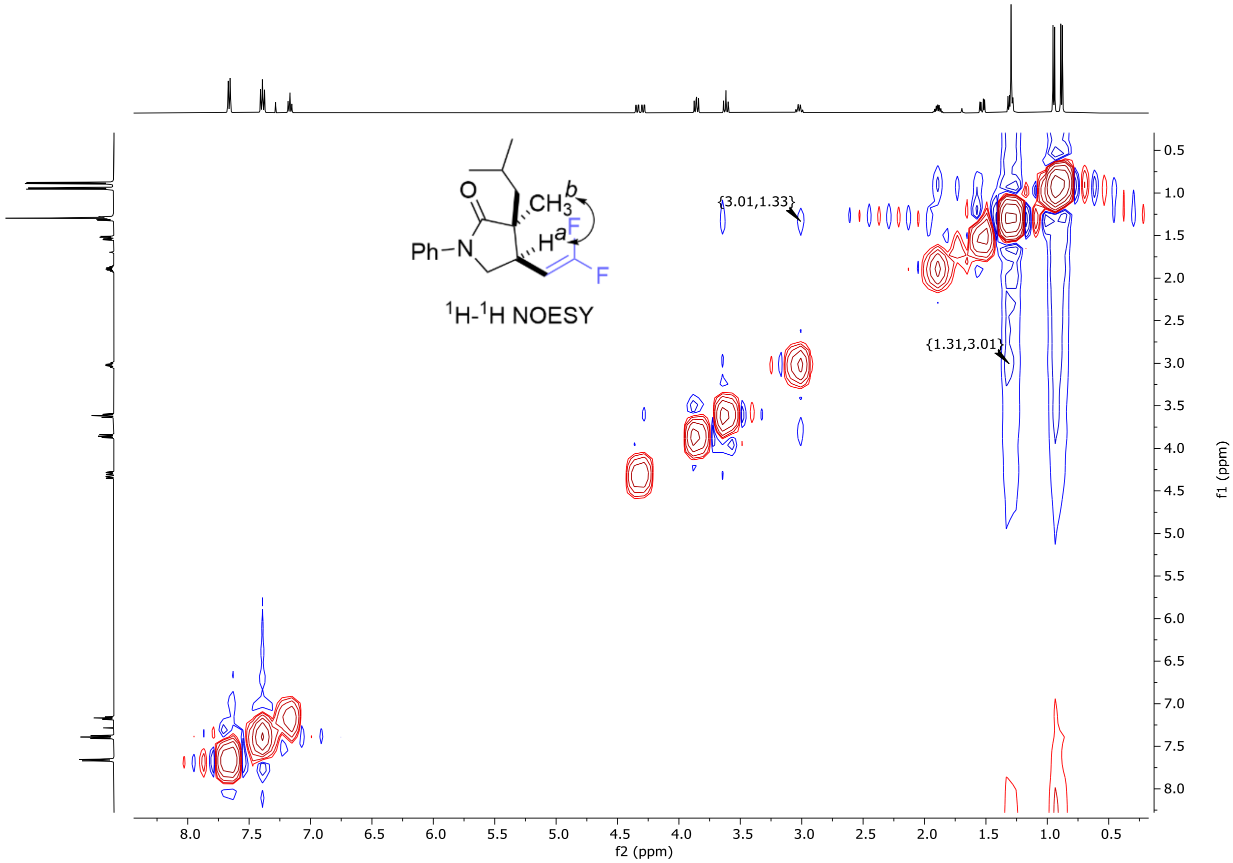

***trans*-4-(2,2-difluorovinyl)-3-isobutyl-3-methyl-1-phenylpyrrolidin-2-one (*trans*-3r)**

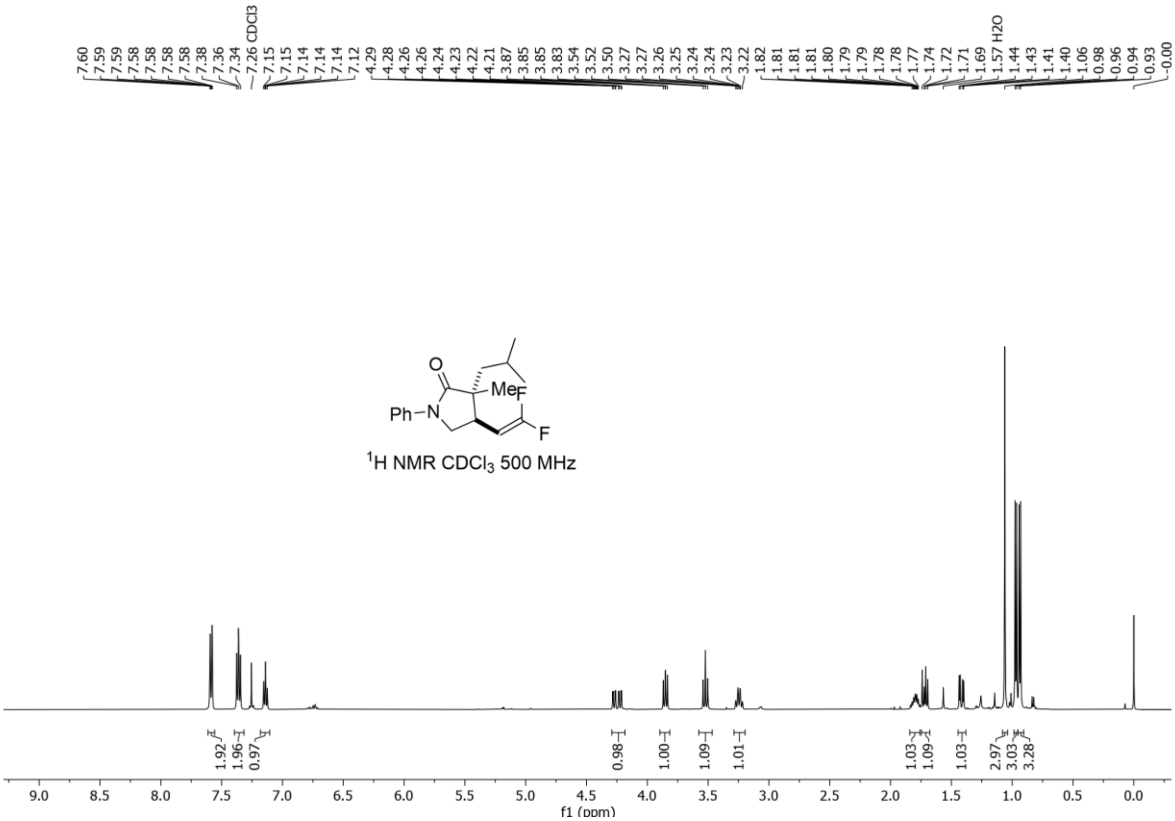

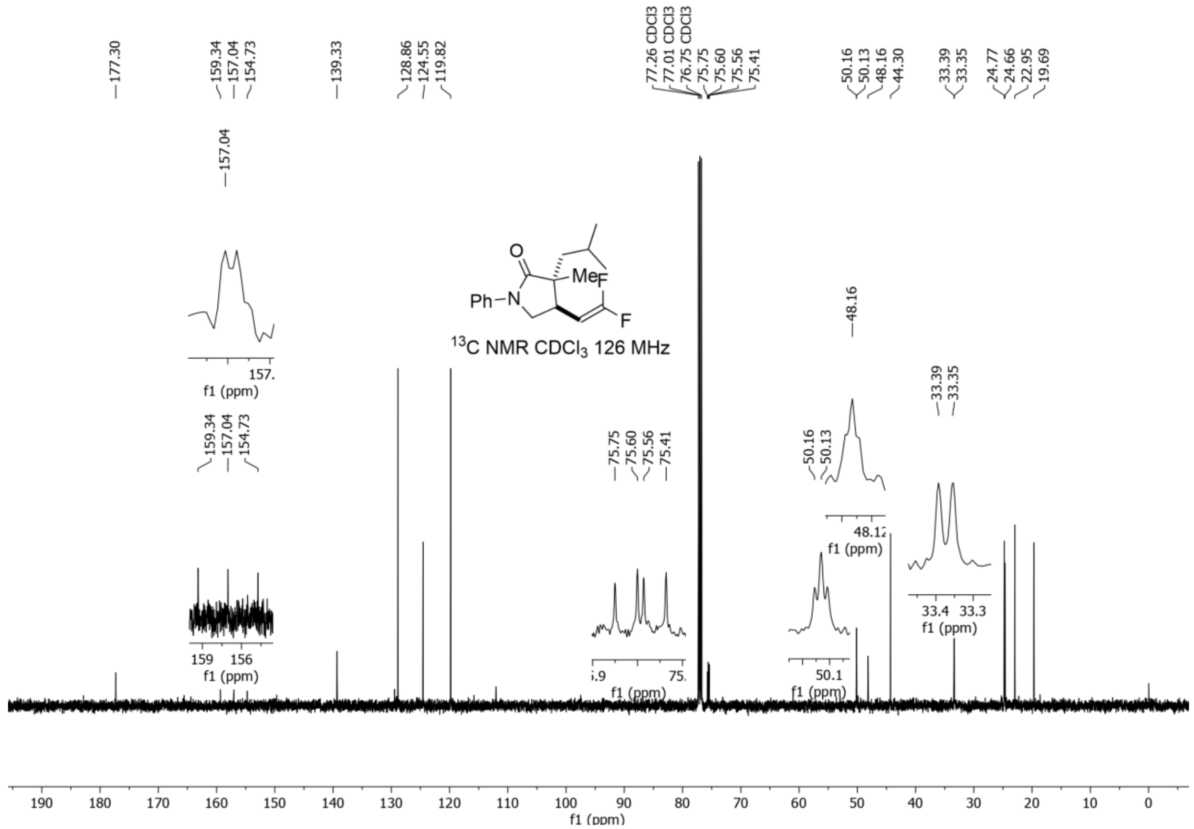
177.30
159.34
157.04
154.73
139.33
128.86
124.55
119.82
77.26 CDCl3
77.01 CDCl3
76.75 CDCl3
75.75
75.60
75.56
75.41
50.16
50.13
48.16
44.30
33.39
33.35
24.77
24.66
22.95
19.69
O
Me
F
F
Ph
N
13C NMR CDCl3 126 MHz
f1 (ppm)

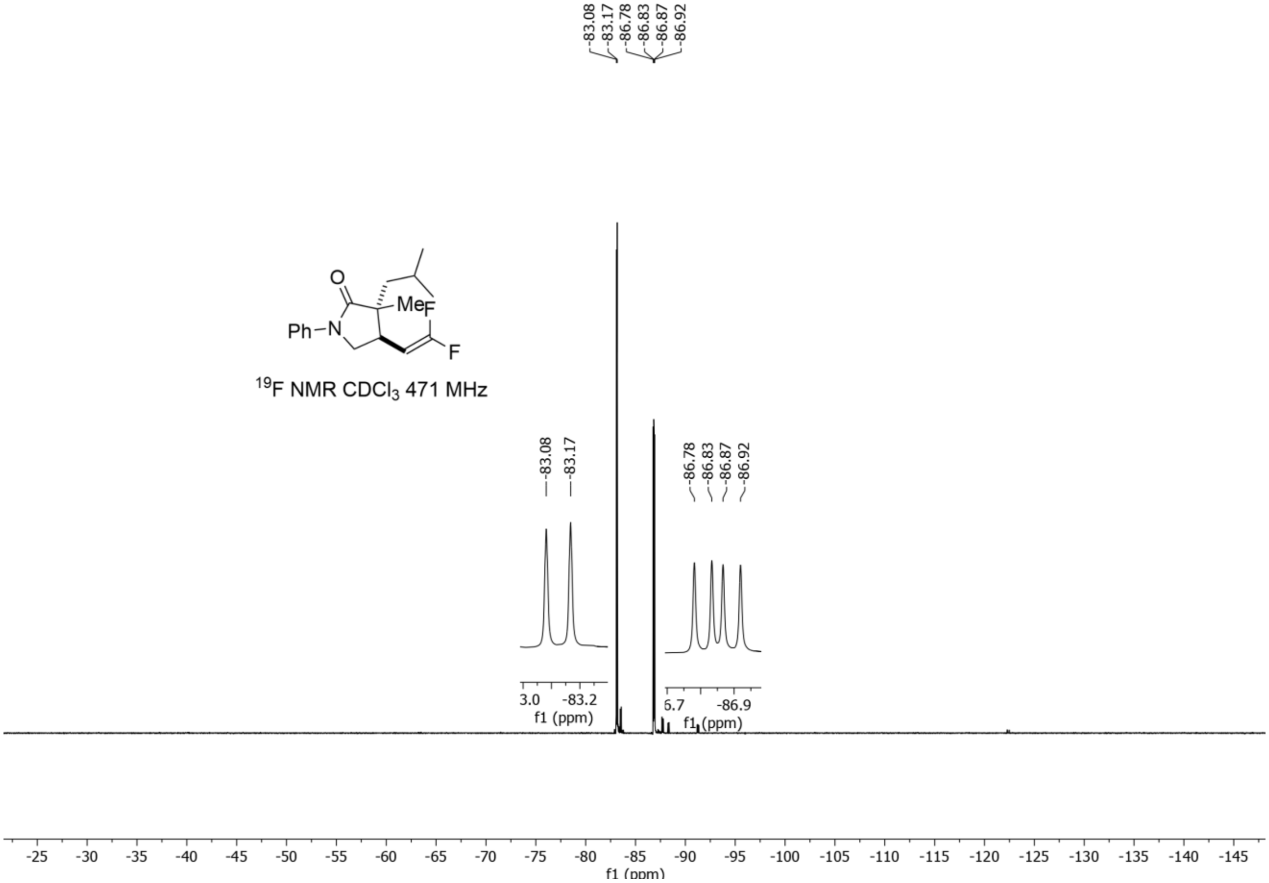
-83.08
-83.17
-86.78
-86.83
-86.87
-86.92
O
Me
F
F
Ph
N
19F NMR CDCl3 471 MHz
f1 (ppm)

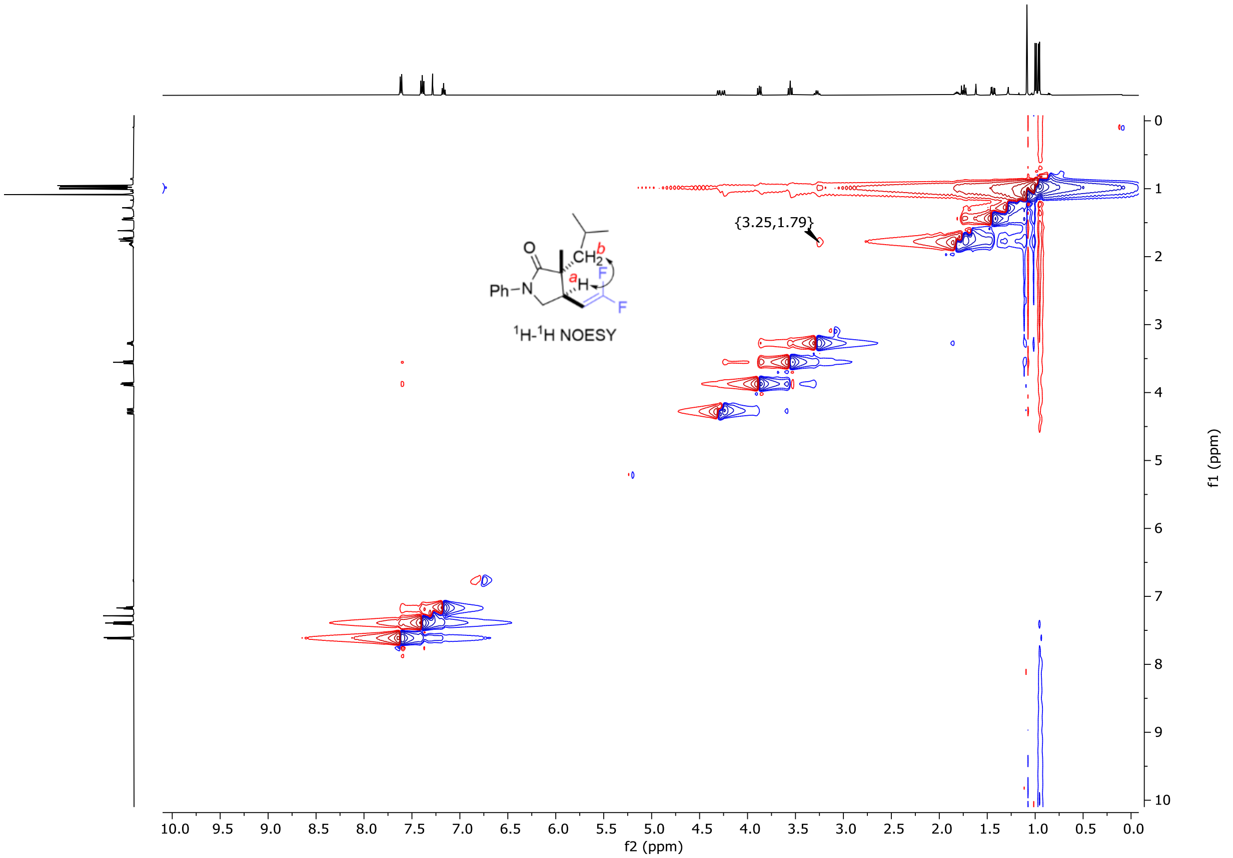

cis-3-cyclopropyl-4-(2,2-difluorovinyl)-3-methyl-1-phenylpyrrolidin-2-one (_cis_-3s)

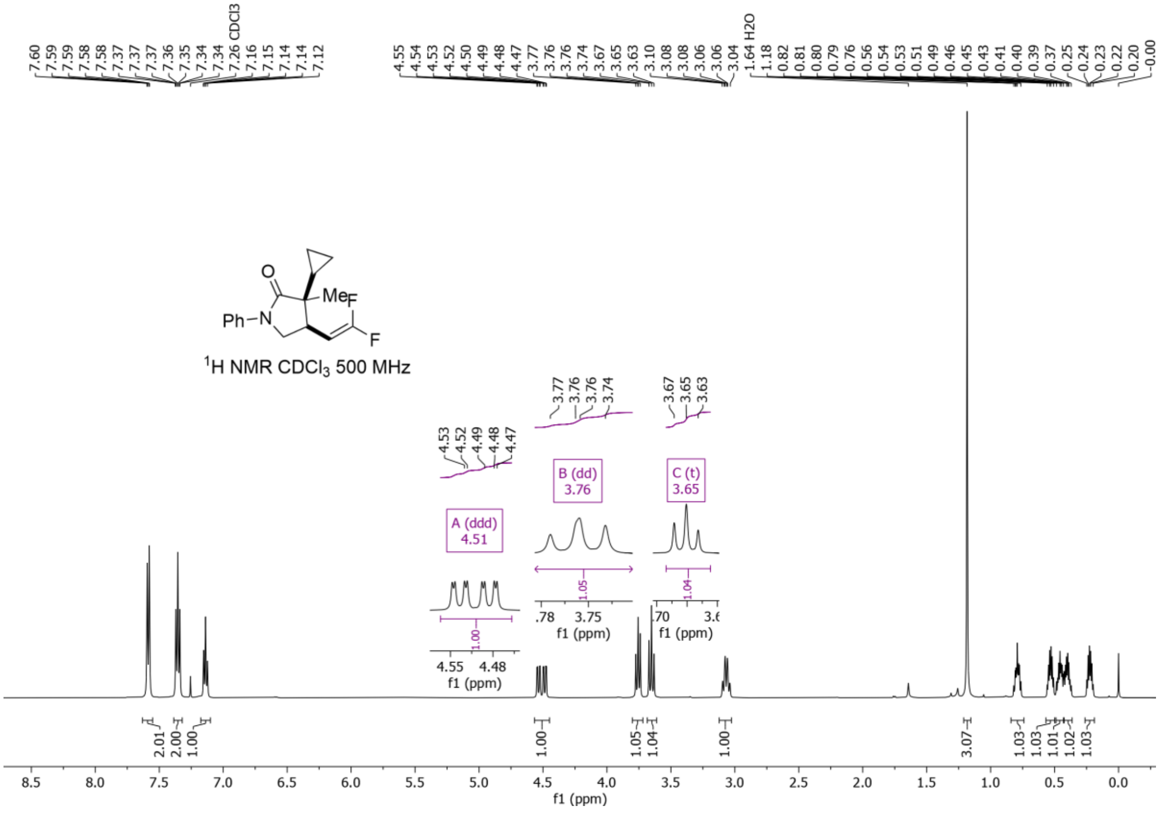

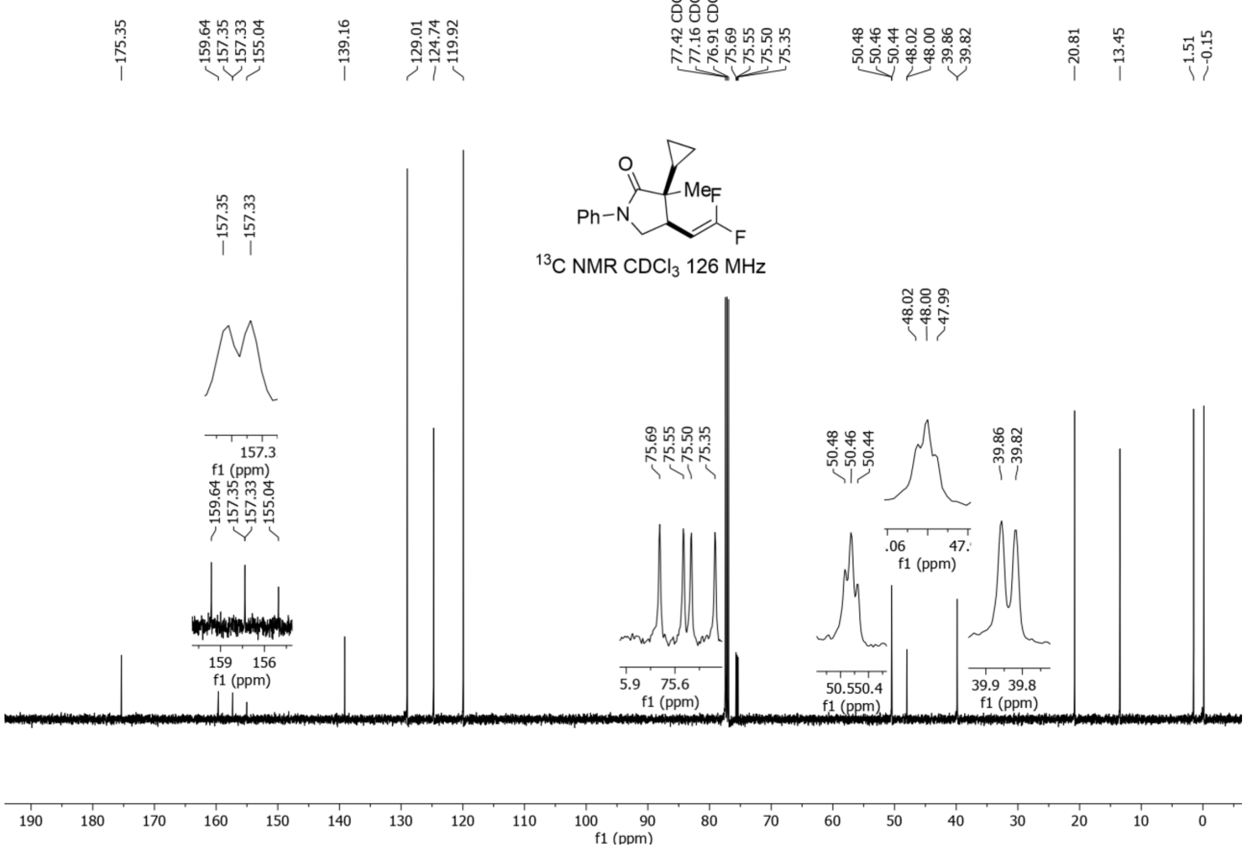
175.35
159.64
157.35
157.33
155.04
139.16
129.01
124.74
119.92
77.42 CDCl3
77.16 CDCl3
76.91 CDCl3
75.69
75.55
75.50
75.35
50.48
50.46
50.44
48.02
48.00
39.86
39.82
20.81
13.45
1.51
-0.15
O
Me
F
Ph
N
F
^{13}C NMR CDCl$_3$ 126 MHz
48.02
48.00
47.99
f1 (ppm)
f1 (ppm)

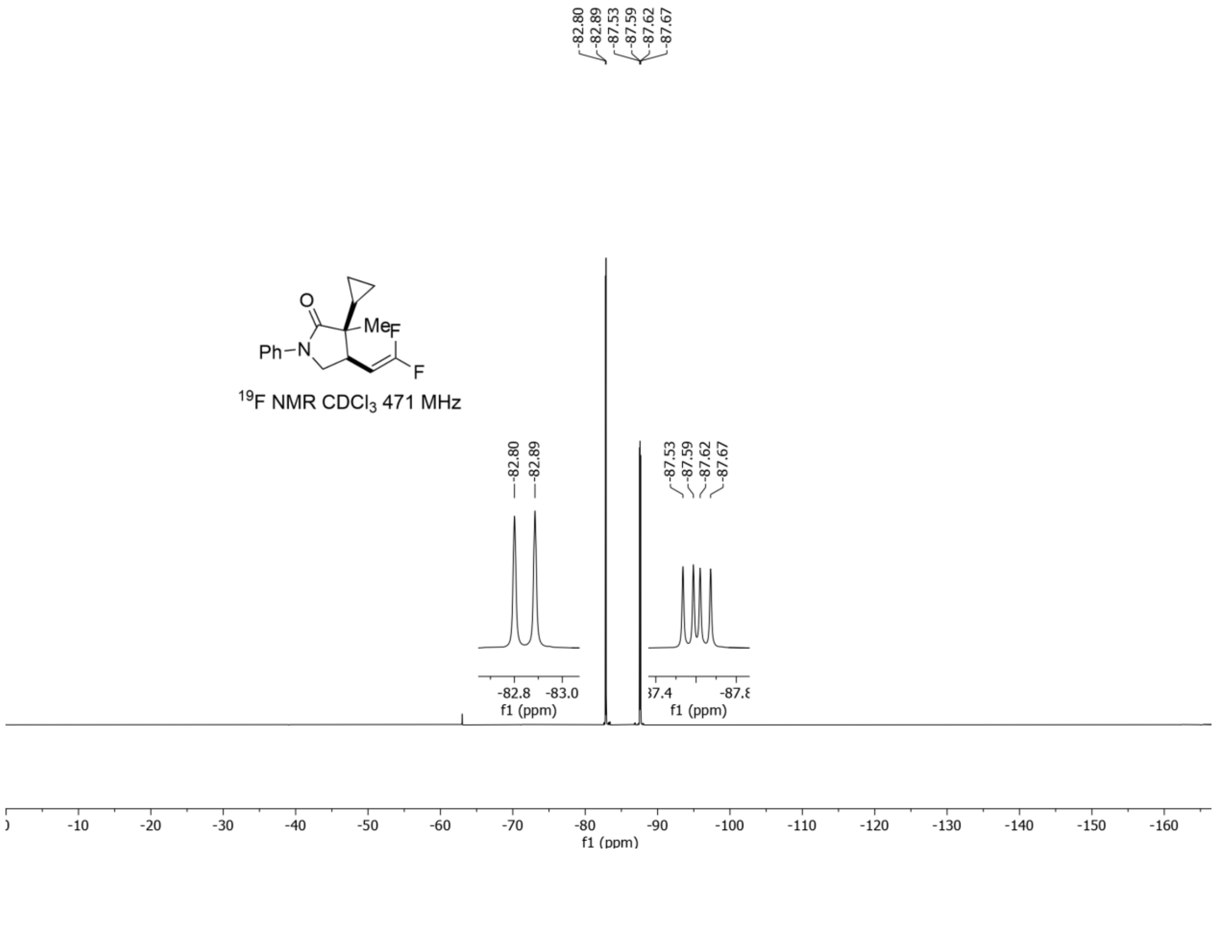
-82.80
-82.89
-87.53
-87.59
-87.62
-87.67
O
Me
F
Ph
N
F
^{19}F NMR CDCl$_3$ 471 MHz
f1 (ppm)

***trans*-3-cyclopentyl-4-(2,2-difluorovinyl)-3-methyl-1-phenylpyrrolidin-2-one (*trans*-3t)**

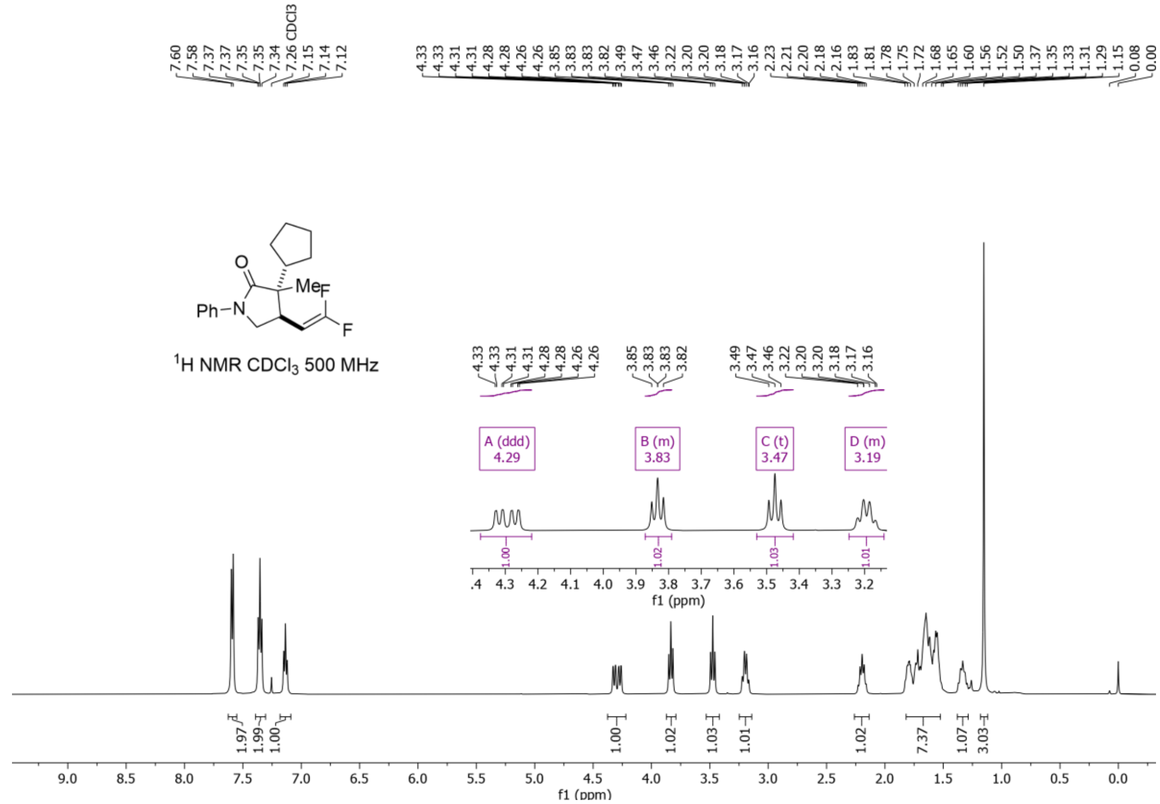

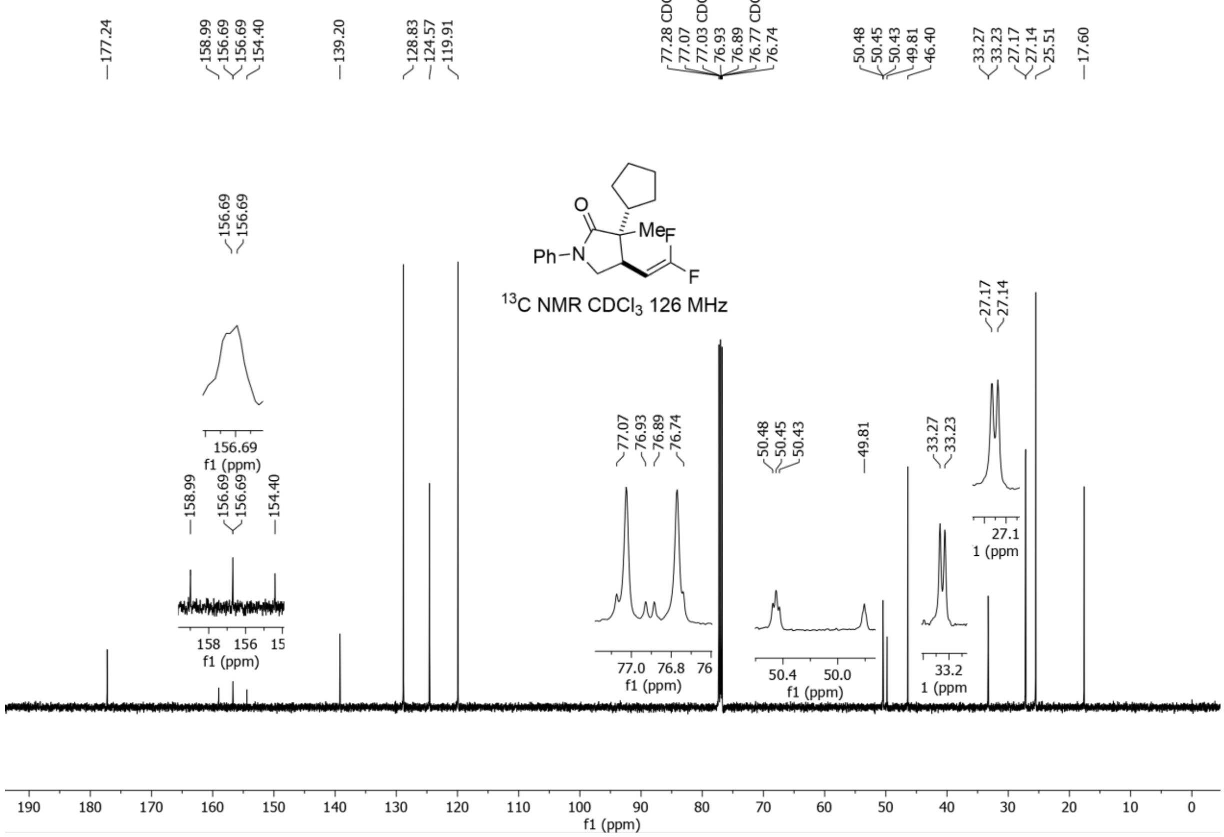

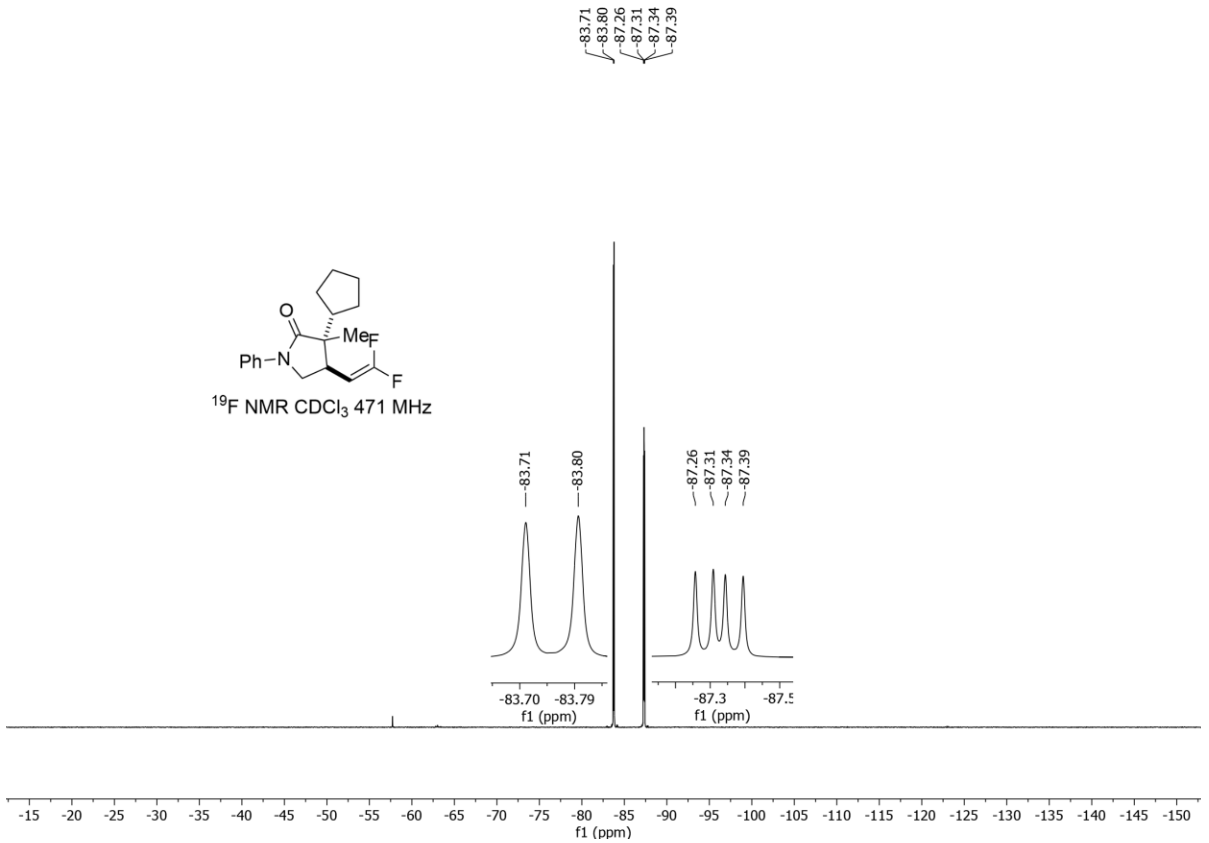

***cis*-3-cyclopentyl-4-(2,2-difluorovinyl)-3-methyl-1-phenylpyrrolidin-2-one (*cis*-3t)**

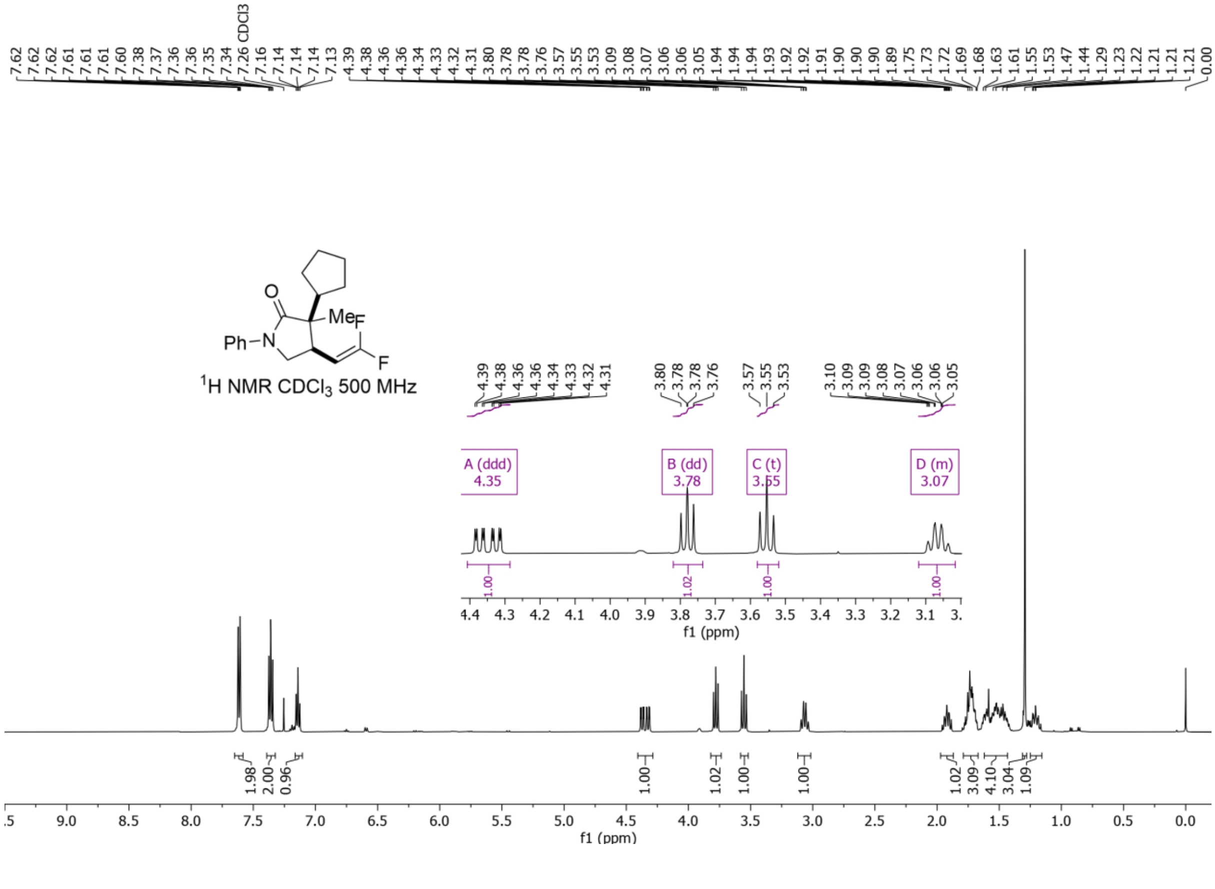

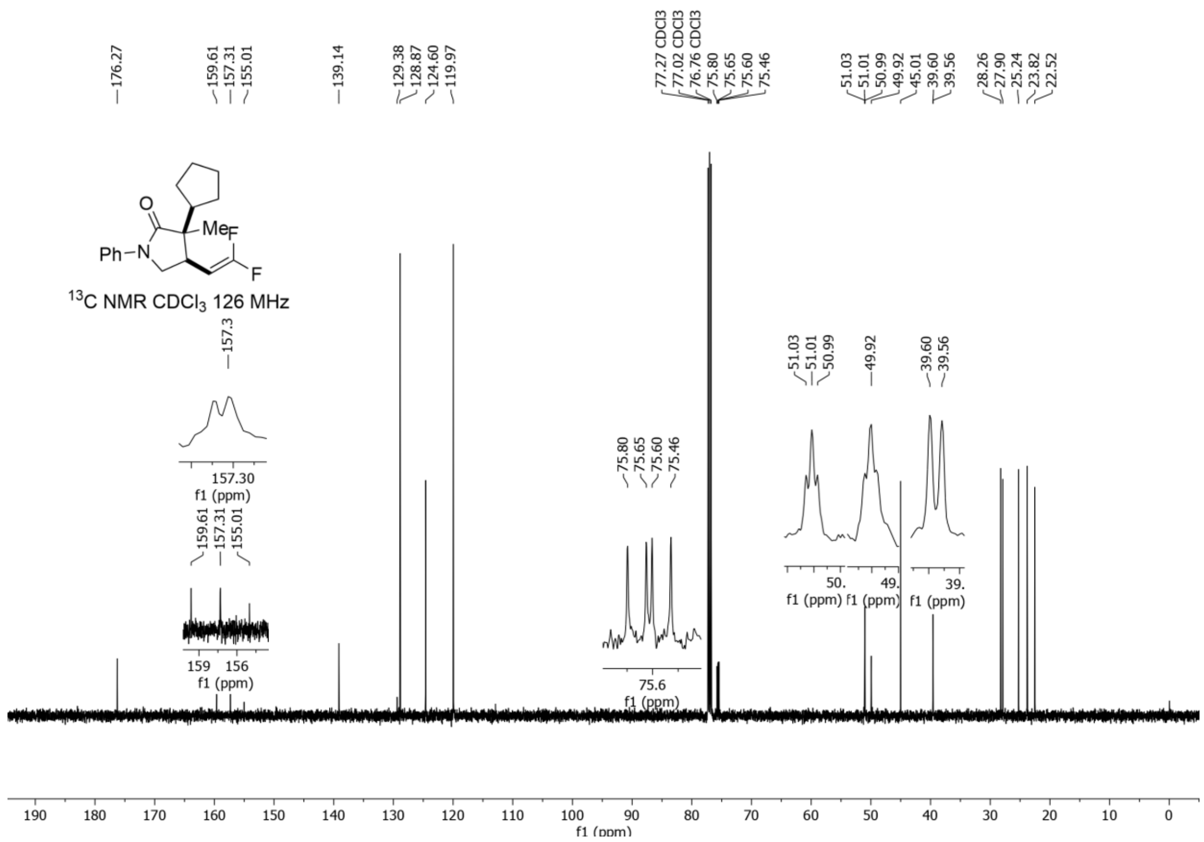

Ph
N
O
Me
F
F
13C NMR CDCl3 126 MHz
176.27
159.61
157.31
155.01
139.14
129.38
128.87
124.60
119.97
77.27 CDCl3
77.02 CDCl3
76.76 CDCl3
75.80
75.65
75.60
75.46
51.03
51.01
50.99
49.92
45.01
39.60
39.56
28.26
27.90
25.24
23.82
22.52
f1 (ppm)

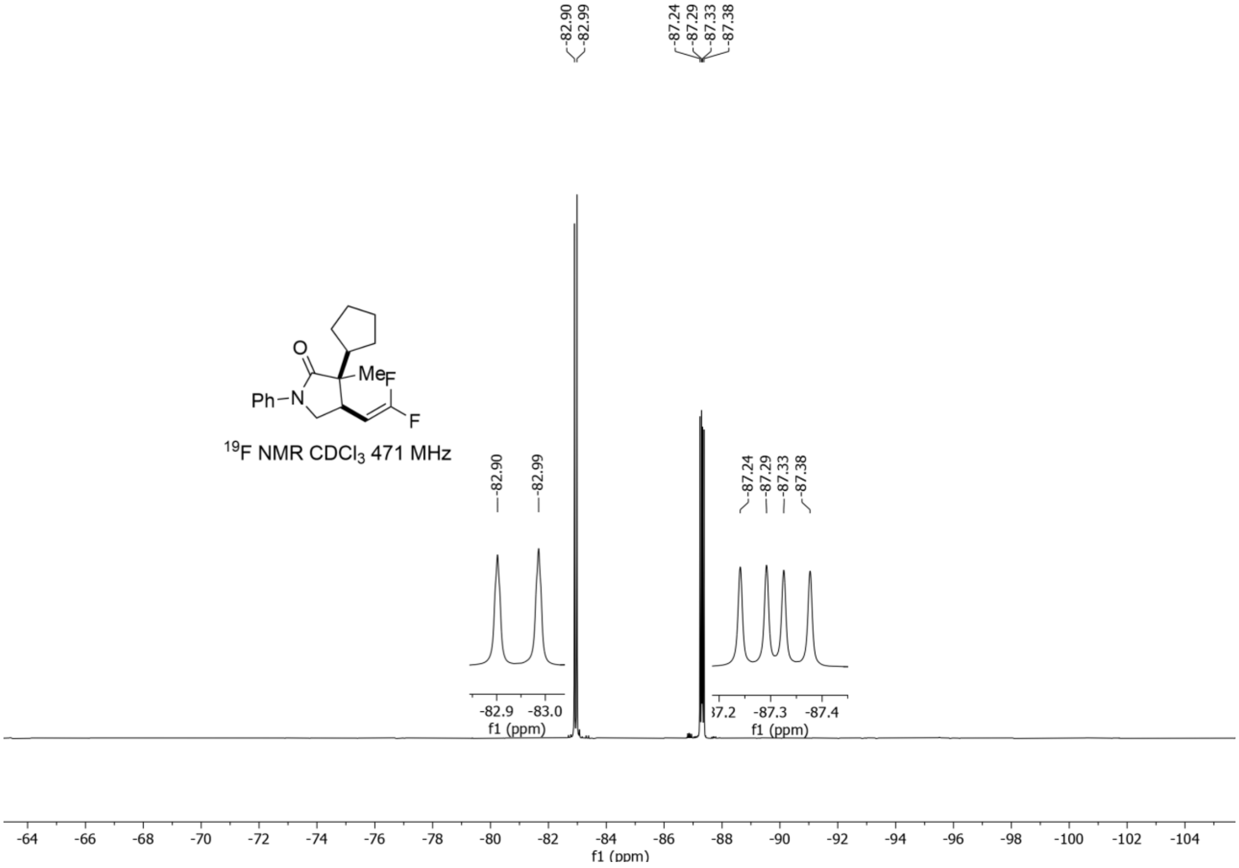

Ph
N
O
Me
F
F
19F NMR CDCl3 471 MHz
-82.90
-82.99
-87.24
-87.29
-87.33
-87.38
f1 (ppm)

cis-1-(4-(_tert_-butyl)phenyl)-4-(2,2-difluorovinyl)-3-methyl-3-phenylpyrrolidin-2-one (_cis_-3u)

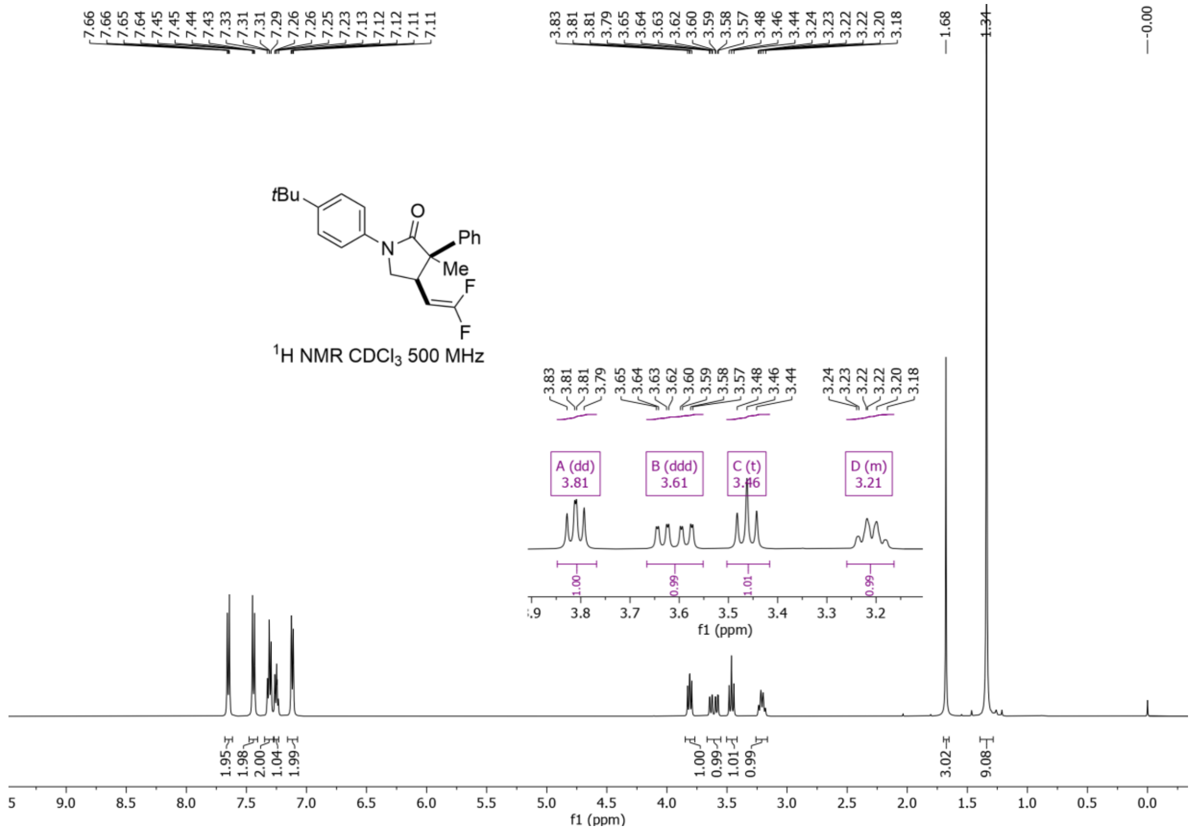

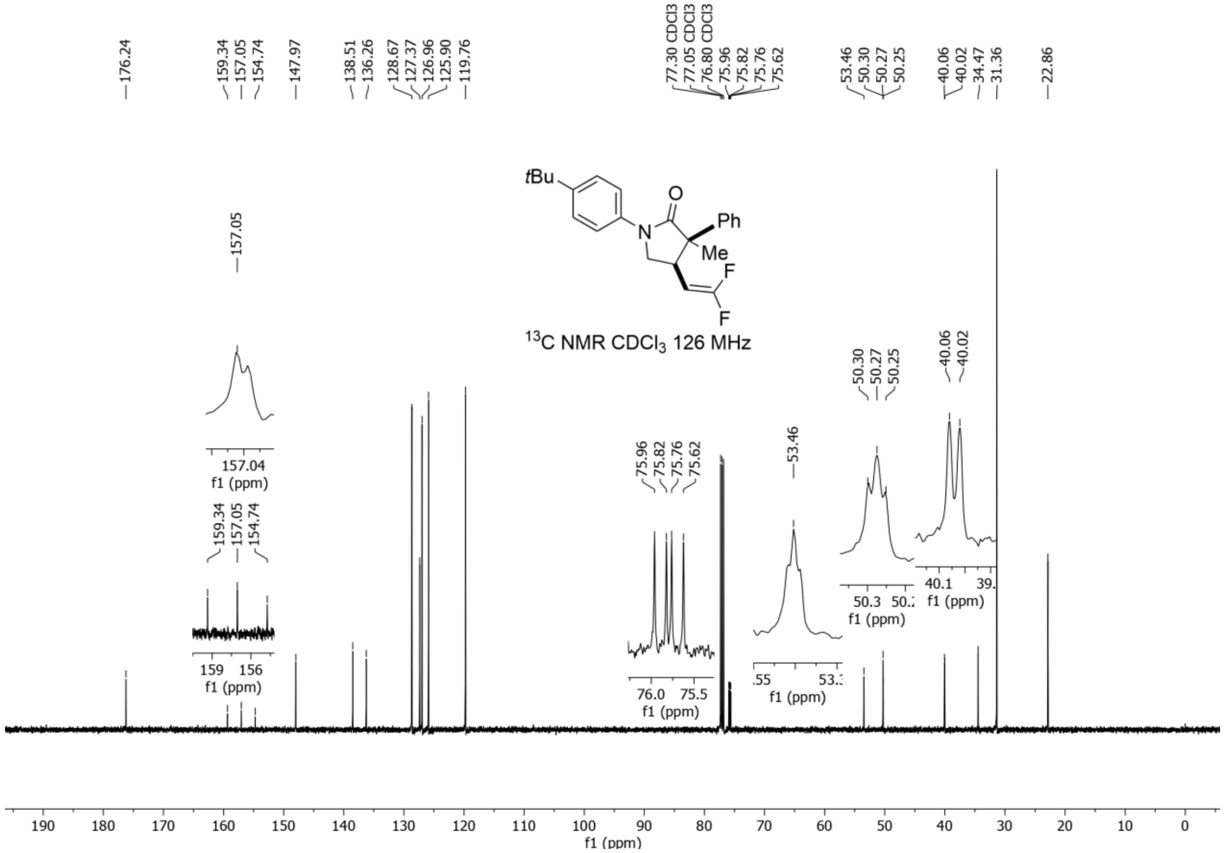

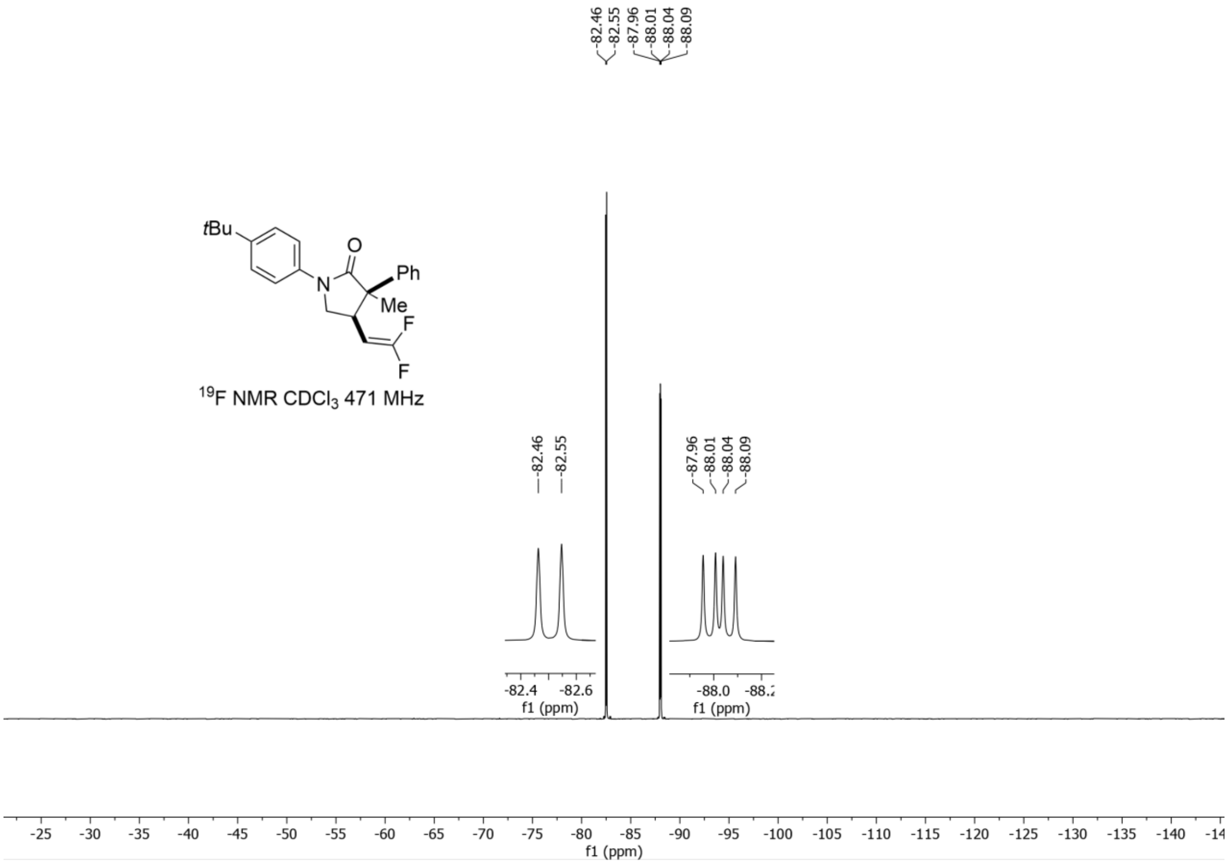

***cis*-1-benzyl-4-(2,2-difluorovinyl)-3-methyl-3-phenylpyrrolidin-2-one (*cis*-3v)**

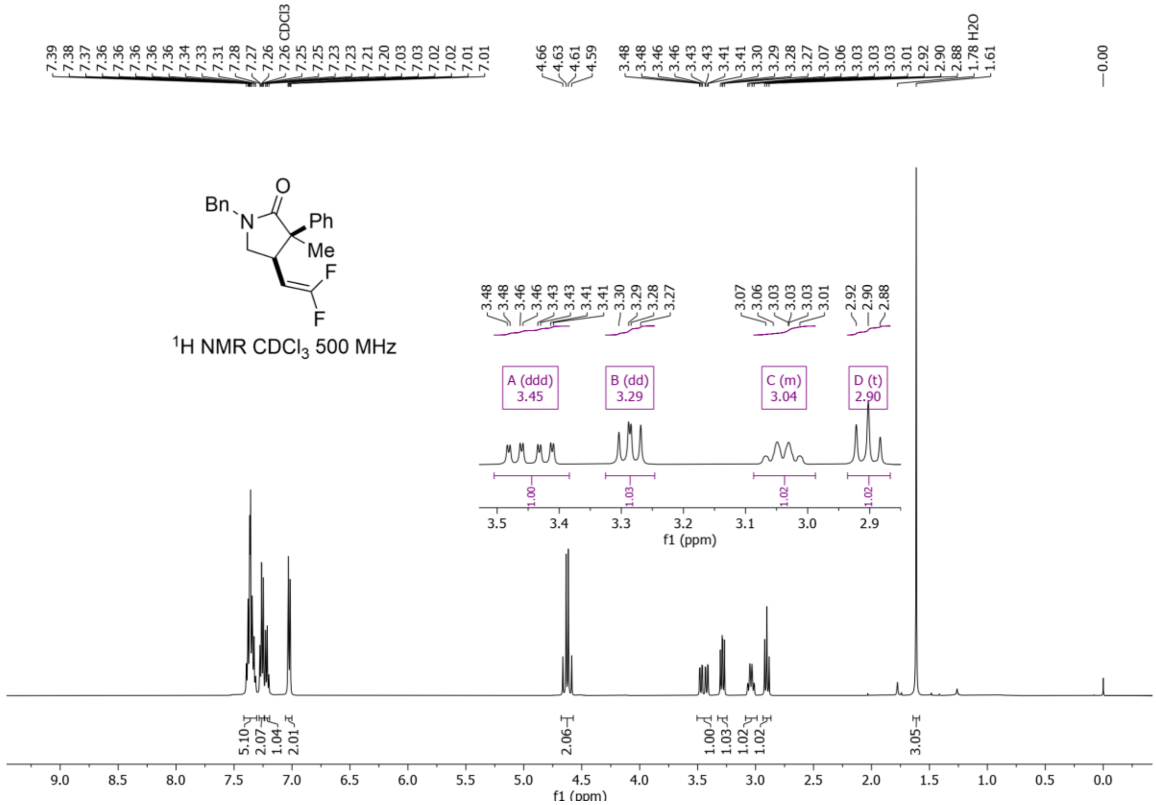

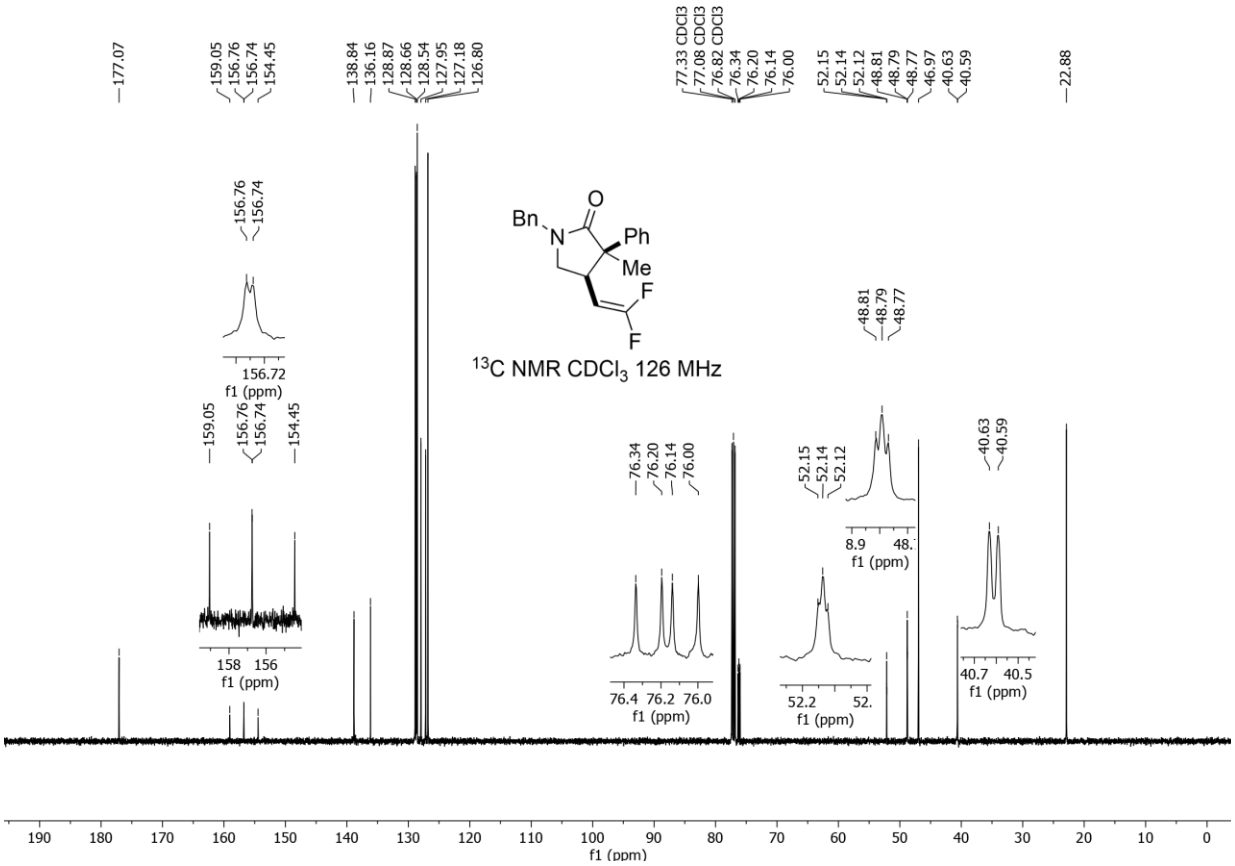
177.07
159.05
156.76
156.74
154.45
138.84
136.16
128.87
128.66
128.54
127.95
127.18
126.80
77.33 CDCl3
77.08 CDCl3
76.82 CDCl3
76.34
76.20
76.14
76.00
52.15
52.14
52.12
48.81
48.79
48.77
46.97
40.63
40.59
22.88
Bn
N
O
Ph
Me
F
F
13C NMR CDCl3 126 MHz
f1 (ppm)

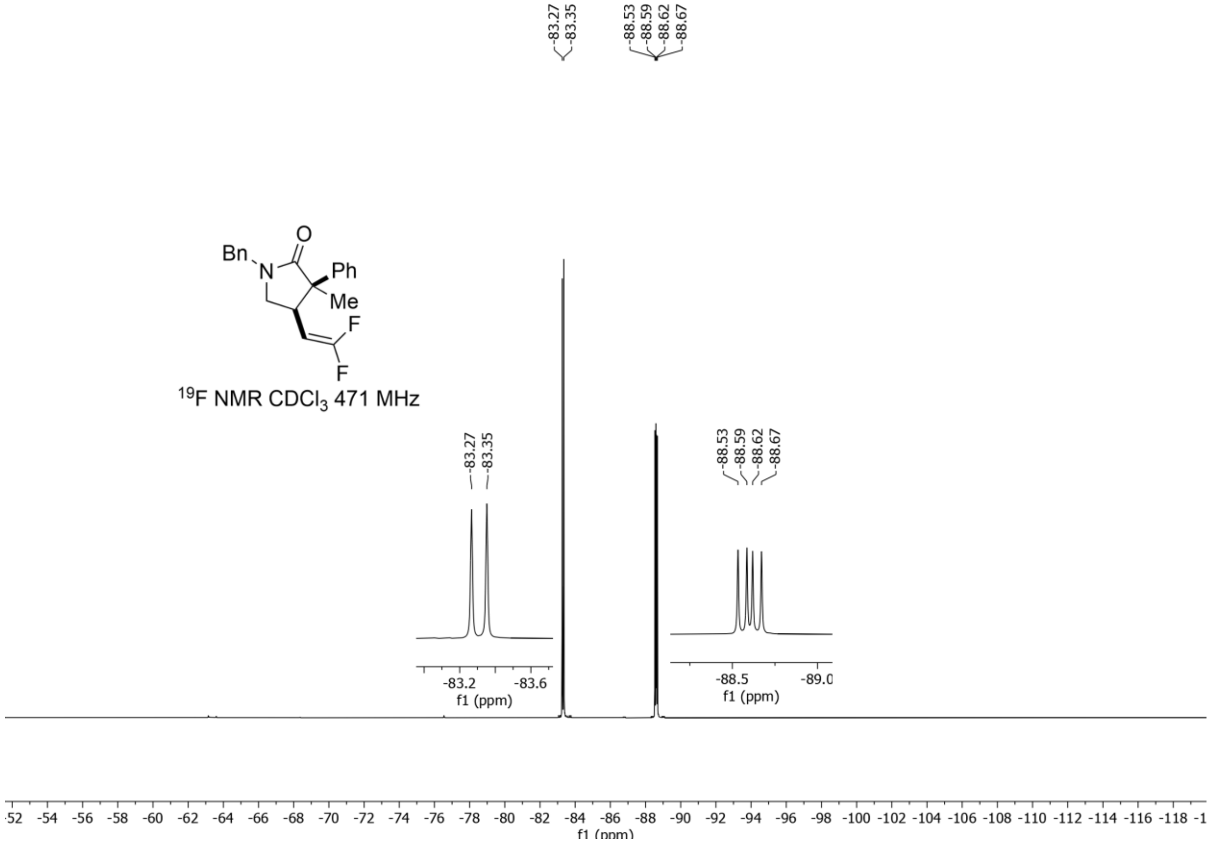
-83.27
-83.35
-88.53
-88.59
-88.62
-88.67
Bn
N
O
Ph
Me
F
F
19F NMR CDCl3 471 MHz
f1 (ppm)

***cis*-4-(2,2-difluorovinyl)-3-methyl-3-phenyl-1-(3-phenylpropyl)pyrrolidin-2-one (*cis*-3w)**

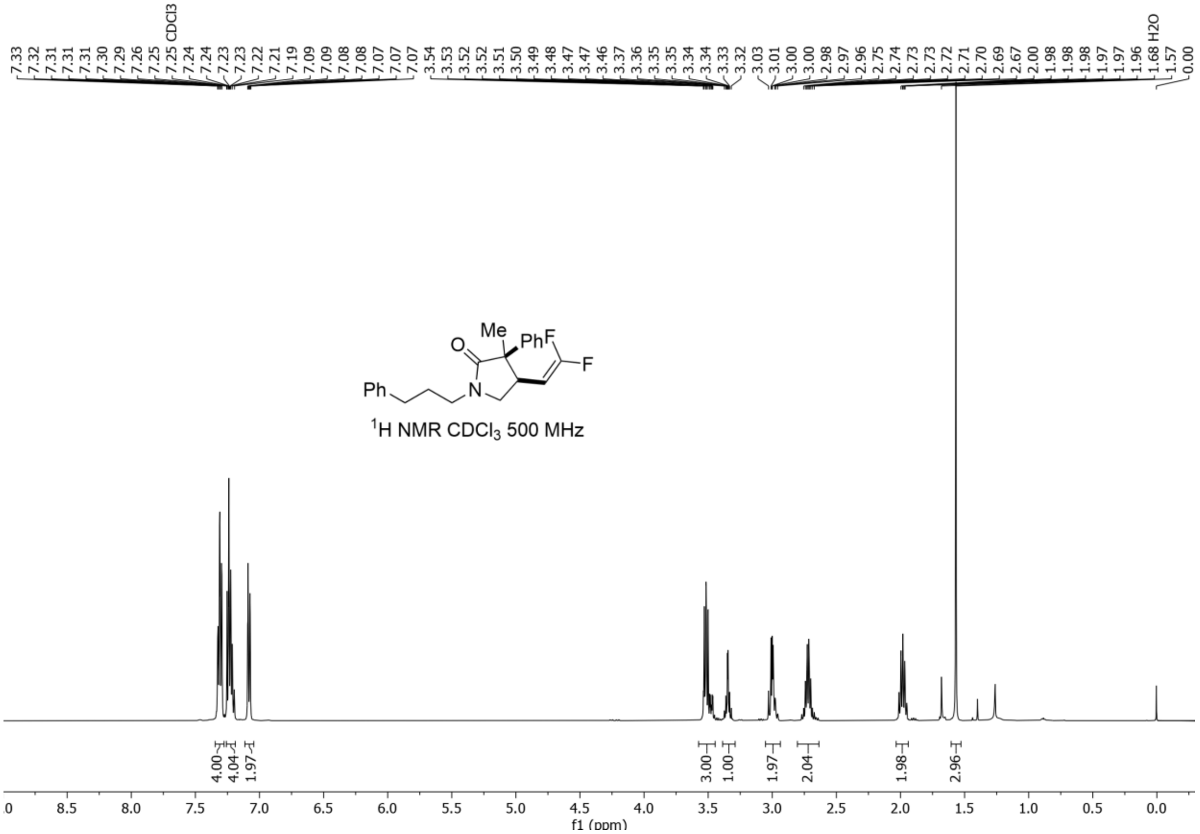

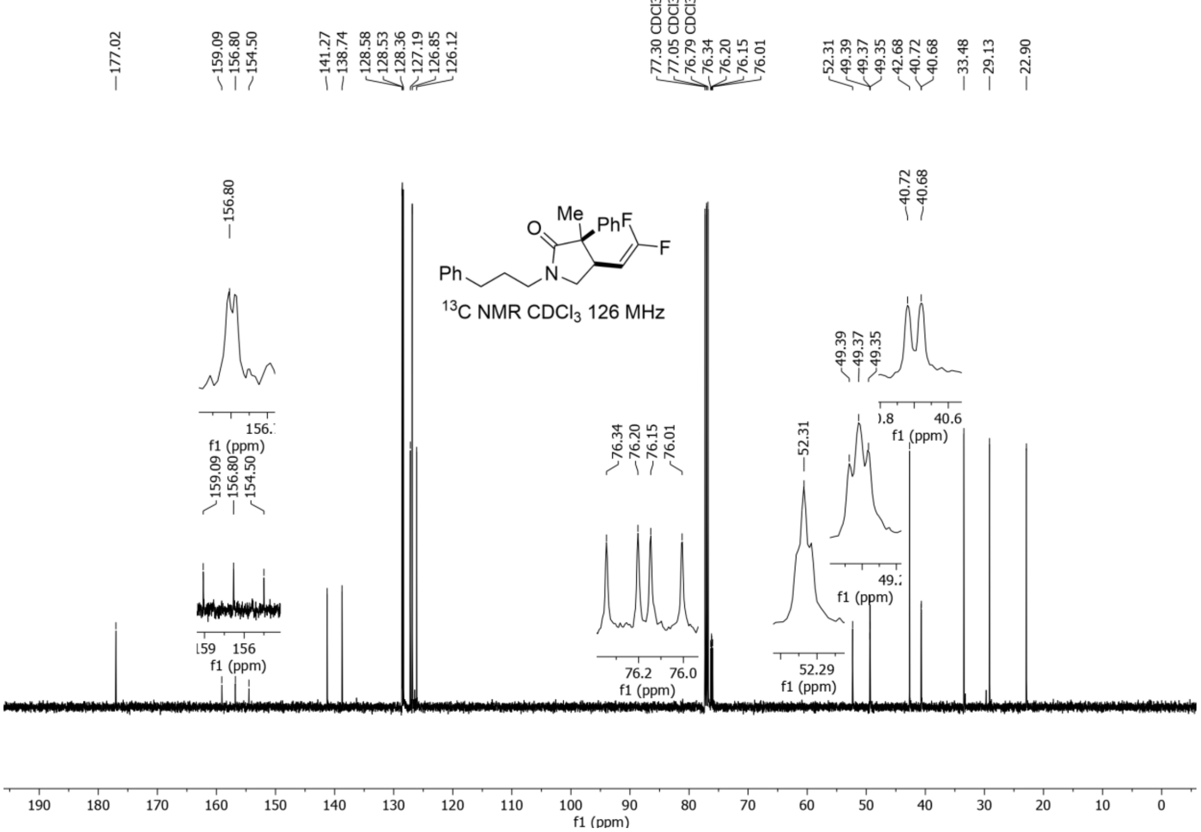

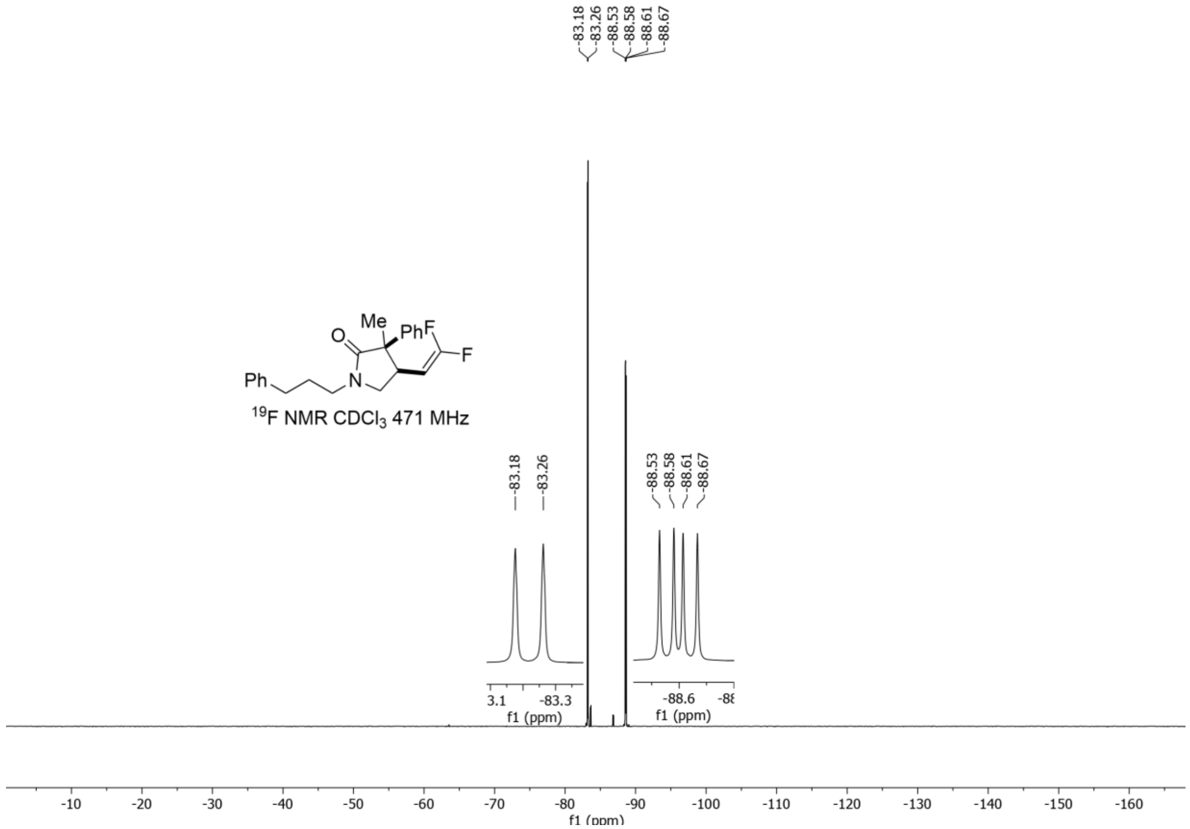

***cis*-4-(2,2-difluorovinyl)-1-hexyl-3-methyl-3-phenylpyrrolidin-2-one (*cis*-3x)**

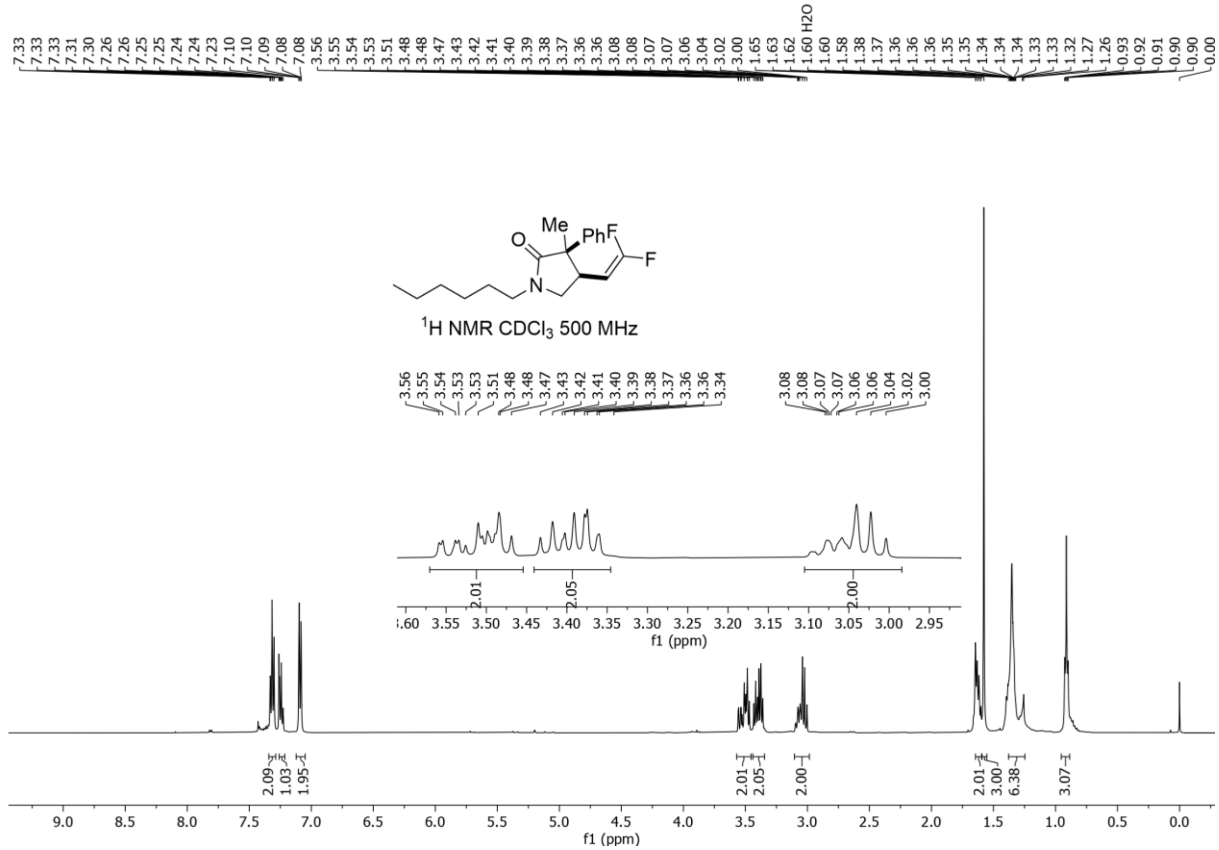

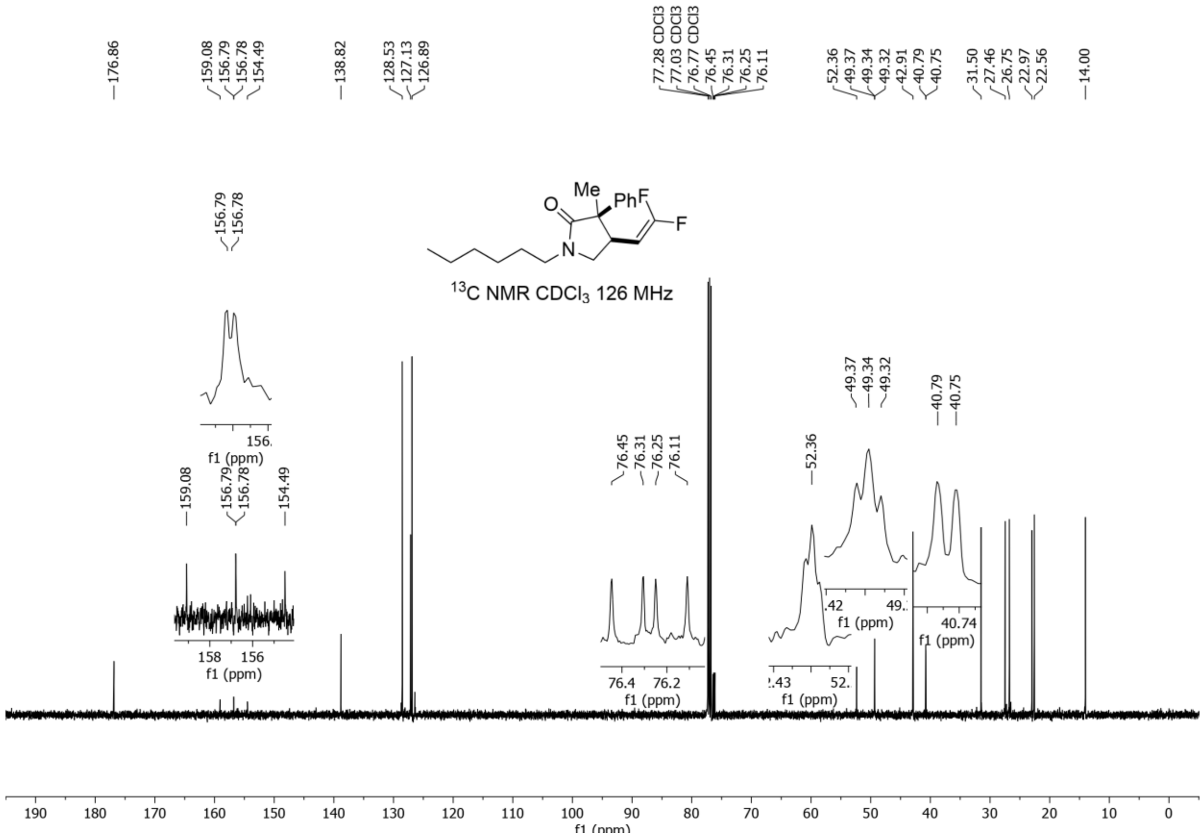

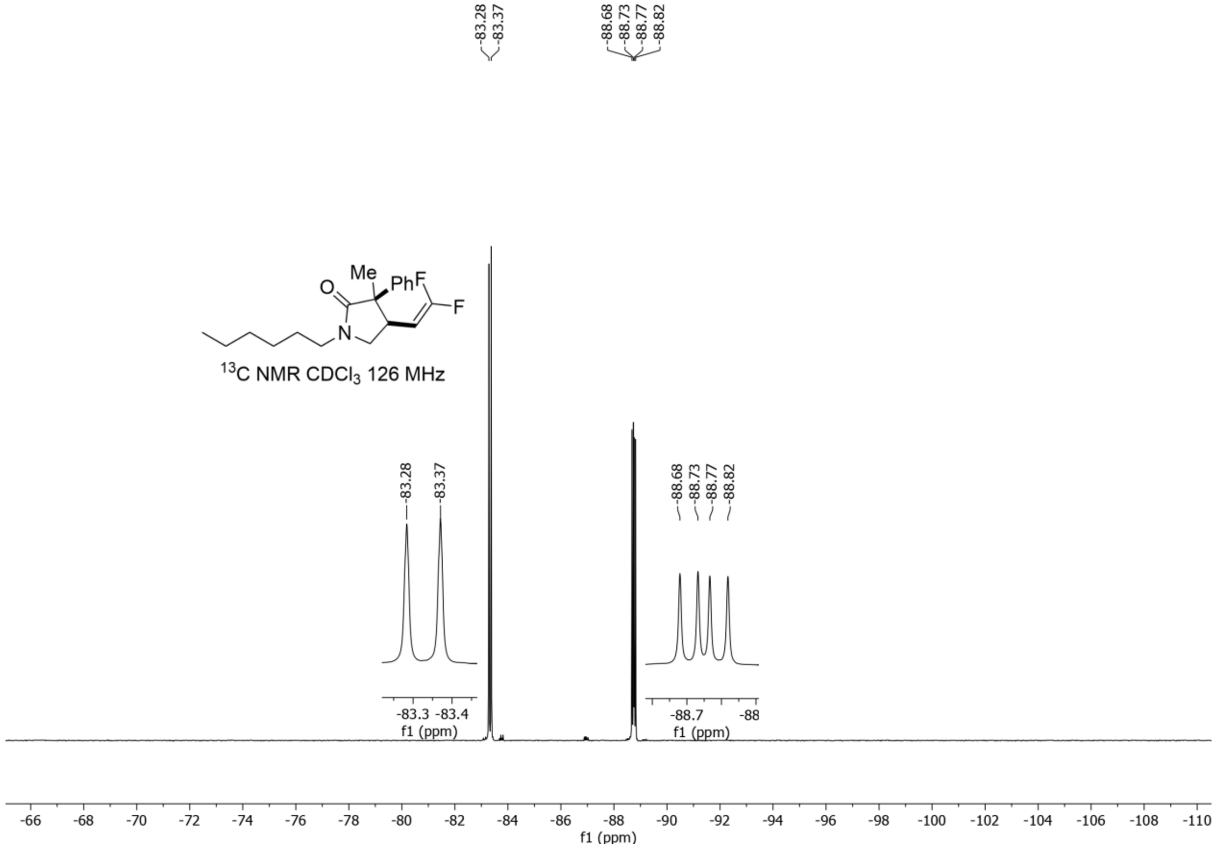

***cis*-4-(2,2-difluorovinyl)-3-methyl-1-neopentyl-3-phenylpyrrolidin-2-one (*cis*-3y)**

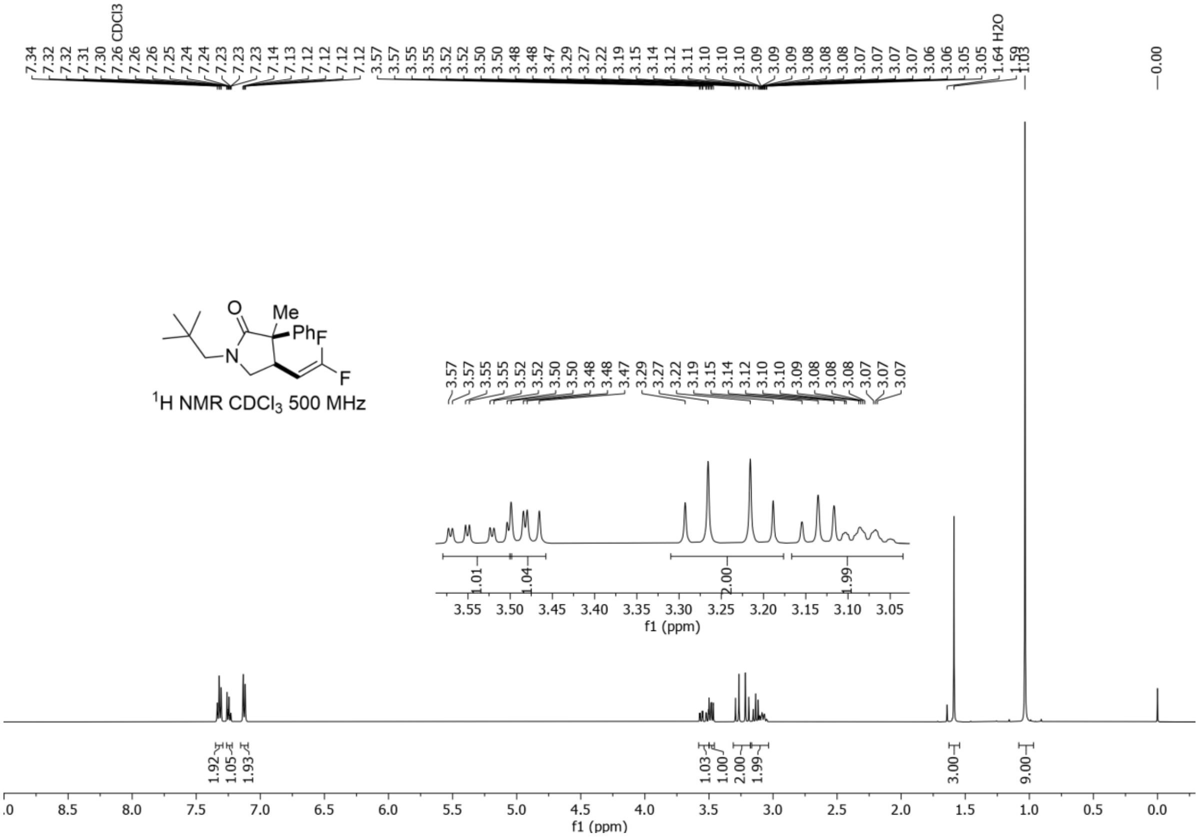

7.34 7.32 7.32 7.31 7.30 7.26 CDCl3 7.26 7.26 7.25 7.24 7.24 7.23 7.23 7.23 7.14 7.13 7.12 7.12 7.12 7.12 3.57 3.57 3.55 3.55 3.52 3.52 3.50 3.50 3.48 3.48 3.47 3.29 3.27 3.22 3.19 3.15 3.14 3.12 3.11 3.10 3.10 3.10 3.09 3.09 3.09 3.08 3.08 3.08 3.07 3.07 3.07 3.07 3.06 3.06 3.05 3.05 1.64 H2O 1.59 1.03
0.00
O
Me
Ph
F
N
F
1H NMR CDCl3 500 MHz
1.01
1.04
2.00
1.99
f1 (ppm)
1.92
1.05
1.93
1.03
1.00
2.00
1.99
3.00
9.00
f1 (ppm)

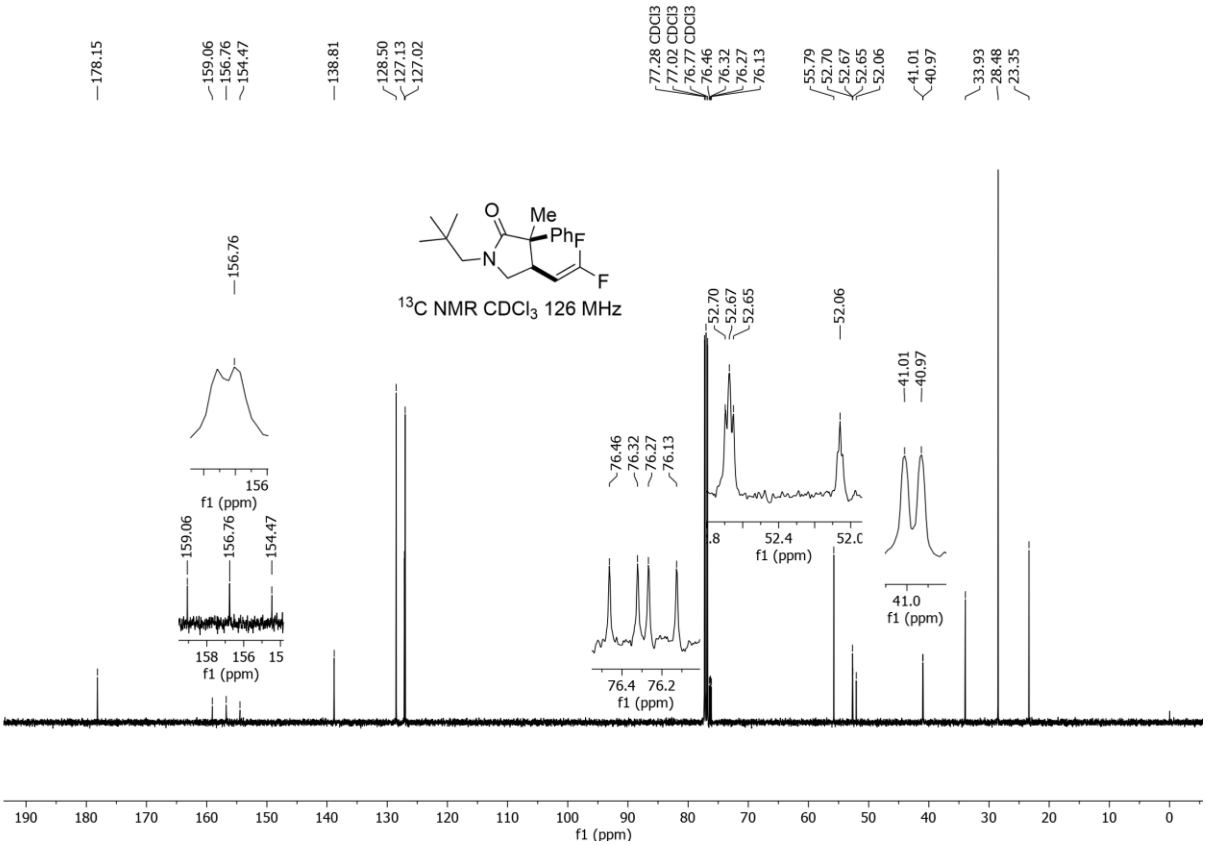

178.15
159.06 156.76 154.47
138.81
128.50 127.13 127.02
77.28 CDCl3 77.02 CDCl3 76.77 CDCl3 76.46 76.32 76.27 76.13
55.79 52.70 52.67 52.65 52.06
41.01 40.97
33.93 28.48 23.35
O
Me
Ph
F
N
F
13C NMR CDCl3 126 MHz
f1 (ppm)

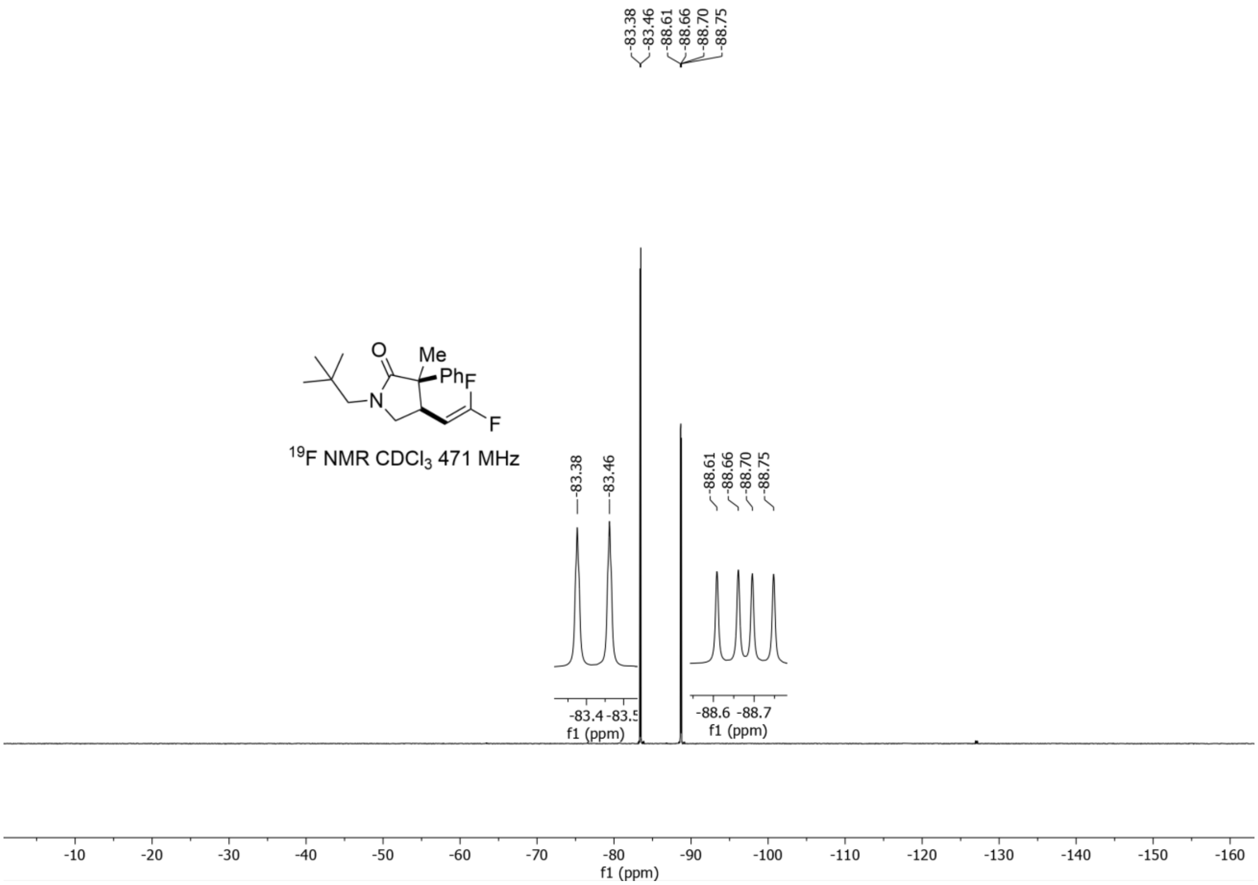

***trans*-4-(2,2-difluorovinyl)-3-methyl-1-neopentyl-3-phenylpyrrolidin-2-one (*trans*-3y)**

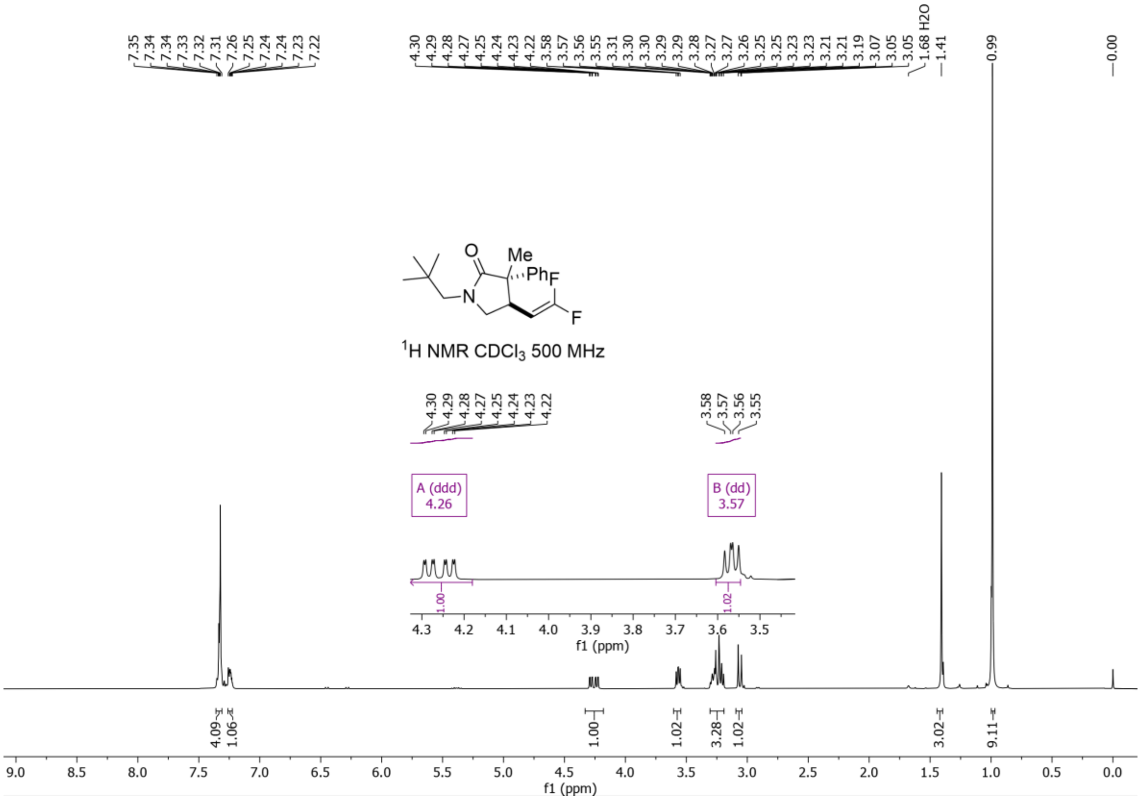

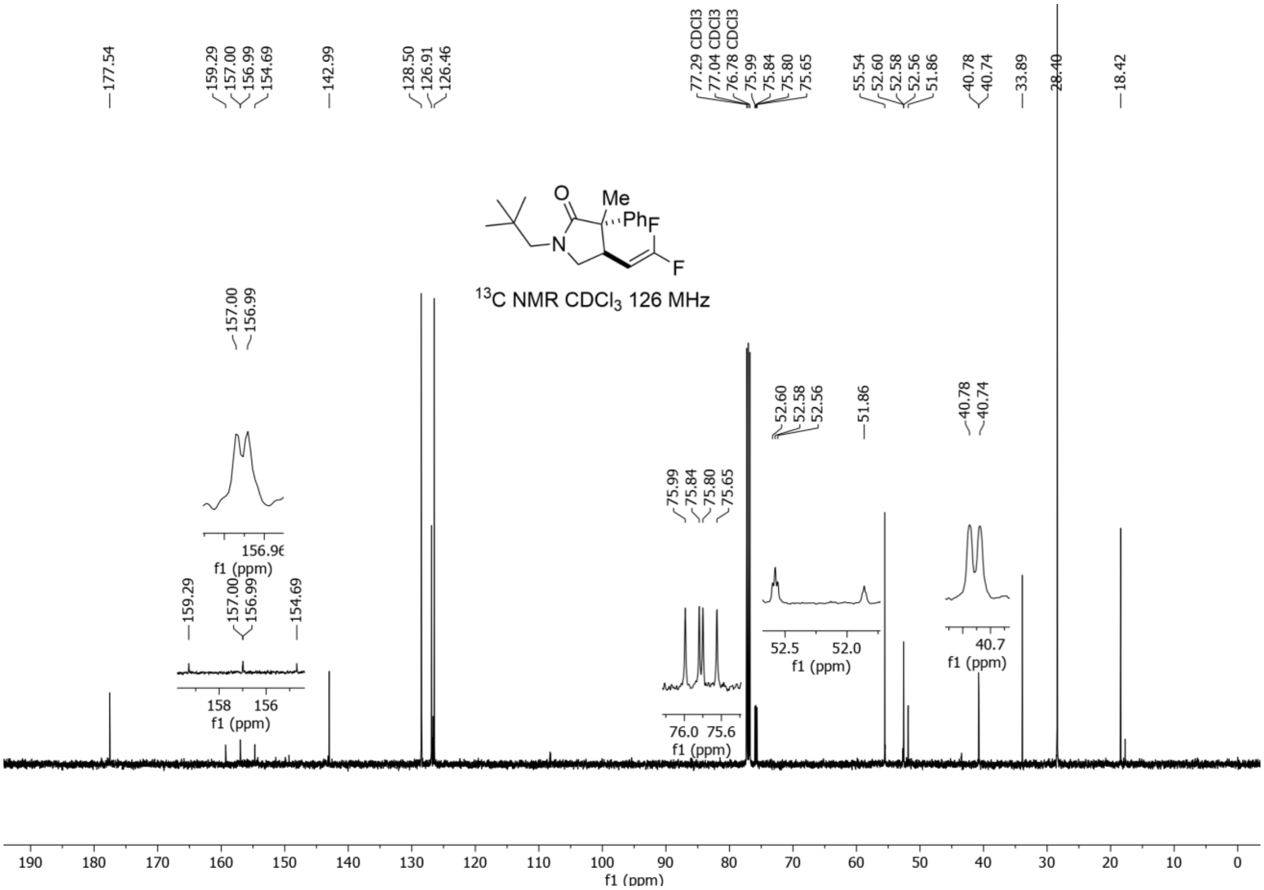

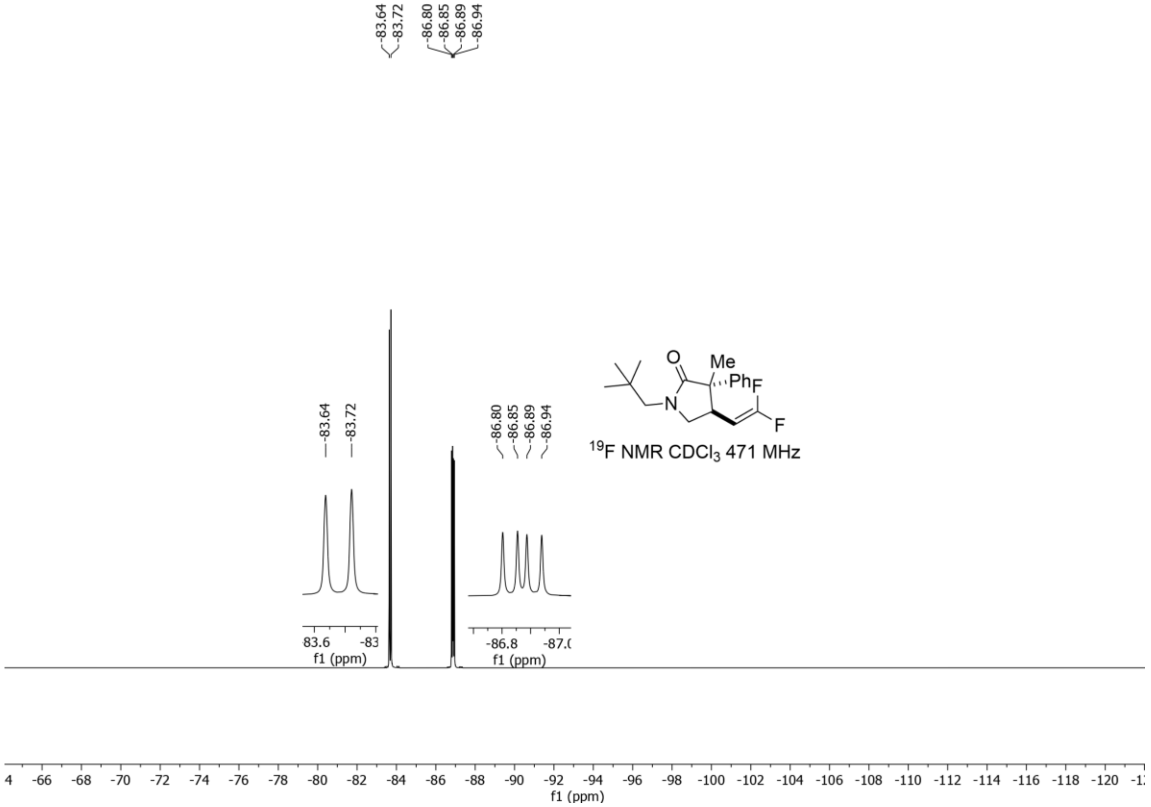

***cis*-4-(2,2-difluoroethyl)-3-methyl-1,3-diphenylpyrrolidin-2-one (4)**

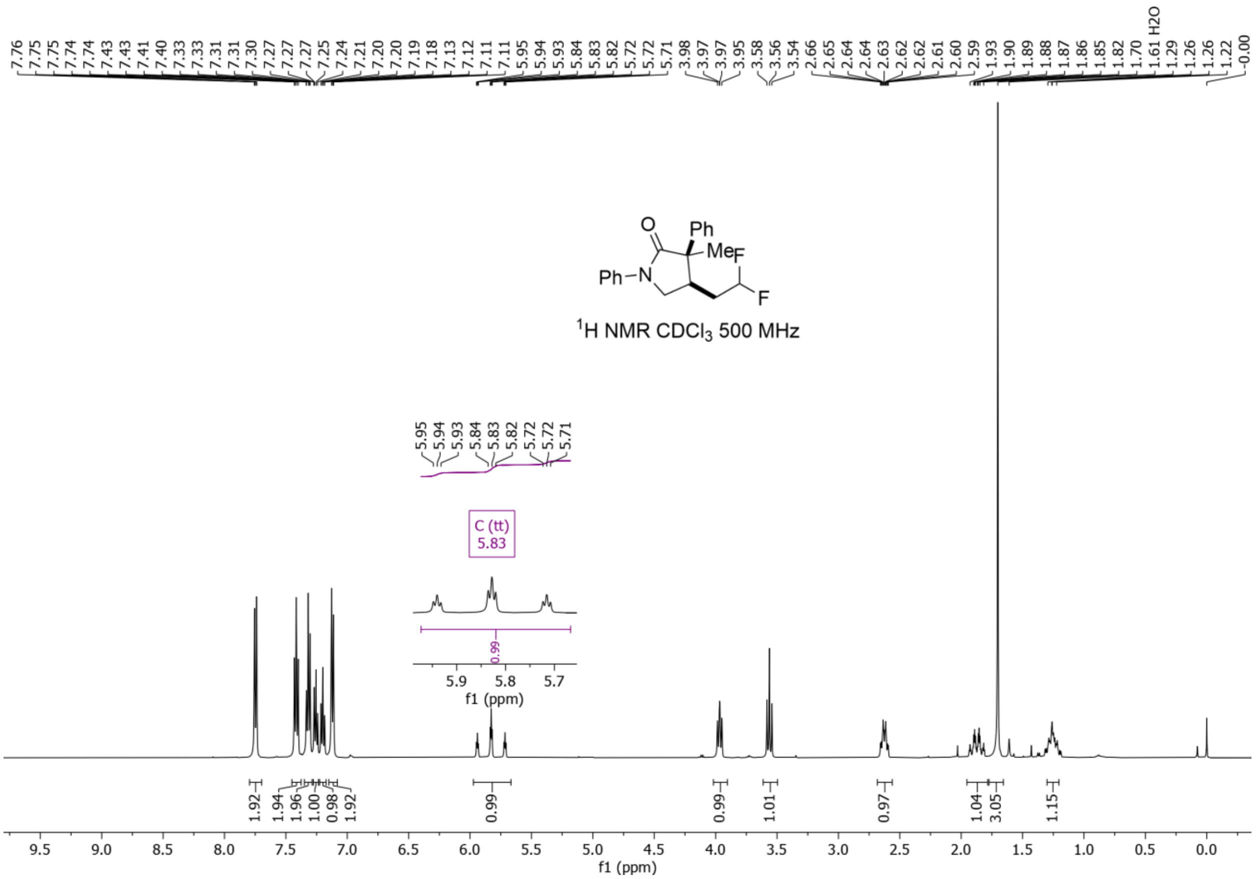

7.76 7.75 7.75 7.74 7.74 7.43 7.43 7.41 7.40 7.33 7.33 7.31 7.30 7.27 7.27 7.27 7.25 7.24 7.21 7.20 7.19 7.18 7.13 7.12 7.11 7.11 5.95 5.94 5.93 5.84 5.83 5.82 5.72 5.72 5.71 3.98 3.97 3.97 3.95 3.58 3.56 3.54 2.66 2.65 2.64 2.63 2.62 2.62 2.61 2.60 2.59 1.93 1.90 1.89 1.88 1.87 1.86 1.85 1.82 1.70 1.61 H2O 1.29 1.26 1.26 1.22 -0.00
O
Ph
Me
F
Ph
N
F
1H NMR CDCl3 500 MHz
C (tt)
5.83
0.99
f1 (ppm)
1.92 1.94 1.96 1.00 0.98 1.92 0.99 0.99 1.01 0.97 1.04 3.05 1.15

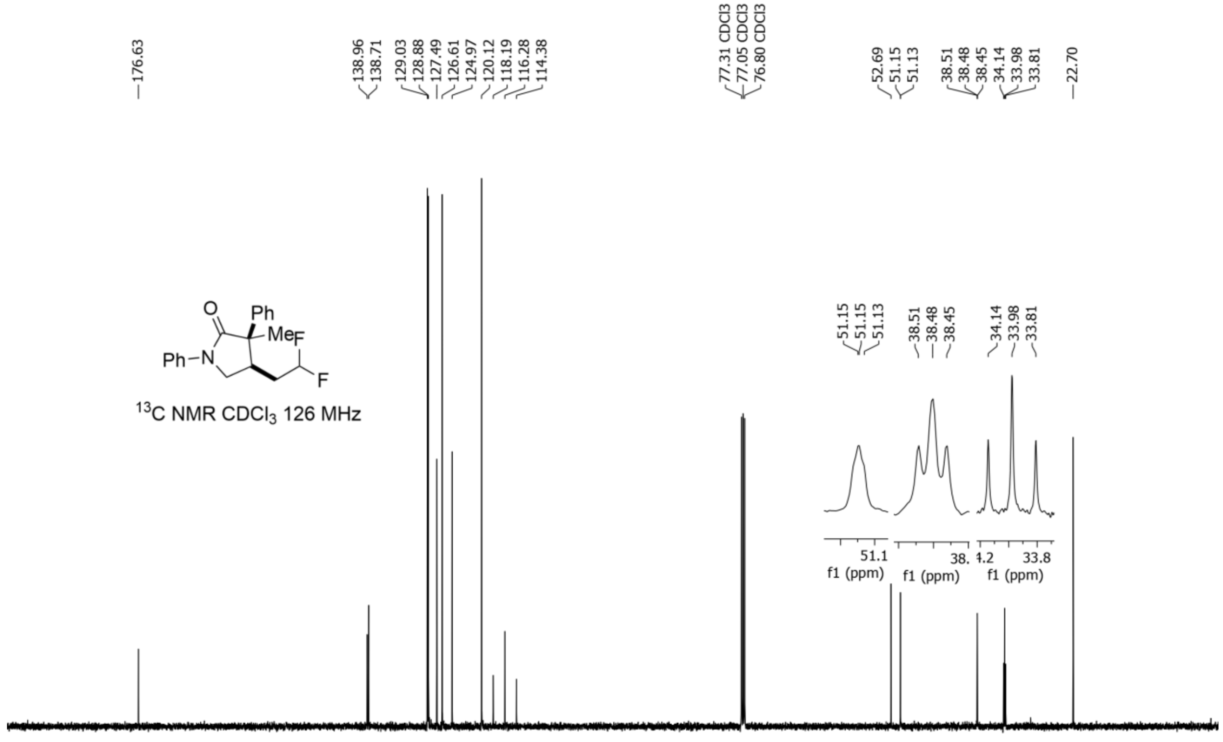

176.63
138.96 138.71 129.03 128.88 127.49 126.61 124.97 120.12 118.19 116.28 114.38
77.31 CDCl3 77.05 CDCl3 76.80 CDCl3
52.69 51.15 51.13 38.51 38.48 38.45 34.14 33.98 33.81 22.70
O
Ph
Me
F
Ph
N
F
13C NMR CDCl3 126 MHz
51.15 51.15 51.13
38.51 38.48 38.45
34.14 33.98 33.81
f1 (ppm)

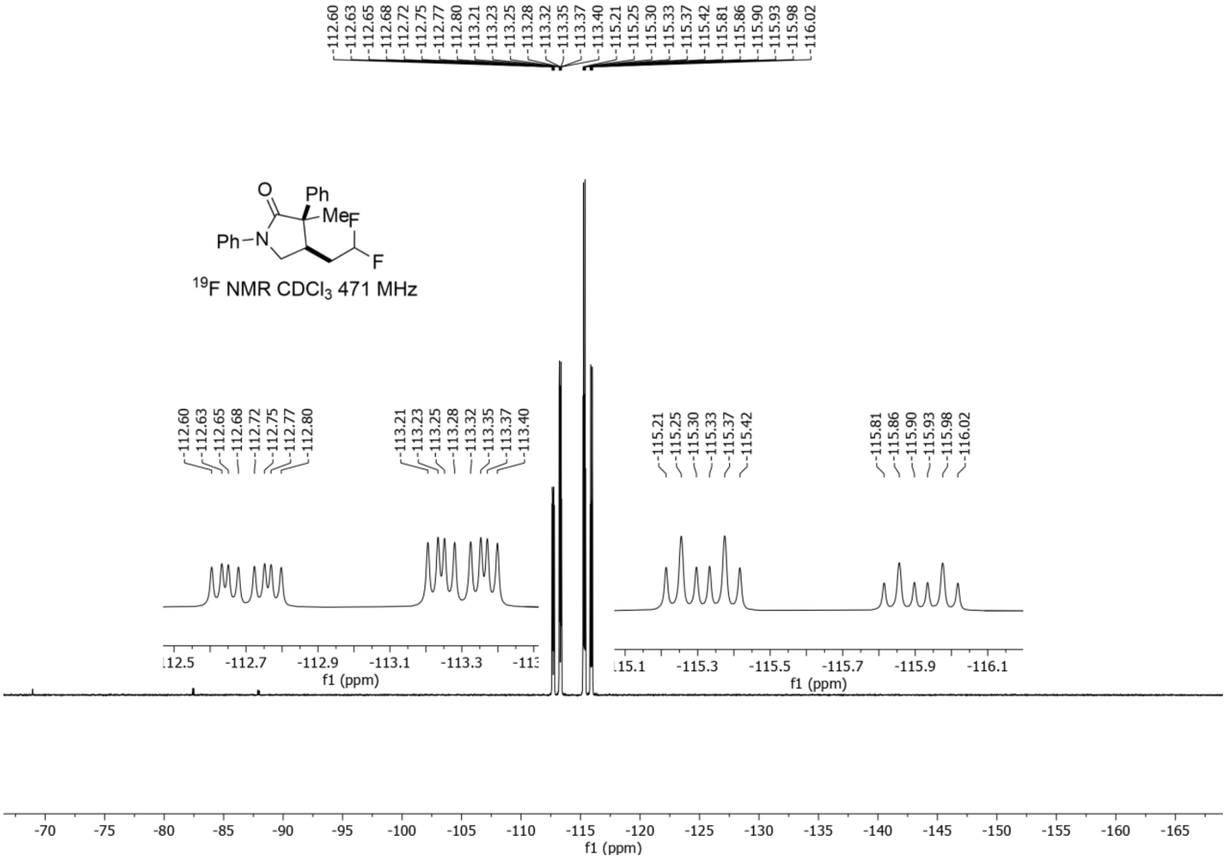

***cis*-(*Z*)-4-(2-fluorovinyl)-3-methyl-1,3-diphenylpyrrolidin-2-one (5)**

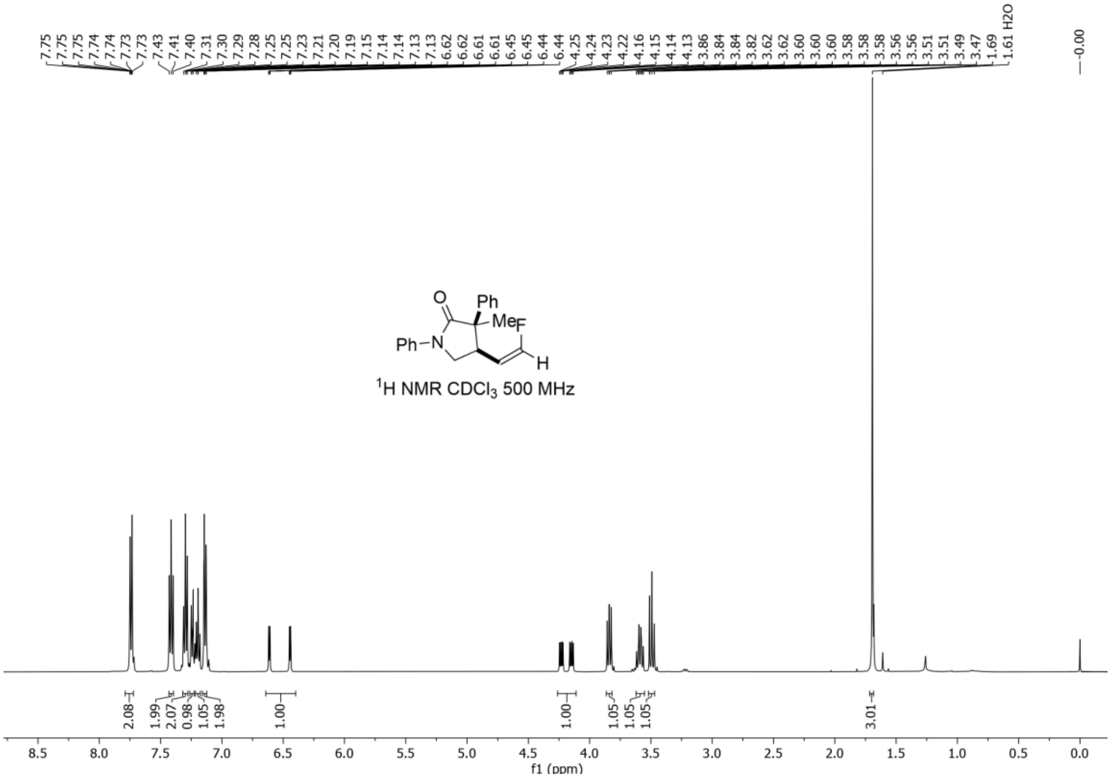

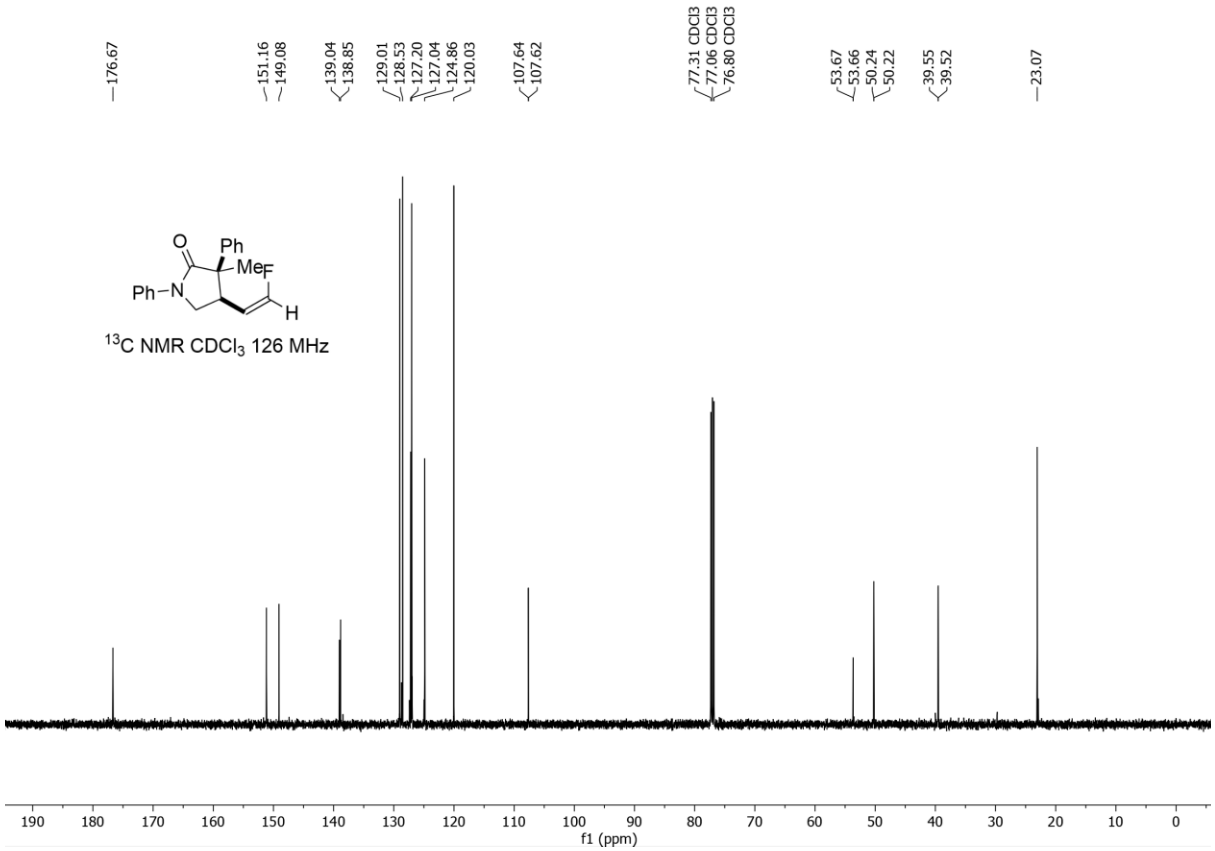

176.67
151.16
149.08
139.04
138.85
129.01
128.53
127.20
127.04
124.86
120.03
107.64
107.62
77.31 CDCl3
77.06 CDCl3
76.80 CDCl3
53.67
53.66
50.24
50.22
39.55
39.52
23.07
Ph
Me
F
Ph–N
O
H
13C NMR CDCl3 126 MHz
f1 (ppm)

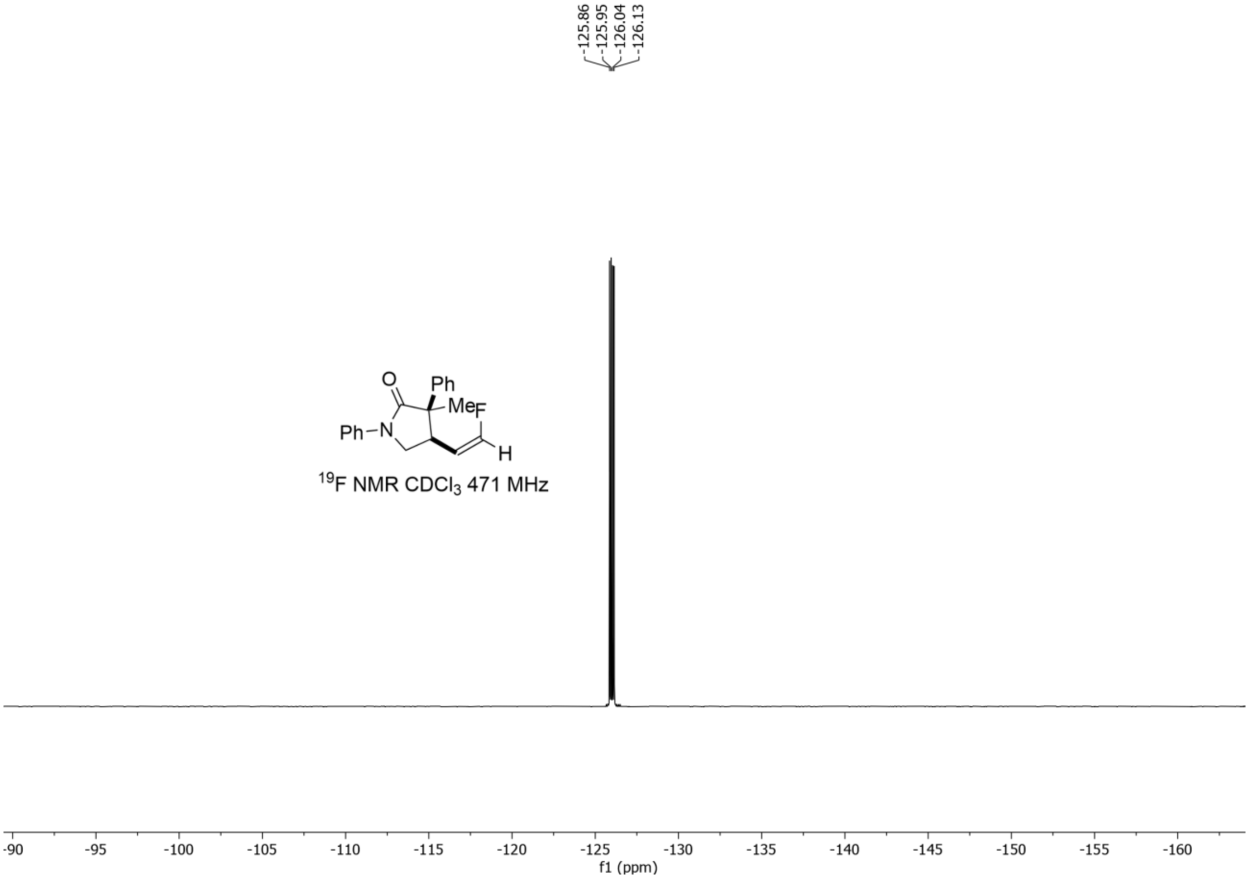

-125.86
-125.95
-126.04
-126.13
Ph
Me
F
Ph–N
O
H
19F NMR CDCl3 471 MHz
f1 (ppm)

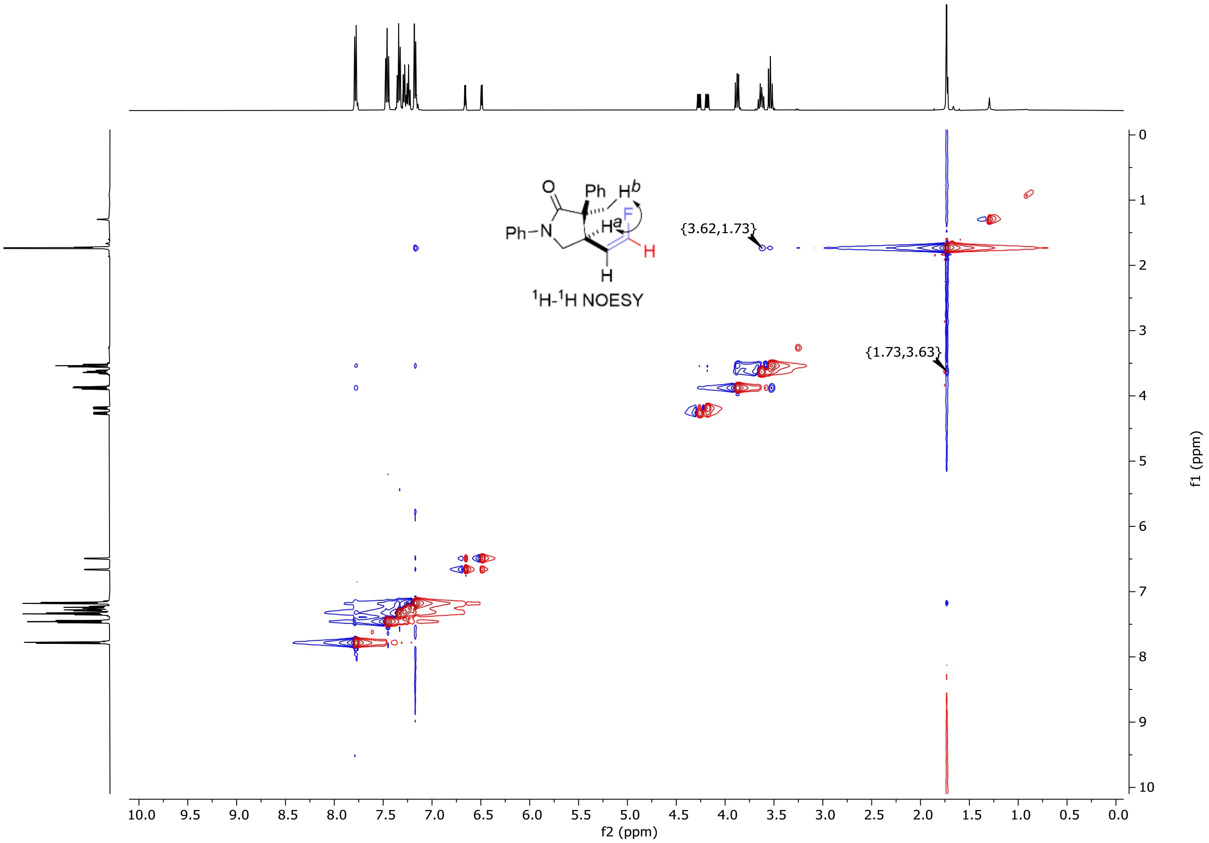

4-(2-azido-2,2-difluoro-1-iodoethyl)-3-methyl-1,3-diphenylpyrrolidin-2-one (6)

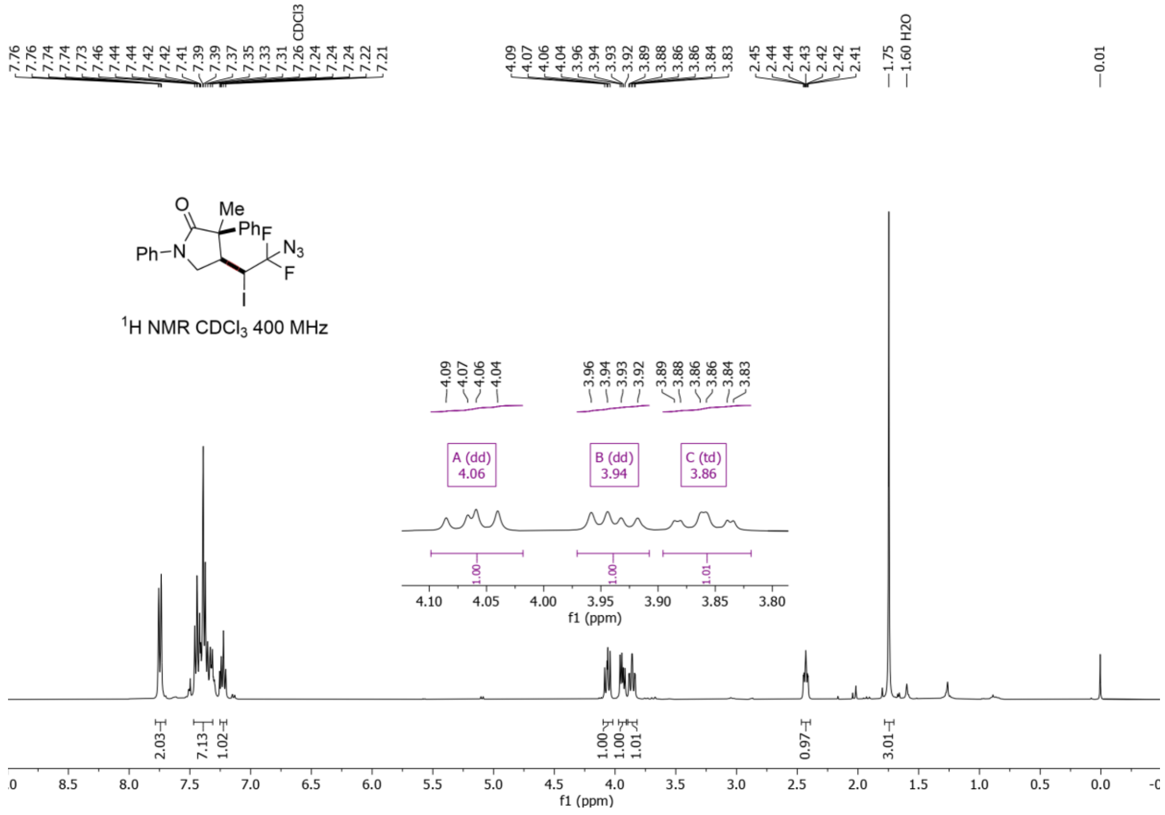

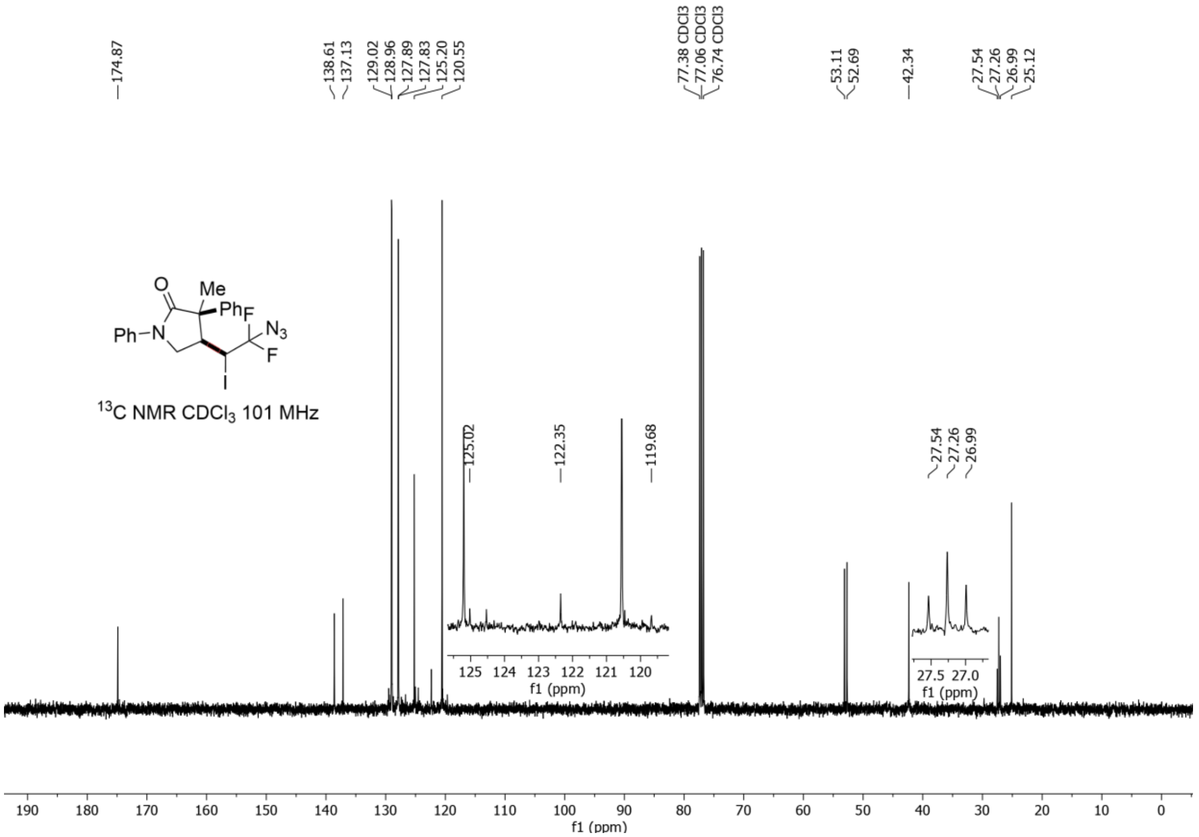

174.87
138.61
137.13
129.02
128.96
127.89
127.83
125.20
120.55
77.38 CDCl3
77.06 CDCl3
76.74 CDCl3
53.11
52.69
42.34
27.54
27.26
26.99
25.12
Me
O
Ph
F
N3
Ph
N
F
I
13C NMR CDCl3 101 MHz
125.02
122.35
119.68
f1 (ppm)
f1 (ppm)

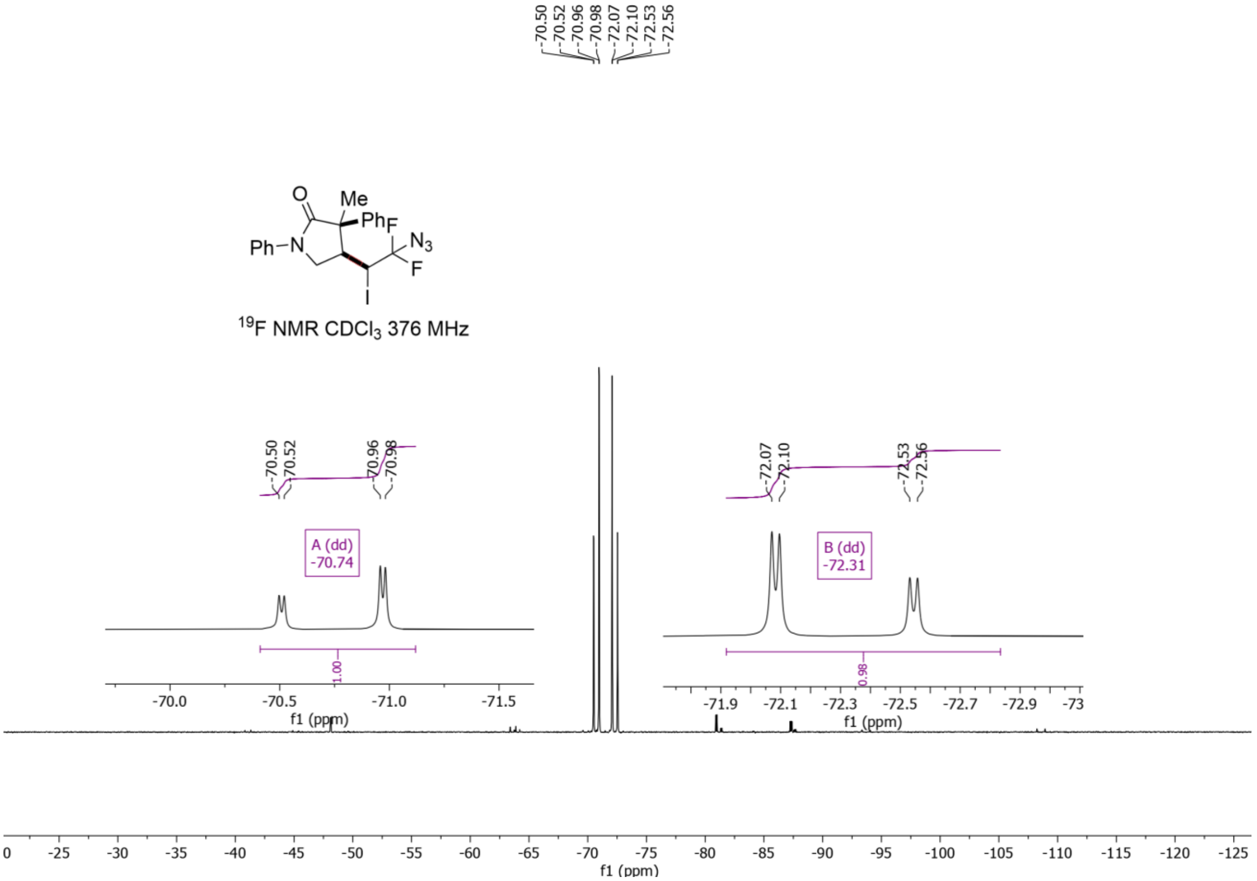

-70.50
-70.52
-70.96
-70.98
-72.07
-72.10
-72.53
-72.56
19F NMR CDCl3 376 MHz
A (dd)
-70.74
B (dd)
-72.31
1.00
0.98
f1 (ppm)
f1 (ppm)
f1 (ppm)

***cis*-3-methyl-4-(2-morpholino-2-oxoethyl)-1,3-diphenylpyrrolidin-2-one (7)**

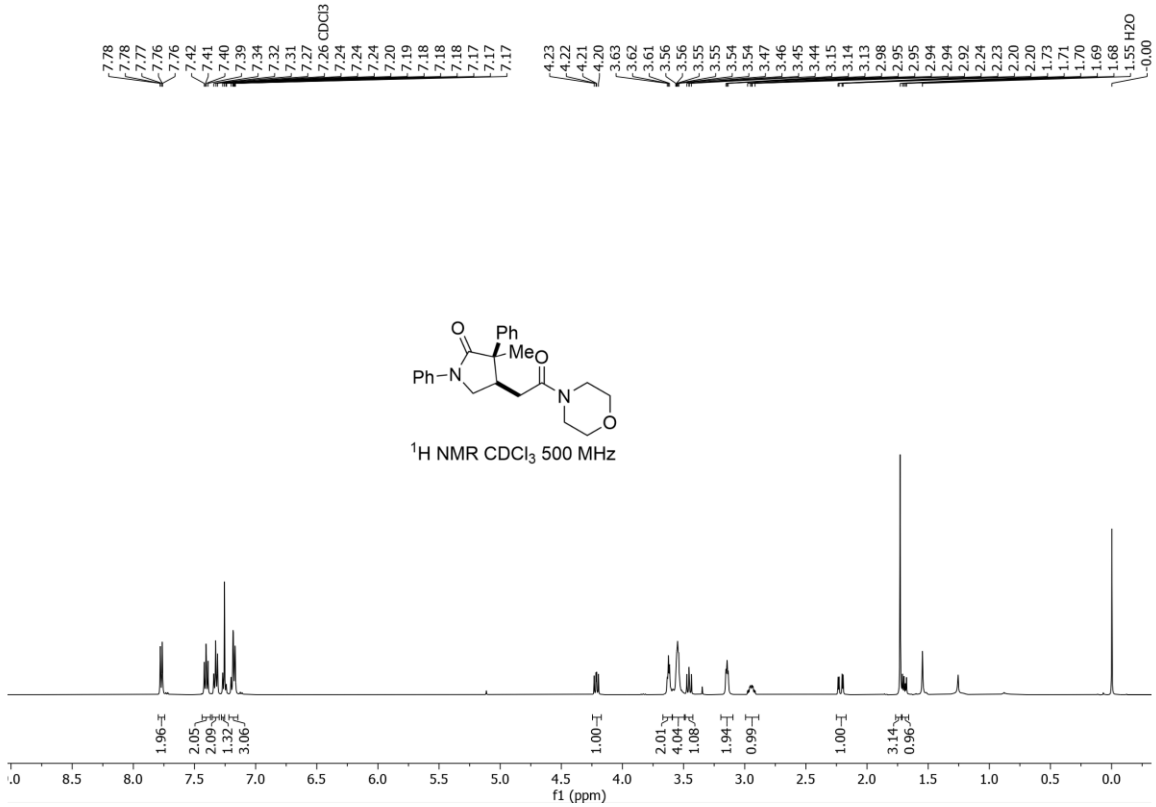

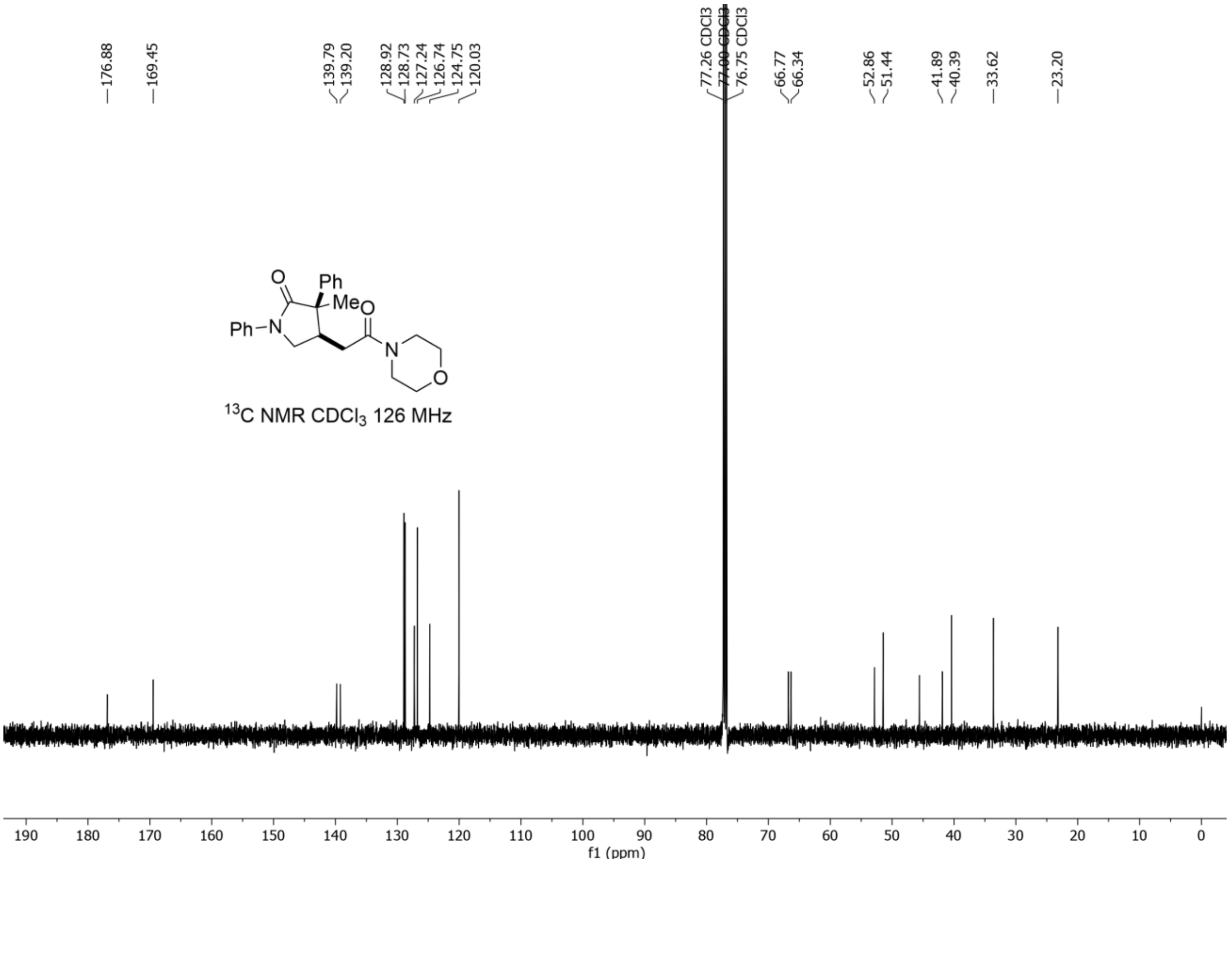

3-methyl-1,3-diphenyl-4-(2,2,2-trifluoro-1-((2,2,6,6-tetramethylpiperidin-1-yl)oxy)ethyl)pyrrolidin-2-one (11)

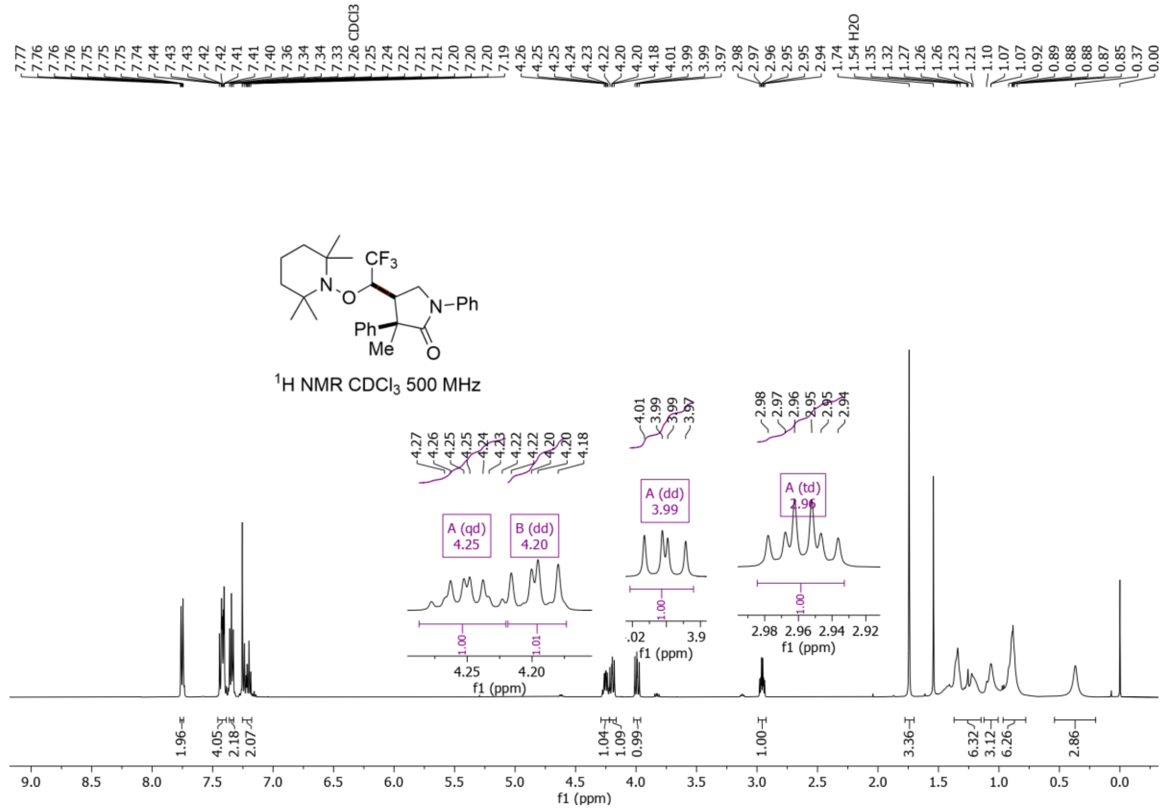

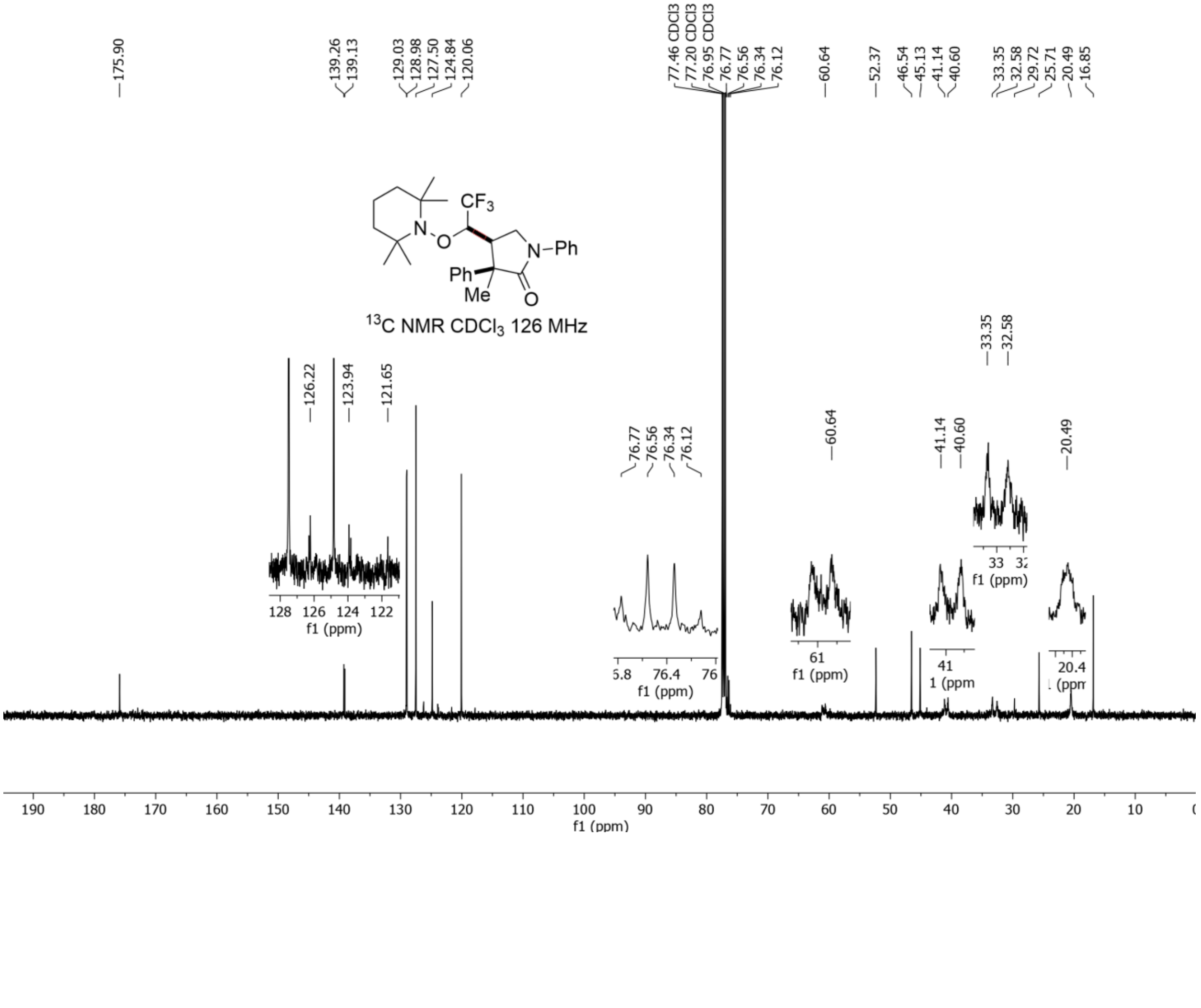

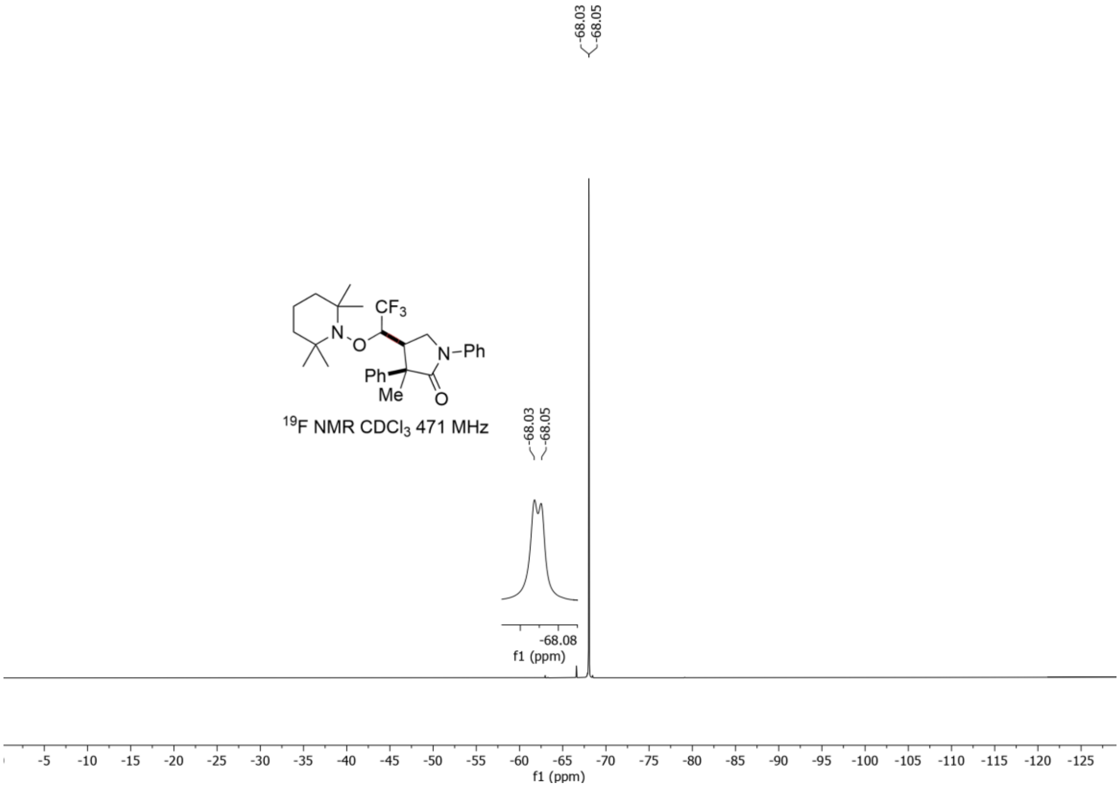

(*E*)-*N*-(but-2-en-1-yl)-N,2-diphenylacrylamide (13)

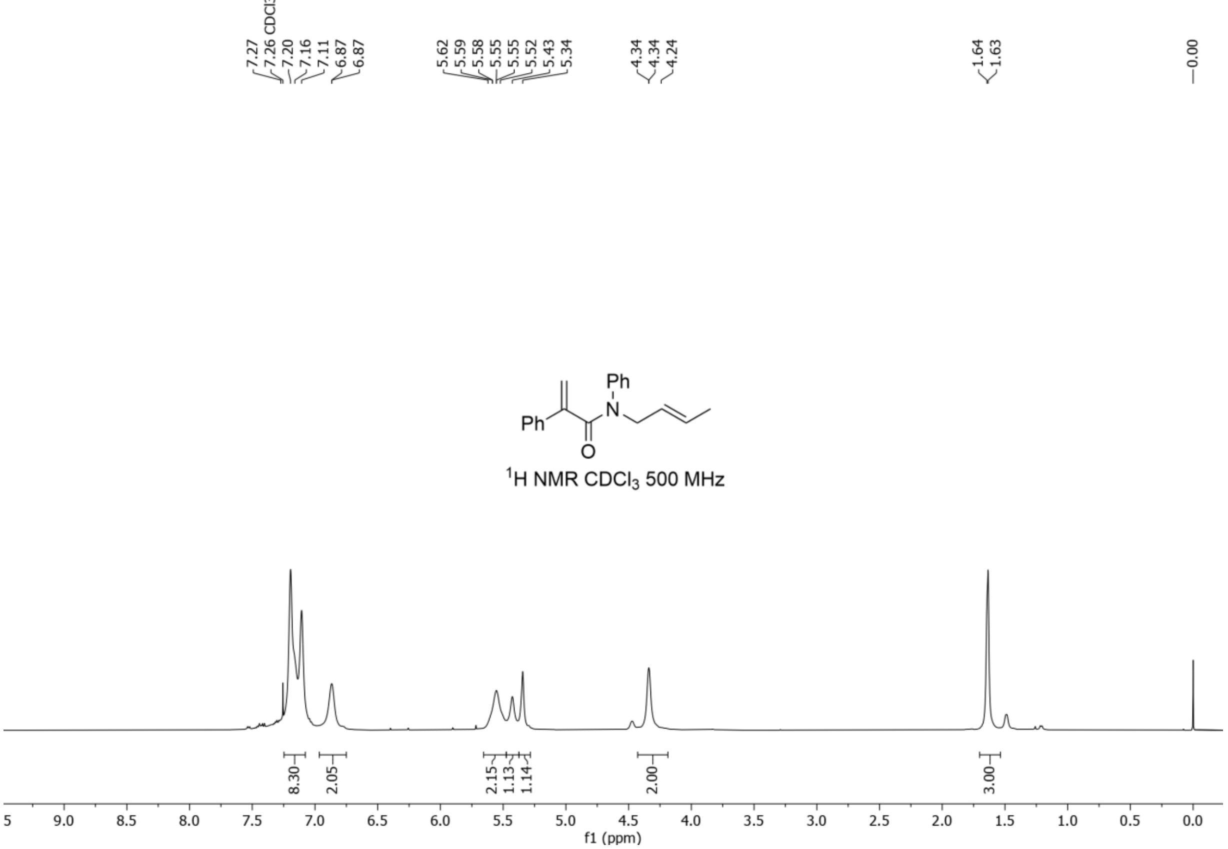

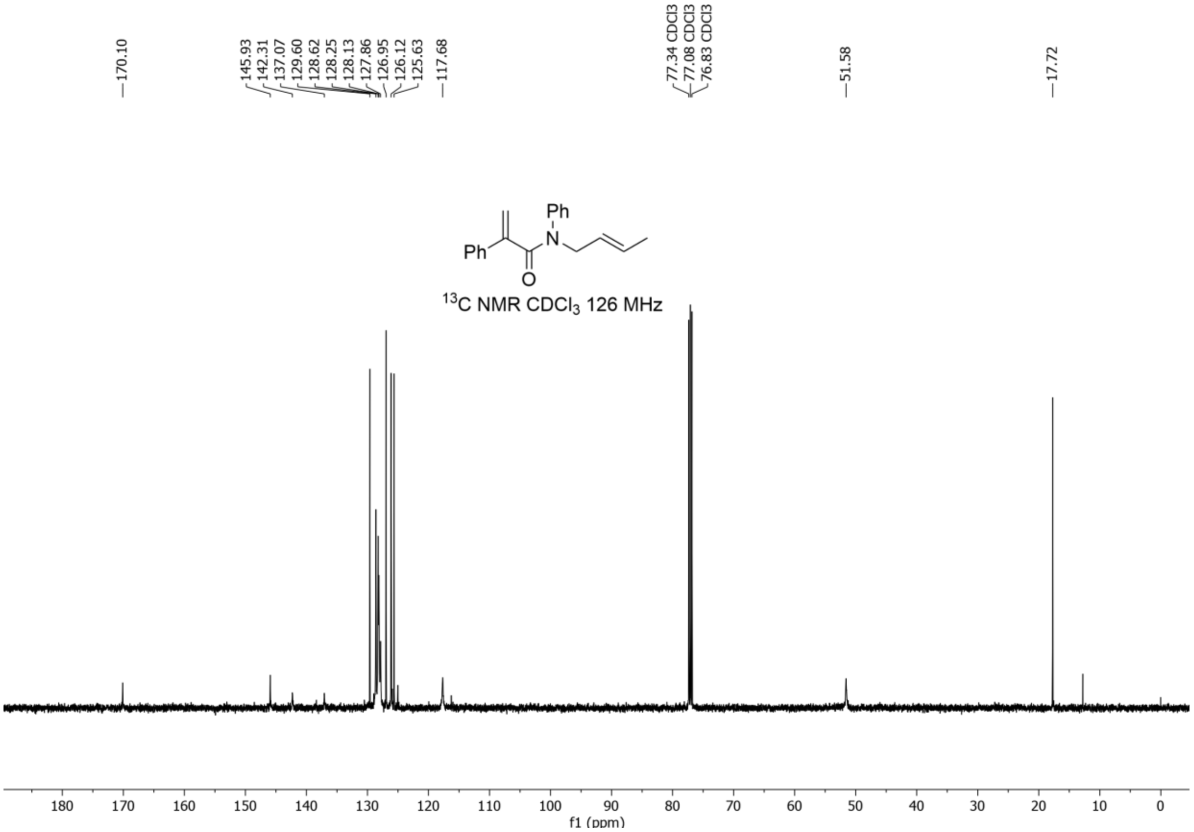

***(E)-N*-(but-2-en-1-yl)-*N*,2-diphenylpropanamide (14)**

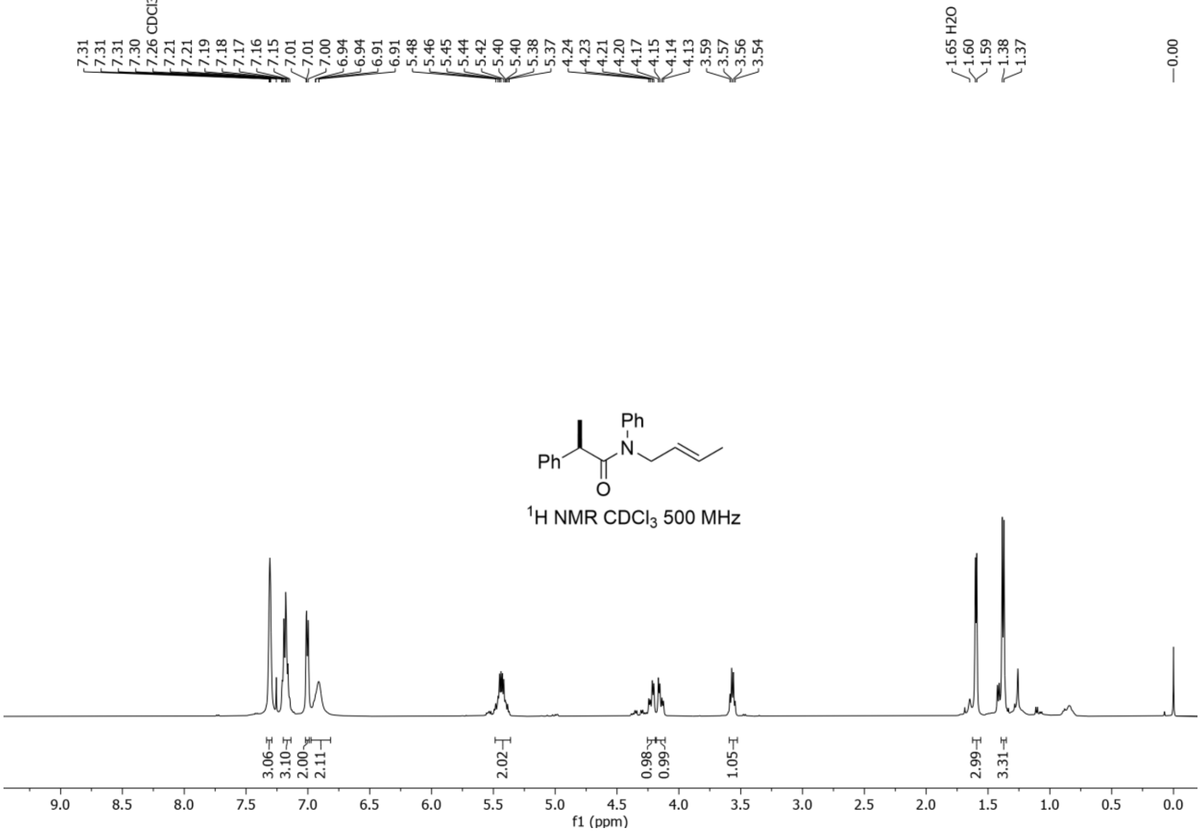

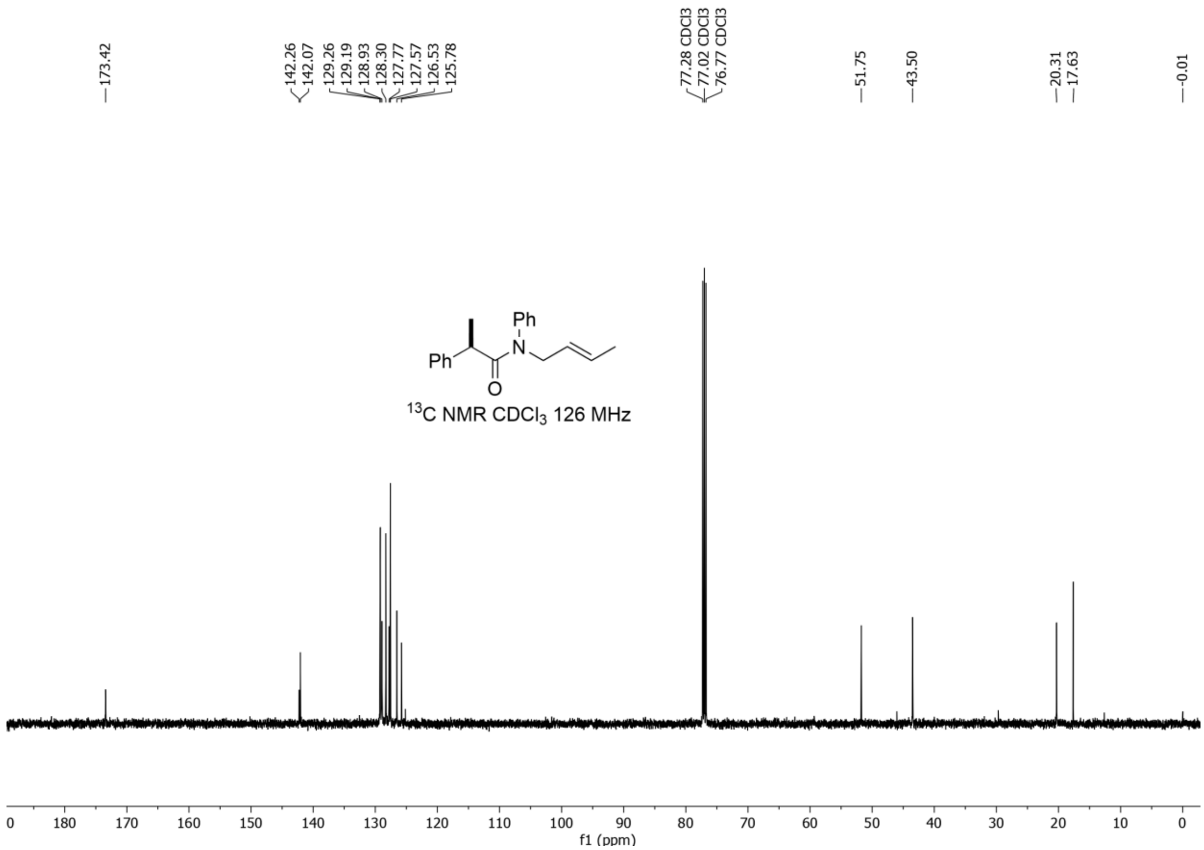
173.42
142.26
142.07
129.26
129.19
128.93
128.30
127.77
127.57
126.53
125.78
77.28 CDCl3
77.02 CDCl3
76.77 CDCl3
51.75
43.50
20.31
17.63
-0.01
Ph
N
Ph
O
^{13}C NMR CDCl$_3$ 126 MHz
f1 (ppm)
